# Phylogenetic characterization and promoter expression analysis of a novel hybrid protein disulfide isomerase/cargo receptor subfamily unique to plants and chromalveolates

**DOI:** 10.1007/s00438-015-1106-7

**Published:** 2015-08-25

**Authors:** Christen Y. L. Yuen, Katharine Wong, David A. Christopher

**Affiliations:** Department of Molecular Biosciences and Bioengineering, University of Hawaii, 1955 East-West Rd., Ag. Science Rm 218, Honolulu, HI 96822 USA

**Keywords:** Endoplasmic reticulum, Cargo receptor, Protein disulfide isomerase, Golgi apparatus

## Abstract

Protein disulfide isomerases (PDIs) play critical roles in protein folding by catalyzing the formation and rearrangement of disulfide bonds in nascent secretory proteins. There are six distinct PDI subfamilies in terrestrial plants. A unique feature of PDI-C subfamily members is their homology to the yeast retrograde (Golgi-to-endoplasmic reticulum) cargo receptor proteins, Erv41p and Erv46p. Here, we demonstrate that plant Erv41p/Erv46p-like proteins are divided into three subfamilies: ERV-A, ERV-B and PDI-C, which all possess the N-proximal and C-proximal conserved domains of yeast Erv41p and Erv46p. However, in PDI-C isoforms, these domains are separated by a thioredoxin domain. The distribution of PDI-C isoforms among eukaryotes indicates that the PDI-C subfamily likely arose through an ancient exon-shuffling event that occurred before the divergence of plants from stramenopiles and rhizarians. *Arabidopsis* has three PDI-C genes: *PDI7*, *PDI12*, and *PDI13*. *PDI12*- and *PDI13*-promoter: β-glucuronidase (GUS) gene fusions are co-expressed in pollen and stipules, while *PDI7* is distinctly expressed in the style, hydathodes, and leaf vasculature. The PDI-C thioredoxin domain active site motif CxxS is evolutionarily conserved among land plants. Whereas PDI12 and PDI13 retain the CxxS motif, PDI7 has a CxxC motif similar to classical PDIs. We hypothesize that *PDI12* and *PDI13* maintain the ancestral roles of PDI-C in *Arabidopsis*, while *PDI7* has undergone neofunctionalization. The unusual PDI/cargo receptor hybrid arrangement in PDI-C isoforms has no counterpart in animals or yeast, and predicts the need for pairing redox functions with cargo receptor processes during protein trafficking in plants and other PDI-C containing organisms.

**Electronic supplementary material:**

The online version of this article (doi:10.1007/s00438-015-1106-7) contains supplementary material, which is available to authorized users.

## Introduction

In eukaryotes, newly synthesized secretory proteins are folded and assembled in the endoplasmic reticulum (ER) before trafficking to the Golgi for key post-translational modifications and sorting to their final destinations. The ER provides an oxidizing environment enriched in protein folding chaperones and enzymes, including members of the large and structurally diverse protein disulfide isomerase (PDI) family. PDIs are characterized by one or more catalytic domains sharing homology to the redox protein, thioredoxin. The classical PDI is a multifunctional enzyme consisting of four modular domains in the arrangement **a-b-b′-a′**, where **a** and **a′** are the thioredoxin domains (Edman et al. 1985), and **b** and **b′** are redox-inactive domains with a thioredoxin-like fold structure (Kemmink et al. 1997). Isoforms of classical PDIs are capable of catalyzing the formation, breakage, or rearrangement of disulfide bonds in a wide range of substrate proteins (Chivers et al. 1998), and can also facilitate protein folding as molecular chaperones (Wang and Tsou 1993; Andème Ondzighi et al. 2008). The activity of classical PDIs is essential in both animals (Winter et al. 2007) and yeast (Scherens et al. 1991).

In terrestrial plants, the PDI family consists of six structurally distinct subfamilies, designated as A, B, C, L, M and S (Selles et al. 2011). Only PDI-L members share the **a-b-b′-a′** domain organization of classical PDIs from animals and yeast. In *Arabidopsis thaliana*, the PDI-L isoform, PDI5, influences embryo development and regulates the timing of programmed cell death by chaperoning and inhibiting cysteine (Cys) proteases during their trafficking from the ER to vacuoles (Andème Ondzighi et al. 2008). Its close paralog, PDI6, is dual-targeted to the ER and chloroplasts, and has been implicated as a modulator of photoinhibition (Wittenberg et al. 2014). Another *Arabidopsis* PDI-L isoform, PDI2, has been shown to interact with both the nuclear transcription factor, maternal effect embryo arrest 8 (MEE8), and the ER-resident molecular chaperone BiP (Cho et al. 2011). PDI2 localizes to both the ER and the nucleus, and has been proposed to traffic to the nucleus without an obvious nuclear localization signal by a piggyback-mechanism through its interaction with MEE8 (Porter et al. 2015). In rice, isoforms of both PDI-L and PDI-M (which are orthologous to the non-classical human PDI, P5) were demonstrated to serve distinct roles in the development of protein bodies (Onda et al. 2011). Members of the PDI-S subfamily are characterized by the domain arrangement **a-a-D**, where the **D** is a unique C-terminal all-helical domain (Freedman 2009). In *Arabidopsis*, expression of truncated versions of PDI11 (the sole member of the PDI-S subfamily in *Arabidopsis*) exhibit disruptions in both pollen tube guidance and embryo sac development; however, true knockouts of the *PDI11* gene do not cause similar phenotypes, and thus the actual function of PDI11 in these processes remains unclear (Wang et al. 2008).

Presently, very little is known concerning the roles of PDI-C isoforms in eukaryotes. Members of the PDI-C subfamily have an unusual domain arrangement that is quite different than classical PDIs, with two predicted transmembrane domains (TMDs), a single catalytic **a**-type domain, and no **b**-type domains (Lu and Christopher 2008). Interestingly, PDI-C isoforms share homology with the *Saccharomyces cerevisiae* ER vesicle (Erv) proteins, Erv41p and Erv46p (d’Aloisio et al. 2010; Selles et al. 2011). In yeast, it has recently been shown that Erv41p and Erv46p, which cycle between the ER and Golgi as a complex, function as a novel cargo receptor for the retrieval of ER proteins lacking the traditional yeast ER retention signal, HDEL (Shibuya et al. 2015). Here, we show that *Arabidopsis* contains three PDI-C isoforms: PDI7, PDI12, and PDI13. To elucidate how PDI-C isoforms are related to Erv41p/Erv46p, we examined the structural similarities and phylogenetic relationships between PDI-C isoforms and other homologs of Erv41p and Erv46p. Furthermore, we analyzed the promoter expression patterns of the three *Arabidopsis* PDI-C genes to gain insight into their potential physiological functions. Our analyses revealed that PDI-C isoforms have a novel domain arrangement, which places a PDI catalytic domain between the conserved N-terminal endoplasmic reticulum–Golgi intermediate compartment (ERGIC-N) domain and coat protein-complex II (COPII)-coated Erv domain of Erv41p/Erv46p-like cargo receptor proteins. No apparent PDI-C ortholog exists among the PDI family of yeast or humans. Thus, PDI-C represents a new class of hybrid PDI-like and cargo receptor-like proteins that are predicted to have novel functions reflective of its unique domain configuration.

## Materials and methods

### Identification of protein homologs and nomenclature

To identify Erv41p/Erv46-like proteins from *Arabidopsis*, BLAST (Basic Local Alignment Search Tool) searches were performed against the TAIR10 protein database on The *Arabidopsis* Information Resource (TAIR) website (https://www.Arabidopsis.org/). Identical results were obtained using the amino acid sequences of either *S. cerevisiae* Erv41p or Erv46p as the BLAST search query sequence. Similar searches were performed against both the National Center for Biotechnology Information (NCBI) non-redundant (nr) protein sequence database (http://blast.ncbi.nlm.nih.gov/Blast.cgi) and the Phytozome v10 (http://phytozome.jgi.doe.gov) protein databases to identify putative homologs of Erv41p and Erv46p among the plant species presented in Table 1, with the exception of *Brassica rapa* cv Chiifu-401-42, which was only available in the NCBI nr database, and *Klebsormidium flaccidum*, which was searched at the *K. flaccidum* Genome Project website: (http://www.plantmorphogenesis.bio.titech.ac.jp/~algae_genome_project/klebsormidium). Whenever possible, incomplete or incorrectly annotated protein sequences were corrected based on available expressed sequence tag (EST) sequences. All sequences and their corresponding accession numbers are provided in Online Resource 1, with alterations to the original source sequences highlighted in yellow.

**Table 1:** *Arabidopsis* PDI family members and their active site sequences

Subfamily	AGI identifier	Lu and Christopher (2008)	Houston et al. (2005)	d’Aloisio et al. (2010)	Selles et al. (2011)	Catalytic domain active site motif(s)
PDI-L	At3g54960	PDI1	PDIL1-3	PDIL2-1	PDI-L2a	CGAC, CGHC
	At5g60640	PDI2	PDIL1-4	PDIL2-2	PDI-L2b	CGHC, CGHC
	At1g52260	PDI3	PDIL1-5	PDIL3-1	PDI-L3a	CARS, CVNC
	At3g16110	PDI4	PDIL1-6	PDIL3-2	PDI-L3b	CARS, CINC
	At1g21750	PDI5	PDIL1-1	PDIL1-1	PDI-L1a	CGHC, CGHC
	At1g77510	PDI6	PDIL1-2	PDIL1-2	PDI-L1b	CGHC, CGHC
PDI-B	At1g35620	PDI8	PDIL5-2	PDIL7-1	PDI-B	CGHC
PDI-M	At2g32920	PDI9	PDIL2-3	PDIL5-2	PDI-M2	CGHC, CGHC
	At1g04980	PDI10	PDIL2-2	PDIL5-1	PDI-M1	CGHC, CGHC
PDI-S	At2g47470	PDI11	PDIL2-1	PDIL4-1	PDI-S	CGHC, CGHC
PDI-C	At4g27080	PDI7	PDIL5-4	PDIL8-2	PDI-C2	CYWC
	At3g20560	PDI12	PDIL5-3	PDIL8-1	PDI-C1	CYWS
	At1g50950	PDI13^a^	–	–	–	CYWS
PDI-A	At1g07960	PDI14^a^	PDIL5-1	PDIL6-1	PDI-A	CKHC

^a^Modification to the original 12 PDI classification by Lu and Christopher (2008)

To identify PDI-C isoforms among non-Viridiplantae (green plant) species, we performed BLAST searches against the predicted protein databases shown in Table 3, using the deduced amino acid sequence of *Arabidopsis* PDI7 as the search query sequence (preliminary searches using the sequences of PDI12 or PDI13 as the search query gave identical results). Only search results containing a thioredoxin domain and at least one of the two conserved domains of Erv41p/Erv46p-like proteins (i.e., the ERGIC-N and COPII-coated Erv domains) were designated as PDI-C isoforms. All identified non-plant PDI-C sequences and their corresponding accession numbers are provided in Online Resource 2, with alterations to the original source sequences based on available EST data highlighted in yellow.

At least four different naming systems have been proposed for the PDI family in *Arabidopsis* (Table 1). In this report, we utilize the nomenclature established in Lu and Christopher (2008), modified to include two additional genes: *PDI13* and *PDI14* (*Arabidopsis* Genome Initiative identifiers At1g50950 and At1g07960, respectively). For all other organisms, we used the classification scheme proposed by Selles et al. (2011) to avoid creating a duplicate series of names for the plant PDI-C isoforms identified in our database searches, matching our designations to theirs wherever the two data sets overlapped.

### Bioinformatic and phylogenetic analyses

The predicted locations of TMDs were obtained using the hidden Markov model-based membrane protein topology prediction program, TMHMM (v. 2.0) (http://www.cbs.dtu.dk/services/TMHMM/; Krogh et al. 2001). Protein secondary structure predictions for α-helices, β-strands and coiled coils were obtained using the program JPred4 (http://www.compbio.dundee.ac.uk/jpred/; Drozdetskiy et al. 2015). The illustrated domain arrangements for the Erv41p/Erv46p family proteins of *Arabidopsis*, humans and yeast were based on the boundaries reported by the Pfam database (Finn et al. 2014) for the ERGIC-N (accession number PF13850), Thioredoxin (PF00085), and COPII-coated Erv (PF07970) conserved domain families. Depictions of protein domain arrangements were generated using the program Domain Graph (DOG) v. 2.0 (Ren et al. 2009).

For phylogenetic analyses, multiple amino acid sequence alignments were performed with MUSCLE (multiple sequence comparison by log-expectation; Edgar 2004) using the default parameters on the European Bioinformatics Institute server (http://www.ebi.ac.uk/Tools/msa/muscle/). The resulting alignments were then visually inspected for errors using the Alignment Explorer sequence editor function of the program MEGA (Molecular Evolutionary Genetics Analysis) version 6.06 (Tamura et al. 2013). Any obvious alignment errors were corrected manually in MEGA6. The alignments were subsequently trimmed with Gblocks (http://molevol.cmima.csic.es/castresana/Gblocks_server.html) using the default stringency settings to remove positions that are not homologous across all proteins in the dataset, or have become saturated by multiple substitutions (Castresana 2000). All multiple sequence alignments utilized in this study are provided in Online Resource 3, with the Gblocks-selected conserved positions indicated above each alignment by red bars.

Phylogenetic reconstructions were performed in MEGA6 using the Gblocks-trimmed sequence alignments. NJ analyses were conducted with Poisson correction distance and pairwise deletion of gap positions. For ML analyses, the trimmed alignments were run through the Find Best DNA/Protein Models function of MEGA6 to identify the best-fit substitution model for each alignment. The Le and Gascuel (2008) substitution matrix with discrete gamma distribution (LG + G) was identified as the best-fit model in each instance. All ML trees were generated in MEGA6 using the LG + G substitution model with 5 discrete rate categories, with the initial tree generated by NJ, and the tree space explored by Nearest Neighbor-Interchange with the branch swap filter set to very strong. The branch confidence values of NJ and ML trees were calculated from 1000 bootstrap replicates.

### Generation of transgenic GUS reporter lines

Constructs for the expression of the β-glucuronidase (GUS) histological reporter gene (*gusA*) under the control of the promoters of *PDI7*, *PDI12* or *PDI13* were assembled in the binary vector, pCAMBIA1302. The 5′-flanking sequences ~2.8 kb upstream of the start codon of *PDI7*, ~3.3 kb upstream of *PDI12*, and ~2.7 kb upstream of *PDI13* were amplified from *Arabidopsis* (Col-0) genomic DNA by PCR, using forward and reverse primers engineered with the restriction sites KpnI (GGTACC) and XhoI (CTCGAG), respectively. The sequences of the primers used to amplify the promoter regions of the three PDI-C genes are as follows (restriction sites underlined): PDI7_pro_F (5′-ACG TGG TAC CGA ACT ACC GA-3′) and PDI7_pro_R (5′-TGG ACT CGA GTT TCG TCG GAG AGG GAG TC-3′) for *PDI7*; PDI12_pro_F (5′-AAT CGG TAC CCG TCA CCT TCT TCG TTA TTG TC-3′) and PDI12_pro_R (5′-GAA ACT CGA GCT GCC ACC CGA GAA GAA TC-3) for *PDI12*; and PDI13_pro_F (5′-AAT TGG TAC CAT GAT TGA TTG ACA AGT AAA ATG T-3′) and PDI13_pro_R (5′-TGA AAC TCG AGC TGT AAC AAA GAA GAA AGG ATT CT-3′) for *PDI13*. The *gusA* gene was amplified from pCAMBIA1304 using primers gusA.F (5′-GGA CTC GAG ACC ATG GTA GAT CTG ACT AG-3′) and gusA.R (5′-CTC CGG TCA CCT ATT GTT TGC CTC CCT GCT GCG-3′), which incorporated the restriction sites XhoI (CTCGAG) and BstEII (GGTNACC), respectively. The *PDI7*_*pro*_:*GUS*, *PDI12*_*pro*_:*GUS* and *PDI13*_*pro*_:*GUS* constructs were assembled by cloning the corresponding KpnI/XhoI-digested promoter fragment and XhoI/BstEII-digested *gusA* fragment between the KpnI and BstEII sites of pCAMBIA1302 by three-way ligation. The authenticity of each construct was verified by DNA sequencing. The constructs were transformed into *Agrobacterium tumefaciens* strain GV3101, and then introduced into *Arabidopsis* (Col-0) plants by *Agrobacterium*-mediated transformation, using the floral dip method (Clough and Bent 1998).

### GUS expression analysis

Histochemical staining of GUS activity was performed as described (Kim et al. 2006). Prior to GUS staining, etiolated *Arabidopsis* (Col-0) seedlings were grown vertically for 5 days in darkness on agar plates. Light-grown seedlings were grown vertically on agar plates for 14 days at 22 °C under a 16 h-light/8 h-dark cycle. The agar plates contained 0.8 % (w/v) Gellan Gum (Sigma-Aldrich), 0.5× Linsmaier & Skoog media (Caisson Laboratories) and 1.5 % (w/v) sucrose. Shoot inflorescences were obtained from 6-week-old plants grown on soil (Farfard Super Fine Germinating Mix, American Clay Works & Supply Company, USA) under a 16 h-light/8 h-dark cycle at 25 °C. To determine the effect of indole-3-acetic-acid (IAA) treatment on GUS expression in roots, 6-d-old seedlings grown vertically on 0.5× LS agar plates were incubated in 0.5× LS liquid media containing either 0 M (negative control) or 1 µM IAA for 24 h prior to GUS staining. Root samples were mounted on glass slides in 50 % glycerol, and images were acquired on an Olympus BX-51 upright microscope. All other images were taken on an Olympus SZX-12 stereomicroscope, with samples submerged in 70 % ethanol in a petri dish.

## Results

### Phylogenetic relationships among plant Erv41p/Erv46p homologs

A novel feature of the *Arabidopsis* PDI-C proteins is their sequence homology to both classical PDIs and the yeast cargo receptor proteins Erv41p and Erv46p. Whereas several studies have examined the plant PDI family in detail (Houston et al. 2005; Lu and Christopher 2008; d’Aloisio et al. 2010; Selles et al. 2011), thus far a comprehensive characterization of Erv41p/Erv46p homologs in plants has not been reported. We, therefore, searched the TAIR10 protein database for all homologs of Erv41p and Erv46p in the model plant, *A. thaliana*. In addition to PDI7 and PDI12, we identified a third (previously unreported) PDI-C paralog, which we designated as PDI13 (At1g50950), as well as three Erv41p/Erv46p homologs lacking sequence similarity to PDIs (At1g22200, At1g36050 and At3g22290). PDI-related and non-PDI homologs of Erv41p and Erv46p were likewise identified across a wide range of green plant (Viridiplantae) species, including terrestrial plants and green algae (Online Resource 1). All identified plant Erv41p/Erv46p homologs possessed the N-proximal ERGIC-N domain (PF13850) and C-proximal COPII-coated Erv domain (PF07970) shared by yeast Erv41p and Erv46p, and their mammalian homologs ERGIC1, ERGIC2 and ERGIC3 (Orci et al. 2003).

**Fig. 1:**
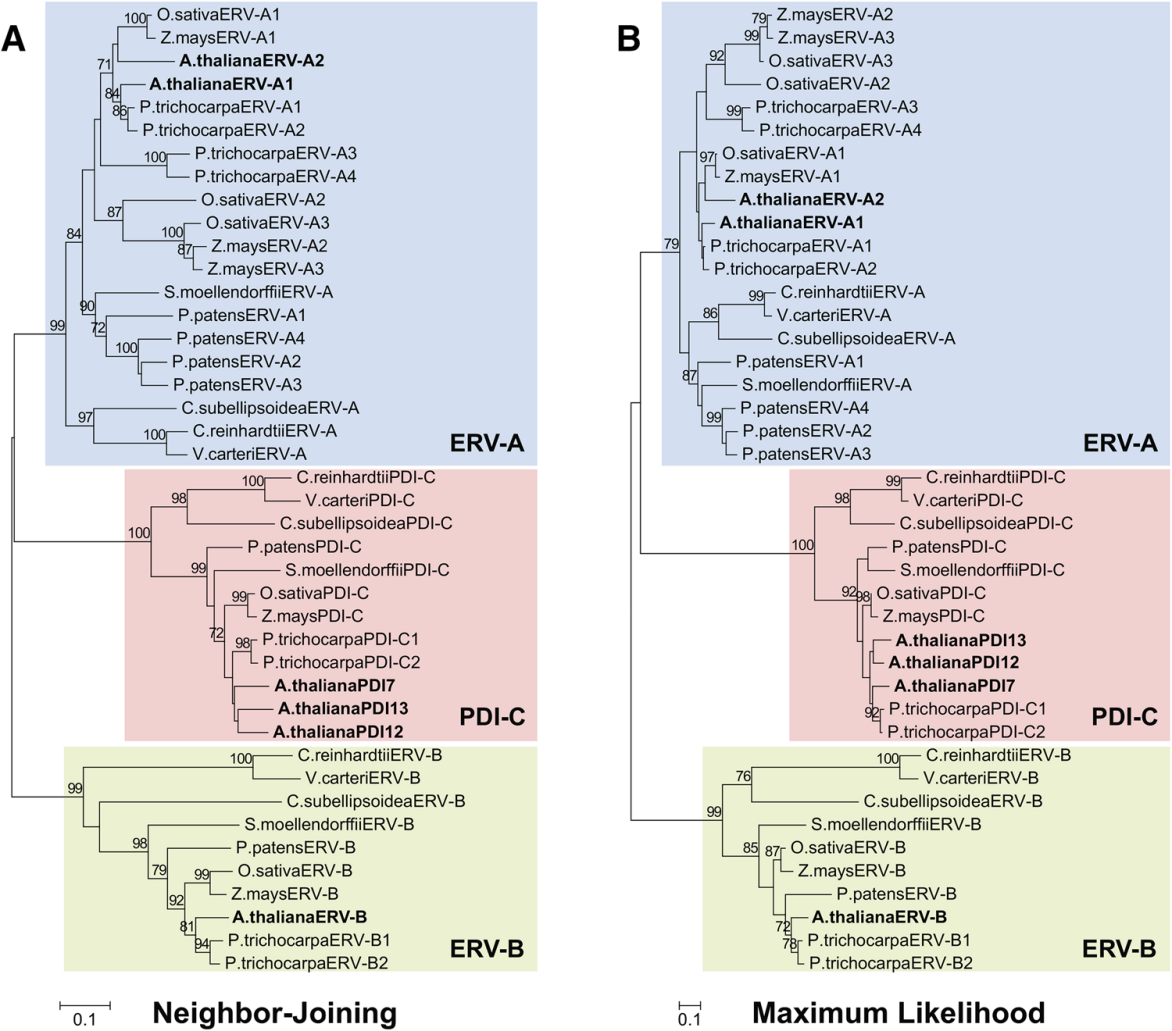
Phylogenetic analysis of plant Erv41p/Erv46p homologs. **a** The unrooted NJ tree was generated with evolutionary distances computed using the Poisson correction method. The tree is drawn to scale, with branch lengths proportional to the number of amino acid substitutions per site. **b** The unrooted ML tree was generated using the LG + G model, with 5 discrete gamma categories (+G, parameter = 0.9366). The tree with the highest log likelihood (−5049.3364) is shown, with branch lengths measured in the number of substitutions per site. NJ and ML analyses were performed using a Gblocks-trimmed multiple sequence alignment consisting of 164 positions. Support values are shown *above the branches*, and are calculated from 1000 bootstrap replicates. Only bootstrap values ≥70 % are shown. The three major clades are *shaded*, with the *Arabidopsis* homolog(s) in each clade highlighted in *bold*

To define evolutionary relationships among the plant Erv41p/Erv46p homologs, phylogenetic analysis was performed using Erv41p/Erv46p homologs from a broad representation of plant species, including the monocots *Oryza sativa* and *Zea mays*,, the dicots *A. thaliana* and *Populus tricocarpa*, the bryophyte *Physcomitrella patens*, the lycophyte *Selaginella moellendorffii*, and the green algae *Chlamydomonas reinhardtii*, *Volvox carteri* and *Coccomyxa subellipsoidea* C-169. Using the alignment-trimming program, Gblocks, we identified five blocks of sequence conservation shared by all the examined homologs. The first and second conserved sequence blocks overlapped with the region defined as the ERGIC-N domain, while the other three blocks were located within the COPII-coated Erv domain (Online Resource 3). Phylogenetic reconstruction using the distance-based NJ method with the concatenated Gblocks alignment revealed that all the examined plant Erv41p/Erv46p-like proteins were resolved into three major groups showing strong (99–100 %) bootstrap support values (Fig. 1a). One group consisted entirely of members of the PDI subfamily, PDI-C (defined as Erv41p/Erv46p homologs possessing a single thioredoxin-like catalytic domain), while the other two groups consisted of Erv41p/Erv46p homologs lacking sequence similarity to PDIs (i.e., lacking thioredoxin-like domains). We designated the two non-PDI groups as Erv41p/Erv46p-like protein subfamily A (ERV-A) and subfamily B (ERV-B). Phylogenetic analysis using the ML method resulted in a similar division into three major clades (Fig. 1b), with strong support for the ERV-B (99 %) and PDI-C (100 %) clades, and moderate support for ERV-A (79 %). Although the NJ and ML methods did not produce trees with identical topology, branches that were at least moderately supported (>70 %) by ML were likewise supported by NJ, and thus the differences between the NJ and ML trees were mainly attributed to weakly supported interior branches.

To gain insight into the overall frequency of genes encoding ERV-A, ERV-B and PDI-C in plants, phylogenetic analysis was performed on all Erv41p/Erv46p homologs identified from the expanded list of plant species (Table 2). A NJ-based phylogenetic tree constructed from a Gblocks-trimmed alignment of these sequences indicated that all surveyed plants encoded at least one isoform from each of the three subfamilies (Table 2; Online Resource 4). Among green algae, a single isoform each of ERV-A, ERV-B and PDI-C was found in the unicellular species *C. reinhardtii* and *C. subellipsoidea*, and in the multicellular species *V. carteri*. In both monocots and dicots, the ERV-A subfamily was generally the most well represented, with 2-4 isoforms found among them. Conversely, ERV-B was generally the least-represented subfamily, with all plants surveyed in this study possessing 1 or 2 ERV-B isoforms. Many plants also have only a single isoform of PDI-C, although poplar has two, the Fabaceae members (*Glycine max*, *Phaseolus vulgaris* and *Medicago truncatula*) possess 2-4, and the Brassicaceae members (*A. thaliana*, *Capsella rubella*, *Euterma salsugineum*, and *Brassica rapa*) possess 3-6.

**Table 2:** Representation of the three Erv41p/Erv46p-like classes in plants

	Species	ERV-A	ERV-B	PDI-C	PDI-C active site motifs	Phytozome 10 dataset
Chlorophytes	*Chlamydomonas reinhardtii*	1	1	1	CHWC	*C. reinhardtii* (v5.5)
	*Volvox carteri*	1	1	1	CHWC	*V. carteri (v2.0)*
	*Coccomyxa subellipsoidea C*-*169*	1	1	1	CPWC	*C. subellipsoidea* C-169 (v2.0)
Charophytes	*Klebsormidium flaccidum*	1	1	1	CPWS	(Not available)
Bryophytes	*Physcomitrella patens*	4	1	1	CPWS	*P. patens* (v3.0)
Lycophytes	*Selaginella moellendorffii*	1	1	1	CIWS	*S. moellendorffii* (v1.0)
Monocots	*Brachypodium distachyon*	3	1	1	CYWS	*B. distachyon* (v2.1)
	*Oryza sativa*	3	1	1	CYWS	*O. sativa* (v7.0)
	*Zea mays*	3	1	1	CYWS	*Z. mays* (6a)
	*Sorghum bicolor*	3	1	1	CYWS	*S. bicolor* (v2.1)
Dicots	*Solanum lycopersicum*	2	2	1	CYWS	*S. lycopersicum (iTAG2.3)*
	*Solanum tuberosum*	2	2	1	CYWS	*S. tuberosum (v3.4)*
	*Vitis vinifera*	2	1	1	CYWS	*V. vinifera (Genoscope.12X)*
	*Theobroma cacao*	2	1	1	CYWS	*T. cacao (v1.1)*
	*Eutrema salsugineum*	3	1	3	CYWS (×2); CYWC	*E. salsugineum (v1.0)*
	*Capsella rubella*	2	1	3	CYWS (×2); CYWC	*C. rubella* (v1.0)
	*Brassica rapa Chiifu*-*401*	4	2	6	CYWS (×4); CYWC (×2)	(Not available)
	***Arabidopsis*** ***thaliana***	2	1	3	CYWS (×2); CYWC	*A. thaliana* TAIR10
	*Prunus persica*	2	1	1	CYWS	*P. persica (v1.0)*
	*Cucumis sativus*	3	1	1	CYWS	*C. sativis* (v1.0)
	*Glycine max*	4	2	4	CYWS (×2); CSWC (×2)	*G. max* (v1)
	*Phaseolus vulgaris*	2	1	2	CYWS, CSWC	*P. vulgaris* (v1.0)
	*Medicago truncatula*	2	1	2	CYWS, CSWC	*M. truncatula* (Mt4.0v1)
	*Populus tricocarpa*	4	2	2	CYWS (×2)	*P. trichocarpa* (v3.0)

The plant species that was the source of the Erv41p/Erv46p-like proteins in this study is denoted in bold

### Comparison of the domain architecture and conserved sequence features of ERV-A, ERV-B and PDI-C isoforms

**Fig. 2:**
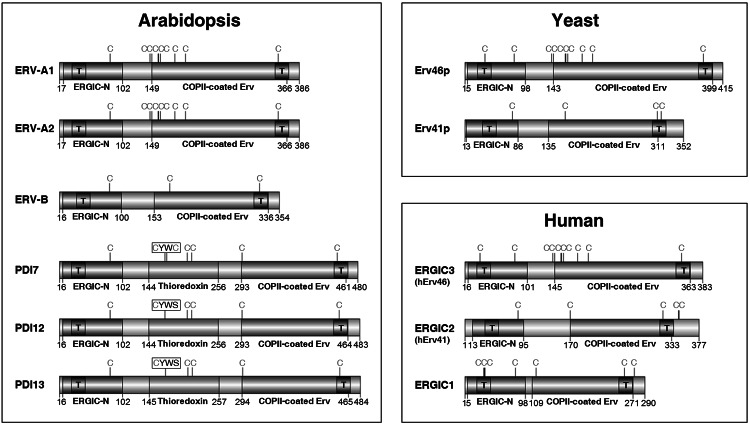
Domain arrangements of Erv41p/Erv46p proteins from *Arabidopsis*, yeast, and humans. ERGIC-N domains are shown in *blue*, COPII-coated ERV domains in *green*, and thioredoxin-like domains in *red*. *Black boxes* labeled with the *letter* *T* indicate the predicted positions of TMDs. The positions of Cys residues are indicated by the *letter C* (colour figure online)

Based on our phylogenetic analysis, the *Arabidopsis* genome encodes two ERV-A, one ERV-B, and three PDI-C members (Fig. 1). Sequence analyses revealed that each of the six *Arabidopsis* Erv41p/Erv46p homologs is predicted to contain two TMDs separated by a large central loop region, with short tail segments at either end of the protein (Fig. 2). The predicted TMDs overlap with the aforementioned ERGIC-N and COPII-coated Erv domains. The boundaries of COPII-coated Erv domains of yeast Erv46p and plant ERV-A proteins, as defined by the Pfam database, are extended by ~30 amino acids at their N-terminal end relative to the COPII-coated Erv domains of ERV-B and PDI-C proteins (Online Resource 5). In yeast Erv41p, the lumenal portion of the ERGIC-N and COPII-coated Erv domains forms large β-sheets that assemble together into a twisted β-sandwich configuration (Biterova et al. 2013). According to the secondary structural analysis via Jpred, the N- and C-proximal conserved domains of *Arabidopsis* ERV-A, ERV-B, and PDI-C isoforms are β-strand-rich similar to the ERGIC-N and COPII-coated Erv domains of yeast Erv41p and Erv46p (Online Resource 5).

Unlike the members of subfamilies ERV-A and ERV-B, PDI-C isoforms contain a thioredoxin-like domain homologous to the catalytic **a** and **a′** domains of classical PDIs. The PDI-C catalytic domain is located between the ERGIC-N and COPII-coated Erv domains (Fig. 2). Whereas the catalytic domains of classical PDIs have the vicinal dithiol active site motif CGHC, the catalytic domains of most monocot and dicot PDI-C isoforms, including *Arabidopsis* PDI12 and PDI13, possess the novel mono-cysteine motif CYWS (Table 2). However, a few dicot PDI-C isoforms with di-cysteine motifs were identified, including *Arabidopsis* PDI7, which harbors the active site vicinal dithiol motif CYWC. In general, all terrestrial plants surveyed possessed at least one PDI-C isoform with a CxxS motif, and all green algae analyzed possessed at least one isoform with a CxxC motif (Table 2).

Comparison of the deduced amino acid sequences of Erv41p/Erv46p-like proteins from plants, yeast and humans revealed that members of the ERV-A subfamily possess 9 conserved cysteine (Cys) residues that are also conserved in yeast Erv46p, although the position of the final (C-terminal-most) Cys residue in yeast Erv46p is shifted by -1 residue relative to plant ERV-A proteins (Online Resource 5). The members of subfamilies ERV-B and PDI-C possess Cys residues at positions analogous to the 1st, 8th and 9th Cys residues of ERV-A proteins, but not the 2nd through 7th Cys residues, which are located within the extended N-terminal portion of the COPII-coated Erv domain of ERV-A isoforms, a region which is absent in both ERV-B and PDI-C (Fig. 2). The 2nd and 3rd Cys residues and the 4th–6th Cys residues correspond to two highly conserved motifs in plant ERV-A proteins with the sequence CG(S/T)C and CCN(N/S/T)C, respectively. Similar motifs are present in yeast Erv46p (CGPC; CCQDC) and human ERGIC3 (CESC; CCNTC). The CxxC and CCxxC motifs of plant ERV-As are typically separated by nine intervening amino acids. The functional importance of specific Cys residues in yeast Erv46p and Erv41p has not been determined, but given their conservation between animals, plants, and yeast, we infer that they must be crucial for protein function and/or stability.

The C-terminus of Erv46p ends in the COPI-binding ER retrieval signal, KKxx (Otte et al. 2001). In plants, the last four amino acids at the C-terminus of ERV-A and PDI-C isoforms share the consensus sequence GKxx, which resembles the common membrane protein ER retrieval motif, xKxx (Jackson et al. 1990). ERGIC3 also possesses a C-terminal xKxx motif (specifically, GKTT). On the other hand, no obvious consensus motif was found among the C-termini of ERV-B isoforms. Based on their conservation of CxxC and CCxxC motifs and the presence of a potential ER retention sequence at their C-termini, we hypothesize that ERV-A isoforms most likely fulfill the role of Erv46p in plants. Likewise, since both plant ERV-B isoforms and yeast Erv41p lack these Cys motifs and do not possess an obvious C-terminal ER retrieval signal, ERV-B may function as the plant equivalent of Erv41p. In contrast, PDI-C isoforms have no obvious counterpart in either humans or yeast.

### Distribution of PDI-C isoforms among eukaryotic organisms

To determine how widespread isoforms of PDI-C are among eukaryotes, we searched for orthologs of PDI7/PDI12/PDI3 encoded by representative species from the major eukaryotic groups. Within the group Archaeplastida, putative PDI-C isoforms were identified in all species of green plants examined, and in two of four species of red algae (*Chondrus crispus* and *Porphyridium purpureum*, but not *Cyanidioschyzon merolae* or *Galdieria sulphuraria*); however, no PDI-C isoform was found in the sequenced genome of the model glaucophyte, *Cyanophora paradoxa* (Table 3). We also identified putative PDI-C isoforms in several stramenopiles, the haptophyte *Emiliania huxleyi*, the cryptomonad *Guillardia theta*, and the rhizarians *Bigelowiella natans* and *Reticulomyxa filosa* (Table 3). In contrast to the terrestrial plants, the CxxC motif is evolutionarily conserved among the PDI-Cs of non-plant species (Table 3). There were no PDI-C isoforms present among alveolates, excavates, amoebozoans, animals or yeast.

**Table 3:** PDI-C isoforms from non-plant species

	Species	PDI-C	PDI-C active site motifs	Searched database
Rhodophyta	*Chondrus crispus*	2	CIHC, CGFC	NCBI nr (taxID: 2769)
	*Porphyridium purpureum*	2	CIWC, CPFC	*P. purpureum* Genome Project^a^
	*Cyanidioschyzon merolae*	0		*C. merolae* Genome Project^b^
	*Galdieria sulphuraria*	0		*G. sulphuraria* Genome Project^c^
Glaucophyta	*Cyanophora paradoxa*	0		*Cyanophora* Genome Project^d^
Haptophyta	*Emiliania huxleyi*	3	CIWC, CPWS, CFWS	NCBI nr (taxID: 2903)
Cryptophyta	*Guillardia theta*	2	CHWC, CHWC	NCBI nr (taxID: 55529)
Stamenopiles	*Albugo candida*	3	CIWC, CPFS, CPFS	NCBI nr (taxID: 65357)
	*Albugo laibachii*	2	CIWC, CPFS	NCBI nr (taxID: 653948)
	*Aphanomyces invadans*	1	CIWC	NCBI nr (taxID: 157072)
	*Aureococcus anophagefferens*	3	CLYC, CIWC, CIWC	NCBI nr (taxID: 44056)
	*Ectocarpus siliculosus*	2	CVWC, CGWC	NCBI nr (taxID: 2880)
	*Nannochloropsis gaditana*	2	CIWC, CVWC	NCBI nr (taxID: 72520)
	*Phaeodactylum tricornutum*	2	CVWC, CSHC	NCBI nr (taxID: 2850)
	*Phytophthora infestans*	2	CVWC, CPFC	NCBI nr (taxID: 4787)
	*Phytophthora parasitica*	2	CVWC, CPFC	NCBI nr (taxID: 4792)
	*Saprolegnia diclina*	2	CIWC, CPFS	NCBI nr (taxID: 112098)
	*Saprolegnia parasitica*	2	CIWC, CPFS	NCBI nr (taxID: 101203)
	*Thalassiosira pseudonana*	3	CIWC (x2), CSHC	NCBI nr (taxID: 35128)
Rhizaria	*Bigelowiella natans*	2	CHWC, CPWC	*B. natans* JGI Portal^e^
	*Reticulomyxa filosa*	2	CSHC, CIHC	NCBI nr (taxID: 46433)
Alveolates	*Cryptosporidium hominis*	0		NCBI nr (taxID: 237895)
	*Cryptosporidium parvum*	0		NCBI nr (taxID: 5807)
	*Paramecium tetraurelia*	0		NCBI nr (taxID: 5888)
	*Tetrahymena thermophila*	0		NCBI nr (taxID: 5911)
Excavates	*Leishmania major*	0		NCBI nr (taxID: 5664)
	*Naegleria gruberi*	0		NCBI nr (taxID: 5762)
	*Trichomonas vaginalis*	0		NCBI nr (taxID: 5722)
	*Trypanosoma brucei*	0		NCBI nr (taxID: 5691)
	*Trypanosoma cruzi*	0		NCBI nr (taxID: 5693)
Amoebozoans	*Dictyostelium discoideum*	0		NCBI nr (taxID: 44689)
	*Entamoeba histolytica*	0		NCBI nr (taxID: 5759)
	*Polysphondylium pallidum*	0		NCBI nr (taxID: 13642)
Choanoflagellates	(Taxonomic group search)	(None)		NCBI nr (taxID: 28009)
Animals	(Taxonomic group search)	(None)		NCBI nr (taxID: 33208)
Yeast	(Taxonomic group search)	(None)		NCBI nr (taxID: 4932)

^a^ *Porphyridium purpureum* Genome Project (http://cyanophora.rutgers.edu/porphyridium)

^b^ *Cyanidioschyzon merolae* Genome Project (http://merolae.biol.s.u-tokyo.ac.jp)

^c^ *Galdieria sulphuraria* Genome Project (http://genomics.msu.edu/galdieria/index.html)

^d^Cyanophora Genome Project (http://cyanophora.rutgers.edu/cyanophora/home.php)

^e^ *Bigelowiella* *natans* Joint Genome Institute (JGI) Portal (http://genome.jgi.doe.gov/Bigna1/Bigna1.home.html)

### Identification of PDI-C duplication events among terrestrial plant lineages

**Fig. 3:**
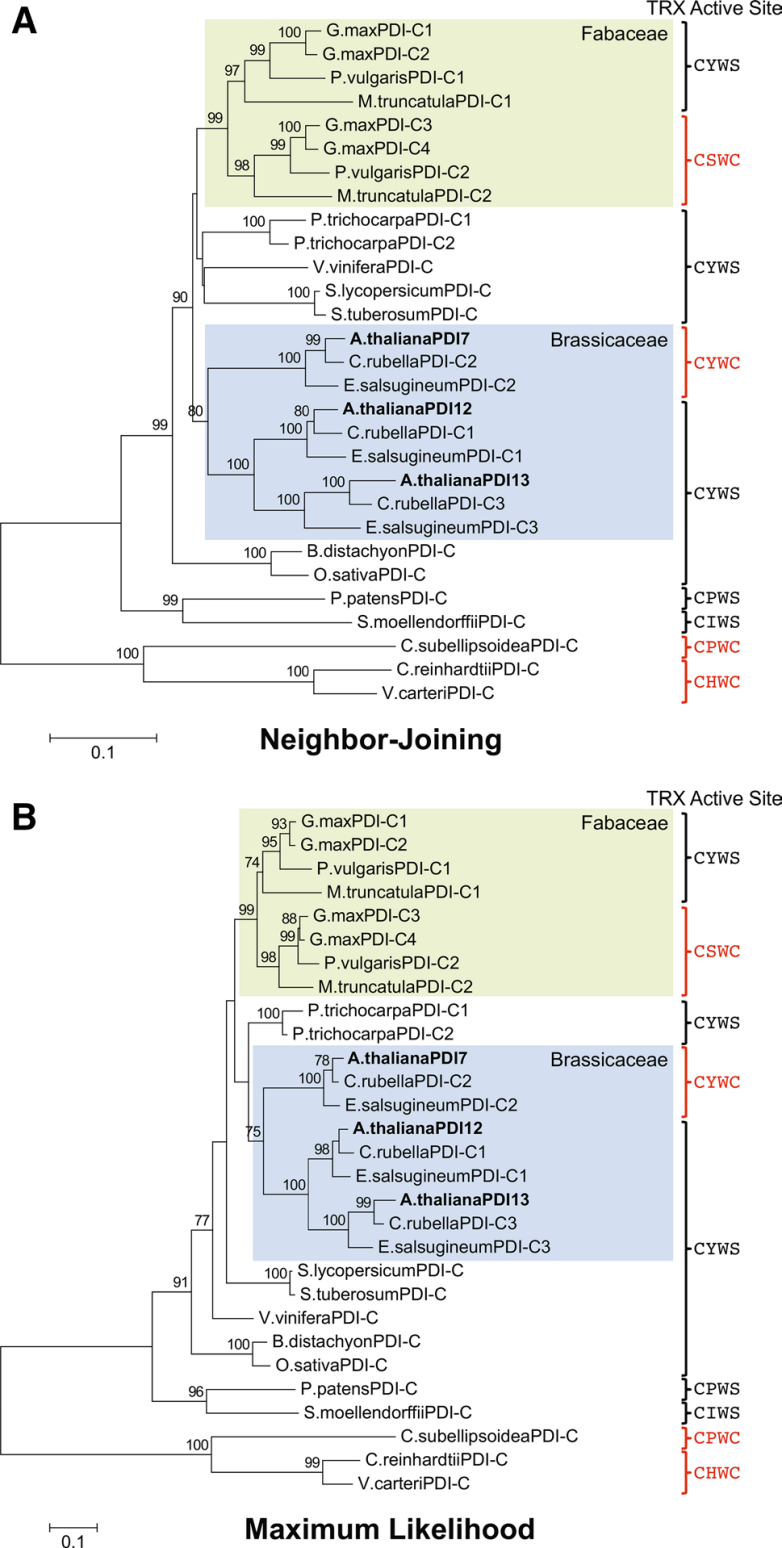
Phylogenetic analysis of plant PDI-C isoforms. **a** The unrooted NJ tree was generated with evolutionary distances computed using the Poisson correction method. The tree is drawn to scale, with branch lengths proportional to the number of amino acid substitutions per site. **b** The unrooted ML tree was generated using the LG + G model, with 5 discrete gamma categories (+G, parameter = 0.9871). The tree with the highest log likelihood (−7366.6878) is shown, with branch lengths measured in the number of substitutions per site. NJ and ML analyses were performed using a Gblocks-trimmed multiple sequence alignment consisting of 407 positions. Support values are shown *above the branches*, and are calculated from 1000 bootstrap replicates. Only bootstrap values ≥70 % are shown. The Fabaceae and Brassicaceae clades are *shaded*. The thioredoxin (TRX) domain active site sequence motifs of the various PDI-C isoforms are shown on the *right*, with motifs containing two Cys residues highlighted in *red* (colour figure online)

To gain insight into the evolution of PDI-C isoforms within land plants, NJ- and ML-based phylogenetic trees were generated from a Gblocks-processed multiple sequence alignment that included only members of the PDI-C subfamily (Online Resource 3). Both trees indicated that within the Brassicaceae family, two PDI-C gene duplication events occurred prior to the divergence of *Arabidopsis* from its salt-tolerant relative *Eutrema salsugineum*. The first duplication event gave rise to the *PDI7* and *PDI12/PDI13* lineages, while the second event divided the latter into separate *PDI12* and *PDI13* lineages (Fig. 3a, b). In the Fabaceae family, a similar gene duplication event occurred prior to the last common ancestor of *G. max*, *M. truncatula*, and *P. vulgaris*, resulting in one PDI-C lineage with the conserved CYWS motif and another with the nonstandard motif CSWC (Fig. 3a, b).

### GUS expression analysis of the PDI-C genes in *Arabidopsis*

The unusual structure of PDI-C isoforms elicits the question about where these novel proteins function during the growth and development of plants. To compare the spatial expression patterns of the promoters of *PDI7*, *PDI12*, and *PDI13* in various organs of *Arabidopsis*, the genomic DNA sequence 2.7- to 3.3-kb upstream of the start codon of each gene was transcriptionally fused to the GUS reporter coding sequence. Promoter:GUS constructs were introduced into wild-type *Arabidopsis* (ecotype Col-0) plants by *Agrobacterium*-mediated transformation, and at least 10 independent transgenic lines were analyzed to establish the consensus expression pattern for each promoter:GUS fusion.

**Fig. 4:**
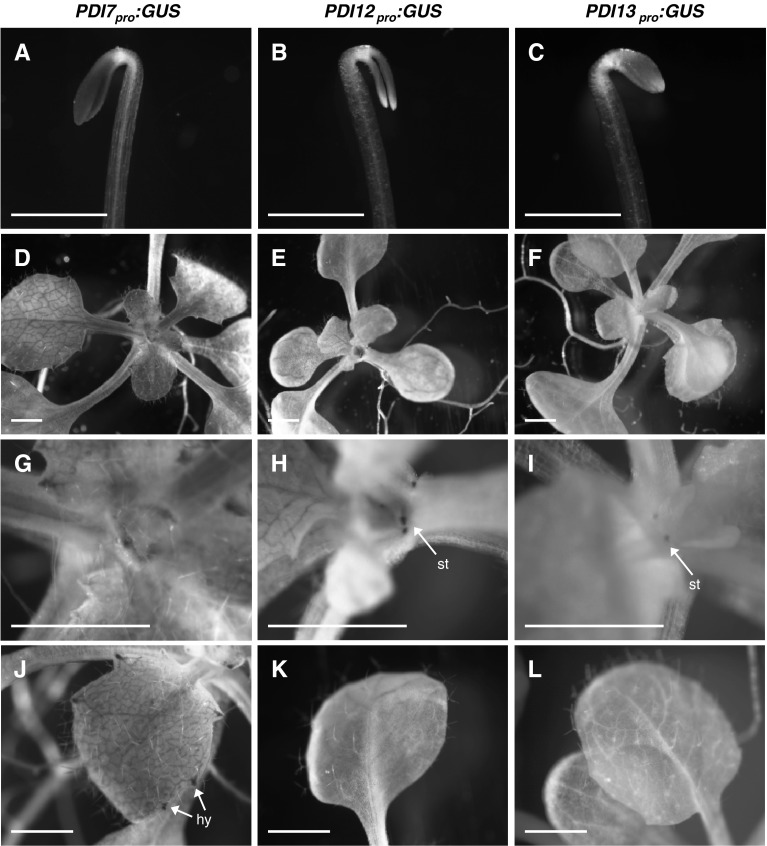
Expression pattern of *Arabidopsis* PDI-C gene promoters in seedlings. The GUS staining pattern of *PDI7*_*pro*_:*GUS* (**a**, **d**, **g**, **j**) *PDI12*_*pro*_:*GUS* (**b**, **e**, **h**, **k**), and *PDI13*_*pro*_:*GUS* (**c**, **f**, **i**, **l**) seedlings were examined after 5 days of growth in darkness (**a**–**c**), or after 2 weeks of grown under a normal 16 h light, 8 h darkness cycle (**d**–**l**). *Arrows* indicate expression in hydathodes (*hy*) and stipules (*st*). The images are representative examples from at least three independent experiments of ≥10 independent transgenic lines per construct. *Scale bar* 1 mm

When grown under constant darkness, strong GUS expression was detected in the cotyledons of etiolated *PDI7*_*pro*_:*GUS* (Fig. 4a), *PDI12*_*pro*_:*GUS* (Fig. 4b), and *PDI13*_*pro*_:*GUS* (Fig. 4c) seedlings, with *PDI12*_*pro*_:*GUS* seedlings also displaying GUS activity in the vasculature of the apical hook (Fig. 4b). Under light-grown conditions, *PDI7*_*pro*_:*GUS* seedlings displayed GUS staining in the leaf vasculature, with the strongest staining detected in younger, expanding leaves, and decreased staining observed in older leaves (Fig. 4d). The *PDI7* promoter was also active in hydathodes (Fig. 4j). *PDI12*_*pro*_:*GUS* seedlings also exhibited GUS activity in the leaf vasculature but, unlike *PDI7*_*pro*_:*GUS*, did not display GUS expression in hydathodes (Fig. 4e, k). Furthermore, prominent stipule staining was observed in *PDI12*_*pro*_:*GUS* seedlings (Fig. 4h), but not in *PDI7*_*pro*_:*GUS* seedlings (Fig. 4g). Transgenic plants harboring the *PDI13*_*pro*_:*GUS* fusion exhibited the weakest overall GUS activity in light-grown seedlings (Fig. 4f). Staining in the shoot tissues of *PDI13*_*pro*_:*GUS* seedlings was mainly restricted to stipules (Fig. 4i), with no GUS activity detected in leaves (Fig. 4l).

**Fig. 5:**
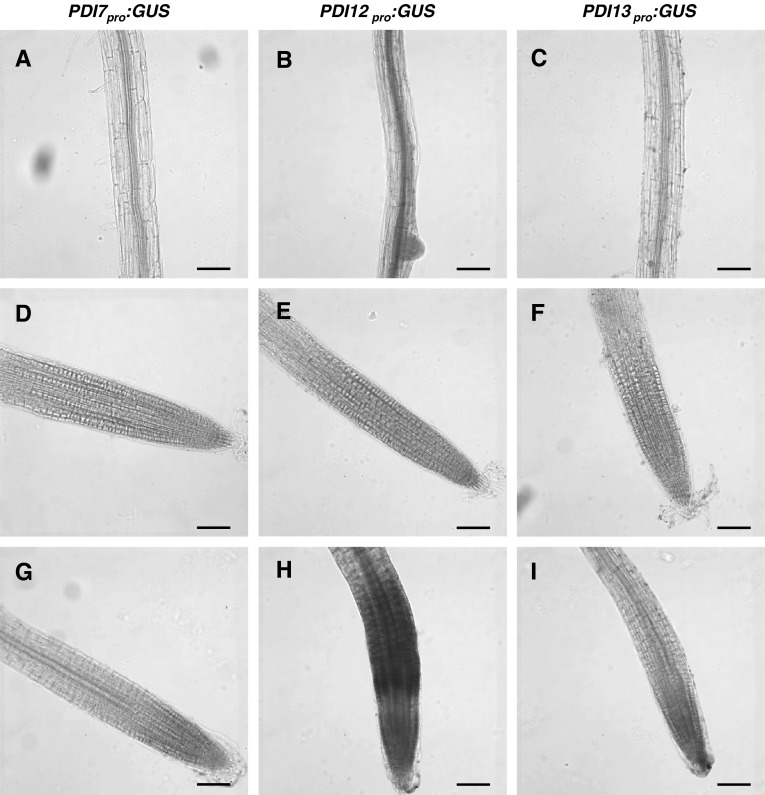
Expression pattern of the three (*PDI7, PDI12, PDI13*) *Arabidopsis* PDI-C gene promoter-GUS fusions in roots. The GUS staining pattern of *PDI7*_*pro*_:*GUS* (**a**, **d**, **g**) *PDI12*_*pro*_:*GUS* (**b**, **e**, **h**), and *PDI13*_*pro*_:*GUS* (**c**, **f** ,**i**) seedlings in the mature zone of roots (**a**–**c**), or at the root apex after incubation in 0 M (**d**–**l**) or 1 µM IAA (**g**–**i**). The images were derived from at least three independent experiments. *Scale bar* 100 µm

Whereas *PDI7*_*pro*_:*GUS* showed the highest overall expression in leaves among the three reporter constructs, *PDI12*_*pro*_:*GUS* displayed the greatest activity in roots. Expression of *PDI12*_*pro*_:*GUS* was detected throughout the root vasculature of 7-day-old seedlings (Fig. 5b), whereas no GUS activity was detected in the root vasculature in either *PDI7*_*pro*_:*GUS* (Fig. 5a) or *PDI13*_*pro*_:*GUS* (Fig. 5c) seedlings. We also observed weak GUS staining in the root caps of *PDI12*_*pro*_:*GUS* seedlings (6 out of 20 roots; Fig. 5e) and *PDI13*_*pro*_:*GUS* seedlings (5 out of 20 roots; Fig. 5f), but not in the root caps of *PDI7*_*pro*_:*GUS* seedlings (0 out of 20 roots; Fig. 5d). Due to the important role of auxin in the regulation of root growth and development (Overvoorde et al. 2010), we examined the effects of exogenously supplied IAA on the promoter activities of *PDI7*, *PDI12*, and *PDI13* in roots. Interestingly, IAA treatment consistently enhanced the intensity of GUS expression in the root caps of *PDI12*_*pro*_:*GUS* (12 out of 12; Fig. 5h) and *PDI13*_*pro*_:*GUS* (12 out of 12; Fig. 5i) 7-day-old seedlings, but did not induce root cap expression of GUS in *PDI7*_*pro*_:*GUS* seedlings (0 out of 12; Fig. 5g). IAA also strongly induced GUS expression in the root elongation zone of *PDI12*_*pro*_:*GUS* (Fig. 5h), but not *PDI7*_*pro*_:*GUS* (Fig. 5g) or *PDI13*_*pro*_:*GUS* (Fig. 5i) seedlings.

**Fig. 6:**
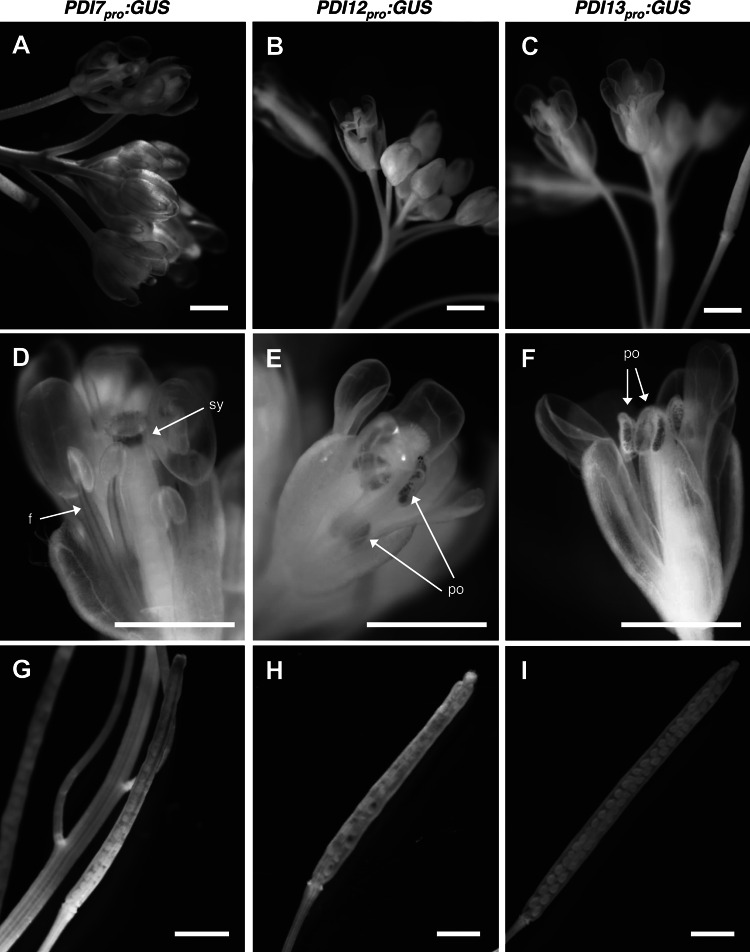
Expression pattern of the three *Arabidopsis* PDI-C gene promoters during flowering and seed formation. The GUS staining pattern of *PDI7*_*pro*_:*GUS* (**a**, **d**, **g**) *PDI12*_*pro*_:*GUS* (**b**, **e**, **h**), and *PDI13*_*pro*_:*GUS* (**c**, **f** ,**i**) at the floral shoot apex (**a**–**c**), in mature flowers (**d**–**f**), and *green* siliques showing developing seeds (**g**–**i**). *Arrows* indicate expression in filaments (*f*), in pollen of anthers (*po*) and the style (*sy*). The images were derived from at least three independent experiments of with a minimum of 10 independent transgenic lines per construct. *Scale bar* 1 mm

In flowering *PDI7*_*pro*_:*GUS* plants, GUS activity was detected in the style, pedicel, and the vasculature of sepals and filaments (Fig. 6a, d), although GUS expression in sepals typically disappeared by stage 14 of flower development (Fig. 6d). *PDI7*_*pro*_:*GUS* plants also displayed staining in inflorescence stems near the shoot apex (Fig. 6a), and in expanding siliques (Fig. 6g). By comparison, GUS staining was restricted exclusively to mature pollen grains in the flowers of *PDI12*_*pro*_:*GUS* (Fig. 6b, e) and *PDI13*_*pro*_:*GUS* (Fig. 6c, f). *PDI12*_*pro*_:*GUS* was also expressed within expanding siliques in the transmitting tract and developing seeds (Fig. 6h); however, no GUS activity was detected in *PDI13*_*pro*_:*GUS* siliques (Fig. 6i).

## Discussion

The PDI and Erv41p/Erv46p-like proteins independently represent two ancient eukaryotic protein families shared among animals, plants and yeast. Whereas domain rearrangements have led to the formation of numerous structurally diverse PDI classes in plants and humans, our comparative analysis of yeast, human and plant homologs of Erv41p and Erv46p indicates that domain rearrangements have not contributed as greatly to the molecular evolution of this family (Fig. 2). One notable exception revealed here is the plant PDI-C subfamily, which has a unique hybrid domain arrangement wherein the thioredoxin-like catalytic domain of PDIs is located between the β-strand-rich ERGIC-N and COPII-coated Erv domains of Erv41p/Erv46p-type proteins (Fig. 2; Online Resource 5). Due to the internal positioning of the thioredoxin domain in PDI-C isoforms, we infer that this subfamily arose through an exon-shuffling event, in which a portion of an ancestral PDI gene was inserted within an ancestral Erv41p/Erv46p-like gene. The combination in PDI-C proteins of the catalytic thioredoxin domain of the PDI family with the conserved domains of Erv41p/Erv46p cargo receptor protein family predicts a need for the pairing redox-related functions with cargo receptor-type processes in the secretory pathway of organisms that encode PDI-C isoforms.

**Fig. 7:**
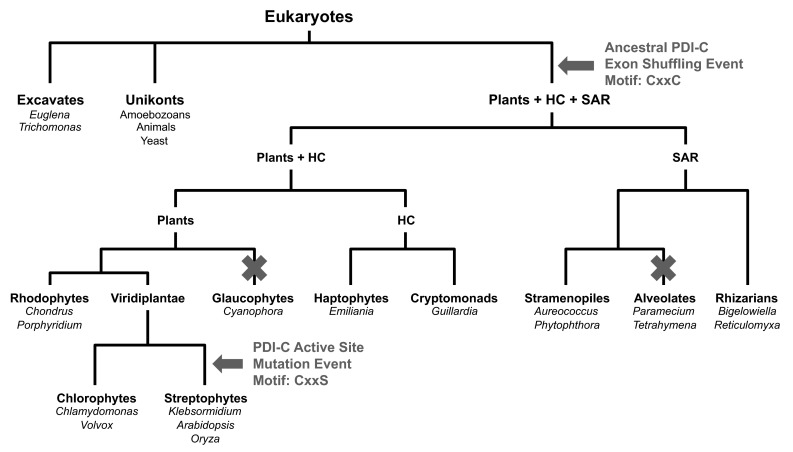
Distribution of PDI-C subfamily among eukaryotes. *X* indicates the loss of the PDI-C subfamily within a lineage. The ancestral PDI-C exon-shuffling event motif (CxxC) and the PDI-C active site mutation event motif (CxxS), are indicated with *red arrows* (colour figure online)

PDI-C isoforms are found among green plants, rhodophytes, haptophytes, cryptomonads, stramenopiles, and rhizarians, but are absent in animals, yeasts, amoebozoans, excavates and alveolates (Tables 2, 3). Based on current models of eukaryotic phylogeny that define the chromalveolates as a polyphyletic grouping (Burki et al. 2008; Hampl et al. 2009), we propose that the PDI-C subfamily emerged from an exon-shuffling event that took place prior to the divergence of the clade plants + HC (haptophytes + cryptomonads) from the clade SAR (stramenopiles + alveolates + rhizarians), with the subsequent loss of the PDI-C subfamily within the alveolate lineage (Fig. 7). The subfamily may also be lost within the glaucophyte lineage, although the nuclear genome of only one glaucophyte species (*C. paradoxa*) has been sequenced thus far (Price et al. 2012). The PDI-C subfamily has also been lost in a subset of red algae (*C. merolae*, *G. sulphuraria*).

With the notable exception of land plants, most eukaryotic organisms that encode members of the PDI-C subfamily possess at least one isoform with a CxxC active site motif, suggesting that the ancestral PDI-C protein likely harbored a di-cysteine active site motif (Fig. 7). After the evolutionary divergence between Chlorophyta and Streptophyta, a presumed mutation event within the PDI-C gene of an ancestral streptophyte converted the active site motif of PDI-C to CxxS, which has since been maintained in land plants (Fig. 7). However, di-cysteine PDI-C isoforms would later reemerge within the Brassicaceae family (CYWC motif) and the Fabaceae family (CSWC motif) following separate gene duplication events. Such an alteration to the number of Cys residues within the active site motif has important implications as to the possible types of reactions these enzymes can catalyze. A catalytic domain with a CxxC motif can potentially mediate the formation, breakage, or rearrangement of disulfide bonds in a client protein, while one with a CxxS motif can only mediate the isomerization of disulfide bonds (Chivers et al. 1998), or the formation of stable enzyme-substrate mixed disulfides (Anelli et al. 2002). Since CxxS-type PDI-C isoforms are conserved among land plants, we hypothesize that PDI12 and/or PDI13 retain the ancestral function(s) of the PDI-C subfamily in *Arabidopsis*, while PDI7 has evolved to acquire new functions requiring a di-cysteine active site sequence, such as catalyzing disulfide bond oxidation or reduction, which would not (at least in theory) be biochemically possible with a CxxS motif.

If PDI12 and/or PDI13 fulfill the ancestral functions of PDI-C isoforms in *Arabidopsis*, then the expression patterns of *PDI12pro*:*GUS* and *PDI13pro*:*GUS* may reflect evolutionarily conserved roles in late stage pollen development or pollen anthesis, and the development or function of stipules, the root cap, and the cotyledons of etiolated seedlings (Figs. 4, 5). *PDI12* promoter expression was also detected in the root vasculature, and in the elongation zone of roots supplied with exogenous auxin, which may represent either additional ancestral functions lost by *PDI13*, or new functions acquired by *PDI12* after the gene duplication event that gave rise to *PDI12* and *PDI13* (Fig. 5). The prominent sites of *PDI7pro*:*GUS* expression are largely non-overlapping with that of *PDI12pro*:*GUS* and *PDI13pro*:*GUS*, suggesting that *PDI7* has evolved at the gene expression level to take on new redox- and/or cargo receptor-related functions in hydathodes and the vasculature of stamen filaments and expanding leaves and sepals.

Most plant PDIs contain ER retention signals and are, therefore, likely to function primarily in the ER lumen. However, the N- and C-terminal similarity of PDI-C isoforms to Erv41p and Erv46p raises the intriguing possibility that these novel hybrid proteins possess the subcellular trafficking properties of Erv41p/Erv46p cargo receptor proteins coupled with the disulfide bond-catalyzing properties of PDIs. Thus, defining the subcellular localization pattern of PDI-Cs will be crucial for understanding the molecular function(s) of these highly atypical members of the PDI family. Since ER-to-Golgi and Golgi-to-ER trafficking is mediated by the COPII and COPI coat protein complexes, respectively (Brandizzi and Barlowe 2013), it would be interesting to determine if PDI-C isoforms possess sequences that interact with subunits of COPI and COPII. Indeed, the presence of the motif GKxx at the C-terminus of plant ERV-A and PDI-C isoforms, as well as human ERGIC1 and ERGIC3, suggests that this motif may serve as a COPI-binding signal in a manner analogous to the dilysine KKxx motif found in Erv46p (Otte and Barlowe 2002). Interestingly, yeast Erv46p, human ERGIC3 and plant ERV-A proteins all possess the motifs CxxC and CCxxC, which is reminiscent of the CxxC vicinal dithiol motif of thioredoxins and thioredoxin domain-containing proteins such as PDIs. Although the CxxC and CCxxC motifs of Erv46p and ERV-A proteins are not nestled within the context of a thioredoxin-fold, it is conceivable that one or both of these motifs may serve some form of redox-related function that is coupled to Erv46p’s role as part of a retrograde cargo receptor complex. If this is indeed the case, then the molecular function of PDI-C isoforms may be similar to Erv46p, with presence of a central thioredoxin domain allowing PDI-C proteins to recognize different cargo substrates than Erv46p, or to conduct redox reactions that are distinct from those catalyzed by the CxxC/CCxxC motifs of Erv46p.

The identification of conserved domains and motifs in PDI-C isoforms and the characterization of the tissue expression patterns of PDI-C genes in *Arabidopsis* presented here have revealed new insights into the potential roles of these proteins at both the molecular and physiological level, and provide a rationale for the in-depth biochemical and structural–functional analysis of these proteins. Such experiments will include examining subcellular localizations, the impacts of various knockout and site-specific gene mutants, redox activity analyses, and identification of interacting substrates, and will provide valuable information on the role of the PDI-C members in the biology of the plant secretory pathway.

## Electronic supplementary material

Online Resource 1. Sequences of identified plant Erv41p/Erv46p homologs identified by database searches. Corrections made to the original sequences due to available EST data or an alternate gene prediction model are highlighted in yellow (PDF 146 kb)

Online Resource 2. Sequences of non-plant PDI-C isoforms identified by database searches. Corrections made to the original sequences due to available EST data or an alternate gene prediction model are highlighted in yellow (PDF 96 kb)

Online Resource 3. Multiple sequence alignments used for phylogenetic analyses. Alignments were generated by the MUSCLE multiple sequence alignment program, and visually inspected and edited (if necessary) using the Alignment Explorer sequence editor of MEGA6. The positions of blocks of conserved sequence identified by Gblocks are indicated by red bars above the alignment (PDF 883 kb)

Online Resource 4. Phylogenetic analysis of all plant Erv41p/Erv46p homologs surveyed in this study. The unrooted NJ tree was generated with evolutionary distances computed using the Poisson correction method. The tree is drawn to scale, with branch lengths proportional to the number of amino acid substitutions per site. NJ analysis was performed using a Gblocks-trimmed multiple sequence alignment consisting of 173 positions. Support values are shown above the branches, and are calculated from 1000 bootstrap replicates. Only bootstrap values ≥70 % are shown. The ERV-A clade is shaded in blue, the ERV-B clade in green, and the PDI-C clade in red (PDF 74 kb)

Online Resource 5. Secondary structure predictions of yeast Erv41p and Erv46p and their *Arabidopsis* homologs. The multiple sequence alignment was generated by MUSCLE. Secondary structure predictions were performed with Jpred4. The Pfam-defined boundaries of the ERGIC-N domain, thioredoxin domain, and COPII-coated Erv domain are boxed in blue, red and green, respectively. The predicted all-beta regions of the ERGIC-N and COPII-coated Erv domains are boxed in orange. The positions of the 9 conserved Cys residues of ERV-A proteins are indicated above the alignment. H: helix, E: β-strand (PDF 146 kb)
