# Supplementary material for: Phylogenetic characterization and promoter expression analysis of a novel hybrid protein disulfide isomerase/cargo receptor subfamily unique to plants and chromalveolates
Source: Mol Genet Genomics. 2015 Aug 25;291:455–69. doi: 10.1007/s00438-015-1106-7 (PMC4729789; doi:10.1007/s00438-015-1106-7)
Supplement: Supplementary file 1 — Online Resource 1. Sequences of identified plant Erv41p/Erv46p homologs identified by database searches. Corrections made to the original sequences due to available EST data or an alternate gene prediction model are highlighted in yellow (PDF 146 kb) [file 438_2015_1106_MOESM1_ESM.pdf]

Plant Erv41p/Erv46p Homologs

NCBI Reference Sequence: NP_001154394.1
>A.thalianaERV-A1
MAGILNKLRNLDAYPKINEDFYSRTLSGGVITLLSSVVMFLLFFSELRLYLHTVTETKLIVDTSRGETLRI
NFDITFPALACSILSVDAMDISGELHLDVKHDIIKRRLDSNGNTIEARQDGIGATKIENPLQKHGGRLGHN
ETYCGSCYGAEAEEHDCCNSCEDVREAYRKKGWGVTNPDLIDQCKREGFLQRVKDEEGEGCNIYGFLEVNK
VAGNFHFAPGKSFHQSGVHVHDLLAFQKDSFNISHKINRLTYGDYFPGVVNPLDKVEWSQDTPNAMYQYFI
KVVPTVYTDIRGHTIQSNQFSVTEHVKSSEAGQLQSLPGVFFFYDLSPIKVTFTEEHISFLHFLTNVCAIV
GGVFTVSGIIDAFIYHGQKAIKKKMEIGKFS

NCBI Reference Sequence: NP_564162.1
>A.thalianaERV-A2
MVGVMNRLRNLDAYPKINEDFYRRTLSGGVITLASSIVMLILFFSELQLYIHPVTETQLRVDTSRGEKLRI
NFDVTFPALQCSIISLDSMDISGERHLDVRHDIIKRRLDSSGNVIEAKQDGIGHTKIEKPLQKHGGRLEHN
ETYCGSCFGAEASDDACCNSCEEVREAYRKKGWALSDPESIDQCKREGFVQKVKDEEGEGCNVHGFLEVNK
VAGNFHFIPGQSFHQSGFQFHDMLLFQQGNYNISHKVNRLAFGDFFPGVVNPLDGVQWNQGKQSGVYQYFI
KVVPSIYTDVHQNTIQSNQFSVTEHFQNMEAGRMQSPPGVFFYYDLSPIKVIFEEQHVEFLHFLTNVCAIV
GGIFTVSGIVDSFIYHGQRAIKKKMEIGKFN

NCBI Reference Sequence: NP_188868.2
>A.thalianaERV-B
MGVKQALRSIDAFPRAEDHLLQKTQSGAVVSIVGLLIMATLFLHELSYYLNTLTVHQMSVDLKRGETLPIH
VNMTFPSLPCDVLSVDAIDMSGKHEVDLDTNIWKLRLNSHGHIIGTEYISDLVEKGHEHGHSPHKHDGKEE
HKNETETEALNILGFDQAAETMIKKVKQALADGEGCRVYGVLDVQRVAGNFHISVHGLNIYVAQMIFGGSK
NVNVSHMIHDLSFGPKYPGIHNPLDDTNRILHDTSGTFKYYIKIVPTEYRYLSKDVLSTNQYSVTEYFTPM
TEFDRTWPAVYFLYDLSPITVTIKEERRSFLHLITRLCAVLGGTFALTGMLDRWMFRFIESFNKKPSTRA

NCBI Reference Sequence: NP_567765.2
>A.thalianaPDI7
MVSTSKIKSVDFYRKIPRDLTEASLSGAGLSIIAALSMIFLFGMELNNYLAVSTSTSVIVDRSADGDFLRL
DFNISFPSLSCEFASVDVSDVLGTNRLNVTKTIRKFSIDSNMRPTGSEFHAGEVLSLINHGDETGEEIVED
SVPLTGRNFDTFTHQFPILVVNFYAPWCYWCNLLKPSWEKAAKQIKERYDPEMDGRVILAKVDCTQEGDLC
RRNHIQGYPSIRIFRKGSDLKDDNAHHDHESYYGDRDTESLVKMVVSLVEPIHLEPHNLALEDKSDNSSRT
LKKAPSTGGCRVEGYMRVKKVPGNLMVSARSGSHSFDSSQMNMSHVVNHLSFGRRIMPQKFSEFKRLSPYL
GLSHDRLDGRSFINQRDLGPNVTIEHYLQIVKTEVVKSNGQALVEAYEYTAHSSVAHSYYLPVAKFHFELS
PMQVLITENSKSFSHFITNVCAIIGGVFTVAGILDSILHHSMTLMKKIELGKNF

NCBI Reference Sequence: NP_566664.1
>A.thalianaPDI12
MVSSTKLKSVDFYRKIPRDLTEASLSGAGLSIVAALFMMFLFGMELSSYLEVNTTTAVIVDKSSDGDFLRI
DFNISFPALSCEFASVDVSDVLGTNRLNITKTVRKFPIDPHLRSTGAEFHSGLALHNINHGEETKEEFPDG
AIPLTSASFEALSHHFPILVVNFNAPWCYWSNRLKPSWEKAANIIKQRYDPEADGRVLLGNVDCTEEPALC
KRNHIQGYPSIRIFRKGSDLREDHGHHEHESYYGDRDTDSIVKMVEGLVAPIHPETHKVALDGKSNDTVKH
LKKGPVTGGCRVEGYVRVKKVPGNLVISAHSGAHSFDSSQMNMSHVVSHFSFGRMISPRLLTDMKRLLPYL
GLSHDRLDGKAFINQHEFGANVTIEHYLQTVKTEVITRRSGQEHSLIEEYEYTAHSSVAQTYYLPVAKFHF
ELSPMQILITENPKSFSHFITNLCAIIGGVFTVAGILDSIFHNTVRLVKKVELGKNI

NCBI Reference Sequence: NP_175508.2
>A.thalianaPDI13
MVSTSKIKSVDFYRKIPRDLTEASLSGAGLSIVAALAMLFLFGMELSSYLAINTSTSVIVDKSSDGDFLNI
DFNISFPALSCEFASVDVSDVFGTHRLNISKTIRKVPIDPHLRATAEEFHSTSDLHLINHGDEDHGDNSTY
ADIPLTGAAFEKFTHHFQILVVNFYAPWCYWSNRLKPSWVKASQITRERYNPGTDDRVLLGSVDCTEEPTL
CKSNHIQGYPSIRIFRRGSGLREDHGNHEHESYYGDRDTDSLVKMVEELLKPIKKEDHKLALDGKSDNAAS
TFKKAPVSGGCRIEGYVRAKKVPGELVISAHSGAHSFDASQMNMSHIVTHLTFGTMVSERLWTDMKRLLPY
LGQSYDRLNGKSFINERQLDANVTIEHYLQIIKTEVISRRSGQEHSLIEEYEYTAHSSVARSYHYPEAKFH
FELSPMQVLISENPKSFSHFITNVCAIIGGVFTVAGILDSIFQNTVRMVKKIELGKNI

NCBI Reference Sequence: XP_003579883.1
>B.distachyonERV-A1
MDGLMSKLRNLDAYPKVNEDFYSRTLSGGVITLASSFVMLLLFVSELRLYLHAVTETTLRVDTSRGEKLRI
NFDITFPALQCSIISIDVMDISGQEHLDVKHDVFKQRIDANGNVIATKQDAVGGMKVEKPLQMHGGRLEHN
ETYCGSCYGAEEPGEQCCNSCEDVREAYRKKGWGVSNPDSIDQCKREGFLQTIKDEEGEGCNIYGFVEINK
VAGNFHFAPGKSFQQSNVHVHDLLPFQKDSFNVSHKINKLSFGEPFPGVVNPLDGAHWFQHSPYGMYQYFV
KVVPTVYSHINEQIILSNQFSVTEHARSSESVRMQALPGVFFFYDLSPIKVTFTERHVSFLHFLTNVCAIV
GGVFTVSGIIDSFVYHGQRAITKKREIGKFN

NCBI Reference Sequence: XP_003558026.1
>B.distachyonERV-A2
MDLWNKLRSLDAYPKVNEDFYSRTLSGGLITIASSLAILLLFFSEIRLYLYSATESKLTVDTSRGERLHIN
FDVTFPALPCSLVAIDTMDVSGEQHYDIRHDIFKKRIDHLGNVIESRKDGVGSPKIERPLQNHGGRLDHNE
AYCGSCYGSEESDDQCCNSCEEVRDAYRKKGWALTNVESIDQCKREGFVQRLKDEQGEGCNIHGFVDVNKV
AGNFHFAPGKHLDQSFNFLQDMLNFQPENYNISHKINKLSFGKEFPGVVNPLDGVEWKQEQATGLTGMYQY
FVKVVPTIYTDIRGRKIHSNQFSVTEHFREAIGFPRPPPGVYFFYEFSPIKVDFTEENTSLLHFLTNICAI
VGGIFTVAGIIDSFVYHGHRAIKKKMEIGKLG

NCBI Reference Sequence: XP_003568216.1
>B.distachyonERV-A3
MDGFLQKLKGLDAYPKVNEDFYKRTLSGGVVTLVSAVVMLLLFISETSSYLNSATETKLVVDTSRGERLRV
NFDITFPSIPCTLLSVDTRDISGEQHQDIRHDIEKKRLNSHGNVIESRKEGIGGAKIERPLQKHGGRLDKG
EQYCGTCYGAEESDEQCCNSCDEVREAYKKKGWALTNPDLIDQCAREDFVERVKTQHGEGCSVHGFLDVSK
VAGNFHFAPGRGFYESNVDVPELSSLEGGFNITHKINKLSFGTEFPGVVNPLDGAQWTQPASDGTYQYFIK
VVPTNYTDTRGRKIDSNQFSVTEHFRDGNVHPRPQPGVFFFYDFSPIKVIFTEENKSFLHYLTNLCAIVGG
IFTVSGIIDSFIYHGQKALKKKMEIGKYR

NCBI Reference Sequence: XP_003558212.1
>B.distachyonERV-B
MGRIPSLKNFNAFPHAEDHLLKKTYSGAIVTIFGLIIMFTLFVHELKFYLTTYTMHQMSVDLKRGETLPIH
INMSFPSLPCEVLSVDAIDMSGKHEVDLHTNIWKLRLDKYGTIIGTEYLSDLVEKEHGAHHHDNGHEHHDE
EKKPEHTFNEDADKMVKSVRQALENGEGCRVYGMLDVQRVAGNFHISVHGLNIYVAEKIFEGSSHVNVSHV
IHELSFGPKYPGIHNPLDDTTRILHDASGTFKYYIKVVPTEYRYLSKQVLPTNQFSVTEYFVPIRPADRSW
PAVYFLYDLSPITVTIKEERRNFLHFITRLCAVLGGTFAMTGMLDRWMYRIIESVSSSKPRSVLR

NCBI Reference Sequence: XP_010237612.1
>B.distachyonPDI-C
MISSSKLKSVDFYRKIPRDLTEASLSGAGLSIVAALAMVFLFGMELSSYLAVNTTTSVIVDRSSDGEFLRI
DFNMSFPALSCEFASVDVSDVLGTNRLNITKTVRKFSIDRNLVPTGSEFHSGPIPTVNKHGDDVEEYHADG
SVALSSRNFDSYSHQYPILVVNFYAPWCYWSNRLKPSWEKTAKIIKERYDPEMDGRILLAKVDCTEEGELC
KRHHIQGYPSIRIFRKGSDMKENQGHHDHESYYGERDTDSLVAAMETYVGNLPKEAHMLALDDKSNKTVDP
AKRPAPMTSGCRVEGFVRVKKVPGSVIISARSGSHSFDPSQINVSHYVTQFSFGNRLSPNMFSELKRLIPY
VGGHHDRLAGQSYIVKHGDNNANVTIEHYLQIVKTELVTLRSSKELKVFEEYEYTAHSSLVHSFYVPVVKF
HFEPSPMQVLVTELPKSFSHFITNVCAIIGGVFTVAGILDSILHNTLRLVKKVELGKDI

NCBI Reference Sequence: XP_009107911.1
>B.rapaERV-A1
MAGILNKLRNLDAYPKINEDFYSRTLSGGVITLVSSVVMFLLFFSELRLYLHSVTETKLVVDTSRGETLRI
NFDMTFPALACSILSVDAMDISGELHLDVKHDVVKRRLDSHGNIIESRQDGIGAAKIEKPLQKHGGRLEHN
ETYCGSCYGAEAEEHDCCNSCEDVREAYRKKGWGVTNPDLIDQCKREGFLQRVKDEEGEGCNIYGFLEVNK
VAGNFHFAPGKSFHQGGVHVHDLLAFQKDSFNISHKINRLTFGDYFPGVVNPLDKVQWSQDTPNAMYQYFI
KVVPTVYTDISGHTIQSNQFSVTEHVKSSEAGQLQSLPGVFFFYDLSPIKVTFTEEHISFLHFLTNVCAIV
GGVFTVSGIIDAFIYHGQKAIKKKMEIGKFS

NCBI Reference Sequence: XP_009106456.1
>B.rapaERV-A2

MAGVMNRLKNLDAYPKINDDFYSRTLSGGLITLASSLVMLILFFSELRLYLHPVTESQLRVDTSRGEKLRI
NFDATFPALACSIISLDSMDISGEQHLDVRHDMSKRRLDAYGNVIETRQDGIGHTKIENPLQKHGGRLEHN
ETYCGSCYGAEASDDECCNSCEEVREAYRKKGWAMTDPDIIDQCKREGFVQRVKEEEGEGCNIFGFLEVNK
VAGSFHFVPGKTFRQSGFHLQDLLVFQGDSYNISHKVNSLTFGDRFPGVVNPLDGVQWTQETLNGMYQYFI
KVVPTEYTDVRGHVIQSNQFSVTEHFEKTEAGRTQSLPGVFFFYDLSPIKVIFTEQHVEFLHFLTNVCAIV
GGIFTVSGIIDSFVYHGQRAIKKKMEIGKFG

NCBI Reference Sequence: XP_009103253.1
>B.rapaERV-A3
MNRLRNLDAYPKINEDFYNRTLSGGLITLFSSLLMLILFFSELRLYMHPVTETQLRVDTSRGEKLRINFDV
TFPALQCSIISIDTMDISGERHLDVRHDIIKRRLDSHGNVIESKQDGIGHTKIEKPLQKHGGRLEHNETYC
GSCFGAETTDDTCCNSCEEVREAYRKKGWALSDPESIDQCKREGYVQKVKDEEGEGCNLHGFLEVNKVAGN
FHFVPGQSFHQSGFQFGDLIFFQQGNYNISHKVNRLAFGDFFPGVVNPLDGVQWNQEKQNGVYQYFVKVVP
SIYTDVHGHTIQSNQFSVTEHFQKMEAGRMQSPPGVFFYYDLSPIKVIFEEQHVEFLHFLTNVCAIVGGIF
TVSGIVDSFIYHGQRAIKKKMEIGKFN

NCBI Reference Sequence: XP_009145365.1
>B.rapaERV-A4
MNRLRNLDAYPKINEDFYKRTLSGGVITLVSSVVMLILFFSELRLYMHPVTESHLRVDTTRGEKLRINFDV
TFPALQCSIISIDTMDISGERHLDVRHDIFKRRLDSHGNVIEAKQGGIGHTKIEKPLQKHGGRLEDNEKYC
GSCFGAEETDDACCNSCEEVREAYRKKGWGLSDPESIDQCQREGYVQKVKDEEGEGCNVHGFVEVNKVAGN
FHFVPGQSFHQSGFQFHDMIMFQQGNYNISHKVNRLAFGDFFPGVVNPLDGVQWNQDKQNGVYQYFIKVVP
TIYTDVHGKTIQSNQFSVTEHFQNAEAGRMQSPPGVFFYYDLSPIKVIFEEQHVEFLHFLTNVCAIVGGIF
TVSGIVDSFIYHGQRAIKKKMEIGKFN

NCBI Reference Sequence: XP_009135799.1
>B.rapaERV-B1
MGVKQALRSIDAFPRAEDHLLQKTQSGAVVSIVGLLIMATLFLHELSYYLNTLTVHQMSVDLKRGETLPIH
VNMTFPSLPCDVLSMDAIDMSGKHEVDLDTNIWKLRLNSHGHIIGTEYISDLVEKEHDHSSHKHDGKEEHK
NETEAVNTLGFDEAAETMIKKVKQALADGEGCRVYGVLDVQRVAGNFHVSVHGLNIYVAQMIFGGSKNVNV
SHMIHDLSFGPKYPGIHNPLDDTNRILRDTSGTFKYYIKIVPTEYRYLSKDVLTTNQYSVTEYYTPMNEFD
RTWPAVYFLYDLSPITVTIKEERRSFLHLITRLCAVLGGTFALTGMLDRWMFRLIESFTKKPSTRSIHK

NCBI Reference Sequence: XP_009145393.1
>B.rapaERV-B2
MGVKQALRSIDAFPRAEDHLLQKTQSGAVVSIVGLLIMVILFLSELSYYLNTLTVHQMSVDLKRGETLPIH
VNMTFPSLPCDVLSMDAIDMSGKHEVDLDTNIWKLRLNSHGHIIGTEYISDLVEKEHDHSSHKHDEHKNET
DALNLLGFDEAAETMIKKVKQALADGEGCRVYGVLDVQRVAGNFHISVHGLNIYVAQMIFGGSKNVNVSHM
IHDLSFGPKYPGIHNPLDGTNRILHDTSGTFKYYIKIVPTEYRYLSKDILTTNQYSVTEYYTPMNEFDRTW
PAVYFLYDLSPITVTIKEERRSFLHLITRLCAVLGGTFALTGMLDRWMFRIIESFTKKPSTRSIHK

NCBI Reference Sequence: XP_009145612.1
>B.rapaPDI-C1
MVSPTKLKSMDFYRKIPRDLTEASLSGAGLSIVAALVMMLLFGMELSSYLAVNTTTAVVVDKSADGDFLRI
HFNISFPALSCEFASVDVSDVLGTNRLNITKTIRKFPIDPHLKTTGEEFHSGHGSHDINHGEETKEEIPDG
SVPLVSSSFDSFSKHFPLLIVNFNAPWCYWSNRLKPSWEKASSIIYHKYNPETDGRVLLGSVDCTEEAELC
KRNHIQGYPSIRIFRKGSDLKEDHGHHEHESYYGDRDTDSIVKMVDELVAPIHPETHKLDLDGISNKTLKH
LKKAPVTGGCRVEGYVRVKKVPGNLIISAHSGAHSFDSSKMNMSHVVSHLSFGRMFSPRLLTDMRRLLPYI
GQSHDKLNEKAFINQHEFGANVTIEHYLQVVKTEVITRRTAQEHSLVEEYEYTAHSSIAQTYYLPVAKFHF
ELSPMQIMITENPKSFSHFITNLCAIIGGVFTVAGILDSIFHNTIRLVKKVELGKNF

NCBI Reference Sequence: XP_009135698.1
>B.rapaPDI-C2
MVSPTKLKSVDFYRKIPRDLTEASLSGAGLSIVAALVMMLLFGMELSSYLEVTTTTAVVVDKSSDGDFLRI
DFNISFPALSCEFASLDVNDVLGTNRLNITKTVRKFPIDPHLKATGGEFHSGLASHHINHGEEIKQEFPDG
AIQLTNGGFQSLSHHFPLLIVNFNAPWCYWSNRLKPSWEKAATIIKQRYNPDTDGRVLLGSVDCTEEPALC
RRNHIQGYPSIRIFRKGNDLKEDHGHHEHESYYGDRDTESIVKMVDELVAPIHPETHKLALDWGISNDTAK

LLKKAPVTGGCRVEGYVRVKKVPGNLVISAHSGAHSFDSSQMNMSHVVTHLSFGRMIDTRLLTDLKRLLPY
LGQSHDKLDEKAFINQHEFGANVTIEHYLQIVKTEVITRRYGQEHSLTEEHEYTAHSSITQTYYLPVAKFH
FELSPMQILITENPKSFSHFITNLCAIIGGVFTVAGIIDSVLHNTIRLIKKVELGKNI

NCBI Reference Sequence: XP_009137636.1
>B.rapaPDI-C3
MVSTSRIKSVDFYRKIPRDLTEATLSGAGLSIVAALSMLFLFGMELNNYLAVSTTTSIIVDRSSDGDFLRM
DFNISFPSLSCEFASVDVSDVLGTNRLNVTKTIRKFSIDSNLRPTGSEFHSGEVLSHVNHDEAGEEVVEDS
VSLTSRNFDTLLHQFPISVVNFYAPWCYWCNLLKPSWEKAAKQIKERYDPEMDGRVILAKVDCTQEADLCR
KNHIQGYPSIRIFRQGSDLKDNAHHDHESYYGDRDTESLVKMVIGLVEPIHLEPHKLALEDKSDNASKTLK
KAPSTGGCRIEGYIRVKKVPGNLMVSARSGSHSFDSTQMNMSHVVNHLSFGRKILPQTFTDLKRLSPYLGQ
SHDRLNGRPFINQRDLGPNVTIEHYLQIVKTEVLKSNGHAMVEEYEYTAHSSVAHSYYLPVAKFHFELSPM
QVLITENSRSFSHFITNVCAIIGGVFTVAGILDSILHQTMTLMKKIELGKNF

NCBI Reference Sequence: XP_009109150.1
>B.rapaPDI-C4
MISPRKIKSVDFYRKIPRDLTEASLSGAGLSIVAALSMLLLFGMELSSYLTVSTTTSIIIDRSSDGDFLRM
DFNISFPSVSCEFASVDVSDVLGTNRLNVTKTIRKFSIDSNLRPTGSEFHSGEFLSRVNHGDESAEELVEG
SVSLGARNFDTFLHQYPISVVNFYAPWCYWCNLLKPSWEKAANQIKERYDPEMDGRVILAKVDCTQEADLC
RRNHIQGYPSIRIFRKGSDLRDDNAHHDHESYYGDRDTESLVKMVIGLVEPIHLEPHKLALEDKSGNASKT
LKKAPSTGGCRIEGYMRVKKVPGNLMVSARSESHSFDTSQMNMSHVVNHLSFGKRILPEAFSDLKRLAPYL
GGSHNRLDDRSFINQHDLGPNVTIEHYLQIVKTEVLKSNGHAMIEEYEYTAHSSVAHTYYLPVAKFHFELS
AMQVLITENSKSFSHFITNVCAIIGGVFTVAGILDSILHQTMTLMKKIELGKNF

NCBI Reference Sequence: XP_009147793.1
>B.rapaPDI-C5
MVSTTKIKSVDFYRKIPRDLTEASLSGAGLSIIAALAMMFLFGMELSTYLAVTTQTSVVVDNSSDDDFLQI
DFNVSFPALSCEFATFEVSDVLSTNRLNLTKTIKKVPIDPHLRDTGEEYHPTPDSDLINHGDEHHDDNTYA
AIPLSGGTFDKISHKFPILVVNFYAPWCYWSSRLRPSWEKAAEITRQKYGPENDGRVLLGSVDCTEEPTLC
TKYHIQGYPSIRIFHNGSDLRGDDGHQEHDSYHGNRDTESLVKMVEELLRPIKKFDGTTNHAASRIRKAPV
SGGCRIEGYVRAKKVPGELVISAVSGSHSFDASRMNMTHFVNHLSFGRLISDRLLTDMKRLLPYLGLSHDR
LNGKWFVNEGKFAANVTIEHYLQVVKTEVVSRRFGQEHSVIEEYEYTAHSSVAHGYYYPVAKFHFDLSPMQ
VLISENPKSFSHFITNVCAIIGGVFTVAGILDSIFQSTYGIMKKVELGKNF

NCBI Reference Sequence: XP_009144931.1
>B.rapaPDI-C6
MVSTTKIKSVDFYRKIPRDLTEASLSGAGLSIIAALAMVFLFGMELSTYLAVTTNTSVIVDNSSDGDFLRI
DFNVSFPSLSCEFASVDVSNVLGTKRLNLTKTIKKVPIDPYLRATGAEVHSTSGLHLINHGDEDHGNNTYA
AIPLTGATFDKFSHHFQILVVNFYAPWCYWSNRLKPSWEKAAEITRQRYNPETDGRVLLGSVDCTEETTLC
KRNHIQGYPSIRIFRKGSDLKEDHGHHEHESYHGDRDTESILKMVEELLKPIKKEDHKLALDGKTDNVVSG
IKKAPVSGGCRIVGYVRAKKVPGEIIISAHSGAHSFDASQMNMSHYVSHLTFGKMISERLLTDMKRLMPYL
GLSHDRLNSKWFVNEGQFAANVTIEHYLQVVKTEVVSRRFGQEHSVIEEYEYTAHSSVAHGYYYPVAKFRF
DLSPMQVLISENPKSFSHFITNVCAIIGGVFTVAGILDSIFQNTFRLVKKIELGKNI

NCBI Reference Sequence: XP_006305051.1
>C.rubellaERV-A1
MAGILNKLRNLDAYPKINEDFYSRTLSGGVITLLSSVVMFLLFFSELRLYLHTVTETKLIVDTSRGETLRI
NFDITFPALACSILSVDAMDISGELHLDVKHDIIKRRLDSNGNTIEARQDGIGATKIEKPLQKHGGRLEHN
ETYCGSCYGAEAEEHDCCNSCEDVREAYRKKGWGVTNPDLIDQCKREGFLQRVKDEEGEGCNIYGFLEVNK
VAGNFHFAPGKSFHQSGVHVHDLLAFQKDSFNISHKINRLTYGDYFPGVVNPLDKVQWSQDTPNAMYQYFI
KVVPTVYTDIRGHTIQSNQFSVTEHVKSSEAGQLQSLPGVFFFYDLSPIKVTFTEEHISFLHFLTNVCAIV
GGVFTVSGIIDAFIYHGQKAIKKKMEIGKFN

NCBI Reference Sequence: XP_006305055.1
>C.rubellaERV-A2
MVGVMNRLRNLDAYPKINEDFYRRTLSGGVITLVSSLLMLVLFFSELQLYIHPVTETQLRVDTSRGEKLRI
NFDVTFPALQCSIISLDSMDISGERHLDVRHDIIKRRLDSTGNVIEAKQDGIGHTKIEKPLQKHGGRLEHN

ETYCGSCFGAELSDDACCNSCEEVREAYRKKGWALSDPESIDQCKREGFVQKVKDEEGEGCNIHGFLEVNK
VAGNFHFIPGQSFHQSGFQFHDMLLFQQGNYNISHTVNRLAFGDFFPGVVNPLDGVQWNQGKQSGVYQYFI
KVVPSIYTDVHRNTIQSNQFSVTEHFQGMEAGRMQSPPGVFFYYDLSPIKVIFEEQHVEFLHFLTNVCAIV
GGIFTVSGILDSFIYHGQRAIKKKMEIGKFN

NCBI Reference Sequence: XP_006298011.1
>C.rubellaERV-B
MGVKQALRSIDAFPRAEDHLLQKTQSGAVVSIVGLLIMATLFLHELSYYLNTLTVHQMSVDLKRGETLPIH
VNMTFPSLPCDVLSVDAIDMSGKHEVDLDTNIWKLRLNSHGHIIGTEYISDLVEKGHEHGHSSHKHDGKEE
HKNETEIETLNSLGFDQAAETMIKKVKQALADGEGCRVYGVLDVQRVAGNFHISVHGLNIYVAQMIFGGSK
NVNVSHMIHDLSFGPKYPGIHNPLDDTNRMLHETSGTFKYYIKIVPTEYRYLSKDVLSTNQYSVTEYYTPM
TDFDRTWPAVYFLYDLSPITVTIKEERRSFLHLITRLCAVLGGTFALTGMLDRWMFRLIESFNKNTNTRSM
H

NCBI Reference Sequence: XP_006297547.1
>C.rubellaPDI-C1
MVSSTKLKSVDFYRKIPRDLTEASLSGAGLSIVAALFMVFLFGMELSSYLEVNTTTSVIVDKSTDGDFLRI
DFNISFPALSCEFASVDVSDVLGTNRLNITKTIRKFPIDPHLRSTGAEFHSGHTLHHINHGEETKEEFPDG
AIPLTSGSFESLSHHFPILVVNFNAPWCYWSNRLKPSWEKAANIIKQKYDPETDGRVLLGNVDCTEEPALC
KRNHIQGYPSIRIFRKGSDLREDHGHHEHESYYGDRDTKSIVKMVDELVAPIHPETHKVALDGKSNDTVKH
LKKAPVTGGCRVEGYVRVKKVPGNLVISAHSGAHSFDSSQMNMSHVVSHLSFGRMISPRLLTDMKRLLPYL
GQSHDRLDGKAFINQHEFGANVTIEHYLQIVKTEVITRRSGLEQAVIEEYEYTAHSSVAQTYYLPVAKFHF
ELSPMQILITEDPKSFSHFITNLCAIIGGVFTVAGILDSIFHNTIRLIKKVELGKNI

NCBI Reference Sequence: XP_006283569.1
>C.rubellaPDI-C2
MVSTSKIKSVDFYRKIPRDLTEATLSGAGLSIIAALSMLFLFGMELNNYLAVSTSTSVIVDRSSDGDFLRL
DFNISFPSLSCEFASLDVSDVLGTNRLNVTKTIRKFSIDSNMRPTGAEFHAGEVLSLINHGDETGEEVAED
SVSLTGRNFETFTHQFPILVVNFYAPWCYWCNLLKPSWEKAAKQIKERY-----------------------
---DPEMDGRVIVAKVDCTQEGDLCRRNHIQGYPSIRIFRKGSDLKDDNAHHEHESYYGDRDTESLVKMVV
SLVEPIHLEPHNLALEDKSGNSSKTLKKAPSTGGCRIEGYMRVKKVPGNLMVSARSGSHSFDSSQMNMSHV
VNHLSFGRRILPQKFSELKRLSPYLGRNHDRLDGRPFINQRDLGPNVTIEHYLQIVKTEVVKSNGQVLIEE
YEYTAHSSVAQSYYLPVAKFHFELSPMQVLITENSKSFSHFITNVCAIIGGVFTVAGILDSILHHTMTLMK
KIELGKNF

NCBI Reference Sequence: XP_006307368.1
>C.rubellaPDI-C3
MVSTTKIKSVDFFRKIPRDLTEASLSGAGLSIVAALAMLFLFGMELSSYLAVNTSTSVIVDKSSDGDILNI
DFNISFPALSCEFASVDVSDVLGTHRLNLTKTIRKVPIDPHLRATGTEFHSTSGLHLINHGDEDHEENTYI
DIPLTGATFEKYKHHFQILVVNFYAPWCYWSNRLKPSWEKASQITRERYDPETDDRVLLGSVDCTKESTLC
KSNHIQGYPSIRIFRKGSDLRDDHGHHDHESYYGDRDTDSLVKMVEELLKPIKKEDHKLALDGKSENAAST
VKKAPVSGGCRIEGYVRAKKVPGELVISAHSGAHSFDASQMNMSHIVTHLTFGTMVSERLWTDMKRLLPYL
GQSHDRLNGKSFINQRQLDANVTIEHYLQIVKTEVISRRFGREHSLIEEYEYTAHSNVAHSYHYPEAKFHF
ELSPMQVLISENPKSFSHFITNVCAIIGGVFTVAGILDSIFQNTVRLVKKIELGKNI

NCBI Reference Sequence: XP_004142610.1
>C.sativusERV-A1
MESLMNKIRKLDAYPKISEDFYNRTLSGGFITIASSIIMFLLFFSELRLYVHTATETKLIVDTSRGEHLRI
NFDVTFPALPCSVLSLHAMDISGEQHLDVKHDIVKKRIDYQGNVIDSRPDGIGSTEIERPLQKHGGRLKQN
ETYCGSCYGASGEDCCNSCQDVREAYHRKGWALSHPDLIDQCKREGFFQRVKNEEGEGCNIYGFLEVNKVA
GNFHFAPGRGFQLSYFQIHNPLASFQWDAFNISHRINRLTFGDDFPGVVNPLDGVQWNQGTLSGMFQYFIK
VVPTVYKAVNGKAIKSNQFSVTQHLRGIDGESFQALHGVFFFYDLSPIKVTFTEEHISFFHFLTNVCAIVG
GVFTISGILDSIIYHGQKAIKKKMALGKFT

NCBI Reference Sequence: XP_004150658.1
>C.sativusERV-A2

MDNIISKLRNLDAYPKINEDFYSRTLSGGVITLSSSILMLLLFISELRLYLHAVTETKLVVDTSRGETLRI
NFDVTFPALPCSLLSLDAMDISGEQHLDVKHDIIKKRLDSHGNAIEARPDGIGAPKIEKPLQRHGGRLEHN
ETYCGSCFGAESADDDCCNSCEEVREAYRKKGWALSNPDLIDQCKREGFLQRIKDEDGEGCNIYGFLEVNK
VAGNFHFAPGKSFQQSNVHVHDLLAFQKDSFNISHKINRLAFGEYFPGVVNPLDSVQWKQETPSATYQYFI
KVVPTVYNSVSGYTIQSNQFSVTEHVRTAEVGRLQSLPAVFFFYDLSPIKVTFTEEHVSFLHFLTNVCAIV
GGVFTVSGILDSFIYHGQKVIKKKMEIGKFS

NCBI Reference Sequence: XP_004137169.1
>C.sativusERV-A3
MDAIFNKLRNLDAYPKINEDFYRRTFSGGLITLASSFFMLFLFFSELRMYLHAKTETQLVVDTSRGGELHI
NFDLSFPAIPCSILSLDAIDISGEQHLDIRHNIIKKRIDHLGTVIEARPDGIGAPKIEKPLQKHGGRLEHN
ETYCGSCFGAEASDDDCCNSCEEVREAYRKKGWAITNQDLIDQCQREDFIQKVKDEEGEGCNIEGSLEVNK
VAGSFHFVPGKSFYQSSFNFLGLLALQTSDYNVSHRINRLAFGNHYDGLVNPLDGVHWEYNEQNVMHQYFV
KVVPTIYKNIRGRTVHSNQYSVTEHFKSVEFGSSQSIPGVFFYYDLSPVKVTYTEEHVPFLHFMTHICAII
GGVFSVAGIIDAFIYHGQRKMKKKVEIGKFG

NCBI Reference Sequence: XP_011655240.1
>C.sativusERV-B
MGLKQTIKSLDAFPRAEEHLLQKTQTGAFVSVVGLVIMATLFLHELRYYLSTYTVHQMSVDLKRGETLPIH
INMTFPSLPCDVLSVDAIDMSGKHEVDLDTNIWKLRLNSHGQIIGTEYLSDLVEKEHVDHKHDHDHDKEKD
HPHIHGFDQAAENLVKKVKQALEEAQGCRVYGVLDVQRVAGNFHISVHGLNIFVAQMIFGGSKHVNVSHMI
HDLSFGPKYPGIHNPLDGTVRILRDTSGTFKYYIKIVPTEYKYISKAVLPTNQFSVTEYFSPMTDSDRSWP
AVYFLYDLSPITVTIKEERRSFLHFITRLCAVLGGTFAVTGMLDRWMFRFLEALTKPKRRTR

NCBI Reference Sequence: XP_004151953.1
>C.sativusPDI-C
MISSTKLKSVDFYRKIPRDLTEATLSGAGLSIVAALSMVFLFGMELSNYLSVSTSTSVIVDNSTDGDFLRM
DFNISFPALSCEFAAVDVNDVLGTNRLNITKTIRKFSIDSNLRSTGSEFHSGPLSNLIKHGDEVDEEANEG
SAVLNTRNFDRYANQHPILVVNFYAPWCYWSNRLKPSWEKAAKTIRERYDPELDGRILMAKVDCTEEGDLC
RKHHIQGYPSIRIFRKGSDVRDDHGHHDHESYYGDRDTDSLVKTMEDLIAPLPAGSQKLALEDKSNNETGN
VKRPAPSAGGCRIEGYVRVKKVPGSLVIAARSESHSFDASQMNMSHIISHLSFGRKISPKAFSDAKQLIPY
IGISHDRLNGRSFINQRDLGANVTIEHYLQIVKTEVLTRRSGKLLEEYEYTAHSSVSQSLYIPVVKFHFVL
SPMQVVITENQKSFSHFITNVCAIIGGVFTVAGILDALLHNTIRLMKKVELGKNF

NCBI Reference Sequence: XP_006403044.1
>E.salsugineumERV-A1
MAGILNKLRNLDAYPKINEDFYSRTLSGGVITLFSSVVMFLLFFSELRLYLHTVTETKLVVDTSRGETLRI
NFDITFPALACSILSVDAMDISGELHLDVKHDIIKRRLDSHGNTIEARQDGIGATKIENPLQKHGGRLEHK
ETYCGSCYGAEAEEHDCCNSCEDVREAYRKKGWGVTNPDLIDQCKREGFLQRVKDEEGEGCNIYGFLEVNK
VAGNFHFAPGKSFHQAGVHVHDLLAFHKDSFNISHKINRLTFGDYFPGVVNPLDRVQWGQDTPNAMYQYFI
KVVPTVYTDISGHSIHSNQFSVTEHVKSSEAGQLQSLPGVFFFYDLSPIKVTFTEEHISFLHFLTNVCAIV
GGVFTVSGIIDAFIYHGQKAIKKKMEIGKFS

NCBI Reference Sequence: XP_006416221.1
>E.salsugineumERV-A2
MVGVMNRLRNLDAYPKINEDFYRRTLSGGVITLVSSIVMLILFFSELQLYLHPVTETQLRVDTSRGEKLRI
NFDVTFPALQCSIISIDTMDISGERHLDVRHDIIKRRLDSHGNVIEAKQDGIGHTKIEKPLQKHGGRLEHN
ETYCGSCFGAEMTDEACCNSCEEVREAYRKKGWALSDPESIDQCKREGFVQKVKDEEGEGCNVHGFLEVNK
VAGNFHFVPGQSFHHSGFQFHDLIMFHQGNYNISHKVNRLAFGDFFPGVVNPLDGVQWNQEKQNGVYQYFI
KVVPSVYTDAHENTIQSNQFSVTEHFQNAEAGRMQSPPGVFFYYDLSPIKVIFEEQHVEFLHFLTNVCAIV
GGVFTVSGIVDSFIYHGQRAIKKKMEIGKFN

NCBI Reference Sequence: XP_006390025.1
>E.salsugineumERV-A3
MPGVMNRLRNLDAYPKINDDFYSRSLSGGLITLVSSIFMLILFFSELRLYLHPVTESQLRVDTSRGEKLRI
NFDVTFPALACSIISLDSMDISGEQHLDVRHDIAKRRLDSSGNVIETRQDGIGHTKIENPLQRHGGRLEHN
ETYCGSCYGAEATDEECCNSCEEVGEAYRKKGWAMSDPEIIDQCKREGFVQRVKDEEGEGCNIYGFLEVNK

VAGSFHFVPGKTFRHSGFHLQDLLAFQGDTYNISHKVNRLTFGDYFPGVVNPLDSVQWTQETLNGMYQYFI
KVVPTEYTDVKEHIIQSNQFSVTEHFEKTEAGHTQSLPGVFFFYDLSPIKVIFTEQHVEFLHFLTNVCAIV
GGIFTVSGIIDSFIYHGQRAIKKKMEIGKFG

NCBI Reference Sequence: XP_006406186.1
>E.salsugineumERV-B
MGVKQALKSIDAFPRAEDHLLQKTQSGAVVSIVGLIIMATLFLHELSYYLNTLTVHQMSVDLKRGETLPIH
VNMTFPSLPCDVLSMDAIDMSGKHEVDLDTNIWKLRLNSHGHIIGTEYISDLVEKEHDHSTHKHEHKNETE
ALNVLGFDQAAETMIKKVKQALADGEGCRVYGVLDVQRVAGNFHVSVHGLNIYVAQMIFGSSKNVNVSHMI
HDLSFGPKYPGIHNPLDDTNRILRDTSGTFKYYIKVVPTEYRYLSKDVLSTNQYSVTEYYTPMNEFDRTWP
AVYFLYDLSPITVTIKEERRSFLHLITRLCAVLGGTFALTGMLDRWMFRFIESFTKKPSTRSIHK

NCBI Reference Sequence: XP_006406369.1
>E.salsugineumPDI-C1
MVSSTKIKSVDFYRKIPRDLTEASLSGAGLSIVAALVMMLLFGMELSSYLEVSTTTAVVVDNSADGDFLRI
TFNISFPALSCEFASVDVSDVLGTNRLNITKTVRKFPIDPHLRTTGAEFHSGLALHDINHGEESKEEFPDG
AIPLTSGSFESYSHHFPILIVNFYAPWCYWSNRLKPSWEKAANIIKQRYDPETDGRVLLGNVDCTEEAALC
RRNHIQGYPSIRIFRKGSDLREDHGHHEHESYYGDRDTDSIVKMVDGLVAPIHPETHKLALDGISNDTAKN
LKKAPVTGGCRVEGYVRVKKVPGNLVISAHSGAHSFDSTQMNMSHVVTHLSFGRMISPRLLTDMRRLLPYL
GQSHGRLDEKAFINQHEFGANVTIEHYLQIVKTEVITRKSGQEHSLIEEYEYTAHSSVAQTYYLPVAKFHF
ELSPMQILITENPKSFSHFITNLCAIIGGVFTVAGILDSIFHNTIRLVKKVELGKNI

NCBI Reference Sequence: XP_006413109.1
>E.salsugineumPDI-C2
MVSTSKIKSVDFYRKIPRDLTEATLSGAGLSIVAALSMLFLFGMELNNYLAVSTTTSIIVDRSSDGDFLRM
DFNISFPSLSCEFASVDVSDVLGTNRLNVTKTIRKFSIDSNLRPTGTEFHSGEVLSLINHGDEAGEELVED
SVSLTGRNFDTLLHQFPISVVNFYAPWCYWCNLLKPSWEKAAKQIKERYDPEMDGRVILAKVDCTVESDLC
RRNHIQGYPSIRIFRKGSDLRDDNAHHDHESYYGDRDTESLVKMVIGLVEPIHLEPQRLALEDKSDNASKI
LKKAPSTGGCRIEGYMRVKKVPGNLMVSARSGSHSFDSSQMNMSHVVNHLSFGRRILPQTFADLKRLSPYL
GRSHDRLDGRPFINQRDLGPNVTIEHYLQIVKTEVVKSNGQALIEEYEYTAHSSVAQSYYLPVAKFHFELS
PMQVLITETSKSFSHFITNVCAIIGGVFTVAGILDSILHQTMTLMKKIELGKNF

NCBI Reference Sequence: XP_006393093.1
>E.salsugineumPDI-C3
MVSTTKIKSVDFYRKIPRDLTEASLSGAGLSIVAALAMVFLFGMELSNYLAVSTNTSVIVDKSSDGDFLRI
DFNVSFPSLSCEFASVDVSDVLGTSRLNLTKTIRKVPIDAHLRATGAEFHSTTGLDLINHGDEDHHENNSY
AAIPLTGATFDKFSHHFPILVVNFYAPWCYWSNRLKPSWEKAAEITRQKYDPEDDGRVLLGSVDCTEEPTL
CKRNHIQGYPSIRIFRKGSDLREDHGHHEHESYYGDRDTESLLKMVEELLKPIKKEDHKLPLDGISVNTAS
SIKKAPVSGGCRIEGYVRAKKIPGEVVISAHSGAHSFDASQMNMSHFVTHLSFGKLISERLLTDMKRLLPY
LGQSHDRLNGKWFLNQGQFAANVTIEHYLQIVKTEVVSRRFGQEHSLIEEYEYTAHSNVAHSYYYPVAKFH
FELSPMQVLISENPKSFSHFITNVCAIIGGVFTVAGILDSIFQNTIRLVKKVELGKNI

NCBI Reference Sequence: XP_003544786.1
>G.maxERV-A1
MDSIMSKLRNLDAYPKINEDFYSRTLSGGVITLASSILMLLLFFSELRLYLHAVTETKLVVDTSRAETLRI
NFDVTFPALPCSILSLDAMDISGEQHLDVKHDIIKKRLDSHGNVIETRQEGIGAPKIEKPLQRHGGRLEHN
ETYCGSCYGAEESDDDCCNSCEDVREAYRKKGWALSNPDLIDQCKREGFLQRIKDEEGEGCNVYGFLEVNK
VAGNFHFAPGKSFQQSGVHVHDLLAFQKDSFNLSHHINRLAFGEYFPGVVNPLDNVHWTQETPSGMYQYFI
KVVPTVYTDVSGHTIQSNQFSVTEHFRTGDVGRLQSLPGVFFFYDLSPIKVTFTEENVSFLHFLTNVCAIV
GGIFTVSGILDSFIYHGQRAIKKKMELGKFN

NCBI Reference Sequence: XP_003542443.1
>G.maxERV-A2
MESIISKLRNLDAYPKINEDFYSRTLSGGVITLASSILMLLLFYSELRLYLHAVTETKLVVDTSRAETLRI
NFDVTFPALPCSILSLDAMDISGEQRLDVKHDIIKKRLDSRGNVIETRQEGIGAPKIEKPLQRHGGRLEHN
ETYCGSCYGSEVSDDDCCNSCEDVREAYRKKGWALSNPDLIDQCKREGFLQRIKDEEGEGCNVYGFLEVNK
VAGNFHFAPGKSFQQSGVHVHDLLAFQKDSFNLSHHINRLTFGEYFPGVVNPLDNVHWTQETPSGMYQYFI

KVVPTVYTDVSGHTIQSNQFSVTEHFRTGDMGRLQSLPGVFFFYDLSPIKVTFTEENVSFLHFLTNVCAIV
GGIFTVSGILDSFIYHGQRAIKKKMELGKFN

NCBI Reference Sequence: XP_003524744.1
>G.maxERV-A3
---MDKVFNKLRNLDAYPKVNEDFYNR
TLAGGVVTVVSAAVMLFLFFSELSLYLYTVTESKLLVDTSRGDTLHINFDVTFPAVRCSILSLDAMDISGE
QHLDIRHNIVKKRIDANGNVIEERKDGIGAPKIERPLQKHGGRLGHDEKYCGSCFGAEESDEHCCNSCEEV
REAYRKKGWAMTNMDLIDQCQREGYVQRVKDEEGEGCNLQGSLEVNKVAGNFHFATGKSFLQSAIFLADLL
ALQDNHYNISHRINKLSFGHHFPGLVNPLDGVKWVQGPAHGMYQYFIKVVPTIYTDIRGRVIHSNQYSVTE
HFKSSELGVAVPGVFFFYDISPIKVNFKEEHIPFLHFLTNICAIIGGVFTVAGIIDSSIYYGQRTIKRKME
LGKFT

Phytozome *Glycine max* Wm82.a2.v1 Proteome ID: Glyma.08G073600.1
>G.maxERV-A4
MDKVFNKLRNLDAYPKVNEDFYNRTLAGGVVTVVSAAVMLFLFFSELSLYLYTVTESKLLVDTSRGDTLHI
NFDVTFPAVRCSILSLDAMDISGEQHLDIRHNIVKKRIDANGNVIEERKDGIGAPKIERPLQKHGGRLGHD
EKYCGSCFGAEESDEHCCNSCEEVREAYRKKGWAMTNMDLIDQCQREGYVQRVKDEEGEGCNLQGSLEVNK
VAGNFHFATGKSFLQSAIFLADVLALQDNHYNISHRINKLSFGHHFPGLVNPLDGVRWVQGPTHGMYQYFI
KVVPTIYTDIRGRVIHSNQYSVTEHFKSSELGVAVPGVFFFYDISPIKVNFKEEHTPFLHFLTNICAIIGG
VFTVAGIIDSSIYYGQRTIKRKMELGKFT

NCBI Reference Sequence: XP_003542168.1
>G.maxERV-B1
MGMKQVIKNLDAFPRAEDHLLQKTQSGALVSVIGLIIMATLFVHELGYYLTTYTVHKMSVDLKRGETLPIH
INMTFPSLPCDVLSVDAIDMSGKHEVDLDTNIWKLRLNSYGHIIGTEYISDLVEKEHTNQEHDDNKDHDHH
HEHSEQKIHLQNLDESTENIIKKVKEALKNGEGCRVYGVLDVQRVAGNFHISVHGLNIYVAQMIFDGAKNV
NVSHFIHDLSFGPKYPGLHNPLDDTTRILHDTSGTFKYYIKVVPTEYRYISKEVLPTNQFSVSEYYSPINQ
FDRTWPAVYFLYDLSPITVTIKEERRSFLHFITRLCAVLGGTFAVTGMLDRWMYRLLEALTKSKSKR

NCBI Reference Sequence: XP_006605681.1
>G.maxERV-B2
MGMKQVIKNLDAFPRAEDHLLQKTQSGALVSVIGLIIMATLFVHELGYYLTTYTVHQMSVDLKRGETLPIH
INMTFPSLPCDVLSVDAIDMSGKHEVDLDTNIWKLRLNSYGHIIGTEYISDLVEKEHTHHKHDDNKNHEHS
EQKIHLQNLDESTENIIKKVKEALKNGEGCRVYGVLDVQRVAGNFHISVHGLNIYVAQMIFDGAKNVNVSH
FIHDLSFGPKYPGLHNPLDDTTRILHDTSGTFKYYIKVVPTEYRYISKEVLPTNQFSVSEYYSPINQFDRT
WPAVYFLYDLSPITVTIKEERRSFLHFITRLCAVLGGTFAVTGMLDRWMYRLLETLTKSKSKR

NCBI Reference Sequence: XP_003543298.1
>G.maxPDI-C1
MISSSKIKSVDFYRKIPRDLTEASLSGAGLSIVAALAMIFLFGMELNSYLSVTTSTQVIVDKSSDGDYLRI
DFNISFPALSCEFAAVDVSDVLGTNRLNLTKTVRKFSIDSNLRPTGAEFHSEPAANSIKHDNEVNEESVEG
SVVLKTQNFDKYAHQFPITVVNFYAPWCYWSQRLKPSWEKAAKIIKERYDPEMDGRIILGRVDCTEDGDLC
RSHHIQGYPSIRIFRKGSDVRSNHGHHDHESYYGDRDTDSLVKTMENLVASLPSESQKLPLEDKSDVAKNT
ERPAPSTGGCRIDGYVRVKKVPGNLIFSARSNAHSFDASQMNMSHVINHLSFGRKVSPRVMSDVKRLIPYV
GSSHDRLNGRSFINTHDLGANVTMEHYLQIVKTEVITRKDYKLVEEYEYTAHSSVAQSLHIPVAKFHLELS
PMQVLITENQKSFSHFITNVCAIVGGIFTVAGIMDAILHNTIRLMKKVELGKNF

NCBI Reference Sequence: XP_003540413.1
>G.maxPDI-C2
MISSSKIKSVDFYRKIPRDLTEASLSGAGLSIVAALAMIFLFGMELNSYLSVSTSTQVIVDKSSDGDYLRI
DFNISFPALSCEFAAVDVSDVLGTNRLNLTKTVRKFSIDSNLRPTGAEFHSEPAANSIKHDNEVNEESVEG
SVVLTTQNFDKYAHQFLITVVNFYAPWCYWSQRLKPSWEKTAKIIKERYDPEMDGRIILGRVDCTEDGDLC
RSHHIQGYPSIRIFRKGSDVRSDHGHHDHESYYGDRDTDSLVKTMENLVASLPSESQKLPLEDKSNVATNT
KRPAPSTGGCRIDGYVRVKKVPGNLIISARSNAHSFDASQMNMSHVINHLSFGRKVSLRVMSDVKRLIPYV
GSSHDRLNGRSFINTHDLGANVTIEHYLQIVKTEVITRKEYKLVEEYEYTAHSSVAQSLHIPVAKFHLELS
PMQVLITENQKSFSHFITNVCAIIGGIFTVAGIMDAIFHNTIRLMKKVELGKNF

Phytozome *Glycine max* Wm82.a2.v1 Proteome ID: Glyma.12G134500.1
>G.maxPDI-C3
MISATKLKSVDFYRKIPRDLTEASLSGAGLSIVAALVMMFLFGMELSSYMSVSTSTSVIVDKSSDGDYLRI
DFNISFPALSCEFASVDVSDVLGTNRLNITKTVRKFSIDSNLRPTGAEFHSGTVANAVKHDDEVDEESVEG
SFSLTTHNFDKYVHQFPVTIVNFYAPWCSWCQRLKPSWEKTAKIMKERYDPEMDGRIILAKVDCTQEGDLC
RRNHIQGYPSIRIFRKGSDLRSEHGHHEHESYYGDRDTESLVKFMEDLVTSLPTESQKLALEDKSNAADNA
KRPAPSAGGCRVEGYVRVKKVPGNLIISARSDAHSFDASQMNMSHVINNLSFGKKVTPRAMSDVKLLIPYI
GSSHDRLNGRSFINTRDLGANVTIEHYIQIVKTEVVTRKGYKLIEEYEYTAHSSVAHSLDIPVAKFHLELS
PMQVLITENQRSFSHFITNVCAIIGGVFTVAGILDSILHNTIRMVKKIELGKNF

NCBI Reference Sequence: XP_003527321.1
>G.maxPDI-C4
MISATKLKSVDFYRKIPRDLTEASLSGAGLSIVAALAMMFLFGMELSSYLSVSTSTSVIVDKSSDGDYLRI
DFNISFPALSCEFASVDVSDVLGTNRLNITKTVRKFSIDSNLRPTGAEFHSGTVANAVKHDDEVDEESVEG
SFSLTTHNFDKYVHQFPITAVNFYAPWCSWCQRLKPSWEKTAKIMKERYDPEMDGRIILAKVDCTQEGDLC
RRNHIQGYPSIRIFRKGTDLRSEHGHHEHESYYGDRDTESLVKFMEDLVTSLPTESQKLALEDKSNASDNA
KRPAPSAGGCRVEGYVRVKKVPGNLIISARSDAHSFDASQMNMSHFINNLSFGKKVTPRAMSDVKLLIPYI
GSSHDRLNGRSFTNTHDLGANVTIEHYIQIVKTEVVTRNGYKLIEEYEYTAHSSVAHSVDIPAAKFHLELS
PMQVLITENQRSFSHFITNVCAIIGGVFTVAGILDSILHNTIRMMKKVELGKNF

***Klebsormidium flaccidum* proteome V1.0: kfl00198_0280**
>K.flaccidumERV-A
MGDVLNYLKKLDAYPKINEDFYSRTLSGGLITLVSSIFIVLLFFTELGLFLTTKTVNELSVDTSRGEQLQI
NFDVTYPHLACSILSLDAMDVSGEQHLDVVHNIFKRRLSPEGKPIDVGTKDEIGGPKVQTGEVKVKESEGG
PAYCGSCWGAEENAEDCCNTCEEVREAYRRKGWAFSNAELIEQCSREGFVEKLKEQEGEGCNVYGYLEVNK
VAGNFHFAPGKSFQQAHMHVHDLMPFASQKFNVSHIINKLSFGADYPGVVHPLDNVRRIQEADGGMYQYFI
KVVPTIYTDLSGHVISTNQFSVTEHFREQEVASGRSLPGVFFFYDLSPIKVKFTEQRQSFLHFVTNVCAIV
GGVFTVSGIIDAFVYHGHKVIKKKMELGKLN

***Klebsormidium flaccidum* proteome V1.0: kfl00263_0210**
>K.flaccidumERV-B
MAFKIAALSRIDAYPRAESHLTQRTISGAAISIMGISLMVLLFVNELQFYMTPYTENEMTVDVKGREKLPI
HINITFPSLPCSVLSLDALDMSGKHEVDISNNIWKARLDSQGRPGIWQQVDKLDHADDTAHNEAHAEDTTE
HDFALANARRAAQADANKQVEDIKAALANQEGCRVYGHLDVERVAGNFHISVHGQSYYVLGQVFASASTVN
VSHHIESVSFGPPIPGTTNPLDGYTRILKTEQESGTFKYFIKVVPTEYFPLKGDHVETNQYSVTEYFMPSS
MQPGGLPAVYFLYDLSPIAVKVTERRRNFGHFLTRICAVLGGTFAVTGMIDKWTYKTVQLFTSQRSSGSQ

***Klebsormidium flaccidum* proteome V1.0: kfl00062_0160**
>K.flaccidumPDI-C
MNRVLGRLRSVDFYRRIPRDLTEATLAGGTLSVVASIAMVLLFGMELQAYLTTSATTTVIIDRSRDGDVMR
INFNFSFPALSCEFASVDVSDVLGTHRINLSKTVRKFPIDHAGRITGPEFHHIAHTVREPAHDDPAPDPID
EGAAEMEAKERQLAEGGQPPEGDPPPIKDPGGALPLSEATFDTITKESFPIVMVNFFAPWCPWSQRLEPVW
EASAKAMLKKYDVDVNPQILLAKVDCTVEVALCRKHHVQGFPSIRIFRSGQDVRAAQGHHDHESYYGDRTE
AAITQFADDLATGLQQRAHALPGATAQQVVPPWMGKDGRALRVVAKAPQSSGCQIEGFVLVKKVPGNLQVA
AVSAGHSFDPATMNMSHSVNSFSFGRKPTLKQYHEIDRIWSHHPSAGEDRLTGRYFHSHSDNLTHEHYLQV
VLTSVEPLKLPAHQYVQSYDYTAHSNVFNSAAVPVAKFHYELSPMQVVITENSRSFSHFITNVCAIIGGVF
TVAGIVDGILFQAASAIKKVQLGKQF

NCBI Reference Sequence: XP_003615024.1
>M.truncatulaERV-A1
MDSIMNKLRNLDAYPKINEDFYSRTLSGGLITIVSSILMLLLFFSELRLYLHAATETKLVVDTSRGETLRI
NFDVTFPALACSIVSLDAMDISGEQHLDVRHDIIKKRIDSHGNVIETRQDGIGSPNIEKPLQRHGGRLEHN
ETYCGSCYGAEASDEECCNSCEEVREAYRKKGWALSSPDSIDQCKREGFLERIKEEEGEGCNVYGFLEVNK
VAGNFHFAPGKSFQQSGVHVHDLLAFQKESFNLSHHINRIAFGDYFPGVVNPLDRVHWTQETPSGMYQYFI
KVVPTMYTDVSGNTIQSNQFSVTEHFRTADVGRLQSLPGVFFFYDLSPIKVTFTEEHVSFLHFLTNVCAIV
GGIFTVSGILDSFIYHGQKAIKKKMELGKFS

GenBank: ACJ84247.1
>M.truncatulaERV-A2
MDKVFNKLRNLDAYPKVNEDFYNRTLAGGVVTVVSAAVMLFLFISELRLYLYTVTESKLLVDTSRGETLNI
NFDVTFPAVRCSILSLDTMDISGERHHDILHNIMKQRIDANGKVIEARKEGIGAPKIERPLQKHGGRLEHD
EKYCGSCFGAEESDDHCCNNCEEVREAYRKKGWALTNIDLIDQCQREGFVQKVKDEEGEGCNIHGSLEVNK
VAGNFHFATGQSFLQSAIFLTDLLALQDNHYNISHQINKLSFGHHYPGLVNPLDGIKWVQGNDHGMCQYFI
KVVPTVYTDIRGRVIHSNQYSVTEHFKSSELGAAVPGVFFFYDISPIKVNFKEEHIPFLHFLTNICAIIGG
IFTIAGIVDSSIYYGQKTIKKKMEIGKYR

GenBank: KEH35189.1
>M.truncatulaERV-B
MGMKQVIKNLDAFPRTEDHLLQKTQSGALVSIIGLIIMATLFLHELGYYLTTYTVHQMSVDLKRGETLPIH
INMTFPSLPCDVLSVDAIDMSGKHEVDLDTNIWKLRLNSYGQIIGTEYISDLVEKGHEHDHDHGTHKHDDS
KDHHEHSEQKVHLQTFDEATENTIKKVKEALKNGEGCRVYGVLDVQRVAGNFHISVHGLNIYVAQMIFDAG
KNVNVSHVIHDLSFGPKYPGIHNPLDETSRILHDASGTFKYYIKIVPTEYRYISKEVLPTNQFSVTEYFSP
ITSQFDRTWPAVYFLYDLSPITVTIKEERRSFLHFITRLCAVLGGTFAVTGMLDRWMYRLVEAATKPKNKK

GenBank: AES66908.2
>M.truncatulaPDI-C1
MTTASKIKSVDFYRKIPRDLTEASLSGAGLSIVAALAMMFLFGMELNEYLSVHTSTSVIIDKSSDGEFLRI
DFNLSFHALSCEFASVDVSDVLGTNRMNLTKTVRKFSIDSNLRPTGSEFYLRPSTNVIKHDDKVDDESLEG
AFVFTSNNFDKYSHQFPITAVNFYAPWCYWSQRLRPSWEKTAKIIRERYDPEMDGRILLGKVDCTKEADLC
RRHHIQGYPSIRIFRKGSDVRSDHGHHEHESYYGDRDTDSLVKTMENILASFPSEYYKLALEDKLNVTEDS
KRPAPSSGGCRIEGYVRVKKVPGNLIISARSDAHSFDASQMNMSHAVHHLSFGKKLSPKLMSDVQRLIPYV
GNSHDRLDGLSFINSHDFGANVTLEHYLQIVKTEVITRQGYQLVEEYEYTAHSSLAHSLHVPVARFHLQLS
PMQVLITEDHKSFSHFITNVCAIVGGVFTVAGITESILHNTIRLMRKVELGKNF

NCBI Reference Sequence: XP_003607653.1
>M.truncatulaPDI-C2
MLSASKLKSVDFYRKIPRDLTEASLSGAGLSILAALAMMFLFGMELSNYFAVTTSTSVIVDKSSDGDFLRI
DFNFSFPALSCEFASVDVSDVLGTNRLNITKTVRKFSIDSKLRPTGSEFHSGTIANAVKHDDEVDTDFVEG
SLPLTSQHFDKYVQLFPITVVNFYAPWCSWCQRLKPSWEKAAKIMRERYDPEMDGRILLAKVDCTQEGDLC
RRHHIQGYPSIRIFRKGSDVRSDHGHHEHESYYGDRDTESLVKTMETLVASLPTGSQHLALEDKSNGTKRP
APSTGGCRVEGYVRVKKVPGSLVVSARSDAHSFDASQMNMSHVINHLSFGKKVTPRAMIDVKHWIPYLGIN
HDRLNGRSFINTRDLEGNVTIEHYIQVVKTEVITRKGYKLIEEYEYTAHSSVAHSVNIPVARFHLELSPMQ
VLITENQKSFSHFITNVCAIIGGVFTVAGILDSILHNTIKAMKKIEIGKNF

GenBank: CAE02574.2
>O.sativaERV-A1
MEGLLSKLRSLDAYPKVNEDFYSRTLSGGIITLASSVVMLLLFVSELRLYLHAVTETTLRVDTSRGETLRI
NFDVTFPALQCSIISLDAMDISGQEHLDVKHDIFKQRIDVHGNVIATKQDAVGGMKVEQPLQRHGGRLEHN
ETYCGSCYGAEESDEQCCNSCEDVREAYRKKGWGVSNPDLIDQCKREGFLQSIKDEEGEGCNIYGFLEVNK
VAGNFHFAPGKSFQKANVHVHDLLPFQKDSFNVSHKINKLSFGQRFPGVVNPLDGAQWMQHSSYGMYQYFI
KVVPTVYTDINEHIILSNQFSVTEHFRSSESGRIQAVPGVFFFYDLSPIKVTFTEQHVSFLHFLTNVCAIV
GGVFTVSGIIDSFVYHGQRAIKKKMEIGKFN

GenBank: ABF95668.1
>O.sativaERV-A2
MDLWNKLRSLDAYPKVNEDFYSRTLSGGLITIASSLAILLLFLSEIRLYLYSATDSKLTVDTSRGERLHIN
FDVTFPALPCSLVAVDTMDVSGEQHYDIRHDIIKKRIDNLGNVIESRKDGVGAPKIERPLQKHGGRLDHNE
VYCGSCYGSEESDDQCCNSCEDVRDAYRKKGWALTNIEEIDQCKREGFVQRLKDEQGEGCSIHGFVNVNKV
AGNFHFAPGKSLDQSFNFLQDLLNFQQENYNISHKINKLSFGVEFPGVVNPLDGVEWIQEHTNGLTGMYQY
FVKVVPTIYTDIRGRKINSNQFSVTEHFREAIGYPRPPPGVYFFYEFSPIKVDFTEENTSLLHFLTNICAI
VGGIFTVAGIIDSFVYHGHRAIKKKMEIGKLG

NCBI Reference Sequence: NP_001055898.1

>O.sativaERV-A3
MEGFLQKLKGLDAYPKVNEDFYKRTLSGGVVTVVASVVMLLLFVSETRSYFYSATETKLVVDTSRGERLRV
NFDVTFPSVPCTLLSVDTMDISGEQHHDIRHDIEKRRLDAHGNVIEARKEGIGGAKIESPLQKHGGRLSKG
EEYCGTCYGAEESDEQCCNSCEEVREAYKKKGWALTNPDLIDQCTREDFVERVKTQQGEGCNVHGFLDVSK
VAGNLHFAPGKGFYESNINVPELSALEHGFNITHKINKLSFGTEFPGVVNPLDGAQWTQPASDGTYQYFIK
VVPTIYTDLRGRKIHSNQFSVTEHFRDGNIRPKPQPGVFFFYDFSPIKVIFTEENSSLLHYLTNLCAIVGG
VFTVSGIIDSFIYHGQKALKKKMELGKYR

NCBI Reference Sequence: NP_001051473.1
>O.sativaERV-B
MGRIPSLKNFNAFPHAEDHLLKKTYSGAIVTIFGLIIMVTLFAHELKFYLTTYTVHQMSVDLKRGETLPIH
INMSFPSLPCEVLSVDAIDMSGKHEVDLHTNIWKLRLDKYGHIIGTEYLNDLVEKEHGTHNHDHDHEHEDE
QKKQEHTFNEDAEKMVKSVKQAMENGEGCRVYGVLDVQRVAGNFHISVHGLNIFVAEKIFDGSSHVNVSHI
IHDLSFGPKYPGIHNPLDETTRILHDTSGTFKYYIKIVPTEYRYLSKQVLPTNQFSVTEYFVPKRATDRSA
WPAVYFLYDLSPITVTIKEERRNFLHFLTRLCAVLGGTFAMTGMLDRWMYRLIESVTKSKTRSVLR

NCBI Reference Sequence: NP_001059821.1
>O.sativaPDI-C
MISSSKLKSVDFYRKIPRDLTEASLSGAGLSIVAALAMVFLFGMELSNYLAVNTSTSVIVDRSSDGEFLRI
DFNLSFPALSCEFASVDVSDVLGTNRLNITKTVRKYSIDRNLVPTGSEFHPGPIPTVSKHGDDVEENHDDG
SVPLSSRNFDSYSHQYPVLVVNFYAPWCYWSNRLKPSWEKTAKIMRERYDPEMDGRIILAKVDCTEEIDLC
RRHHIQGYPSIRIFRKGSDLKENQGHHDHESYYGDRDTESLVAAMETYVANIPKDAHVLALEDKSNKTVDP
AKRPAPLTSGCRIEGFVRVKKVPGSVVISARSGSHSFDPSQINVSHYVTQFSFGKRLSAKMFNELKRLTPY
VGGHHDRLAGQSYIVKHGDVNANVTIEHYLQIVKTELVTLRSSKELKLVEEYEYTAHSSLVHSFYVPVVKF
HFEPSPMQVLVTELPKSFSHFITNVCAIIGGVFTVAGILDSIFHNTLRLVKKVELGKNI

NCBI Reference Sequence: XP_001759631.1
>P.patensERV-A1
MALQMIQKLKSLDAYPKINEDFYSRTLSGGIITIISATFMVLLFFSELKLYLAAQVANDLVVDTERGGTIQ
INLDVTFPALACSVVSLDAMDISGEAHLDVKHNIFKKRLDVNGKVIEPARQESINQPKLDKPLQKHGGRLE
HNETYCGSCFGAETEEDHCCNNCEEVREAYRKKGWALNNPDLIDQCKREGFLQKIKDEDGEGCNVYGTLEA
NKVAGNFHFAPGKSFQQANMHVHDLMAFGKDSFNVSHKINEISFGVRYPGAVNPLDKLERIQTTTHGMYQY
FIKVVPTVYTDTRGRKISTNQFAVTDHFKGVGPGEDHALPGVFFFYDLSPIKVKFTEKRMSFFHFLTNVCA
IVGGVFSVSGIIDAFVYHGQKQIKKRLGKDT

NCBI Reference Sequence: XP_001764962.1
>P.patensERV-A2
MAVFNKLKQLDAYPKISEDFYSRTLSGGVITLVSTVFMFVLFVTEISLYLSAQTQNQLVVDTSRGETLQIN
LDITFPALACSMVSLDAMDISGEQHLNVRHNIFKKRLDVHGKVVNAPKPDAINAPKVQKPLQKHGGRLEHN
ETYCGSCFGAESSDDECCNNCEEVREAYRKKGWALTNADLIDQCHREGFIERVKEEAGEGCNIYGKLEVNK
VAGNFHFAPGKSFQQSAMHLLDLMGFITDSFNVSHTINELSFGAHFPGAVNPLDKVTNIQKDLNGMYQYFI
KVVPTVYTDIKGRKISTNQFSVTEHYTAGDHGPRFVPGVFFFYDLSPIKVKFSEERPSFLHFLTNVCAIVG
GVYSIAGIIDSFVYHGHRAIKKKMELGKLS

NCBI Reference Sequence: XP_001754958.1
>P.patensERV-A3
MAIFNKLKQLDAHPKISEDFYSRTLSGGVITLVSSIFMFLLFVTEFRIYLSAQTQNQLVVDTSRGETLQIN
LDITFPALACSVVSLDAMDISGELHLDVRHNIYKKRLDVHGKAVDAPKPDAINAPKVQKPLQKHGGRLEDH
ETYCGSCFGAESSDDQCCNSCEEVREAYRKKGWALTNTDLIDQCHREGFIERIKEEAGEGCNIYGKLEVNK
VAGNFQIAPGKSFQQSAMHLLDLMGFVTDSFNVSHTINELSFGAYFPGAVNPLDKVTSIQKDQNGMFQYFI
KVVPTVYTDIKGRKISTNQFSVMEHYTAGDHGPRVIPGVFFFYDLTPIKVKFTEERPSFLHFLTNVCAIIG
GIYTIAGIVDSFIYHGHRAIKKKMELGKLS

NCBI Reference Sequence: XP_001762360.1
>P.patensERV-A4
MSFFNKLKHLDAYPKISEDFYSRTLSGGLITLVSSVFMTLLFITEFRIYLSAQTQNQLVVDTSRGETLQIN
LDITFSALACSVVSLDAMDISGEQHLNVRHNIFKKRLDVHGKAIDAPKPDAINAPKVQRPLQKHGGRLEHN

ETYCGSCFGAASSDDECCNSCEEVREAYRKKGWALINIDIIDQCHREGFIERVKEEAGEGCNIYGKLEVNK
VAGNFHIAPGKLFQQSAMHLLDLLGIRSDSFNVSHIVNELSFGAHFPGRVNPLDKITSIQKDQNGMYQYFI
KVVPTVYTDIRGSEIATNQFSVTEHYTAGDHGPRVVPGVFFFYDLSPIKVKFTEKRPSFLHFLTTVCAIVG
GVYTVASIIDSFIYHGHRAVKKKMELGKFS

NCBI Reference Sequence: XP_001754824.1
>P.patensERV-B
MRKEKWQVIKNLDAFPRAEEHLLQKTSSGAAVSAIGLFIMGVLFFHELRFYLETVTVHEMSVDVKRGEKLP
IHINMTFPALPCEVLSLDAIDMSGKHEVDLDTNIWKLRIHRDGYVLGSEFVNDLVEGEHRKEEPKADKKDE
HKDGDHRKKDPQKVINEVKKAIDDGEGCQIFGVLDVERVAGNFHISMHGLSLYVASKIFEAGYEVNVSHVI
HDLSFGPTYPGHHNPLDGSERILHDTSGTFKYFLKIVPTEYHYLHGEVMPTNQFSVTEYYQRTKPSDRSYP
AVYFVYDLSPIVVTIREHRRNFGHFITRLCAVLGGTFAVTGMLDRWMSRIIDFVMSTSKQGFL

Phytozome *Physcomitrella patens* v3.0 proteome: Phpat.014G071500.2
>P.patensPDI-C
MVSTSKLKSIDFYRKIPRDLTEASLSGAGLSIIAALTMVFLFGMELSAYLSTTTSTSVVVDRSRDGEYLRI
DFNLSFPALSCEFASVDVSDVLGTHRFNLTKTVRKYPIDPLLQRIGQEFHAGSVPNIKSHGDEDVGEDMFE
HLGEGAVELNKNTFDVYAQQFSVLVVNFYAPWCPWSNKLKASWEKAAKIIADKYNPEMDGRILLAKVDCTV
NVELCRSHHIQGYPSIRIFRKGHDVRDEHGRHDHESYYGERDTESLVAFMVELVPPATVDGKFQLEDKSSI
TVNATIKRPAPKAGGCRVEGFVRVKKVPGELMISAHSGSHSFDATSMNMTHYVGFFSFGRKTSWRSVHWVN
EMLPALDSNIDRLTGQVFPSEYENITHDHYLQVVKTEVITLHRKQDLRVLEQYDYTAHSNMIQSTKVPVVK
FHYELSPMQVLVKENPKSFSHFLTNLCAIIGGVFTVAGIIDSMLHNAMHIMKKVELGKQY

NCBI Reference Sequence: XP_007227487.1
>P.persicaERV-A1
MENMMSRLRNLDAYPKINEDFYSRTLSGGVITLASSIVMLLLFLSELRLYLHAVTETKLVVDTSRGETLRI
NFDVTFSALPCSILSLDAMDISGEQHLDVKHDIIKKRLDSHGNVIESRPDGIGAPKIEKPLQRHGGRLEHN
ETYCGSCYGAEAADEDCCNSCEDVREAYRKKGWAISNPDVIDQCKREGFLQRIKDEEGEGCNIYGFLEVNK
VAGNFHFAPGKSFQQSNVHVHDLLAFQKDSFNISHTINRLSFGDYFPGVVNPLDGVHWAQATPSGMYQYFI
KVVPTVYTDVSGHTIQSNQFSVTEHFRGTEVGNLQYLPGVFFFYDLSPIKVTFTEEHISFLHFLTNVCAIV
GGVFTVSGILDSFIYHGQKAIKKKMEIGKFS

NCBI Reference Sequence: XP_007200362.1
>P.persicaERV-A2
MDSILQKLRNLDAYPKINDDFFSRTLSGGIITLASSLLILLLFFSELRLYLHTVTESKLLVDASRGETLHI
NFDVTFPAIRCSLLSLDTMDISGEQHFDIRHDIVKKRIDAHGNVIEAKKDGIGAPKIDNPLQRHGGRLEHN
EKYCGSCFGAEMSDDDCCNSCDEVRDAYRKKGWALTNADMIDQCKREGFIQKIKDEDGEGCNIEGSLEVNK
VAGNFHFVSGKSFHQSNIHVQDLLAFQTDSYNISHKINRLAFGDYFPGKLNPLDGVQWRQETPNGMYQYFI
KVVPTLYTNIRGRTIRSNQYSVTEHFKSSELGHSQLLPGVFFFYDLSPIKVTLKEEHVPFLHFMTHICAIV
GGIFTVAGLIDSFVYHGQRAMKKKMEIGKFG

NCBI Reference Sequence: XP_007215619.1
>P.persicaERV-B
MGVKQALKSLDAFPRAEEHLLQKTQTGAVVSVVGLLIMATLFVHELRYYLTTYTVHQMSVDLKRGETLPIH
INITFPSLPCDVLSVDAIDMSGKHEVDLDTNIWKLRLNSYGHIIGTEYLSDLVEREHSHKHDDSKDHHEDK
DQEIHLQGAFDQAAEDLIKRVKHAIANGEGCQVFGVLDVQRVAGNFHISVHGLNIFVAQMIFEGSKNVNIS
HIIHDLSFGPKYPGIHNPLDGTERILHDTSGTFKYYIKVVPTEYRYISKEVLPTNQFSVTEYFSPMKQFDR
TWPAVYFLYDLSPITVTIKEERRSFLHFITRLCAVLGGTFALTGMLDRWMYRFLEAVTKPNARSVLR

NCBI Reference Sequence: XP_007213964.1
>P.persicaPDI-C
MISTGKIKSVDFYRKIPRDLTEASLSGAGLSIIAALAMMFLFGMELNSYLAFSTSTSVIVDKSSDGDFLRI
EFNISFPALSCEFASIDVSDVLGTNRLNITKTIRKFSIGPDLKPTGSEFHSGPAFHDIKHGDGDEYGGDGS
VSITARTFEKFTHQHPILVVNFYAPWCYWSNRLKPSWEKAAKIIRERYDPEIDGRILLAKVDCTEEGDLCK
RNHIQGYPSIRIFRKGSDVRDDHGHHDHESYYGDRDTDSLVKTMETLVAPIPVEAQKLALEGKSDNGTDNA
KRPAPLTGGCRIEGYVRVKKVPGNLVISAHSGAHSFDASQMNMSHVISHFSFGRMIAPKVMSDVKRLVPYL

GGSHDRLNGRSFINHRDLGANVTIEHYLQIVKSEVITGRNHKLIEEYEYTAHSSLVQSLQIPVAKFHFELS
PMQVLITENQKSFSHFITNVCAIIGGVFTVAGIVDSILHNTIRMMRKVELGKNF

NCBI Reference Sequence: XP_002306582.1
>P.trichocarpaERV-A1
MEGLMSKLRNLDAYPKINEDFYSRTLSGGVITLASSVVMFLLFFSELRLYLHAVTETKLVVDTSRGETLRI
NFDVTFPALPCSILSLDAMDISGEQHLDVKHDIIKKRLDFHGNVIEARQDGIGAPKIEKPLQRHGGRLEHN
ETYCGSCYGAEASDEDCCNSCEDVREAYRKKGWAVTNPDLMDQCKREGFLQKIKDEEGEGCNIYGFLEVNK
VAGNFHFAPGKSFQQSGVHVHDLLAFQKDSFNITHKINRLTFGEYFPGVVNPLDGVQWTQETPSGMYQYFI
KVVPTVYTDVSGHTIQSNQFSVTEHFRGTDIGRLQSLPGVFFFYDLSPIKVTFTEEHVSFLHFLTNVCAIV
GGVFTVSGILDTFIYHGQKAIKKKMEIGKFS

NCBI Reference Sequence: XP_002302286.1
>P.trichocarpaERV-A2
MDGLMSKLRNFDAYPKINEDFYSRTLSGGVITLASSIVMFLLFFSELRLYLHAVTETKLVVDTSRGETLRI
NFDVTFPALPCSILSLDAMDISGEQHLDVKHDIIKKRLDSHGNVIESRQDGIGAPKIEKPLQRHGGRLEHN
ETYCDEDCCNSCEEVREAYQKKGWAVTNPDLMDQCKREGFLQRIKDEEGEGCNIYGFLEVNKVAGNFHFAP
GKSFQQSGVHVHDLLAFQKDSFNTSHKINRLAFGEYFPGVVNPLDGVQWTQETPSGMYQYFIKVVPTVYTD
VSGHTIQSNQFSVTEHFRGADIGRLQSLPGVFFFYDLSPIKVTFTEEHVSFLHFLTNVCAIVGGVFTVSGI
LDSFIYHGQKAIKKKMEIGKFS

Phytozome *Populus trichocarpa* v3.0 Proteome: Potri.001G206100.1
>P.trichocarpaERV-A3
MEGIYQKLRNLDAYPKINEDFYSRTLSGGLITLISSIIMLFLFFSEFSLYLHAVTETKLLVDTTRGQTLRI
NFDITFPAIRCSLLSVDAIDISGEQHHDIRHDITKKRINAHGDVIEVRQDGIGAPKIDKPLQKHGGRLEHN
EEYCGSCFGAEMSDDHCCNSCDEVREAYRKKGWALTNMDLIDQCIREGFVQMIKDEEGEGCNINGSLEVNR
VAGNFHFVPGKSFHQSNFQLLDLLDMQKESYNISHRINRLAFGDYFPGVVNPLDGIQLMHETQNGVQQFFI
KVVPTIYTDIRGRTVHSNQYSVTEHFTKSELMRLDSLPGVYFIYDFSPIKVTFKEEHTSFLHFMTSICAII
GGIFTIAGIVDSFIYHGRRAIKKKMEIGKFS

Phytozome *Populus trichocarpa* v3.0 Proteome: Potri.003G023500.1
>P.trichocarpaERV-A4
MDRIYQKVRNLDAYPKINEDFYSRTLSGGLITLISSVLILFLFFSELSLYLHKVTETKLLVDTSRGQSLRI
NFDVTFPAIRCSLLSVDAIDISGEQHLDIRHDISKKRINAHGDVIEVRQEGIGAPKIDRPLQSHGGRLGHN
EEYCGSCFGGEMSHDDCCNTCEEVREAYRRKGWAMTNMDLIDQCKREGFIQMIKDEEGEGCNINGSLEVNR
VAGSFHFAPWKSFHLSNFLIQDLLDLQKDSYNISHRINRLAFGDYFPGVVNPLAGIQLMHDTPNGVQQFFI
KVVPTIYTDIRGRTVHSNQYSATEHFKKSELTPLDSLPGVYFFYDFSPIKVIFKEEHISFLHFMTSICAII
GGIFTIAGIIDSFIYYGQRAITKKVGIGKFG

NCBI Reference Sequence: XP_002307923.1
>P.trichocarpaERV-B1
MGMKQAIKKLDAFPRAEEHLLQKTQSGALVSIIGLVTMATLFYHELAYYLTTYTVHQMSVDLTRGETLPIH
INITFPSLPCDVLSVDAIDMSGKHEVDLDTSIWKLRLNSYGHITGTEYLSDLVEKEHEAHNHDHNKDHHED
SHAKQHTHGFDDAAETMVKKVKQALANGEGCRVYGVLDVQRVAGNFHISVHGLNIFVAQMIFDGAKHVNVS
HIIHDLSFGPKYPGIHNPLDGTTRILHETSGTFKYYIKIVPTEYRYISKEVLPTNQFSVTEYFSPMTDFDR
TWPAVYFLYDLSPITVTIKEERRSFLHFITRLCAVLGGTFALTGMLDRWMCRLLEALTKPNPRSVLR

Phytozome *Populus trichocarpa* v3.0 Proteome: Potri.016G022800.3
>P.trichocarpaERV-B2
MGVKQAIKSLDAFPRAEEHLLQKTQSGALVSVIGLVIMATLFYHELAYYLTTYTVHQMSVDLQRGEILPIH
VNITFPSLPCDVLSVDAIDMSGKHEVDLDTNIWKLRLNSHGHITGTEYLSDLVEKEHEAHNHDHDKDHHKD
SHEEQHTHGFDDAAETMIKKVKQALANGEGCRVYGVLDVQRVAGNFHISVHGLNIFVAQMIFDGAKHVNVS
HIIHDLSFGPKYPGIHNPLDGTARILRETSGIFKYYIKIVPTEYRYISKDVLPTNQFSVTEYFSPITDFDR
TWPAVYFLYDLSPITVTIKEERRSFLHFITRLCAILGGTFALTGMLDRWMYRLLEALTKPNRGSGFL

NCBI Reference Sequence: XP_002317580.1
>P.trichocarpaPDI-C1

MVSTNKLKSVDFYRKIPRDLTEASLSGAGLSIVAALAMMFLFGMELNNYLTVNTSTTVIVDNSSDGEFLRI
DFNISFPSLSCEFASVDVSDVLGTNRLNITKTIRKFSIDHDLKPTGSEFHSGPVLHQIKHGDEVDEEGGEG
SVSLKAHNFDQYSHQYPILVVNFFAPWCYWSNRLKPSWEKAAKIIERYDPEMDGRILLAKVDCTEEGDLC
RRNHIQGYPSIRIFRKGSNLREDHGRHDHESYYGDRDTESLVKTMEALVAPIAMESQRQALEHKPENATQH
VKRPAPSAGGCRIEGYVRVKKVPGNLMISALSGAHSFDSKQMNLSHVISHFSFGMKVLPRVMSDVKRLLPY
IGRSHDKLNGRSFINHRDVGANVTIEHYLQVVKTEVVTRRSSSERKLIEEYEYTAHSSLSQTVYMPTAKFH
FELSPMQVLITENSKSFSHFITNVCAIIGGVFTVAGILDSILHHTVRMMKKVELGKNF

NCBI Reference Sequence: XP_006370647.1
>P.trichocarpaPDI-C2
MVSTNKLKSVDFYRKIPRDLTEASLSGAGLSIVAALAMVFLFGMELNNYLTVNTSTSVIVDNSSDGEFLRI
DFNLSFPSLSCEFASVDVSDVLGTNRLNITKTIRKFSIDHDLKPTGSEFHSGPVLHHINHGDEVHEEGSEG
SVSLKAHNFDQYTHQYPILVVNFYAPWCYWSNRLKPSWEKAAKIIERYDPEIDGRILLAKVDCTEEGDLC
RRNHIQGYPSIRIFRKGSDLRDDHGHHDHESYYGDRDTDSLVKTMEGLVAPIAMESQRHALEHKPENATEH
VKRPAPSAGGCRIEGYVRVKKVPGNLVISARSGAHSFDSAQMNLSHVISHFSFGMKVLPRVMSDVKRLIPH
IGRSHDKLNGRSFINHRDVGANVTIEHYLQVVKTEVVTRRSSAEHKLIEEYEYTAHSSLAQTVYMPTAKFH
FELSPMQVLITENPKSFSHFITNVCAIIGGVFTVAGILDSILHNTFRMMKKVELGKNF

NCBI Reference Sequence: XP_007141162.1
>P.vulgarisERV-A1
MEGIMSKLRNLDAYPKINEDFYSRTLSGGVITLASSIIMLLLFFSELRLYLQSVTETKLVVDTSRGETLRI
NFDVTFPALPCSILSLDAMDISGEQHLDVKHDIIKKRLDSHGNVIETRQEGIGAPKIEKPLQRHGGRLEHN
ETYCGSCFGAEASDDDCCNSCEDVREAYRKKGWALSNPDLIDQCKREGFLQRIKDEDGEGCNVYGFLEVNK
VAGNFHFAPGKSFQQSGVHVHDLLAFQKDSFNLSHHINRLSYGEYFPGVVNPLDNVHWIQETPSGMYQYFI
KVVPTVYTDVNGHAIQSNQFSVTEHFRTGDVGRLQSLPGVFFFYDLSPIKVTFTEEHVSFLHFLTNVCAIV
GGIFTVSGILDSFIYHGQRAIKKKMELGKFN

NCBI Reference Sequence: XP_007159091.1
>P.vulgarisERV-A2
MDKVFNKLRNLDAYPKVNEDFYSRTLAGGVVTVVSAAVMLFLFFSELNLYLYTVTESTLLVDTSRGDTLHI
NFDVTFPAVRCSILSLDAMDISGEQHLDIRHNIVKKRIDANGNVIEERKDGIGAPKIERPLQKHGGRLGHD
EKYCGSCFGAEETDEQCCNSCEEVREAYRKKGWALSNMDLIDQCQREGYVQRVKDEEGEGCNIQGSLEVNK
VAGNFHFATGKSFLQSAIFLADLLALQDNHYNISHRINKLSFGHHFPGLVNPLDGVKWVQGPTHGMYQYFI
KVVPTIYTDIRGRVIHSNQYSVTEHFKSSELGVAVPGVFFFYDISPIKVNFKEEHIPFLHFLTNVCAIIGG
IFTVAGIIDSSIYYGQRTIKKKMEIGKYT

NCBI Reference Sequence: XP_007145973.1
>P.vulgarisERV-B
MGMKQVIKNLDAFPRAEDHLLQKTQSGALVSVIGLIIMATLFVHELRYYLTTYTVHQMSVDLKRGETLPIH
INMTFPSLPCDVLSVDAIDMSGKHEVDLDTNIWKLRLNSDGHIIGTEYISDLVEKGHTHHKHDDHKDHDEH
SEQKIHLQNLDESTENIIKKVKEAIKNQEGCRVYGVLDVQRVAGNFHISVHGLNIYVAQMIFDGAKNVNVS
HVIHDLSFGPKYPGIHNPLDDTNRILHDTSGTFKYYIKVVPTEYRYISKEVLPTNQFSVSEYYSPINQFDR
TWPAVYFLYDLSPITVTIKEERRSFLHFITRLCAVLGGTFAVTGMLDRWMYRLIEALTKSKSKR

NCBI Reference Sequence: XP_007149711.1
>P.vulgarisPDI-C1
MISSSKLKSVDFYRKIPRDLTEASLSGAGLSIVAALCMVFLFGMELNSYLSVSTATSVIVDKSSDGDYLRI
DFNMSFPALSCEFAAVDVSDVLGTNRLNLTKTIRKFSIDSNLRSTGNEFHSEPTTTNIKHDNEQHEESIGG
ALELTTDNFDKYAHQFPITVINFYAPWCYWSQRLRPSWEKASKIIKERYDPETDGRIVMGRVDCTQNGELC
RSHHVQGYPSIRIFRKGSDVRTDHGHHDHESYYGDRDTDSLVKTMDNLVASLPTESQKSHSGDKSNLASHI
KRPAPSSGGCRIEGYVRVKKVPGNLIISARSDAHSFDASQMNMSHVVNHLSFGRKVSPRVMSDVKRLIPYV
GSSHDRLNGLSFINTRDFGANVTMEHYLQIVKTEVITRKDHKLVEEYEYTAHSSVAQSLHIPVAKFHLELS
PMQVLITENPKSFSHFITNVCAIIGGVFTVAGILDSILHNTIRLMKKVELGKNF

Phytozome *Phaseolus vulgaris* v1.0 proteome: Phvul.011G139400.1
>P.vulgarisPDI-C2

MISATKLKSVDFYRKIPRDLTEASLSGAGLSIVAALSMMFLFGMELSNYLTVSTSTSVIVDKSSDHDYLRI
DFNISFPALSCEFASVDVSDVLGTNRLNITKTVRKFSIDSHLRPTGAEFHSGAVSNAVKHDDEVDGESVEG
SFSFTTHNFDKYAHQFPITVVNFYAPWCSWCQRLKPSWEKTAKIMKERYDPEMDGRIIMGKVDCTLEGDLC
RRNHIQGYPSIRIFRKGSDLRSEHGHHEHESYYGDRDTESLVEFMENLVTSLPTASQKPALEDKSNATDNA
KRPGPSAGGCRIEGYVRVKKVPGNLIISARSDAHSFDASQMNMSHVVNNLSFGKKVTPRAMSDVKLLIPYL
GSSHDRLSGRSFSNTRDFGANVTIEHYIQIVKTEVVNRKGYKLIEEYEYTAHSSVAHSVDIPVAKFHIELS
PMQVLITENQRSFSHFITNVCAIIGGIFTVAGILDSILHNTIRMMKKVELGKNF

NCBI Reference Sequence: XP_002447951.1
>S.bicolorERV-A1
MDGLLSKLRSLDAYPKVNEDFYSRTLSGGVITLASSVIMLLLFVSELRLYLHAVTETTLRVDTSRGETLRI
NFDVTFPALQCSIISLDAMDISGQEHLDVKHDVFKQRIDAHGNVIATRQDAVGGMKMEAPLQHHGGRLEHN
ETYCGSCYGAQESDGQCCNSCEDVREAYRKKGWGVSNPDLLDQCKREGFLQSIKDEEGEGCNIYGFIEVNK
VAGNFHFAPGKSFQQSNVHVHDLLPFQKDSFNVSHKINRLSFGEYFPGVVNPLDGASWVQHSSYGMYQYFI
KVVPTVYTDINEHIILSNQFSVTEHFRSGESGRMQALPGVFFFYDLSPIKVTFTEQHVSFLHFLTNVCAIV
GGVFTVSGIIDSFVYHSQRAIKKKMEIGKFN

NCBI Reference Sequence: XP_002465347.1
>S.bicolorERV-A2
MELWSKLRNLDAYPKVNEDFYSRTLSGGLITILSSLAILLLFFSEIRLYLYSATESKLTVDTSRGERLHIN
FDVTFPALPCSLVAVDTMDVSGEQHYDIRHDITKKRIDHLGNVIESRKDRVGAPKIERPLQKHGGRLDHNE
VYCGSCYGAEETDDQCCNSCEEVRDVYRKKGWAINNVELIDQCKREGYVQRLKDETGEGCTIHGFVNVNKV
AGNFHFAPGKSLDQSFNFLQDLLNIQPETYNISHKINKLSFGEEFPGVVNPLDGVEWIQDNSNGLTGMYQY
FVKVVPTIYTDIRGRKIYSNQFSVTEHFREAIGYPRPPPGVYFFYEFSPIKVDFTEENTSLLHFLTNICAI
VGGIFTVAGIIDSFVYHGHRAIKKKMELGKLG

NCBI Reference Sequence: XP_002439992.1
>S.bicolorERV-A3
MDAFLQRLKRLDAYPKVNEDFYKRTLSGGIVTLVAAVVMLLLFISETRSYFYSATETKLVVDTSRGERLRV
NFDITFPSIPCTLLSVDTMDISGEQHHDIRHDIEKRRLDSHGNVIEARKEGIGGAKIERPLQKHGGRLDKG
EQYCGTCYGAEESDEQCCNSCEEVREAYKKKGWALTNPDLIDQCAREDFVERVKTQQDEGCNVHGFLDVSK
VAGNFHFAPGKGFYESNIDVPELSVLEGGFNITHKINKLSFGTEFPGVVNPLDGAQWIQPASDGTYQYFIK
VVPTIYTDIRGHNIHSNQFSVTEHFRDGNILPKPQPGVFFFYDFSPIKVIFTEENRSLLHYLTNLCAIVGG
VFTVSGIIDSFIYHGQKALKKKMELGKYR

NCBI Reference Sequence: XP_002458688.1
>S.bicolorERV-B
MARIPSLKSLNAFPHAEEHLLKKTYSGAVVTILGLLVMITLFVHELQFYLTTYTVHQMSVDLKRGETLPIH
INMSFPSLPCEVLSVDAIDMSGKHEVDLHTNIWKLRLDKYGHIIGTEYLSDLVEKGHGAHHDHDHGQEHHD
EQKKPEQTFNEEAEKMIKSVKQALGNGEGCRVYGMLDVQRVAGNFHISVHGLNIFVAEKIFEGSSHVNVSH
VIHELSFGPKYPGIHNPLDETSRILHDTSGTFKYYIKVVPTEYKYLSKKVLPTNQFSVTEYFLPIRPSDRA
WPAVYFLYDLSPITVTIKEERRNFLHFITRLCAVLGGTFAMTGMLDRWMYRLIESVTNSKTRSVLR

Phytozome *Sorghum bicolor* v2.1 proteome: Sobic.002G322400.1
>S.bicolorPDI-C
MISSSKLKSVDFYRKIPRDLTEASLSGAGLSIVAALAMVFLFGMELSNYLAVNTTTSVIVDRSSDGEFLRI
DFNISFPALSCEFVSVDVSDVLGTNRLNIRKTVRKYSIDRNFVPTGSEFHPGPIPTVNKHGDDVEEDHVDG
AFSLSSRNFDSFSHQYPVLVVNFYAPWCYWSNRLKPSWEKTAKIIRERYDPEMDGRILLGKVDCTEEVDLC
RRHHIQGYPSIRVFRKGSDIKENQGHHDHESYYGERDTESLVAAMETYVANIPKEAHVLALEDKSNKTADP
AKRPAPMTSGCRIEGFVRVKRVPGSVIVAARSGSHSFDPSQINVSHYVTQFSFGKRLSHRMLDEFTRLTPY
LSGYNDRLAGQSYIVKHGEVNANVTIEHYLQVVKTEIVTQRSSKELKVLEEYEYTAHSSLVHSFYVPVVKF
HVEPSPMQVLVTEVPRSFSHFITNVCAIIGGVFTVAGILDSIFHNTLRMVKKVELGKNI

NCBI Reference Sequence: XP_004237893.1
>S.lycopersicumERV-A1
MDSFVSKIRSLDAYPKINEDFYSRTLSGGVITLASSIIMTLLFISELRLYLHAATETKLVVDTSRGETLRI
NFDITFPAIPCSILSVDAMDISGEQHLDIRHDIIKKRIDVLGNVIETRKEGIGSPTIDRPLQRHGGRLEHN

ETYCGSCYGAEGSDDHCCNTCEDVREAYRKKGWALTNPDEIDQCKREGFLEKIKEEEGEGCNMYGFLEVNK
VAGNFHFAPGKSFQQSNVHVHDLLTFQKDSYNISHKINRLTYGEYFPGVVNPLDGVKWTQETPHGMYQYFI
KVVPTVYTDVSGHTIQTNQFSVTEHFKGADFGRFQSIPGVFFFYDLSPIKVTFTEQHASFLHFLTNVCAIV
GGVFTVSGILDSFIYHGQKAIKKKMELGKFS

NCBI Reference Sequence: XP_004230558.1
>S.lycopersicumERV-A2
MERVFSKLRNLDAYPKINEDFYNRTLSGGIITLVASVIMLVLFVNELGLYIHSYTETQLVVDTSRGGKLHI
HFDITFPAVPCSLLSLDARDISGEEHFDIRHDIFKKRIDSHGAVIEVRQDGIGAPKIEKPLQRHGGRLEHN
ETYCGSCFGAETADDECCNSCEEVREAYRKRGWGMTNPDLIDQCKREGFVQKIKDEEGEGCNIHGSLEVNK
VAGNFHFAAGKSFHQSTFQLFELISLQSDTYNISHRVNKLAFGDSIPGVVNPLDGVQWTQEAQNGMYQYFI
KVVPTIFKGVRGRTIDSNQFSVTEHFKGSDLGLFQSITGVYFFYDLSPIKVTFTEEHVSFFHFLTNVCAII
GGVFTVAGILDACIYHSQKAIKKKVELGKFG

NCBI Reference Sequence: XP_004243621.1
>S.lycopersicumERV-B1
MGVKQVLRAMDAFPRAEEHLLQKTKSGAFVSIVGLVIMSTLFLHELSYYLNTITVHQMAVDLRRGETLPIH
INMTFPSLPCDVLSVDAIDMSGKHEVDLDTNIWKLRLNSDGHITGTEYLSDLVEKEHKHDDHKDHHDDSDN
KTHMQGFDQEAENLIKKVKQALAHGEGCRVYGVLDVQRVAGNFHISVHGLNIFVAQMIFGGTTHVNVSHII
HDLSFGPKYPGGHNPLDGTERILRGASGTFKYYIKIVPTEYRYLSKEVLPTNQFSVSEYFSPINEFDRTWP
AVYFLYDLSPITVTIREERRNFLHFITRLCAVLGGTFALTGMLDKWMFRFLEAVMKKNSRSLVR

NCBI Reference Sequence: XP_010313686.1
>S.lycopersicumERV-B2
MGVKQALRAIDAFPRAEEHLLLKTKFGAFVSIVGLLIMVTLFLHELSYYLNIYTVHQMSVDSRRGENLPIH
INMTFPSLPCDVLSVDAIDMSGKHEVDLDTNIWKLRLNSDGHITGTEYLSDLVEKEREAHKHDVHKEHHED
SDKIHLQGIDEESQNMIKKVKQALADGEGCRVYGVLDVQRVAGNFHLSVHGLNIFVAQMIFEKSTHVNVSH
IIHDLSFGPKYPGIHNPLDGTSRILRGTSGTFKYYIKVVPTEYRYISKEVLPTNQFSVTEYFSPIHDFERT
WPAVYFLYDLSPITVTIREERRSFLHFITRLCAVLGGTFALTGMLDGWMYKILESVTKKNSRTIVR

NCBI Reference Sequence: XP_004244141.1
>S.lycopersicumPDI-C
MVSTSKIKSVDFYRKIPRDLTEASLSGAGLSIVAALCMMFLFGMELNNYLTVSTTTSVIVDKSSDAEFLRI
DFNMSFPALSCEFASVDVSDVLGTNRLNITKTVRKHSIDKNLRPTGSEFHSGSTATELKHDAEDDEEYGEG
SVSLNGHSFDRVTHHFPILVVNFFAPWCYWSNRLKPSWEKAANIIRERYDRESDGRILVAKVDCTEEVDLC
RRNHIQGYPSIRIFRKGTDVRDDHGHHDHESYYGDRDTDSLVKMMEDLVAPIKLDSQTITSDNSSTKLETG
LKRPAPVTGGCRIEGFVRVKKVPGNLVISARSAAHSFDASQMNMSHVISSFSFGKTITPKVMSDIKILLPH
LGRSHDRLNGNSYVTNPRDSTENVTIEHFLQVVKTEVMTRSYKLVEEYEYTAHSSLVHSLHIPVAKFHYEP
SPMQVLITENPKSFSHFLTNVCAIIGGVFTVAGILDSILHNTMRMVKKVELGKNF

NCBI Reference Sequence: XP_002977139.1
>S.moellendorffiiERV-A
MQMLKKLQQLDAYPKINEDFHSRTLSGGVITVVSSIFMAILFITELKLFLLPGTTSELLVDTSRGETLQIN
FDITFPALACSVISLDAMDVSGEQHLDVKHNIFKKRLDPSGKVVQPPVQEDIGGPKIDKPLQKHGGRLEHN
ETYCGSCFGAEQSDDECCNSCEEVREAYRKRGWAIHNADLIDQCKREGWLTKIKEEEGEGCNIYGSLEVNK
VAGNFHFAPGKSFSQQHVHVHDVQSLHKEKFNVSHYINELSFGARFPGVVNPLDKEKRIQKFPSAMYQYFI
KVVPTAYTDMTGHKIVTNQFSVTDHFKAVEGLNGRSLPGVFFFYELSPIKVLFTERKTSFLHFLTNVCAII
GGVFTVSGIIDSFIYHGHRAIKKKMEIGKYI

NCBI Reference Sequence: XP_002993277.1
>S.moellendorffiiERV-B
MGLKMKNINAFAHADEHLTQKTVSGAILTIVGVSIILVLFAYEFKFYLSTNVVHQMSVDTTRGQNLPIHIN
ITFPSLPCQILSVDAIDMSGKHEVDLDTNIWKLRLHKDGHILGSEYLSDLVEKEHAHDNLTGIFHSHEELR
SAVKVVNEINKALQDGEGCRVFGVLDVERVAGNFHISMHGMSLQVARQIFHSVKEVNVSHIINDLSFGPKY
PGIHNPLDRTVRILRDTAGTFKYFIKIVPTEYRYLNGGKLPTNQFSVGEYYLAARDDDISWPAVYFLYDLS
PITVLIKEERRSFGHLLTRFCAIVGGTFSLTGMLDRWIYRLVESITRAKGVLI

NCBI Reference Sequence: XP_002982011.1
>S.moellendorffiiPDI-C
MTTASKIKSIDFYRKIPRDLTEASLSGAGLSLIAAFAMIFLFGMELNNYLTVSSTTNVVVDRSKDGEYLRI
QFNMSFPALSCEFASVDVSDALGTNRYNLTKTVRKYPIDPNLKIVGPEFHPGPIPNPTSHGDDDHEGEGAH
VLTSSTFDEYARRYSVLVVNFYAPWCIWSARLKPSWDKAAGIIAEKYHPDTGRILLGKVDCTDNNDLCRKH
HIQGFPSIRIFHKGHDLKDEHGHHEHDSYYGERDTDSLVKAMEALVPKETTLALEDKTNGTVKRPAPRAGG
CRIEGFIRAKK-VPGNIIISAHSGSHSFDASAMNMTHYVSQFTFGRELNFWMRRELYRIYPHLASVYDTVE
ANLTGRIYVSQHENITHDHYLQVVKTEVVSLRKRKEFSLLEQYDYTSHSNTIQNTNVPVAKFHYELSPMQV
LVKENPKSFSHFITNVCAIIGGVFTVAGIVDSMLHGAMRMVKKIELGKQF

NCBI Reference Sequence: XP_006354000.1
>S.tuberosumERV-A1
MDSFISKIRSLDAYPKINEDFYSRTLSGGVITLASSIIMTLLFISELRLYLHAATETKLIVDTSRGETLRI
NFDITFPAIPCSILSVDAMDISGEQHLDIRHDIIKKRIDVLGNVIETRKEGIGSPTIDRPLQRHGGRLEHN
ETYCGSCYGSEGSDDHCCNTCEDVREAYRKKGWALTNPDEIDQCKREGFLEKIKEEEGEGCNMYGFLEVNK
VAGNFHFAPGKSFQQSNVHVHDLLTFQKDSYNISHKINRLTYGEYFPGVVNPLDGVMWTQETPHGMYQYFI
KVVPTVYTDVSGHTIQSNQFSVTEHFQGADFGRFQSIPGVFFFYDLSPIKVTFTEQHVSFLHFLTNVCAIV
GGVFTVSGILDSFIYHGQKAIKKKMELGKFS

NCBI Reference Sequence: XP_006351793.1
>S.tuberosumERV-A2
MERVFSKLRNLDAYPKINEDFYNRTLSGGIITLVASIIMLVLFVNELGLYIHSYTETQLVVDTSRGGKLHI
HFDITFPAVPCSLLSLDARDISGEEHFDIRHDIFKKRIDSHGAVIEVRQDGIGAPKIEKPLQRHGGRLEHN
ETYCGSCFGAETADDECCNSCEEVREAYRKRGWGMTNPDLIDQCKREGFVQKIKDEEGEGCNIHGSLEVNK
VAGNFHFAAGKSFHQSTFQLFELISLQSDTYNISHRVNKLAFGDSIPGVVNPLDGVQWTQEAQNGMYQYFI
KVVPTIYKGVRGRTIDSNQFSVTEHFKGSDLGLFQSITGVYFFYDLSPIKVTFTEEHVSFFHFLTNVCAII
GGVFTVAGILDACIYHSQKAIKKKVELGKFG

Phytozome *Solanum tuberosum* v3.4 proteome: PGSC0003DMT400002424
>S.tuberosumERV-B1
MGVKQVLRAMDAFPRAEEHLLQKTKSGAFVSIVGLVIMSTLFLHELSYYLNTITVHQMAVDLRRGETLPIH
INMTFPSLPCDVLSVDAIDMSGKHEVDLDTNIWKLRLNSDGHITGTEYLSDLVEKEHKHDDSDNKTHMQGF
DQDAENLIKKVKQALAHGEGCRVYGVLDVQRVAGNFHISVHGLNIFVAQMIFGGTTHVNVSHIIHDLSFGP
KYPGSHNPLDGTERILRGASGTFKYYIKIVPTEYRYLSKEVLPTNQFSVSEYFSPINEFDRTWPAVYFLYD
LSPITVTIREERRNFLHFITRLCAVLGGTFALTGMLDKWMFRFLEAVMKKDSRGLVR

NCBI Reference Sequence: XP_006351995.1
>S.tuberosumERV-B2
MGVKQALRAIDAFPRAEEHLLQKTKFGAFVSIVGLLIMVTLFLHELSYYLNIYTVHQMSVDSRRGENLPIH
INMTFPSLPCDVLSVDAIDMSGKHEVDLDTNIWKLRLNSDGHITGTEYLSDLVEKEHEVHKHDAHKEHHED
SDKIHLQGIDEESQNMIKKVKQALADGEGCRVYGILDVQRVAGNFHLSVHGMNIFVAQMIFEKSTHVNVSH
IIHDLSFGPKYPGIHNPLDGTSRILRGSSGTFKYYIKVVPTEYRYISKEVSPTNQFSVTEYFSPIHDFERT
WPAVYFLYDLSPITVTIREERRSFLHFITRLCAVLGGTFALTGMLDGWMYKILESFTKKNSRTIVR

NCBI Reference Sequence: XP_006366039.1
>S.tuberosumPDI-C
MVSTSKIKSVDFYRKIPRDLTEASLSGAGLSIVAALSMMFLFGMELNNYLTVSTTTSVVVDKSSDAEFLRI
DFNMSFPALSCEFASVDVSDVLGTNRLNITKTVRKHSIDKNLRPTGSEFHSGSTATELKHDAEDDEEYGEG
SVSLNGHSFDRVTHHYPILVVNFFAPWCYWSTRLKPSWEKAANIIRERYDRESDGRILVAKVDCTEEVDLC
RRNHIQGYPSIRIFRKGSDVRDDHGHHDHESYYGDRDTDSLVKMMEDLVAPIKLDSQMISSDNSSTKLETG
LKRPAPVTGGCRIEGFVRVKKVPGNLVISARSAAHSFDASQMNMSHVISSFSFGKTITPKVMSDIKILLPH
LGRSHDRLNGNSYVTNPRDSTENVTIEHFLQVVKTEVMTRSYKLVEEYEYTAHSSLVHSLHIPVVKFHYEP
SPMQVLITENPKSFSHFLTNVCAIIGGVFTVAGILDSILHNTMRMVKKVELGKNF

NCBI Reference Sequence: XP_007018816.1
>T.cacaoERV-A1

MDGIMNKLRNLDAYPKINEDFYSRTLSGGVITLVSSVVMFFLFFSELRLYLHAVTETKLVVDTSRGETLRI
NFDVTFPALACSILSLDAMDISGEQHLDVRHDIIKKRLDAHGNVIESRQDGIGAPKIEKPLQRHGGRLEHN
ETYCGSCYGAEASDDDCCNSCEDVREAYRKKGWALSNPDLVDQCKREGFLQKIKDEEGEGCNIYGFLEVNK
VAGNFHFAPGKSFQQSNVHVHDLLAFQKDSFNISHKINRLAFGDYFPGVVNPLDGVHWTQEQPSGMYQYFI
KVVPTVYTDVSGHTIQSNQFSVTEHFKGAEINRLQSLPGVFFFYDLSPIKVTFTEQHVSFLHFLTNVCAIV
GGVFTVSGILDSFIYHGQRAIKKKIEIGKYS

NCBI Reference Sequence: XP_007042992.1
>T.cacaoERV-A2
MENVFNKLRNLDAYPKVNEDFYSRTLSGGIITLVSSLAIFFLFFSEFRLYLHTVTETKLLVDTSRGEALRI
NFDVTFPAIPCTLLNLDAMDISGEQHLDIKHDIIKKRINAHGDVIESRQDGIGAPKIEKPLQRHGGRLEHN
ETYCGSCFGAEQSDDDCCNSCEEVREAYRRKGWAMTNVDLIDQCKREGFIQRVKDEDGEGCNIHGSLEVNK
VAGNFHFAPGKSFHQTNIFLSDLLAFQKDSYNISHRINRLAFGEYFPGVVNPLDGAQWIHETSNGMYQYFI
KVVPTIYTDIRGRTVHSNQYSVTQHFKNLEVIYPNSHPGVFFIYDFSPIKVTFKEEHISFLHFITNICAVI
GGIFTVAGIIDSFVYHGQRRMKKKMEIGKFR

NCBI Reference Sequence: XP_007048888.1
>T.cacaoERV-B
MGVKQALKSLDAFPRAEEHLLQKTQSGALVSIVGLVIMAALFFHELTYYLTTYTVHQMSVDLKRGETLPIH
INMTFPSLPCDVLSVDAIDMSGKHEVDLDTNIWKLRLNSLGQIVGTEYLSDLVEKEHAAHKHDDEKEHHDD
SDKKLHALGFDQEAENMIKKVKQALANGEGCRVYGVLDVQRVAGNFHISVHGLNIYVAQMIFGGATHVNVS
HMIHDLSFGPKYPGLHNPLDGTVRILHDTSGTFKYYIKIVPTEYRYISKEVLPTNQFSVSEYFSPMHEFDR
TWPAVYFLYDLSPITVTIKEERRSFLHFITRLCAVLGGTFALTGMLDRWMFRLIEGLTKPSHKGVLR

NCBI Reference Sequence: XP_007021017.1
>T.cacaoPDI-C
MISSSKIKSVDFYRKIPRDLTEASLSGAGLSIVAALAMMFLFGMELSNYLTVSTSTSVIVDKSSDGEFLRI
DFNISFPALSCEFASVDVSDVLGTNRLNITKTIRKFSIDPHLRSTGAEFHAGPVPHFIKHGDEVDEETVEG
SVPLNGVSFDKLSHLYPILVVNFYAPWCYWSNRLKPSWEKAAKIIKERYDPEMDGRILLAKVDCTEEVDLC
RRHHIQGYPSIRIFRKGSDLREDHGHHDHESYYGDRDTESLVKTMEELVAPIPLESQKLALEDKSNITKRP
APKTGGCRIEGYVRVKKVPGNLIISARSAAHSFDASQMNMSHVISHLSFGKTISPRVLSDVKRLIPYIGRS
HDRLNGRSFINHRELDANVTIEHYLQIVKTEVVTRRSSREHTLIEEYEYTAHSSLAQSIYIPVTKFHFELS
PMQVLITENPKSFSHFITNVCAIIGGVFTVAGILDSILHNTIRLMKKVELGKNF

NCBI Reference Sequence: XP_002285801.1
>V.viniferaERV-A1
MDNIINKLRNLDAYPKINEDFYSRTLSGGVITLASSIFMLLLFISELRLYLHAVTETKLVVDTSRGETLRI
NFDVTFPALPCSILSLDAMDISGEQHLDVRHDIIKKRIDAHGSVIEARQDGIGSPKIEKPLQKHGGRLEHN
ETYCGSCYGAEASDDDCCNNCEEVREAYRKKGWAMSNPDLIDQCKREGFLQRIKDEEGEGCNIYGFLEVNK
VAGNFHFAPGKSFQQSNIHVHDLLAFQKDSFNISHKINRLAFGDYFPGVVNPLDGVQWIQATPSGMYQYFI
KVVPTVYTHVSGHTISTNQFSVTEHFRNAELGRLQSLPGVFFFYDLSPIKVTFTEEHVSFLHFLTNVCAIV
GGVFTVSGILDSFIYHSQKAIKKKIEIGKFS

NCBI Reference Sequence: XP_002264644.1
>V.viniferaERV-A2
MDRVFQRLRNLDAYPKINEDFYSRTFSGGLITLISSIVMLFLFFSELRLYLHTVTETKLVVDTSRGGTLRI
NFDVTFPAVPCSVLTLDAMDISGEQHHDIKHDIVKKRIDAHGNVVAVRQDGIGGPQIEKPLQRHGGRLEHN
EKYCGSCYGAEVTDDDCCNSCDEVREAYRKKGWGMTNPDLIDQCKREGFVQKVKEEEGEGCNVYGFLEVNK
VAGNFHFSPGKGFYQSNIHVNDLLAISKDGYNISHRINKLAFGDHFPGVVNPLDGAQWFQDAPDGMYQYFI
KVVPTIYTDIRGHTIQSNQFSVTEHFRSAEPGRPHSLPGVYFFYDLSPIKVTSKEEHSSFLHFMTNICAIV
GGIFTVSGIIDSFVYHGHRAIKKKMELGKFS

NCBI Reference Sequence: XP_010656946.1
>V.viniferaERV-B
MGVKQFIKSLHAFPRAEEHLLQKTQSGAVVSIIGLVIMATLFLHELRYYLTTYTVHQMSVDLKRGETLPIH
INMTFPSLPCDVLSVDAIDMSGKHEVDLDTNIWKLRLNRDGFIIGTEYLSDLVEKEHADHKHDHNKDHHGD
SDQKLHAHSFDQDAENMVKKVKQALANGEGCRVYGVLDVQRVAGNFHISVHGLNIFVAQMIFDGAIHVNVS

HIIHDLSFGPKYPGLHNPLDGTVRILRGASGTFKYYIKIVPTEYRYISKEVLPTNQFSVMEYFSPMNEFDR
TWPAVYFLYDLSPVTVTIKEERRSFLHFITRLCAVLGGTFALTGMLDRWMYRFLEMLTKPNAKSVYR

NCBI Reference Sequence: XP_002281649.1
>V.viniferaPDI-C
MVSTSKIKSVDFYRKIPRDLTEASLSGAGLSVIAALSMMFLFGMELSNYLSVSTSTSVIVDQSSDGDFLRI
EFNISFPALSCEFASVDVSDVLGTNRLNITKTIRKYSIDPDLRPTGAEFHSGPVGKVIKHGDETDEEYSEG
SASLTAQNFYKYSHQHAILVVNFFAPWCYWSTRLKPSWEKAAKIIERYDPELDGRIVMAKVDCTEEGELC
RRHHIQGYPSIRIFRKGSDVRDDHGHHDHESYYGDRDTDTLVTTMETLVAPIPLESQRLALENKSDSTADH
IKRPAPRTGGCRIEGFVRVKKVPGNLVISARSGSHSFDPSQMNMSHVISHLSFGRKIAPRVMSDMKRVLPY
IGGSHDRLNGRSYISHPSDSNANVTIEHYLQVVKTEVITTRDHKLVEEYEYTAHSSLVQSLYIPVAKFHFE
LSPMQVLVTENRKSFWHFITNVCAIIGGVFTVAGILDSVLHNTMRLMKKIELGKNF

NCBI Reference Sequence: NP_001148795.1
>Z.maysERV-A1
MDGLLSKLRSLDAYPKVNEDFYSRTLSGGIITLVSSAVMLLLFVSELRLYLHAVTETTLRVDTSRGETLRI
NFDVTFPALQCSIISLDAMDISGQEHLDVKHDVFKQRIDAHGNVIATRQDVVGGMKMEAPLQHHGGRLEHN
ETYCGSCYGAQESDDQCCNTCEDVREAYRKKGWGVSNPDLLDQCKREGFLQSIKDEEGEGCNIYGFIEVNK
VAGNFHFAPGKSFQQSNVHVHDLLPFQKDSFNVSHKINRLSFGEYFPGVVNPLDGANWVQHSSYGMYQYFI
KVVPTVYTDINEHIILSNQFSVTEHFRSGESGRMQALPGVFFFYDLSPIKVTFTEQHVSFLHFLTNVCAIV
GGVFTVSGIIDSFVYHSQRAIKKKMEIGKFN

NCBI Reference Sequence: NP_001141198.1
>Z.maysERV-A2
MDAFLQRLKRLDAYPKVNEDFYKRTLSGGIVTLVAAVVMLLLFISETRSYFYSATETKLVVDTSRGERLRV
NFDITFLSIPCTLLSVDTMDISGEQHQDIRHDIEKIRLDAHGNVIEARKVSIGGAKIERPLQKHGGRLDKG
EQYCGTCYGAEESDEQCCNSCEEVREAYKKKGWALTNPDLIDQCAREDFVERVKTQQDEGCNVHGFLDVSK
VAGNFHFAPGKGFYESNIDVPELSLLEGGFNITHKINKLSFGTEFPGVVNPLDGAQWTQPASDGTYQYFIK
VVPTIYTDIRGHNIHSNQFSVTEHFRDGNVRPKPQPGVFFFYDFSPIKVIFTEESRSLLHYLTNLCAIVGG
VFTVSGIIDSFIYHGQKALKKKMELGKYR

NCBI Reference Sequence: NP_001132255.1
>Z.maysERV-A3
MDAFLHRLKRLDAYPKVNEDFYKRTLSGGIVTLVAAVVMLLLFISETRSYFYSSTETKLVVDTSRGERLRV
NFDITFPSIPCTLLSVDTTDISGEQHHDIRHDIEKRRLNSHGNVIEARKEGIGGAKVERPLQKHGGRLDKG
EQYCGTCYGAEESDEQCCNSCEEVREAYKKKGWALTNPDLIDQCAREDFIDRVKTQQDEGCNVLGFLDVSK
VAGNFHFAPGKGFYESNIDVPELSLLEGGFNISHKINKLSFGTEFPGVVNPLDGAQWTQPASDGTYQYFIK
VVPTIYTDIRGRGIHSNQFSVTEHFRDGNVRPKSQPGVFFFYDFSPIKVIFTEENRSLLHYLTNLCAIVGG
VFTVSGIIDSFIYHGQKALKKKMELGKYR

NCBI Reference Sequence: XP_008654279.1
>Z.maysERV-B
MARIPSLKSLNAFPHAEEHLLKKTYSGAVVTIFGLLIMITLFVHELQFYLTTYTVHQMSVDLKRGETLPIH
INMSFPSLPCEVLSVDAIDMSGKHEVDLHTNIWKLRLDKYGHIIGTEYLSDLVEKGHGAHHDHDHDHDHHD
EQKKHEQTFNEEAEKMIKSVKQALGNGEGCRVYGMLDVQRVAGNFHISVHGLNIFVAEKIFEGSNHVNVSH
VIHELSFGPKYPGIHNPLDETSRILHDTSGTFKYYIKVVPTEYKYLSKKVLPTNQFSVTEYFLPIRPTDRA
WPAVYFLYDLSPITVTIKEERRNFLHFVTRLCAVLGGTFAMTGMLDRWMYQLIKTVTNSKTRSVLR

NCBI Reference Sequence: NP_001105762.1
>Z.maysPDI-C
MISSSKLKSVDFYRKIPRDLTEASLSGAGLSIVAALAMVFLFGMELSSYLAVNTTTSVIVDRSSDGEFLRI
DFNMSFPALSCEFASVDVSDVLGTNRLNITKTVRKYSIDRNLVPTGSEFHPGPIPILNKHGDDVEEDHVDG
AFSLSSRNFDSFSHQYPVLVVNFYAPWCYWSNRLKPSWEKTAKIMRERYDPEMDGRILLGKVDCTEEVELC
RRNHIQGYPSIRVFRKGSDIKENQGHHDHESYYGERDTESLVAAMETYVANIPKEAHALEDKSNKTVDPAK
RPAPMASGCRIEGFVRVKRVPGSVVISARSGSHSFDPSQINVSHYVTQFSFGKRLSPRMLHEFIRLTPYLR
GYHDRLAGQSYTVKHGEVNANVTIEHYLQVVKTELVTQRSSKELKVLEEYEYTAHSSLVHSFYVPVVKFHF
EPSPMQVLVTEVPKSFSHFITNVCAIIGGVFTVAGILDSIFHNTLRMVKKIELGKNI

Phytozome *Chlamydomonas reinhardtii* v5.5 proteome: Cre13.g579850.t2.1
>C.reinhardtiiERV-A
MSGGGFLGKLKALDAYPKINEDFFTKTMSGGIITIVSSVVMVLLFLSELRLFLTTSSAHELSVDVGRGEKI
KIHFDVTFPKVPCAWLSLDAMDISGELHLDLDHDVYKQRLNANGSPVKEVEKHNVDATKKKPPSAVNATAS
AAAGGAPAGGAAGAAGAEGGAAGGAGGGENATALANGCGSCYGAEDKQGDCCNTCDEVRAAYRRKGWALSN
VDHIEQCAHDLYTEAIKEQAGEGCHMWGMLEVNKVAGNFHFAPGRSYQQGSMHVHDIAPFGDAVIDFRHVI
HKLSFGEPYPGMKNPLDGAKAGQAAAAAAAATGMFQYFLKVVPTSYTDLSNKTLSTNQFSVTENFREAQGG
AGRTLPGVFFFYDLSPIKVKIVEHGSSFLSFLTSVCAIVGGVFTVSGIVDAFVYTGTRMIKKKMELGKFS

Phytozome *Chlamydomonas reinhardtii* v5.5 proteome: Cre08.g358579.t1.1
>C.reinhardtiiERV-B
MGKGFRLSSLSAYVKPEAHLVNQTIHGALVTLCGVLLATLLTLHEIKSFYQMHRVTVLSVDLARRHALTIN
LDITFPSVPCAVLSIDVLDISGTAENDASFAHHMRVHKMRLDKAGNQIGKAEYHTPQSQQIMDTGGEQLVS
VNIQEAMQHLVDMEDEADHHEGCHVYGTMEVKRVAGRLHLSVHQNMVFQMLPQLLGTHHIPKILNMSHVIK
HLGFGPHYPGQLNPLDGYVRMVGREPFSYKYFLKVVPTEYYNRLGRATETHQYSVTEYAQPLQRGYAPAVD
VHYDLSPIVMTINERPPSLLHFVVRLCAVVGGVFAITRLTDRWVDWLVRLVNKAAARGP

NCBI Reference Sequence: XP_001699770.1
>C.reinhardtiiPDI-C
MVRLFSRLKAIDFFKKIPSDLTEATLTGAWLSIVAAVLMILLFVAELSAFLSTTTSSQLVVDRSPQNELLK
LNFNISFPALSCEFATVDVSDSLGTKRMNLTKTVRKVPITLDMERQGAAVEDTAHKVGPKYDAEGHFDEEP
DIDITVPLSHENFEATLARYPIAVINFYAPWCHWCQRLEPTWEAATKEVHDKYPEWDGRVRFAKVDCTAEV
DLCRQHFIQGFPSIRVFRKGHDDIYIGGMHEHEAYMGDRTKDALVAFADSLVPSAGQPHRKLAGLSAAPKT
PGCNLAGFVMVKKVPGTVHFVARSEGHSFDHTWMNMTHMIHSFHVGTRPSPRKYQQLKRLHPAGLTADWAD
KLHDQLFVSEHTQSTHEHYLQVVLTTIEPRHSRHTGNYDAYEYTAHSHSYQSDSIPSARFTYDLSPIQILV
HETSKPWYQFLTTSCAIIGGVFTVAGILDALLYQSFKVVKKLNLGKQG

NCBI Reference Sequence: XP_005650551.1
>C.subellipsoideaERV-A
MEGIVSKLKNLDAYPKVNEDFFQRTLSGGIITIGSSIIMLCLFLSELSLFMKITTTNELSVDTTRGDQLSI
NFDMTFPALPCEWISLDLMDISGEMHLDVDHDVYKRRLDSNGVVIPDSIEKHQVGPELDDTLLHKANETEC
GSCYGAAPDEECCNNCEEVRAAYRRKGWGFTDPQQISQCAKEGFVEKLRAQEGEGCHMWGSLAVNKVAGNF
HFAPGKSFQQGPMHVHDLVPFQGVTFDLSHRIDKLSFGHEYPGMTNPLDRVNLPKFNTRNPQGLPGAYQYF
LKVVPTIYVNSHNHTINSNQYSVTEHFKGSQDFQAQLPGVFFYYDLSPIKVKYHETRMSFLHFLTSVCAIV
GGIFTVAGIVDAFIYHGHQAIKKKVDLGKQI

NCBI Reference Sequence: XP_005651581.1
>C.subellipsoideaERV-B
MKLKSFNRFSAYARAESHLVQRTYFGAIVTVLGVILAIVLFANELREYTTPFSIQTMSVDTSRAHYIRMNF
NFTYPSMPCQVLSLDATDMSGEKSGDSGHAANGEIHKVRLNEAGEKIGLGEYIPPRRWGFMMGKPRQ-EVM
EVNQAMDAHEGCNIFGWLDLQRVAGNFRVSVHVEDFFALTRLQADTTGINSSHIIHRVSFGPTFPGQVNPL
DGAERILDKESGTFKYFLKVVPTEYVKLDGTRTTTNQYSVTEYDTVVHKGEMQMPSVWFSYDISPISVTIS
EIRKSFAHLLVRFCAVVGGVFAVTGMFDRWVHRIVTAIFSASS

NCBI Reference Sequence: XP_005642637.1
>C.subellipsoideaPDI-C
MARVLQKLRSVDFYRKIPNDLTEATLAGAGISLVAAFTIVVLLTAELSSFLAIETKEELIVDRSAHGDLLR
INFNISFPSLSCEFATLDVSDALGTKRMNLTKTIRKLPIDEDGQRAGYYVHDDLSNVDIKYDEPSVSQDFA
LPLSKDSFKATLEAYSIVVVNFYAPWCPWCQRLEPTWEAVTQEVHTKYPDADGRIRFAKVDCTTEVDLCRE
HQITGFPSIRVFRSGHDEVNVHGVKEHESYRGDRTQASLLAFADNLAPSAGQPHHYIRGVTRMAKTSGCAL
SGFVLVKKVPGALHFLAKSPGHSFDYQAMNMSHVVNYLYFGNKPSPRRHQSLAKLHPAGLSDDWADKLAGQ
DFFSRAAKATFEHYMQVVLTTIEPSKHRPELSYDAYEYTVHSHTYDTADIPAAKFTYDLSPIQILVSEKRR
AWYHFVTTTCAIIGGVFTVAGIVDGLVHTGARFAKKVELGKHT

NCBI Reference Sequence: XP_002948747.1
>V.carteriERV-A

MSNSGGGFLSKLKALDAYPKINEDFFTKTMSGGIITIVASVVMVLLFLSELRLYMTTQSVHELSVDVGRGE
KIQIHFDLTFPKVPCSWLSLDAMDISGELHLDLDHDVYKQRLSANGSPVKEVEKHNVEATKKVVPVNGTEN
STATPVCGSCYGAEDRQGDCCNTCDEVRAAYRRKGWALANVDHIEQCAHDLYTESIKEQTGEGCHMWGMLE
VNKVAGNFHFAPGRSYQQGSMHVHDIAPFGDAVIDFRHTVNKLSFGAPYPGMKNPLDNAKAGYKSAAATGM
YQYFLKVVPTSYTGIDNKTLATNQFSVTENFRESSQGGAGKTLPGVFFFYDLSPIKVRIVEHSSSFLSFLT
SVCAIVGGVFTVSGIVDAFIYTSTRLIRKKMELGKFS

NCBI Reference Sequence: XP_002958233.1
>V.carteriERV-B
MKFKLSSLSAYVKPEAHLVQQTVHGALVTLCGILLAAMLFVHELGSFYRQHRVTQMSVDLARRNALTINID
LTFPAIPCAVLSIDVLDIAGTAENDASYAHHMHIHKLRLDGAGKPIGKAEYHTPQSQQIMDTGAEQLVSVN
IQEAMQHLVDMEEEAEHHEGCHVYGTMDVKRVAGRLHFSVHQNMVFQMLPQLLGAHRIPKVANISHTIKHL
GFGPHYPGQLNPLDGYVRMVKGPPQSFKYFLKVVPTEYYNRLGRVTETHQYSVTEYTQPLEPGYVPTLDVH
YDLSPIVMTINERPPSLLHFVVRLCAVVGGAFAITRMTDRWVDWFVRLVTKLK

Phytozome *Volvox carteri* v2.0 proteome: Vocar20014205m
>V.carteriPDI-C
MARLFSKLKAIDFFKKIPSDLTEATLTGAWISILAAVIMVFLFTAEMMSFLSTTTTTQLIVDRSPQNELLK
LNFNISFPALSCEFATVDVSDTLGTKRMNLTKTVRKMPITTELERMGSAVEDSSHKPGPKYDEEGRFDDEP
DIDITVPLSHVNFEATLARYPIVVVNFFAPWCHWCQRLEPTWEAATKEVHDKYPEWDGRIRFAKVDCTQEM
ELCRTHFIQGFPSIRVFRKGHDDIVIGGMHEHESYMGDRTKEALVAFADSLVPSAGQPHRKHAALSAAPKT
PGCNLAGFVMVKKVPGTLTVVARSEGHSFDHTWMNMTHLVHTFHVGTRPSPRKYQQLKRLHPAGLTHDWAD
QLRDQFFLSEHPQSTHEHYLQIVLTSIEPRRSRHSGNYDAYEYTAHSHTYQSDAIPSARFTYDLSPIQILV
QETARPWYQFLTTSCAIIGGVFTVAGILDALLYQSFKVVKKLNLGKQG
