# Supplementary material for: Phylogenetic characterization and promoter expression analysis of a novel hybrid protein disulfide isomerase/cargo receptor subfamily unique to plants and chromalveolates
Source: Mol Genet Genomics. 2015 Aug 25;291:455–69. doi: 10.1007/s00438-015-1106-7 (PMC4729789; doi:10.1007/s00438-015-1106-7)
Supplement: Supplementary file 2 — Online Resource 2. Sequences of non-plant PDI-C isoforms identified by database searches. Corrections made to the original sequences due to available EST data or an alternate gene prediction model are highlighted in yellow (PDF 96 kb) [file 438_2015_1106_MOESM2_ESM.pdf]

Non-plant PDI-C Isoforms

NCBI Reference Sequence: XP_005716385.1
>C.crispusPDI-C1
MLKLSSYDVFRKLPRDLTHGTSQGGVLSVLAVALILLVFLFELWTYVAGEVETAVMLDTNREALLQINFK
VTALRLPCEFASVDVWDYLGNTRLDLTKNLHKTMVSGPRGRDILGAYSDGNAFSNGDSEDEVKAHIGLSE
SEEIMQSNFDQKLAENAWTFVDFFAPWCIHCVKLAPTWEAFAKTVHDKDIRVKVFKVDCTKNEFLCRSQQ
ISGYPTLRVYKGNHPQHPDYFGKRTVSTLISWMESMTHEHAHAKTRLHKEEGCLVEGAIWVNRVPGNFHI
TAKSKGHDFDPKAMNLSHVVHHFSFGKPLPRRVIRHLPDDVKRNINPLDGRSFVNTEEHTTHEHYIKVVS
THYRVGKSSIFNREDSLGYQMATSNHRYKSDPGIPEAKFSFDLSPTAVVIIQGGKRWYEFVTSLCAIIGG
IFTVVSLFDGAMYSISKRVKGVQGKLS

NCBI Reference Sequence: XP_005715706.1
>C.crispusPDI-C2
MFKISMLDLFRKVPVDLTQATRRGGLLSLAVATIIGLVLFCEVWTYLEGETKSRIILDSNTESKLEINFE
ISFFELPCRFANVEVWDYLGNAKLNVDADISKTILGHEQNENHLTEYKDKGTVATQLVDKHEDAHLPEQV
VELTSSNYGQYLKQNEYTFVIYYVNWCGFCKMALPVWSKFAKHLPSIRPNVKIAQVDCEKNAELCQATKI
TGYPTFIMFKGVNPLEEDYGGERSVEAFSKYVEKVSDTPPDDHNLKYQWQEGCLLGGRLLVNRVPGNFHI
SAKSDAHNFDQKSTNTSHLIHHLSFGGEMQHDLYMAVPKDIRVNISPLDDILFINHKEHMSHEHYIKVVS
THYQAGSLRRRKDVLGYQMATSNHQYKSDPNVPETRFSYDLSPTAVVISQAGRRWYEFLTSLCAIIGGTF
TTFSLMDGVLHSMQRRLARPVTPSSKLT

The *Porphyridium purpureum* Genome Project: evm.model.contig_3636.1
>P.purpureumPDI-C1
MRLAVYDLYRKVREGAESTVTGGFLSVLGVALIVVLGIAETVSFLGAPKSMAVEVDRNMDESLQVNLDIL
LMRMPCNMVSLETWDYTGTSQLRHEDLSTKYVVFGDQGEFNLGEAGVMGHLEHMKNEHLLEQVTHEAAEE
LVDANFASKLNKEPLAIVKFYTPWCIWCQRLVPHFAALPDALAQVGLPGTKLFKVNCVENLESCRGIAGF
PTVRYFVKGKPQTPDYRGDRTAQDMAQWVANGVKKVDSASGETNKFVVRKQAHGTVMRRVADENLPEGCR
IIGALKVSRVPSNIHIVAAATTFDINPREVNLSHRVNIFSFGSMMSPQRLLRLPIFAQYNVNPFNGQSFV
ATEKHILFDHYLKVVTTRFARHRGKRAWSLDDALQDRRAYLVTASTKSTIADPNVPEIRFSYDFSPFATV
VFQKTDKTWYDYLTNLCALIGGVWTILQLVDSTLFNLVHSKIKTMQGKLG

The *Porphyridium purpureum* Genome Project: evm.model.contig_2262.7
>P.purpureumPDI-C2
MVVVEGNRASKAAWRMRFGELDLFRKVPRDLVQSTRLGGALSLLALALLSVLLMTELSDMGRATTSTSLQ
VDRAPSSEPVTLSFRIALMSLPCKYVDVQLLDTRLRTATQSQRLVLKKTELADGAKYLDSLSKPLEQIRR
YDKQGVVRPLGAPEEDNDSADQSGVTITVEQFRNNIVTRSPYTLVYFYLPYCPFCKSLVPVWEEVERKIS
SDVARFSRGVIKVVKIDCITSKELCIDQHVMAVPTIRVYERDTVLRPDYHSAKREAEDILAWVLGKITGA
DAKRAAAASGQDAAREISSKEGCVVSGSAQVQKSPAFMRISANAENYSVEAQHVNMTHLVEYFTANYEIP
GDTTSKLHPMIALNLDTMHHTAFVTSKSQEGMSFDHYLKFIPTTYRSRWGPKQRLFQMTAASHGYYKQHG
AAEIRVSYDFSPLVHVVEKQNTKQWYEFLAKLFAIIGGVICVVGLMDKGIYTYVAYQQKVRQGKQI

NCBI Reference Sequence: XP_005786077.1
>E.huxleyiPDI-C1
MGIADALESLHLYRKVPRDLTDATRLGGAISLATAFLMAYLFLSNIAEFMSVRSSTDVALDDSTEAKMRI
YFNITMERLPCQFASVDVSDVMGTSLTNVTQHIIKFKVAPEAGHRKAEYYRERDDDVEHEVLDDEEQGYL
DTLPATLPQLNDHSFEQTIKSYDLVLVAFGAPWCPWSQRLDPVWRKTWELLKQKPYRDHVRIGKVDCTAS
DSHSTCQKHHIHAFPTIRIYRHRQVHSHENYLGNRDSAVFIEFVEEALPKHLVTGGGGAPLDADKAAAAA
RSVESHTLAGEGCQLTGSLEISRVPGSFRISAQSDAHSFNSRVMNVSHHVDKLLFAYTDERPAKTHKVIS
IEERSSLYQMGFMMHQELATLKHYLKVVPFHHHDLSGHVTQAYLYKANYNEYRPRKLEWYEGKADAHVDT
QMVPNAVFHYDISPVKVVVQEESEALAAFVTKICAVIGGIYTVVGLLDSVMYHSVQSFSKKK

NCBI Reference Sequence: XP_005782141.1
>E.huxleyiPDI-C2
MVRLRDAHFFRKVPVDVSEGTAVGGLLSVAAVLVACWLVSEEISAYSTAKLTTQMALDHTAMPSPLGPAA
YESIRVNFNISMLRIPCQYATLFVSDHVGAHKSGGARNMHKVRLDREGRSLGMYSPHRYAGSYRPGPYQL
DDGTALDEQGFQALAKSKAGVMVNFFAPWCFWSQKLVPAWEAVGRRLHARAYSQSFAFVRVDCTSAKGRD

LCRSHSIHAFPTVRTYRGSVHAFEPYEYGREENVIWLHMVKIAAEVVVQRMHDPSPLDEDGSPLEMWEEE
AGHEGCNLFGYVEVSRAPGTIHLAPHSSRHSFDFSHVNTSHHIDHLSFGLELGAIERRGLPPDVRKHLLS
LDGRAFTAATAHLTQEHHVNIVPTRYQPAGSVAHVETYQFTATSHARTKDTLPSLLISYDVSPIQVNIAE
RRQPTSELVLSFCAIIGGVVAVFGIIDGMLYTGSAVVRQKLSAGKLH

NCBI Reference Sequence: XP_005783917.1
>E.huxleyiPDI-C3
--------------MGRKSYGPVLNYFKHVPDDLVVTSTPGLIISALGTVVLITLFLFELSAYLTVTSTT
DLVVDELVDETLRVNFNVTLHQVPCEFLSVDVSDMTGAATHDIRKDILKWRLDSNQRVVRDAAAVSAVET
RDAKRAADAAMSKEPVSPSYGDEDEEEPPDTDLSQPLSPDTFVPFLQQHELSLVNFYAPWCIWCQRLEPV
YLEAAAKVPSLEFHGHTRLAQVDCVAFQEFCVKHMIRAYPTLRMYKDGNDKEFELFTDERSVGAILGFVR
KQMDSYRTSHAVLRKQHAARFEVRRGALSAGRDLYRAKMPIVAANTFCGNNEECIGFTYAAEEMPAKDAK
PDPFMPGENPLIYFKSGRAGEAQVNADKQWTSYLKINNATAPVSSSGSLHHGPEGCMVAGHLRVRKVPGV
LKLVLHSPEHDHEHALINSSHAVNEFWYGEPLSRFQRSRLSEADRFEIDSPTSHRLESIPFISDAAGDSH
VHYLKVVTKVVRHYSARDADTLVYKYTVHSNKYSSPEAQEPSVDFKYDLSPISIVVQQQRMPAYRFVTST
CAIIGGVFTVIGIIEAVLHTATESFLKKQM

NCBI Reference Sequence: XP_005830975.1
>G.thetaPDI-C1
MSGFLQGLKSVDFYRKLKRDLQQELTEASVSGAALSIIAAVIMIGLVAAELTAYLTVQSESRVVLDHFES
SSDDTLQVNFNFTFPHLKCDYASVDATNFMGTHDAGLAARVSKIRLDKNGNLVGRHDDNKKELRHTTDEV
HDGPETSIALTTDNFEASHHKYAIMIVNFYAPWCHWCQRLAPVWEKSAATIAQKFPGDERIILAKVDCTH
QSSEALCIKYRIDAFPTILVFRKDDKGDSQHESYHGERSVPAITQWAEHFMSQLNNEVPKSRTVDANKDG
VVDSHNGVGCMVSGLLHVQRAPGMLKVQAVSDSHEFNWETMDVSHTVNHLSFGPFLSETAWMVLPPHIAA
SVGSLDDRSFTSDQHVPTTHEHYVKVVRHEVTPPSSWKVAQITSYGYVVHSNNIQKAGEVPTVRINYDIL
PIIVQFHEKKQAFYHFVTNLCAIVGGVFTVAGIIASLMDKSINLMRKKQELGKLG

NCBI Reference Sequence: XP_005841401.1
>G.thetaPDI-C1
MPTLAQMALSWRKIKHDLNAELTEGTITGSIISILTGVLMVYLIVAQIFAWRALNSETSVVLDHYSHMKT
GADSLLQINFNFTFNHLSCEYASVDAANFMGTHDAGISSKVTKVHLDKNGRQLGVHKERKNLKHTIDEAP
HEGESKLITLTAGDFDKHRFEHEILVVNFYTPWCHWCQKLEPVWEKSAKKFGEAHPDDARLVLAKVDCTS
DKAESLCTKYHIDAFPSIMVFRKDDPLDKDHEKYHGERSVDAIVSWAEHLMKQVNLQAPKSRVVDKEQDG
EKESHNGVGCMVAGMLHVQRAPGSIILQAVSDGHEFNWATMDVSHTVNHLSFGPFLSETAWVVMPPDIAQ
AVGSLDDKKFLSEERTPTVWEHYVKVVKNVVELPRSWGIPPVEAHGYVVHTNKVQRYAEVPTARINYDIL
PIIVHVKTSRESNYHFLTKLCAIVGGVFTVSGIFASMVEGGIASLTHKETIGKLG

JGI Genome Portal: estExt_Genewise1Plus.C_230042
>B.natansPDI-C1
MQSLKQFDFYKRIPADFRVQTSGGGTISMLAALFMTLLFTLELWSFIAGHESTTIVVDQDSATSIWVNFN
VTVPSCHCDHISVDVEDMLGTNVMDIQKNVEKVPLDENGEQMPDLDHSHHHAAPKDHVSEAENLVANEAT
TKDTYEPTVLAADTFTKYVDSHEWVFVYFGASWCHWCQKLDPIWKSAGEVLKNRGAAITLPKVECTKDTS
LCQEHQVHALPTIQLFHNGHLVPPNYRGPRTVNDLVHFAENAAVSKKKAEEAKVANKGKTRSDVGCSVSG
HLLVHRVPGSVQFSVKSGEHNFNREHINFTHVVHHFSFNSVPESWEQMLAVMEDDSHRALVSKWYGRNFN
SPKDHVVHEHYLKVVQTLTRPLGLSSEKQMYEHTISSHSYVSEQLAHVRFHYDLSPMQVVKEWKRRPFYE
FITNLLALIGGTFTVMGIVNEAYNGVVAKARMGKLG

JGI Genome Portal: aug1.35_g11347
>B.natansPDI-C2
MSVNDALKHFDFYKTIQTDIRDKTVAGGAVSLLAAVIMGTPIHSITIDDHDNVSPTAGAFWVNFDITLPA
ITCDHISVDVEDRLGSRIFDIQKNVEKIPLDKEGVEAAILPDEIHIKAPDGHVTLKELQDVVANPDIFKR
TNDTVNLEADKFMEWVESHEWSLVSFRVDWCPWCQMLVPIWKSSAVILSNREVPVPLATVECTKHMDLCK
EAGVTAFPTIKLYHWGKHVAPDYKGKRTVNDIVHFAETAADQKAVAEKAGVNVSDTHRSDVGCTVVGHLF
VHKVPGRIQLSLKSDTHTFNREAVNFTHQVHHLSFNDIPETEEEMEAIMLDHEIQDKVSRWLSQTFTSHT
KHTMHEHYLKVECRLFKDIRDTAISCVVVKMSSEDYVVEYEYKNQGFGWANANRIYEHSISSHSYATDSH
LPAVKFHYDLSPLQVSVEHQSRPWFSFITMVCALVGGAWSIMSVVNDVATSSGLGKMFADAKTLRASS

GenBank: ETO29283.1
>R.filosaPDI-C1
MDVLLKFDAFRKIPKELTNPTLHGAYLTVVAYVVMALLFFMELGAYLSTTVQKSVELDEHFDEHIWIDFD
VQMFELPCEYTHVVVKDIVGNQELEILDQQITKERISVDSNTFKGVIDEEKDAEHRGLNEDIDHHHAQTK
KYEHPELEADWDSTSDQFQHNDFNAVLEYHDFTMVNFYANWCSHCRNFAPTWKEAEDRTDQMEFRDKNGA
VVATKLLRVNCVDFG---------------------------ELCASIGIRAYPTVRLYKHDKSFTQYTG
PREVQGIVNFITDFIHNEDTGKHEVVTHHSSLQEGCRVHGELLVRRVPGYFLLEADSKLDSLDPRMTNVS
HRVNHLYIVGDRDAMRDYVKNTASKVTQDILKNVQPMKRANFITSKAHTAPQHYLNVIPTRFDDKVVVYQ
ATVQSHIADVEITSVPQARFQYIFSPLSIHINTKGRPLYDFLTSVFAIIGGTYTFISLLDKFWDSVSVRL
KKNIGKLG

GenBank: ETO21548.1
>R.filosaPDI-C2
MDFLLKFDAFRKIPKELTNPTLHGAYLTLVAYVVMSLLFTMELYAYLSTSEQKSVELDEHFDEHIWIDFD
VHMFELPCEHTHVIVKDIVGNHELEILDQKISKERIGLDSNEFKGLLDEDKDAEHRGLNKDIDHHLAHIE
KSAHPELEADWDSTSDHFQHTDFNAVLEYHDFTVVNFYANWCIHCRHFAPTWKEAEEETDRMEFRDKNRE
VVVAKLLRVNCVDFGDLCARIGIRAYPTVRLYKHDKSFTQYTGPREKQSIINFVKDFIHNEDTGKHEVVA
HHRLLQEGCRVHGELLVRRVPGYFFLEAESKLDSLDPFMTNVSHRVNHLHIVGDRAAMQDYAKRTASQVT
RDIFKNTEPMKSTSFITFKPHTAPQHYLNVIPTRFDDKVVVYQATVQSHLADVEITSVPQARFQYVFSPL
SIKVSTKGRPLYDFLTSVFAIIGGTYTFISLIDKFWDTISVRFKTQINKLT

GenBank: CCI45882.1
>A.candidaPDI-C1
MGAMDVLKKWDFYKKIPEDLTVSTLPGVSLSIVGCFIMLILFILEFNAYLTVNYAYDIVIDEGLDEKFEI
NFNITIPDLPCEFASIDVSDMTGTRKHNMTKHVAKIRMDGKGRLLGLANAEITHPKYANDDDYGDLPESD
AVVTKLDASNFESFLKEHHYVAVDFFAPWCIWCKRLEPIWTRVAKTLPSLHYGQRMRVASVDCDDHADLC
MKQFVVRAFPTILFYKDGETSPVEMYYGDRTLEAFVDKFKKIFDGEVDDTEVRKKVLFEQDTKHAIQLGRV
APKNAGPEGCQLYGHLIVKRVPGNFHIHLSHPSYSMNSSLVNASHTVNELWFGETLSASALAKLPPHTRL
DSHRLAGQEFTAYMENYTYVHYIKVVTNTYVQRNGEAISAYRYTAHSNEYLETEDLPSVMFRYDLSPMSV
RVTERAMPFYHFVTSACAIIGGVFTVIGIIDQLVHQTVRAMNKKVL

GenBank: CCI46463.1
>A.candidaPDI-C2
MVPKSFSKFDLFRKVPDHLSEHSSLGTVFTVLTFVLSVYLILVNFRSYQGTSNHSIVIMDDHQEDQLRIN
FNISLLAIPCQFASVDVSDYIGMQLINITRHLRHFQLATTDQSSSHVQRVQEIFIHDSNKGLPIWGGVSR
TTRQGVHYSHQLDTETFDVYMKKYELVLINYYAQWCPFSQQLNPEWEKAAAQLQDHPEYSETVAMATVDC
TDRKSAWLCRRAHVRAFPSMLIYIYGNTYTRYIYNGPRDANHILQFLDLFYRRLEPDMDFAEETFLSEGT
GGLAHHQGLNHKNLQSLNAENSQKKVKLPVGAVEGCEVSGSLNVNRVPSRLVFTARSKDLSFDLSGINVT
HIVHHLSFGQVTRKQYSKATQSSMSFDHFSMDGKIFRTENENITVEHFLSVIGVNHMEAKSKRFGLVERT
YEIVARSNQYNATDMLPAALFTFDISPLVIQLSLDSMPLYRFITSLCAIVGGMVTIIGFVDAGVFHTMNS
IKRKRRLGKLN

GenBank: CCI46462.1
>A.candidaPDI-C3
MPMVPKSFSKFDLFRKVPDHLSEHSSLGTVFTVLTFVLSVYLILVNFRSYQGTSNHSIVIMDDHQEDQLR
INFNISLLAIPCQFASVDVSDYIGMQLINITRHLRHFQLATTDQSSSHVQRVQEIFIHDSNKGLPIWGGV
SRTTRQGVHYSHQLDTETFDVYMKKYELVLINYYAQWCPFSQQLNPEWEKAAAQLQDHPEYSETVAMATV
DCTDRKSAWLCRRAHVRAFPSMLIYIYGNTYTRYIYNGPRDANHILQFLDLFYRRLEPDMDFAEETFLSE
GTGGLAHHQGLNHKNLQSLNAENSQKKVKLPVGAVEGCEVSGSLNVNRVPSRLVFTARSKDLSFDLSGIN
VTHIVHHLSFGQVTRKQYSKATQSSMSFDHFSMDGKIFRTENENITVEHFLSVIGVNHMEAKSKRFGLVE
RTYEIVARSNQYNATDMLPAALFTFDISPLVIQLSLDSMPLYRFITSLCAIVGGMVTIIGFVDAGVFHTM
NSIKRKRRLGKLN

GenBank: CCA19024.1
>A.laibachiiPDI-C1
MGAMDVLKKWDFYKKIPEDLTVSTLPGVSLSIVGCFIMLILFILEFNAYLSVNHAYDIVIDEGLDEKFEI
NFNITIPDLPCEFASIDVSDMTGTRKHNMTKNVSKFRIDTKGRLVGFASDEVTHPKYSNDEEYGELPESD

AIVTKLDATNFESFLKEHHYVAVDFFAPWCIWCRRLEPIWTRVAKTLPSLHYGQRMRVASVDCEEHAELC
QKQFVRAFPSILFYKDGETSPVEMYYGDRTLESFVNKFKSLFDGEVDMAEVRKKHYLEQDTKDAAKLGLA
APTNAGPEGCQLYGHLIVKRVPGNFHIHLSHPFYSMNSSLVNASHTVNELWFGEVLSASALAKLPPNTRL
DSHRLARQEFTAYMQNYTYVHYIKVVTNTYVQRNGEVISAYRYTAHSNEYLETEDLPSVMFRYDLSPMSV
RITERSMPFYHFVTSACAIIGGVFTVIGIIDQLVHQTVRAMNKKVL

GenBank: CCA20033.1
>A.laibachiiPDI-C2
MTMVPKSFSKFDLFRKVPEHLSERSSLGTVFTVLTLVLSVYLITVNFRSYQDTSIHSIVVMDDHQEDQLR
INFNISLLAIPCQFASVDVSDYIGMQLINITRHLRHFQLATTAHSPGNVQRVQEIVIHDGDKGLPTWGGV
SRTTREGVHYSHQLDTETFDMFMKKYELVLINYYAQWCPFSQQLNPEWEEAAAQLRDHPEYSETVAMATV
DCTDQKSAWLCRRAHVRAFPSMLIYIYGNTYTRYIYNGPRDAAHILQFLDLFYRRLEPDMDFAEETFLSE
GTGALAHHQGLNQENLQSLNANNPEKNVKLPVGSVEGCEVSGSLNVNRVPSRLVFTARSKDLSFDLRGIN
VTHVVHHLSFGQVTRKQSTKSTQLSMSFDHFPLDGKTFRTENENITVEHFLSVIGVDHMEAKSKHMGLVE
RTYQIVARSNQYNATDMLPAALFTFDISPLVIQMSSDSTPFYRFLTSLCAIVGGMVTIIGFVDAGAYHAM
NSIKRKRQLGKLN

NCBI Reference Sequence: XP_008868410.1
>A.invadansPDI-C1
MGAMDVLKKWDFYKKVPEDLTVSTLPGVSLSIIGCVIMFILFVLEFNAYLTVAHDYQIVMDEGLDEMLRI
NFNITVPDLRCEFATVDVSDQTGTRKHNMTTDIYKIRIDSKRRILGMSAEEQPKPQYADDSIYGDLPESD
AVVTVLTPNTFESFLKEHHYVAVDFFAPWCIWCQRLEPIFVRTAKSLPSLHYGQRMRVASVDCQQYGELC
ESQFIRAYPTIMFYKDGDISPVEMYHGDRTLEAFLAKFKSLFDGEQDFSENRKKQLHEEDKKEAASKGVV
IAKAPGAEGCQLYGHLMVKRVPGNFHVHLQNPSYSMGVVNASHVVGELWFGEPVAYDQMNRLPKDAHAHL
YAHRLEGREFISYATEHTYVHYIKVVTNSYVQSNGDVLNVYKYTAHSNEYKEENDMPSIMFRYDLSPMSV
KISEKSVPFYHFLTSACAIIGGVFTVIGLLDQIIHQTVRALNKKVL

NCBI Reference Sequence: XP_009040032.1
>A.anophagefferensPDI-C1
-MDFYRKVPDELKEASRTGGLLSLCACGVVALTLVTEIGAFLRTEVRTKIDVDTFAGSQLRVNFNLSFPH
LHCDYASVDLWDKIGRNQANVTQNIEKWQLDEDGVKRMYQGRNRRAFDIDHDVHHPPIEEMHANGVHVWH
VNADEWEGLHEHHEYVFVDFYAPWCLYCQSLRSTWEALAEELEKRDLGVAVAAVDCVDNEAFCHDMKVQT
FPTLRFYHHGEQVNEGEYRFDRTVAALTDFVTRKLESENIYRQYPEARVAHAANWNTDHPGCLVSGFLLV
NRVPGNFHVMAHSRHHSLNTLRTNLSHTVHHLSFGVPLTDAQHRKLATIDVRHARTDTLDGEDYYHDDYH
YAYQHFVHIVPTKYNLGVFWRDRFAAFQTLHSHHLLKYAEHVPPEARFSYDISPMAVVVDTVRVKWYDFL
TSLLAIVGGTFALFKLANDTAARLF

NCBI Reference Sequence: XP_009040624.1
>A.anophagefferensPDI-C2
MSGLKVIDFFRKIPADLTEATVVGATLSIAAGVFMAILFVVELWAFLSTTIETGVMLDTNAETLLRINFNV
TMLDLRCDYAAVDVVDVLGTNSMNVTKNVEKWQLDETGKRMIFQGRNREQKAIQHDEHHPEIEELHKNGVH
AMPLDETNFESFLSENPYVFVNFFAPWCIWCQRLEPTWEAMAEEVERLNREVEQVDERSTSIDVDVVKVDC
VANRNLCGTQRIQAFPTLRFFKDAQQYGIDYKQDRTVAAMVDFLKQKVELEKTMEDWHPRRKQRMLEIKEH
PGCMVSGHVLVNRVPGNFHIEARSIHHNLNAAMTNLSHVVNHLSFGTPLAKDMQRKVSKYPQFQSVHPLDG
GIFVSRDYHQVHHHYSKVVSTHFEVGGMMTKSREIVGYQMLAQSQIMHYNEMDVPEAKFSYDLSPMAVLVS
SKGRRWYDFVTSVCAIIGGTFTVVGIVDAVLYKIIKGGKQL

NCBI Reference Sequence: XP_009040131.1
>A.anophagefferensPDI-C3
MSGLKMVDFFRKIPADLTEATMVGATLSITAGVFMAILFLVELWAFLSTTIETGVMLDTNAETLLRINFNV
TMLDLQCDYAAVDVVDVLGTNSMNVTKNVEKWQLDETGKRMIFQGRNREQKAIQHDAHHPEIQELHKNGVH
AVPLDEANFDMYLSENPYVFVNFFAPWCIWCQRLEPTWEATAEEVERLNENENESIDVDIVKVDCVANRNL
CGTQRIQAFPTLRFFKDSQQYGIDYKQDRTVSAMIDFLKQKVELEKTMEDWHPRRKQRMLEIKEHPGCMVS
GHVLVNRVPGNFHIEARSLHHNLNAAMTNLSHIVNHLSFGTPLARDLQRKVSKYPQFQSAHPLDGGSFINR
DYHQAHHHYSKVVSTHFEVGGMMTKTHEIIGYQMLSQSQVMHYNEMDVPEAKFSYDLSPMAVLVSSKGRRW
YDFVTSVCAIIGGTFTVVGLVDAVLYKIIKGGKQL

GenBank: CBN74236.1
>E.siliculosusPDI-C1
MPTIKTFDFYRKIPLDLTETTLQGAVMSGCALFCMLILFLCELRAFLTPEVYTTVAIDSNQDSKLRINFN
ITMLALPCDYASVDVLDLLGTNKVNMTQNIVKWHTDENGVKREFHGRNKAQEMVKHDDHHRDLDLAHEDG
EHAVPLTSSNFKDFIQGNDNVMVDFFAPWCVWCIKLAPTWEAFAEEVERDASLNGKLMVAKVDCVEEREL
CSTQNLMAFPTIRYFKGGVQDGTDYSKDRSVTSLVQYAQAKVGGDEKALRRYGRLKQDYPGCQLSGFIMV
NRVPGNFHIEARSALHSIDPTAANISHVVKTLKFGTQVPVRGRRVIESGVELEGLPALEDRVYSIDSLHT
APHHYIKVVSTFVGGLAKTDNLQYQMMVSSQTMPYEQDQVPEAKFSYDLSPMSVHIKQRRRKWYDFLTSV
LAIVGGTFTVVGVLDNILFRVVKQKKI

GenBank: CBN74492.1
>E.siliculosusPDI-C2

--MGAWRLLDLYPKIPTD
LSQSTAVGGWFSTLTGVIMLLLFQVELFSFMSAPIESQVVVDNVLETKLQINFNMSFLDLPCEYLSVDAL
DVLGSNRVNITGKEVQKWHLDPQGVKQAFHGRNRQQRDIVQFDEGVVASLQDLHEDGVHAAVLTEENFDT
WLLEHDFTFVAFHAPWCGWCQRLMPTLEVLAEVLEESQRGITVGTVDCTTQQNLCTKRFPVRAFPTLKLF
NEEKLLKPDYSGDRTVEAFSTYLYSKADGKPMKHQRKGRKGFPEVGLHDDKWPGCMVTGHIMVNRVPGNF
HIEAASKSHTFHGATTNLSHIVHHMSFGNDPPRRTQTKINRLTEDLRQNAPLDGNVYVANAYHQAPHHYL
RVVGSMYHLSPMKTPWHGYQIVANSQMMLYDEEEVPEARFSYNISPMSVLVRSEKRPWYDFVTKVLAIVG
GTFSMVGLVDAAVFRASRKAGRQLS

GenBank: EWM21843.1
>N.gaditanaPDI-C1
MSSKRPSARGMDLYRRVPADLTETSTLGGLFSIVAGVFMVALFIVELISFMSHRTETMVVLDHSHDQLIQ
INFNVTMLDLPCRYAVVDVLDVLGTNRMNVSKNIEKWNLDEAGRRRYFQGRNREERSILHDEHHGALEVL
LQNGEHAVPLAADSFDEYLESNEFTFVSFYAPWCIWCQRLAPTWEAFAEHVEEAQIPVKVAKIDCVTHAQ
LCRDQKVHAFPTLRMFNRARPLPPDYNTDRTLVALTSFVTRKLAVEEKKKSWPERDQEYVEHPGCMLSGH
LLVNRVPGNFHIEARSAVHNLNAAMTNLSHVVNHLSFGPLYDSQVLSRLERFSSEFYVSNALDGRDFVLE
EPHKAHHHYLKVVGTLYEVPMRWGRADMLTYQMLPETSLMTYGLDEVPEAKFSYEVSPMAVIVKKTGRRW
YEFVTSLCGILGGTFTVVGLLDSTLHRLLKPSSNLQS

GenBank: EWM21113.1
>N.gaditanaPDI-C2
MAPVSSRGIKSFDFYRKVPLDLTEATWHGGLVSILAILFMLSLFVVELSSFIASTTHSTVVVDFNRDQQI
RINFNITMLDVPCEFATVDVLDVLGTNQQNVTKNIEKWNLDQNAQRRMFQGRNREQRDLVHDDHHPALDL
AHQDGEHALPLGKDDFAGFVAGQEYVFANFYAPWCVWCQRLAPTWEAFAEALERQQFNIKVVKIDCVEHR
DLCAESVIRAFPTLRLYKAGKAISPDYREDRTVEALTSYIERTLDLHAKVASSAPEHREKIERTLFAEAE
HPGCLLSGFLLVNRVPGNFHIEARSKYHNLNPTLTNVSHVVHDLTFGPPVTREYREKLALLPKGFQQTRS
PLADQVYVVSKVHHAFHHYLKVVSTHYEVSRTFGGQKSTVLQYQMVANSQVMHYQDDEVPEAKFSYDISP
LATVISSKKRAWYEFLTSLMAIIGGTFTVLGLLDHMLGKVLKPKKM

NCBI Reference Sequence: XP_002182871.1
>P.tricornutumPDI-C1
MSSVDFYRRVPKDLTEATSLGAIMSVCALVVMGVLFLSETAAFARTGIATSITLDENTSPQIRLNFNITL
TDLQCDYVSIDVWDALGTNKQNVTKNIDKWQLDAQGIRRIFSGRNREGREVVHDSHDRSLDEIHSEDGKA
VVDLTADTFDDFMEEHEMAFVDLYAPWCVWCQRLAPTWELFAQEVKKEGMPIGVAKIDCMAEADLCRAQR
VMAFPTLRWYHEGKAVAPDYKMDRTIPALTSFAKRKLDMDEKFKEWHSKASDSADPAEVEKKRQLYQQNR
PDHPGCQVSGHLMVNRVPGNFHLEAKSKSHNLNAAMTNLSHVVNHLSFGEPIDENNRKSKRILKQVPEEH
RQFAPMDGQAFLTKAFHQAFHHYIKVVSTHLNMGSSDANSMLTYQFLEQSQIVFYDDVNVPEARFSYDLS
PMSVVVEKEGRKWYDYLTSLCAIIGGTFTTLGLIDATLYKVLKPKKL

NCBI Reference Sequence: XP_002177433.1

>P.tricornutumPDI-C2
MVAPAWAGRVDMYRKVPADLMEGSKSGSYFSYFAVAGMLLLFLMESSEFFAKKLVTDLALDSNKDPRVRL
NFNITLMDLRCEYAVVDVVSVLGTEQNVSSHITKWGVDAEGVRKRYAGRNKDQKDIKMFDSSVTSTIEEL
YSDGEDAVSLDEETLEYALRDQQYLFVDFYASWCSHCRALAPTWETLAEVMSDVAEDLVEQHDHEYSEEE
YEHAKKVEMPVMIAKIDCVLHKQVCMKQGILGYPTLRLFVDGERWKGGDYRGDRTVVAFADWLQQVEDAH
KTDTENSSAKNVQLAHMAAKDRLDSEDEGSDEEHEWAEKVKRHKQRLHHSWVDAEHPGCNIAGHLLLDRV
PGNFHIQARSPHHDLVPHMTNVSHVVHHLSIGEPVAERLIEQEKVILPEDVKRKLKPMNGNAYVTKELHE
AYHHYLKVITTNVDGLKFGKRDLRAYQILQSSQLSFYRNDIIPEAKFVFDLSPVAVSYRTTSRRWYDYFT
SILAIIGGTFTVVGLLESTIHATVARKRRY

NCBI Reference Sequence: XP_002899237.1
>P.infestansPDI-C1
MGAVDVLKKWDFYKKIPEDLTVSTLPGVSLSIAGCFIMFLLFILEFNSYLTVDYKYDIVMDEGLDQTMRI
NFNITVPDLPCEFASVDVSDMTGTRKHNMTSDIFKIRLDQKGRMVGLADETQVMPRFAEDTEYGDLPDSD
ATVTILDEESFEPFLKQHHYVAVDFYAPWCVWCKRLEPVWTRVAKTLPSLHYGQQLRVASVDCQAHPQLC
MKQFIRAYPSILFYKDGDVSPVEMYFGDRTVEAFVDKFKQLMAGEMDVVEARKKELFEQDKKDAREQGRA
IARSAVGPEGCRLFGHLYVKRVPGNFHVHLANPAYSMDSSLVNASHTVNELWFGEHLAPGDMSRLPREAQ
TQLYTHRLENQDFTSLYKNHTYVHYIKVVTNSYVQGDGSEINVYKYTAHSNEYLETDDLPSVMFRYDLSP
MSVRISEDTVPFYHFVTSACAIIGGVFTVIGIVDQIIHQTARALNKKVL

NCBI Reference Sequence: XP_002899938.1
>P.infestansPDI-C2
MAKFDFFRHVPEDLKVHTYSGSFFSLLSFAIMGLLLITHWEAYRDQSTKTTVVMDEHQEDRLRVNFNVSL
LNVPCSVASVDLEDHMGQRFTNLTRHIRHFRLGADRSSNEVQRLDEVVIDNHEKGIPVWGGVHRDTKGAN
VHYSTPLTTKNFDDFMAKYELVLVNFYAPWCPFCHQLHPEWERAAAQLPDHPEYSEMVRMASVDCTDPDA
VWLCRRAHIRAFPSMLIYMYGSTSTRYIYNGPRKTEHLLQFLDLFFRRLEPDADFAEEVNVNNDRLGLPL
PVRVSQENLEGIDFKKRRPSSTIQTGAVEGCEISGSISVNRVPGVLVFTARSDDVSFNAQAIDVSHVVNH
FSFGQVRRTENLLSGDNHVLAAPSNRFPLDRKIYTIENENVTVQHFMNVVGFDNQDNTRKTHLQQRSYEFS
ASTTQYEDQTPSALFTFDISPLVVQITTDNIPFYHFITHLCAVIGGVFTILSLVDSGVFHAMNSIKKKQQL
GKLS

GenBank: ETI48787.1
>P.parasiticaPDI-C1
MGAMDVLKKWDFYKKIPEDLTVSTLPGVSLSIAGCFIMFLLFILEFNSYLTVDYKYDIVMDEGLDQTMRI
NFNITVPDLPCEFASVDVSDMTGTRKHNMTSDIFKIRLDQKGRMVGLAEETQVMPRFAEDTEYGDLPESD
ATVTILDEESFEPFLKEHHYVAVDFYAPWCVWCKRLEPVWTRVAKTLPSLHYGQQLRVASVDCQAHPQLC
MKQFIRAYPTILFYKDGDVSPVEMYFGDRTVEAFVDKFKQLMAGEMDAVEARKKELFEQDKKDASEQGRA
IARSAVGPEGCRLYGHLYVKRVPGNFHIHLANPAYSMDSSLVNASHTVSELWFGEHLAPGDMSRLPRDAQ
TQLYTHRLDNQDFTSFYKNHTYVHYIKVVTNSYVQSDGSEINVYKYTAHSNEYLETDDLPSIMFRYDLSP
MSVRISEDSVPFYHFVTSACAIIGGVFTVIGIVDQIIHQTARALNKKVL

GenBank: ETI36765.1
>P.parasiticaPDI-C2
MAKFDFFRHVPEDLKVHTYSGSFFSLLSFAIMGLLLITHWEAYRYQSTKTTVVMDEHQEDRLRVNFNVSL
LNVPCSVASVDLEDHMGQRFTNLTRHIRHFRLAADRSSNQVERLDEVVIDNHEKGIPVWGGVHRDTKGAN
VHYSTPLTTKNFDDFMAKYELVLVNFYAPWCPFCHQLHPEWERAAAQLPDHPEYSEMVRMASVDCTNPEA
VWLCRRAHIRAFPSMLIYMYGSTSTRYIYNGPRKTEHLLQFLDLFFRRLEPDADFAEEVNVNNDRLGLPL
PVRVSQENLEGIDFKKRRPSSTIQTGAVEGCEISGSISVNRVPGGLVFTARSDDVSFNAQAIDVSHVVNH
FSFGQVRRTENLFKGGSHMLAAPSNRFPLDSKMYTIESENVTVQHFLNVVGFDNQDNSRKTHLQQRSYEF
SATTTQYEDKTPSALFTFDISPLVVQITTDNIPFYHFITHLCAVIGGVFTILGLVDSGVFHTMNSIKKKQ
QLGKLS

NCBI Reference Sequence: XP_008607386.1
>S.diclinaPDI-C1
MGAMDTLKKWDFYKKIPEDLTVSTLPGVSLSLVGCFIMFVLFILEFNSYLSVNYRYDIVMDEGLDEMMRI
NFNITVPDLRCEFASIDVSDMTGTRKHNMTKDIYKIRIDSKRRILGMSAEEQPMPAYADDSVYGDLPESD
AVVTMLDEATFEPFLKEHHYVAVDFYAPWCIWCQRLEPVWVRTAKTLPTLHMGQRMRVASVDCQAHPELC

MTQFIRAYPTIMFYKDGDLSPVEMYHGDRSVEAFTEKFRTLFEGEADYAEQRKKEMHESDKKEAATKGEL
IAKAPGAEGCQLYGHLYVKRVPGNFHVHLQNPAYSMDASLVNASHTVGELWFGEPVPYNDLVRLPKDAHA
KLYSHRLEGKEFTAYHKEHTYVHYIKVVTNSYVQSNDDVINVYKYTAHSNEYEEKDDLPSIMFRYDLSPM
SVKISEDSVPFYHFLTSACAIIGGVFTVIGILDQIIHQTVRAMNKKVL

NCBI Reference Sequence: XP_008604596.1
>S.diclinaPDI-C2
MAPRFDFFRKIPEDLQTSSGTSTFFTITTVVVMFYLIVAEYAAYLASSSRTVSRVVMDSHQEDLLRINFN
ISLTSIPCHHVSVDVSDHMGQRFANITRHIRRFELTDEGGSTVRGEEVLMADPSSVQRWGAVTHELHDGE
TVTPSLNDATFDEFMAKYELVLVNYYAPWCPFSQALLPIWEQTALQLQDHPEYSERVTMARVDCTQDNAV
SLCRRARIHAFPSMMIYMYGHTFTRYIYNGPRNAESLLLFLDLFYRRLNPDGDFAEEVIPEFGDLLRLAA
PQNEEHDHDHEGCELSGSIAVQRVPGKLVLYPYSGDQSFDMRDINVTHTINHFSFGQWKSTEQRMNNPRA
VLSTHYPMDAKHYRALDSNITIEHYIKIVGVDHMDTISFLHQIPERMYEFSASSNQYNATNQVPAALFTY
DSSPLVIELYTQSMPFFRFLTSLCAIVGGVYTVLGLVDAGVFHAVTSVQRKAKLGKLI

NCBI Reference Sequence: XP_012193854.1
>S.parasiticaPDI-C1
MGAMDTLKKWDFYKKIPEDLTVSTLPGVSLSLVGCFIMFVLFILEFNSYLSVNYRYDIVMDEGLDEMMRI
NFNITVPDLRCEFASIDVSDMTGTRKHNMTKDIYKIRIDSKRRILGMSAEEQPMPVYADDSVYGDLPESD
AVVTMLDEATFEPFLKEHHYVAVDFYAPWCIWCQRLEPVWVRTAKTLPTLHMGQRMRVASVDCQAHPELC
MTQFIRAYPTIMFYKDGDLSPVEMYHGDRSVEAFTEKFRSLFEGEADYAEQRKKEMHESDKKEAATKGEL
IAKAPGAEGCQLYGHLYVKRVPGNFHVHLQNPAYSMDASLVNASHTVGELWFGEPVPYNDLVRLPKDAHA
KLYSHRLEGKEFTAYHKEHTYVHYIKVVTNAYVQSNDDVINVYKYTAHSNEYEEKDDLPSIMFRYDLSPM
SVKITEDSVPFYHFLTSACAIIGGVFTVIGILDQIIHQTVRAMNKKVL

NCBI Reference Sequence: XP_012197035.1
>S.parasiticaPDI-C2
MAPRFDFFRKIPEDLQTSSGTSAFFTITTVVVMLYLIVAEYAAYLASSSRTVSRVVMDSHQEDLLRINFN
ISLTSIPCHHVSVDVSDHMGQRFANITRHIRRFELTDEGGSTVRGEEVLMADPNSVQRWGAVTHELHDGE
TITPSLNDATFDEFMAKYELVLVNYYAPWCPFSQALLPIWEQTALQLQDHPEYSERVTMARVDCTQDNAV
SLCRRARIHAFPSMMIYMYGHTFTRYIYNGPRNAESLLLFLDLFYRRLNPDGDFAEEVIPEFGDLLRLAA
PQNEDHDHDHEGCELSGSIAVQRVPGKLVFYPYSGDQSFDMRDINVTHTINHFSFGQWKSTEQRMNNPRA
VLSTHYPMDAKHYRALDSNITIEHYIKIVGVDHMDTISFLHQIPERMYEFSASSNQYNATNQVPAALFTY
DSSPLVIELYTQSMPFFRFLTSLCAIVGGVYTVLGLVDAGVFHAVTSVQRKAKLGKLI

NCBI Reference Sequence: XP_002287517.1
>T.pseudonanaPDI-C1
MAPFIANLDMYRKVPVDLLEGTRRGSILSTIAIFTMTTLFFLETKAYFSSTLATSLALDSNSDPNIRVNF
NITMMDLKCDYATIDVVSVLGTQQNVTQHVQKFPIDQYGVRQRMKARNKNQHDVQLFDSTIQETIEELHA
DGEDAISLDETTLNVALQENTYVFVDFFANWCSHCRDLAPTWETLAEVMYEAAESRVEEHLERNPDHHYS
DEDYEEAVKVQLPVMIGKIDCVDHGGLCQRMQIQAYPTLKFFVDGEEMGDYRGHRTVIEMAHFIAEMEKT
HKGKGGVAQDATEIASDRTIQTKEHEDYARALSASRHHIKHSWHDEDHPGCQISGFLLVDRAPGNFHIQA
QSKGHDLAAHMTNVSHIINHLSFGKPFSKYFLKDGLKNTPPGFLETTKPFDGNVYITQNEHEAHHHYLKV
ITTEFEPEKGAQNSKYNKKEPSRAYQILQSSQLSLYRSDIVPEAKFTYDLSPIAVSYNKKYRHWYDYFTS
LMAIIGGTFTVVGMLESGIHSVSSKKRR

NCBI Reference Sequence: XP_002295231.1
>T.pseudonanaPDI-C2
MNNISGPGRRSAMSSVDFYRRVPKDLTEATSLGAIMSICAITVMAILFFSETLAFARTAMVTSIALDEND
QPQIRLNFNITLMDLHCDFVSVDVWDTLGTNRQNVTKNIEKWQLDEDGQRRIFSGRNREQREVVHEEHEE
TLEELHEDGEQAVELHPENFKAFLEGHDMAFIDMYAPWCIWCQRLHPTWEKFGEKVHELGMPVGVGKVDC
VVHAQLCKDEKVMAFPTLRWYKDGEAILPDYKMDRTVDALVGYAKRKLDMEQKYKDWESKNAGGNADARG
KPRGGTSRPEHPGCQVSGHLMVNRVPGNFHIEAKSVNHNLNAAMTNLTHRVNHLSFGEPITKLPPHMENT
PFMRKVKRVLKQVPEEHKQFNPMDDTEYVTAQFHQAFHHYIKVVSTHLNMGSSSKSEYSVNDVNAVTVYQ
MLEQSQIVFYDEVNVPEARFSYDMSPMSVVVQKEGRKWYDYLTSLCAIIGGTFTTLGLIDATLYKVFKPK
KL

NCBI Reference Sequence: XP_002295232.1

```
>T.pseudonanaPDI-C3
MYGDINGGGSRRRAVGTADLYRHVPKEITE------------------------ATKIGVVMSLLSIFIM
ILLFFCETYAFSRSTISSTIAVDPNSEQLLRLNFNVTLYDLHCDYASVDIWDTLGTNQQNITKDIVKWNL
DDQGQRKKFAGRNAEQRAVTHEEHDETLQDLADALGGELHAVALDPESIVEFHKRHNGQAIIDFYAPWCI
WCQRLEPTWEKFARQVSDERINLGVGKVDCVTHAQLCKDQRVMAFPTLRWFENGKAVMPDYRGDRTVDAL
VDYAKRRVGSNEGSNDEEFEEDHHPGCLISGHLMVNRVPGRFQIEARSVNHELHSAMTNLTHRVHDLTFG
ALSGPPGHMLHVLPFFDTVPEKYKHTNPMQDKYYPTYEFHQAFHHHLKIISTHIDYLFSRSTVLYQILEQ
SQLVFYEEVNVPEIQFSFDLSPMSVNVSKEGRKWYEYVTSLCAIIGGTYTTLGLINATLLRIFKPKKL
```
