# Supplementary material for: Phylogenetic characterization and promoter expression analysis of a novel hybrid protein disulfide isomerase/cargo receptor subfamily unique to plants and chromalveolates
Source: Mol Genet Genomics. 2015 Aug 25;291:455–69. doi: 10.1007/s00438-015-1106-7 (PMC4729789; doi:10.1007/s00438-015-1106-7)
Supplement: Supplementary file 3 — Online Resource 3. Multiple sequence alignments used for phylogenetic analyses. Alignments were generated by the MUSCLE multiple sequence alignment program, and visually inspected and edited (if necessary) using the Alignment Explorer sequence editor of MEGA6. The positions of blocks of conserved sequence identified by Gblocks are indicated by red bars above the alignment (PDF 883 kb) [file 438_2015_1106_MOESM3_ESM.pdf]

Multiple sequence alignment for NJ and ML trees in Fig. 1

```
                                         BLOCK#1------------------------------------------
O.sativaPDI-C               1 -----MISSSKLKSVDFYRKIPRDLTEASLSGAGLSIVAALAMVFLFGMELSNYLAVNTS
Z.maysPDI-C                 1 -----MISSSKLKSVDFYRKIPRDLTEASLSGAGLSIVAALAMVFLFGMELSSYLAVNTT
A.thalianaPDI13             1 -----MVSTSKIKSVDFYRKIPRDLTEASLSGAGLSIVAALAMLFLFGMELSSYLAINTS
A.thalianaPDI12             1 -----MVSSTKLKSVDFYRKIPRDLTEASLSGAGLSIVAALFMMFLFGMELSSYLEVNTT
A.thalianaPDI7              1 -----MVSTSKIKSVDFYRKIPRDLTEASLSGAGLSIIAALSMIFLFGMELNNYLAVSTS
P.trichocarpaPDI-C1         1 -----MVSTNKLKSVDFYRKIPRDLTEASLSGAGLSIVAALAMMFLFGMELNNYLTVNTS
P.trichocarpaPDI-C2         1 -----MVSTNKLKSVDFYRKIPRDLTEASLSGAGLSIVAALAMVFLFGMELNNYLTVNTS
P.patensPDI-C               1 -----MVSTSKLKSIDFYRKIPRDLTEASLSGAGLSIIAALTMVFLFGMELSAYLSTTTS
S.moellendorffiiPDI-C       1 -----MTTASKIKSIDFYRKIPRDLTEASLSGAGLSLIAAFAMIFLFGMELNNYLTVSST
C.subellipsoideaPDI-C       1 ----MARVLQKLRSVDFYRKIPNDLTEATLAGAGISLVAAFTIVVLLTAELSSFLAIETK
C.reinhardtiiPDI-C          1 ----MVRLFSRLKAIDFFKKIPSDLTEATLTGAWLSIVAAVLMILLFVAELSAFLSTTTS
V.carteriPDI-C              1 ----MARLFSKLKAIDFFKKIPSDLTEATLTGAWISILAAVIMVFLFTAEMMSFLSTTTT
C.reinhardtiiERV-A          1 --MSGGGFLGKLKALDAYPKINEDFFTKTMSGGIITIVSSVVMVLLFLSELRLFLTTSSA
V.carteriERV-A              1 MSNSGGGFLSKLKALDAYPKINEDFFTKTMSGGIITIVASVVMVLLFLSELRLYMTTQSV
C.subellipsoideaERV-A       1 ----MEGIVSKLKNLDAYPKVNEDFFQRTLSGGIITIGSSIIMLCLFLSELSLFMKITTT
O.sativaERV-A3              1 ----MEGFLQKLKGLDAYPKVNEDFYKRTLSGGVVTVVASVVMLLLFVSETRSYFYSATE
Z.maysERV-A2                1 ----MDAFLQRLKRLDAYPKVNEDFYKRTLSGGIVTLVAAVVMLLLFISETRSYFYSATE
Z.maysERV-A3                1 ----MDAFLHRLKRLDAYPKVNEDFYKRTLSGGIVTLVAAVVMLLLFISETRSYFYSSTE
S.moellendorffiiERV-A       1 -----MQMLKKLQQLDAYPKINEDFHSRTLSGGVITVVSSIFMAILFITELKLFLLPGTT
P.patensERV-A1              1 ---MALQMIQKLKSLDAYPKINEDFYSRTLSGGIITIISATFMVLLFFSELKLYLAAQVA
P.patensERV-A4              1 -----MSFFNKLKHLDAYPKISEDFYSRTLSGGLITLVSSVFMTLLFITEFRIYLSAQTQ
P.patensERV-A2              1 -----MAVFNKLKQLDAYPKISEDFYSRTLSGGVITLVSTVFMFVLFVTEISLYLSAQTQ
P.patensERV-A3              1 -----MAIFNKLKQLDAHPKISEDFYSRTLSGGVITLVSSIFMFLLFVTEFRIYLSAQTQ
O.sativaERV-A2              1 -----MDLWNKLRSLDAYPKVNEDFYSRTLSGGLITIASSLAILLLFLSEIRLYLYSATD
P.trichocarpaERV-A3         1 ----MEGIYQKLRNLDAYPKINEDFYSRTLSGGLITLISSIIMLFLFFSEFSLYLHAVTE
P.trichocarpaERV-A4         1 ----MDRIYQKVRNLDAYPKINEDFYSRTLSGGLITLISSVLILFLFFSELSLYLHKVTE
A.thalianaERV-A2            1 ----MVGVMNRLRNLDAYPKINEDFYRRTLSGGVITLASSIVMLILFFSELQLYIHPVTE
O.sativaERV-A1              1 ----MEGLLSKLRSLDAYPKVNEDFYSRTLSGGIITLASSVVMLLLFVSELRLYLHAVTE
Z.maysERV-A1                1 ----MDGLLSKLRSLDAYPKVNEDFYSRTLSGGIITLVSSAVMLLLFVSELRLYLHAVTE
A.thalianaERV-A1            1 ----MAGILNKLRNLDAYPKINEDFYSRTLSGGVITLLSSVVMFLLFFSELRLYLHTVTE
P.trichocarpaERV-A1         1 ----MEGLMSKLRNLDAYPKINEDFYSRTLSGGVITLASSVVMFLLFFSELRLYLHAVTE
P.trichocarpaERV-A2         1 ----MDGLMSKLRNFDAYPKINEDFYSRTLSGGVITLASSIVMFLLFFSELRLYLHAVTE
C.reinhardtiiERV-B          1 -----MGKGFRLSSLSAYVKPEAHLVNQTIHGALVTLCGVLLATLLTLHEIKSFYQMHRV
V.carteriERV-B              1 -------MKFKLSSLSAYVKPEAHLVQQTVHGALVTLCGILLAAMLFVHELGSFYRQHRV
C.subellipsoideaERV-B       1 ------MKLKSFNRFSAYARAESHLVQRTYFGAIVTVLGVILAIVLFANELREYTTPFSI
P.patensERV-B               1 ---MRKEKWQVIKNLDAFPRAEEHLLQKTSSGAAVSAIGLFIMGVLFFHELRFYLETVTV
S.moellendorffiiERV-B       1 -----MGL--KMKNINAFAHADEHLTQKTVSGAILTIVGVSIILVLFAYEFKFYLSTNVV
O.sativaERV-B               1 -----MGRIPSLKNFNAFPHAEDHLLKKTYSGAIVTIFGLIIMVTLFAHELKFYLTTYTV
Z.maysERV-B                 1 -----MARIPSLKSLNAFPHAEEHLLKKTYSGAVVTIFGLLIMITLFVHELQFYLTTYTV
A.thalianaERV-B             1 -----MGVKQALRSIDAFPRAEDHLLQKTQSGAVVSIVGLLIMATLFLHELSYYLNTLTV
P.trichocarpaERV-B1         1 -----MGMKQAIKKLDAFPRAEEHLLQKTQSGALVSIIGLVTMATLFYHELAYYLTTYTV
P.trichocarpaERV-B2         1 -----MGVKQAIKSLDAFPRAEEHLLQKTQSGALVSVIGLVIMATLFYHELAYYLTTYTV
                                        ERGIC-N-------------------------------------------
```

```
                                 --------> BLOCK#2-------------------------->
O.sativaPDI-C              56 TSVIVDRSSDGEFLRIDFNLSFPALSCEFASVDVSDVLGTNRLN----ITKTVRKYSI--
Z.maysPDI-C                56 TSVIVDRSSDGEFLRIDFNMSFPALSCEFASVDVSDVLGTNRLN----ITKTVRKYSI--
A.thalianaPDI13            56 TSVIVDKSSDGDFLNIDFNISFPALSCEFASVDVSDVFGTHRLN----ISKTIRKVPI--
A.thalianaPDI12            56 TAVIVDKSSDGDFLRIDFNISFPALSCEFASVDVSDVLGTNRLN----ITKTVRKFPI--
A.thalianaPDI7             56 TSVIVDRSADGDFLRLDFNISFPSLSCEFASVDVSDVLGTNRLN----VTKTIRKFSI--
P.trichocarpaPDI-C1        56 TTVIVDNSSDGEFLRIDFNISFPSLSCEFASVDVSDVLGTNRLN----ITKTIRKFSI--
P.trichocarpaPDI-C2        56 TSVIVDNSSDGEFLRIDFNLSFPSLSCEFASVDVSDVLGTNRLN----ITKTIRKFSI--
P.patensPDI-C              56 TSVVVDRSRDGEYLRIDFNLSFPALSCEFASVDVSDVLGTHRFN----LTKTVRKYPI--
S.moellendorffiiPDI-C      56 TNVVVDRSKDGEYLRIQFNMSFPALSCEFASVDVSDALGTNRYN----LTKTVRKYPI--
C.subellipsoideaPDI-C      57 EELIVDRSAHGDLLRINFNISFPSLSCEFATLDVSDALGTKRMN----LTKTIRKLPI--
C.reinhardtiiPDI-C         57 SQLVVDRSPQNELLKLNFNISFPALSCEFATVDVSDSLGTKRMN----LTKTVRKVPITL
V.carteriPDI-C             57 TQLIVDRSPQNELLKLNFNISFPALSCEFATVDVSDTLGTKRMN----LTKTVRKMPITT
C.reinhardtiiERV-A         59 HELSVDVGR-GEKIKIHFDVTFPKVPCAWLSLDAMDISGELHLD----LDHDVYKQRL--
V.carteriERV-A             61 HELSVDVGR-GEKIQIHFDLTFPKVPCSWLSLDAMDISGELHLD----LDHDVYKQRL--
C.subellipsoideaERV-A      57 NELSVDTTR-GDQLSINFDMTFPALPCEWISLDLMDISGEMHLD----VDHDVYKRRL--
O.sativaERV-A3             57 TKLVVDTSR-GERLRVNFDVTFPSVPCTLLSVDTMDISGEQHHD----IRHDIEKRRL--
Z.maysERV-A2               57 TKLVVDTSR-GERLRVNFDITFLSIPCTLLSVDTMDISGEQHQD----IRHDIEKIRL--
Z.maysERV-A3               57 TKLVVDTSR-GERLRVNFDITFPSIPCTLLSVDTTDISGEQHHD----IRHDIEKRRL--
S.moellendorffiiERV-A      56 SELLVDTSR-GETLQINFDITFPALACSVISLDAMDVSGEQHLD----VKHNIFKKRL--
P.patensERV-A1             58 NDLVVDTER-GGTIQINLDVTFPALACSVVSLDAMDISGEAHLD----VKHNIFKKRL--
P.patensERV-A4             56 NQLVVDTSR-GETLQINLDITFSALACSVVSLDAMDISGEQHLN----VRHNIFKKRL--
P.patensERV-A2             56 NQLVVDTSR-GETLQINLDITFPALACSMVSLDAMDISGEQHLN----VRHNIFKKRL--
P.patensERV-A3             56 NQLVVDTSR-GETLQINLDITFPALACSVVSLDAMDISGELHLD----VRHNIYKKRL--
O.sativaERV-A2             56 SKLTVDTSR-GERLHINFDVTFPALPCSLVAVDTMDVSGEQHYD----IRHDIIKKRI--
P.trichocarpaERV-A3        57 TKLLVDTTR-GQTLRINFDITFPAIRCSLLSVDAIDISGEQHHD----IRHDITKKRI--
P.trichocarpaERV-A4        57 TKLLVDTSR-GQSLRINFDVTFPAIRCSLLSVDAIDISGEQHLD----IRHDISKKRI--
A.thalianaERV-A2           57 TQLRVDTSR-GEKLRINFDVTFPALQCSIISLDSMDISGERHLD----VRHDIIKRRL--
O.sativaERV-A1             57 TTLRVDTSR-GETLRINFDVTFPALQCSIISLDAMDISGQEHLD----VKHDIFKQRI--
Z.maysERV-A1               57 TTLRVDTSR-GETLRINFDVTFPALQCSIISLDAMDISGQEHLD----VKHDVFKQRI--
A.thalianaERV-A1           57 TKLIVDTSR-GETLRINFDITFPALACSILSVDAMDISGELHLD----VKHDIIKRRL--
P.trichocarpaERV-A1        57 TKLVVDTSR-GETLRINFDVTFPALPCSILSLDAMDISGEQHLD----VKHDIIKKRL--
P.trichocarpaERV-A2        57 TKLVVDTSR-GETLRINFDVTFPALPCSILSLDAMDISGEQHLD----VKHDIIKKRL--
C.reinhardtiiERV-B         56 TVLSVDLAR-RHALTINLDITFPSVPCAVLSIDVLDISGTAENDASFAHHMRVHKMRL--
V.carteriERV-B             54 TQMSVDLAR-RNALTINIDLTFPAIPCAVLSIDVLDIAGTAENDASYAHHMHIHKLRL--
C.subellipsoideaERV-B      55 QTMSVDTSR-AHYIRMNFNFTYPSMPCQVLSLDATDMSGEKSGDSGHAANGEIHKVRL--
P.patensERV-B              58 HEMSVDVKR-GEKLPIHINMTFPALPCEVLSLDAIDMSGKHEVD----LDTNIWKLRI--
S.moellendorffiiERV-B      54 HQMSVDTTR-GQNLPIHINITFPSLPCQILSVDAIDMSGKHEVD----LDTNIWKLRL--
O.sativaERV-B              56 HQMSVDLKR-GETLPIHINMSFPSLPCEVLSVDAIDMSGKHEVD----LHTNIWKLRL--
Z.maysERV-B                56 HQMSVDLKR-GETLPIHINMSFPSLPCEVLSVDAIDMSGKHEVD----LHTNIWKLRL--
A.thalianaERV-B            56 HQMSVDLKR-GETLPIHVNMTFPSLPCDVLSVDAIDMSGKHEVD----LDTNIWKLRL--
P.trichocarpaERV-B1        56 HQMSVDLTR-GETLPIHINITFPSLPCDVLSVDAIDMSGKHEVD----LDTSIWKLRL--
P.trichocarpaERV-B2        56 HQMSVDLQR-GEILPIHVNITFPSLPCDVLSVDAIDMSGKHEVD----LDTNIWKLRL--
                              ------------------------------------------------->
```

```
O.sativaPDI-C            110 --DRNLVPTG-SEFHPGPIPTVSKHGDDVEEN-----HDDGSVPLSSRNFDSYSHQYPVL
Z.maysPDI-C              110 --DRNLVPTG-SEFHPGPIPILNKHGDDVEED-----HVDGAFSLSSRNFDSFSHQYPVL
A.thalianaPDI13          110 --DPHLRATA-EEFHSTSDLHLINHGDEDHGD----NSTYADIPLTGAAFEKFTHHFQIL
A.thalianaPDI12          110 --DPHLRSTG-AEFHSGLALHNINHGEETKEE-----FPDGAIPLTSASFEALSHHFPIL
A.thalianaPDI7           110 --DSNMRPTG-SEFHAGEVLSLINHGDETGEE-----IVEDSVPLTGRNFDTFTHQFPIL
P.trichocarpaPDI-C1      110 --DHDLKPTG-SEFHSGPVLHQIKHGDEVDEE-----GGEGSVSLKAHNFDQYSHQYPIL
P.trichocarpaPDI-C2      110 --DHDLKPTG-SEFHSGPVLHHINHGDEVHEE-----GSEGSVSLKAHNFDQYTHQYPIL
P.patensPDI-C            110 --DPLLQRIG-QEFHAGSVPNIKSHGDEDVGEDMFEHLGEGAVELNKNTFDVYAQQFSVL
S.moellendorffiiPDI-C    110 --DPNLKIVG-PEFHPGPIPNPTSHGDDDHEG-------EGAHVLTSSTFDEYARRYSVL
C.subellipsoideaPDI-C    111 --DEDGQRAG-YYVHDDLSNVDIKYDEPSVSQ-------DFALPLSKDSFKATLEAYSIV
C.reinhardtiiPDI-C       113 DMERQGAAVEDTAHKVGPKYDAEGHFDEEPDI-------DITVPLSHENFEATLARYPIA
V.carteriPDI-C           113 ELERMGSAVEDSSHKPGPKYDEEGRFDDEPDI-------DITVPLSHVNFEATLARYPIV
C.reinhardtiiERV-A       112 --NANGSPVK-EVEKHNVDATKKKP-----------------------------------
V.carteriERV-A           114 --SANGSPVK-EVEKHNVEA----------------------------------------
C.subellipsoideaERV-A    110 --DSNGVVIP-DSIEKHQVG----------------------------------------
O.sativaERV-A3           110 --DAHGNVIE--ARKEGIGG----------------------------------------
Z.maysERV-A2             110 --DAHGNVIE--ARKVSIGG----------------------------------------
Z.maysERV-A3             110 --NSHGNVIE--ARKEGIGG----------------------------------------
S.moellendorffiiERV-A    109 --DPSGKVVQ-PPVQEDIGG----------------------------------------
P.patensERV-A1           111 --DVNGKVIE-PARQESINQ----------------------------------------
P.patensERV-A4           109 --DVHGKAID-APKPDAINA----------------------------------------
P.patensERV-A2           109 --DVHGKVVN-APKPDAINA----------------------------------------
P.patensERV-A3           109 --DVHGKAVD-APKPDAINA----------------------------------------
O.sativaERV-A2           109 --DNLGNVIE--SRKDGVGA----------------------------------------
P.trichocarpaERV-A3      110 --NAHGDVIE--VRQDGIGA----------------------------------------
P.trichocarpaERV-A4      110 --NAHGDVIE--VRQEGIGA----------------------------------------
A.thalianaERV-A2         110 --DSSGNVIE--AKQDGIGH----------------------------------------
O.sativaERV-A1           110 --DVHGNVIA--TKQDAVGG----------------------------------------
Z.maysERV-A1             110 --DAHGNVIA--TRQDVVGG----------------------------------------
A.thalianaERV-A1         110 --DSNGNTIE--ARQDGIGA----------------------------------------
P.trichocarpaERV-A1      110 --DFHGNVIE--ARQDGIGA----------------------------------------
P.trichocarpaERV-A2      110 --DSHGNVIE--SRQDGIGA----------------------------------------
C.reinhardtiiERV-B       113 --DKAGNQIGKAEYHT--------------------------------------------
V.carteriERV-B           111 --DGAGKPIGKAEYHT--------------------------------------------
C.subellipsoideaERV-B    112 --NEAGEKIGLGEYIP--------------------------------------------
P.patensERV-B            111 --HRDGYVLG-SEFVNDLVE----------------------------------------
S.moellendorffiiERV-B    107 --HKDGHILG-SEYLSDLVE----------------------------------------
O.sativaERV-B            109 --DKYGHIIG-TEYLNDLVEK---------------------------------------
Z.maysERV-B              109 --DKYGHIIG-TEYLSDLVEK---------------------------------------
A.thalianaERV-B          109 --NSHGHIIG-TEYISDLVEKGHEHGHS---------------------------------
P.trichocarpaERV-B1      109 --NSYGHITG-TEYLSDLVE----------------------------------------
P.trichocarpaERV-B2      109 --NSHGHITG-TEYLSDLVE----------------------------------------
```

```
O.sativaPDI-C           162 VVNFYAPWCYWSNRLKPSWEKTAKIMRERYDPEMDGRIILAKVDCTEEIDLCRRHHIQGY
Z.maysPDI-C             162 VVNFYAPWCYWSNRLKPSWEKTAKIMRERYDPEMDGRILLGKVDCTEEVELCRRNHIQGY
A.thalianaPDI13         163 VVNFYAPWCYWSNRLKPSWVKASQITRERYNPGTDDRVLLGSVDCTEEPTLCKSNHIQGY
A.thalianaPDI12         162 VVNFNAPWCYWSNRLKPSWEKAANIIKQRYDPEADGRVLLGNVDCTEEPALCKRNHIQGY
A.thalianaPDI7          162 VVNFYAPWCYWCNLLKPSWEKAAKQIKERYDPEMDGRVILAKVDCTQEGDLCRRNHIQGY
P.trichocarpaPDI-C1     162 VVNFFAPWCYWSNRLKPSWEKAAKIIRERYDPEMDGRILLAKVDCTEEGDLCRRNHIQGY
P.trichocarpaPDI-C2     162 VVNFYAPWCYWSNRLKPSWEKAAKIIRERYDPEIDGRILLAKVDCTEEGDLCRRNHIQGY
P.patensPDI-C           167 VVNFYAPWCPWSNKLKASWEKAAKIIADKYNPEMDGRILLAKVDCTVNVELCRSHHIQGY
S.moellendorffiiPDI-C   160 VVNFYAPWCIWSARLKPSWDKAAGIIAEKYHPDT-GRILLGKVDCTDNNDLCRKHHIQGF
C.subellipsoideaPDI-C   161 VVNFYAPWCPWCQRLEPTWEAVTQEVHTKY-PDADGRIRFAKVDCTTEVDLCREHQITGF
C.reinhardtiiPDI-C      166 VINFYAPWCHWCQRLEPTWEAATKEVHDKY-PEWDGRVRFAKVDCTAEVDLCRQHFIQGF
V.carteriPDI-C          166 VVNFFAPWCHWCQRLEPTWEAATKEVHDKY-PEWDGRIRFAKVDCTQEMELCRTHFIQGF
C.reinhardtiiERV-A      134 -------------------------------PSAVNATASAAAGGAPAGGAAGAAGAEGG
V.carteriERV-A          131 -------------------------------TKKVVP-----------------------
C.subellipsoideaERV-A   127 -------------------------------PELDDTLL---------------------
O.sativaERV-A3          126 -------------------------------AKIESPLQ---------------------
Z.maysERV-A2            126 -------------------------------AKIERPLQ---------------------
Z.maysERV-A3            126 -------------------------------AKVERPLQ---------------------
S.moellendorffiiERV-A   126 -------------------------------PKIDKPLQ---------------------
P.patensERV-A1          128 -------------------------------PKLDKPLQ---------------------
P.patensERV-A4          126 -------------------------------PKVQRPLQ---------------------
P.patensERV-A2          126 -------------------------------PKVQKPLQ---------------------
P.patensERV-A3          126 -------------------------------PKVQKPLQ---------------------
O.sativaERV-A2          125 -------------------------------PKIERPLQ---------------------
P.trichocarpaERV-A3     126 -------------------------------PKIDKPLQ---------------------
P.trichocarpaERV-A4     126 -------------------------------PKIDRPLQ---------------------
A.thalianaERV-A2        126 -------------------------------TKIEKPLQ---------------------
O.sativaERV-A1          126 -------------------------------MKVEQPLQ---------------------
Z.maysERV-A1            126 -------------------------------MKMEAPLQ---------------------
A.thalianaERV-A1        126 -------------------------------TKIENPLQ---------------------
P.trichocarpaERV-A1     126 -------------------------------PKIEKPLQ---------------------
P.trichocarpaERV-A2     126 -------------------------------PKIEKPLQ---------------------
C.reinhardtiiERV-B      127 -------------------------------PQSQQIMD---------------------
V.carteriERV-B          125 -------------------------------PQSQQIMD---------------------
C.subellipsoideaERV-B   126 -------------------------------PR---------------------------
P.patensERV-B           128 ------------------------------------------------------------
S.moellendorffiiERV-B   124 ------------------------------------------------------------
O.sativaERV-B           127 ------------------------------------------------------------
Z.maysERV-B             127 ------------------------------------------------------------
A.thalianaERV-B         134 -------------------------------PH---------------------------
P.trichocarpaERV-B1     126 -------------------------------KEHE-------------------------
P.trichocarpaERV-B2     126 -------------------------------KEHE-------------------------
```

```
O.sativaPDI-C            222 PSIRIFRKG--SDLKENQGHHDHESYYGDRDTESLVAAMETYVANIPKDAHVLALEDKSN
Z.maysPDI-C              222 PSIRVFRKG--SDIKENQGHHDHESYYGERDTESLVAAMETYVANIPKEAH--ALEDKSN
A.thalianaPDI13          223 PSIRIFRRG--SGLREDHGNHEHESYYGDRDTDSLVKMVEELLKPIKKEDHKLALDGKSD
A.thalianaPDI12          222 PSIRIFRKG--SDLREDHGHHEHESYYGDRDTDSIVKMVEGLVAPIHPETHKVALDGKS-
A.thalianaPDI7           222 PSIRIFRKG--SDLKDDNAHHDHESYYGDRDTESLVKMVVSLVEPIHLEPHNLALEDKSD
P.trichocarpaPDI-C1      222 PSIRIFRKG--SNLREDHGRHDHESYYGDRDTESLVKTMEALVAPIAMESQRQALEHKPE
P.trichocarpaPDI-C2      222 PSIRIFRKG--SDLRDDHGHHDHESYYGDRDTDSLVKTMEGLVAPIAMESQRHALEHKPE
P.patensPDI-C            227 PSIRIFRKG--HDVRDEHGRHDHESYYGERDTESLVAFMVELVPPATVDG-KFQLEDKSS
S.moellendorffiiPDI-C    219 PSIRIFHKG--HDLKDEHGHHEHDSYYGERDTDSLVKAMEALVPKETT----LALEDKT-
C.subellipsoideaPDI-C    220 PSIRVFRSG--HDEVNVHGVKEHESYRGDRTQASLLAFADNLAPSAGQPHHYIRGVTRM-
C.reinhardtiiPDI-C       225 PSIRVFRKG--HDDIYIGGMHEHEAYMGDRTKDALVAFADSLVPSAGQPHRKLAGLSAA-
V.carteriPDI-C           225 PSIRVFRKG--HDDIVIGGMHEHESYMGDRTKEALVAFADSLVPSAGQPHRKHAALSAA-
C.reinhardtiiERV-A       163 AAGG--AGG--GENATALANGCGSCYGAEDKQGDCCNTCDEVRAAYRRKGWALSNVDHIE
V.carteriERV-A           137 ------VNG--TENSTATPV-CGSCYGAEDRQGDCCNTCDEVRAAYRRKGWALANVDHIE
C.subellipsoideaERV-A    135 ------HKA-------NETE-CGSCYGA-APDEECCNNCEEVRAAYRRKGWGFTDPQQIS
O.sativaERV-A3           134 ------KHG--GRLSKGEEY-CGTCYGAEESDEQCCNSCEEVREAYKKKGWALTNPDLID
Z.maysERV-A2             134 ------KHG--GRLDKGEQY-CGTCYGAEESDEQCCNSCEEVREAYKKKGWALTNPDLID
Z.maysERV-A3             134 ------KHG--GRLDKGEQY-CGTCYGAEESDEQCCNSCEEVREAYKKKGWALTNPDLID
S.moellendorffiiERV-A    134 ------KHG--GRLEHNETY-CGSCFGAEQSDDECCNSCEEVREAYRKRGWAIHNADLID
P.patensERV-A1           136 ------KHG--GRLEHNETY-CGSCFGAETEEDHCCNNCEEVREAYRKKGWALNNPDLID
P.patensERV-A4           134 ------KHG--GRLEHNETY-CGSCFGAASSDDECCNSCEEVREAYRKKGWALINIDIID
P.patensERV-A2           134 ------KHG--GRLEHNETY-CGSCFGAESSDDECCNNCEEVREAYRKKGWALTNADLID
P.patensERV-A3           134 ------KHG--GRLEDHETY-CGSCFGAESSDDQCCNSCEEVREAYRKKGWALTNTDLID
O.sativaERV-A2           133 ------KHG--GRLDHNEVY-CGSCYGSEESDDQCCNSCEDVRDAYRKKGWALTNIEEID
P.trichocarpaERV-A3      134 ------KHG--GRLEHNEEY-CGSCFGAEMSDDHCCNSCDEVREAYRKKGWALTNMDLID
P.trichocarpaERV-A4      134 ------SHG--GRLGHNEEY-CGSCFGGEMSHDDCCNTCEEVREAYRRKGWAMTNMDLID
A.thalianaERV-A2         134 ------KHG--GRLEHNETY-CGSCFGAEASDDACCNSCEEVREAYRKKGWALSDPESID
O.sativaERV-A1           134 ------RHG--GRLEHNETY-CGSCYGAEESDEQCCNSCEDVREAYRKKGWGVSNPDLID
Z.maysERV-A1             134 ------HHG--GRLEHNETY-CGSCYGAQESDDQCCNTCEDVREAYRKKGWGVSNPDLLD
A.thalianaERV-A1         134 ------KHG--GRLGHNETY-CGSCYGAEAEEHDCCNSCEDVREAYRKKGWGVTNPDLID
P.trichocarpaERV-A1      134 ------RHG--GRLEHNETY-CGSCYGAEASDEDCCNSCEDVREAYRKKGWAVTNPDLMD
P.trichocarpaERV-A2      134 ------RHG--GRLEHNETY-C---------DEDCCNSCEEVREAYQKKGWAVTNPDLMD
C.reinhardtiiERV-B       135 ---------------------TGGEQLVSVNIQEAMQHLVDMEDEA--------------
V.carteriERV-B           133 ---------------------TGAEQLVSVNIQEAMQHLVDMEEEA--------------
C.subellipsoideaERV-B    128 ------RWG--FMMGKPRQE------------------VMEVNQAM--------------
P.patensERV-B            128 -------GE--HRKEEPKADKKDEHKDGDHRKKDPQKVINEVKKAI--------------
S.moellendorffiiERV-B    124 -------------KEHAHDN-LTGIFHSHEELRSAVKVVNEINKAL--------------
O.sativaERV-B            127 ------EHG-THNHDHDHEHEDEQKKQEHTFNEDAEKMVKSVKQAM--------------
Z.maysERV-B              127 ------GHGAHHDHDHDHDHHDEQKKHEQTFNEEAEKMIKSVKQAL--------------
A.thalianaERV-B          136 ------KHD--GKEEHKNET-ETEALNILGFDQAAETMIKKVKQAL--------------
P.trichocarpaERV-B1      130 ------AHN--HDHNKDHHEDSHAKQHTHGFDDAAETMVKKVKQAL--------------
P.trichocarpaERV-B2      130 ------AHN--HDHDKDHHKDSHEEQHTHGFDDAAETMIKKVKQAL--------------
```

```
                                     BLOCK#3------->
O.sativaPDI-C          280 KT-VDPAKRPA--PLTSGCRIEGFVRVKKVPGSVVISARSGSH----------SFDPSQI
Z.maysPDI-C            278 KT-VDPAKRPA--PMASGCRIEGFVRVKRVPGSVVISARSGSH----------SFDPSQI
A.thalianaPDI13        281 NA-ASTFKKA---PVSGGCRIEGYVRAKKVPGELVISAHSGAH----------SFDASQM
A.thalianaPDI12        279 ---NDTVKHLKKGPVTGGCRVEGYVRVKKVPGNLVISAHSGAH----------SFDSSQM
A.thalianaPDI7         280 NSSR----TLKKAPSTGGCRVEGYMRVKKVPGNLMVSARSGSH----------SFDSSQM
P.trichocarpaPDI-C1    280 NA-TQHVKRPA--PSAGGCRIEGYVRVKKVPGNLMISALSGAH----------SFDSKQM
P.trichocarpaPDI-C2    280 NA-TEHVKRPA--PSAGGCRIEGYVRVKKVPGNLVISARSGAH----------SFDSAQM
P.patensPDI-C          284 ITVNATIKRPA--PKAGGCRVEGFVRVKKVPGELMISAHSGSH----------SFDATSM
S.moellendorffiiPDI-C  272 ---NGTVKRPA--PRAGGCRIEGFIRAKKVPGNIIISAHSGSH----------SFDASAM
C.subellipsoideaPDI-C  277 -------------AKTSGCALSGFVLVKKVPGALHFLAKSPGH----------SFDYQAM
C.reinhardtiiPDI-C     282 -------------PKTPGCNLAGFVMVKKVPGTVHFVARSEGH----------SFDHTWM
V.carteriPDI-C         282 -------------PKTPGCNLAGFVMVKKVPGTLTVVARSEGH----------SFDHTWM
C.reinhardtiiERV-A     219 QCAHDLYTEAIKEQAGEGCHMWGMLEVNKVAGNFHFAPGRSYQQGSMHVHDIAPFGDAVI
V.carteriERV-A         188 QCAHDLYTESIKEQTGEGCHMWGMLEVNKVAGNFHFAPGRSYQQGSMHVHDIAPFGDAVI
C.subellipsoideaERV-A  180 QCAKEGFVEKLRAQEGEGCHMWGSLAVNKVAGNFHFAPGKSFQQGPMHVHDLVPFQGVTF
O.sativaERV-A3         185 QCTREDFVERVKTQQGEGCNVHGFLDVSKVAGNLHFAPGKGFYESNINVPEL-SALEHGF
Z.maysERV-A2           185 QCAREDFVERVKTQQDEGCNVHGFLDVSKVAGNFHFAPGKGFYESNIDVPEL-SLLEGGF
Z.maysERV-A3           185 QCAREDFIDRVKTQQDEGCNVLGFLDVSKVAGNFHFAPGKGFYESNIDVPEL-SLLEGGF
S.moellendorffiiERV-A  185 QCKREGWLTKIKEEEGEGCNIYGSLEVNKVAGNFHFAPGKSFSQQHVHVHDVQSLHKEKF
P.patensERV-A1         187 QCKREGFLQKIKDEDGEGCNVYGTLEANKVAGNFHFAPGKSFQQANMHVHDLMAFGKDSF
P.patensERV-A4         185 QCHREGFIERVKEEAGEGCNIYGKLEVNKVAGNFHIAPGKLFQQSAMHLLDLLGIRSDSF
P.patensERV-A2         185 QCHREGFIERVKEEAGEGCNIYGKLEVNKVAGNFHFAPGKSFQQSAMHLLDLMGFITDSF
P.patensERV-A3         185 QCHREGFIERIKEEAGEGCNIYGKLEVNKVAGNFQIAPGKSFQQSAMHLLDLMGFVTDSF
O.sativaERV-A2         184 QCKREGFVQRLKDEQGEGCSIHGFVNVNKVAGNFHFAPGKSLDQSFNFLQDLLNFQQENY
P.trichocarpaERV-A3    185 QCIREGFVQMIKDEEGEGCNINGSLEVNRVAGNFHFVPGKSFHQSNFQLLDLLDMQKESY
P.trichocarpaERV-A4    185 QCKREGFIQMIKDEEGEGCNINGSLEVNRVAGSFHFAPWKSFHLSNFLIQDLLDLQKDSY
A.thalianaERV-A2       185 QCKREGFVQKVKDEEGEGCNVHGFLEVNKVAGNFHFIPGQSFHQSGFQFHDMLLFQQGNY
O.sativaERV-A1         185 QCKREGFLQSIKDEEGEGCNIYGFLEVNKVAGNFHFAPGKSFQKANVHVHDLLPFQKDSF
Z.maysERV-A1           185 QCKREGFLQSIKDEEGEGCNIYGFIEVNKVAGNFHFAPGKSFQQSNVHVHDLLPFQKDSF
A.thalianaERV-A1       185 QCKREGFLQRVKDEEGEGCNIYGFLEVNKVAGNFHFAPGKSFHQSGVHVHDLLAFQKDSF
P.trichocarpaERV-A1    185 QCKREGFLQKIKDEEGEGCNIYGFLEVNKVAGNFHFAPGKSFQQSGVHVHDLLAFQKDSF
P.trichocarpaERV-A2    176 QCKREGFLQRIKDEEGEGCNIYGFLEVNKVAGNFHFAPGKSFQQSGVHVHDLLAFQKDSF
C.reinhardtiiERV-B     160 -------------DHHEGCHVYGTMEVKRVAGRLHLSVHQNMVFQMLPQLLGTHHIPKIL
V.carteriERV-B         158 -------------EHHEGCHVYGTMDVKRVAGRLHFSVHQNMVFQMLPQLLGAHRIPKVA
C.subellipsoideaERV-B  148 -------------DAHEGCNIFGWLDLQRVAGNFRVS----VHVEDFFALTRLQADTTGI
P.patensERV-B          165 -------------DDGEGCQIFGVLDVERVAGNFHIS----MHGLSLYVASKIFEAGYEV
S.moellendorffiiERV-B  156 -------------QDGEGCRVFGVLDVERVAGNFHIS----MHGMSLQVARQIFHSVKEV
O.sativaERV-B          166 -------------ENGEGCRVYGVLDVQRVAGNFHIS----VHGLNIFVAEKIFDGSSHV
Z.maysERV-B            167 -------------GNGEGCRVYGMLDVQRVAGNFHIS----VHGLNIFVAEKIFEGSNHV
A.thalianaERV-B        173 -------------ADGEGCRVYGVLDVQRVAGNFHIS----VHGLNIYVAQMIFGGSKNV
P.trichocarpaERV-B1    168 -------------ANGEGCRVYGVLDVQRVAGNFHIS----VHGLNIFVAQMIFDGAKHV
P.trichocarpaERV-B2    168 -------------ANGEGCRVYGVLDVQRVAGNFHIS----VHGLNIFVAQMIFDGAKHV
                                     COPII-coated Erv (consensus)---------------
```

```
                                BLOCK#4---->
O.sativaPDI-C            327 NVSHYVTQFSFGKRLSAKMFNELKRLTPYVGGHHD----RLAGQ---SYIVKHGDVNANV
Z.maysPDI-C              325 NVSHYVTQFSFGKRLSPRMLHEFIRLTPYLRGYHD----RLAGQ---SYTVKHGEVNANV
A.thalianaPDI13          327 NMSHIVTHLTFGTMVSERLWTDMKRLLPYLGQSYD----RLNGK---SF-INERQLDANV
A.thalianaPDI12          326 NMSHVVSHFSFGRMISPRLLTDMKRLLPYLGLSHD----RLDGK---AF-INQHEFGANV
A.thalianaPDI7           326 NMSHVVNHLSFGRRIMPQKFSEFKRLSPYLGLSHD----RLDGR---SF-INQRDLGPNV
P.trichocarpaPDI-C1      327 NLSHVISHFSFGMKVLPRVMSDVKRLLPYIGRSHD----KLNGR---SF-INHRDVGANV
P.trichocarpaPDI-C2      327 NLSHVISHFSFGMKVLPRVMSDVKRLIPHIGRSHD----KLNGR---SF-INHRDVGANV
P.patensPDI-C            332 NMTHYVGFFSFGRKTSWRSVHWVNEMLPALDSNID----RLTGQ---VF-PSEYEN---I
S.moellendorffiiPDI-C    317 NMTHYVSQFTFGRELNFWMRRELYRIYPHLASVYDTVEANLTGR---IY-VSQHEN---I
C.subellipsoideaPDI-C    314 NMSHVVNYLYFGNKPSPRRHQSLAKLHP--AGLSDDWADKLAGQ---DF-FSRAAK---A
C.reinhardtiiPDI-C       319 NMTHMIHSFHVGTRPSPRKYQQLKRLHP--AGLTADWADKLHDQ---LF-VSEHTQ---S
V.carteriPDI-C           319 NMTHLVHTFHVGTRPSPRKYQQLKRLHP--AGLTHDWADQLRDQ---FF-LSEHPQ---S
C.reinhardtiiERV-A       279 DFRHVIHKLSFG---------------EPYPGMKN----PLDGAKAGQA-AAAAAA-ATG
V.carteriERV-A           248 DFRHTVNKLSFG---------------APYPGMKN----PLDNA---K--AGYKSAAATG
C.subellipsoideaERV-A    240 DLSHRIDKLSFG---------------HEYPGMTN----PLDRVNLPKF-NTRNPQGLPG
O.sativaERV-A3           244 NITHKINKLSFG---------------TEFPGVVN----PLDGA---QW-TQPASD---G
Z.maysERV-A2             244 NITHKINKLSFG---------------TEFPGVVN----PLDGA---QW-TQPASD---G
Z.maysERV-A3             244 NISHKINKLSFG---------------TEFPGVVN----PLDGA---QW-TQPASD---G
S.moellendorffiiERV-A    245 NVSHYINELSFG---------------ARFPGVVN----PLDKE---KR-IQKFPS---A
P.patensERV-A1           247 NVSHKINEISFG---------------VRYPGAVN----PLDKL---ER-IQTTTH---G
P.patensERV-A4           245 NVSHIVNELSFG---------------AHFPGRVN----PLDKI---TS-IQKDQN---G
P.patensERV-A2           245 NVSHTINELSFG---------------AHFPGAVN----PLDKV---TN-IQKDLN---G
P.patensERV-A3           245 NVSHTINELSFG---------------AYFPGAVN----PLDKV---TS-IQKDQN---G
O.sativaERV-A2           244 NISHKINKLSFG---------------VEFPGVVN----PLDGV---EW-IQEHTNGLTG
P.trichocarpaERV-A3      245 NISHRINRLAFG---------------DYFPGVVN----PLDGI---QL-MHETQN---G
P.trichocarpaERV-A4      245 NISHRINRLAFG---------------DYFPGVVN----PLAGI---QL-MHDTPN---G
A.thalianaERV-A2         245 NISHKVNRLAFG---------------DFFPGVVN----PLDGV---QW-NQGKQS---G
O.sativaERV-A1           245 NVSHKINKLSFG---------------QRFPGVVN----PLDGA---QW-MQHSSY---G
Z.maysERV-A1             245 NVSHKINRLSFG---------------EYFPGVVN----PLDGA---NW-VQHSSY---G
A.thalianaERV-A1         245 NISHKINRLTYG---------------DYFPGVVN----PLDKV---EW-SQDTPN---A
P.trichocarpaERV-A1      245 NITHKINRLTFG---------------EYFPGVVN----PLDGV---QW-TQETPS---G
P.trichocarpaERV-A2      236 NTSHKINRLAFG---------------EYFPGVVN----PLDGV---QW-TQETPS---G
C.reinhardtiiERV-B       207 NMSHVIKHLGFG---------------PHYPGQLN----PLDGY---VR-MVGREP---F
V.carteriERV-B           205 NISHTIKHLGFG---------------PHYPGQLN----PLDGY---VR-MVKGPP---Q
C.subellipsoideaERV-B    191 NSSHIIHRVSFG---------------PTFPGQVN----PLDGA---ER-ILDKES---G
P.patensERV-B            208 NVSHVIHDLSFG---------------PTYPGHHN----PLDGS---ER-ILHDTS---G
S.moellendorffiiERV-B    199 NVSHIINDLSFG---------------PKYPGIHN----PLDRT---VR-ILRDTA---G
O.sativaERV-B            209 NVSHIIHDLSFG---------------PKYPGIHN----PLDET---TR-ILHDTS---G
Z.maysERV-B              210 NVSHVIHELSFG---------------PKYPGIHN----PLDET---SR-ILHDTS---G
A.thalianaERV-B          216 NVSHMIHDLSFG---------------PKYPGIHN----PLDDT---NR-ILHDTS---G
P.trichocarpaERV-B1      211 NVSHIIHDLSFG---------------PKYPGIHN----PLDGT---TR-ILHETS---G
P.trichocarpaERV-B2      211 NVSHIIHDLSFG---------------PKYPGIHN----PLDGT---AR-ILRETS---G
```

```
                                                                                   BLOCK#5-------
O.sativaPDI-C                380 TIEHYLQIVKTELVTLRSSKELKLVEEYEYTAH----SSLVHSFYVPVVKFHFEPSPMQV
Z.maysPDI-C                  378 TIEHYLQVVKTELVTQRSSKELKVLEEYEYTAH----SSLVHSFYVPVVKFHFEPSPMQV
A.thalianaPDI13              379 TIEHYLQIIKTEVISRRSGQEHSLIEEYEYTAH----SSVARSYHYPEAKFHFELSPMQV
A.thalianaPDI12              378 TIEHYLQTVKTEVITRRSGQEHSLIEEYEYTAH----SSVAQTYYLPVAKFHFELSPMQI
A.thalianaPDI7               378 TIEHYLQIVKTEVVKSNGQALV---EAYEYTAH----SSVAHSYYLPVAKFHFELSPMQV
P.trichocarpaPDI-C1          379 TIEHYLQVVKTEVVTRRSSSERKLIEEYEYTAH----SSLSQTVYMPTAKFHFELSPMQV
P.trichocarpaPDI-C2          379 TIEHYLQVVKTEVVTRRSSAEHKLIEEYEYTAH----SSLAQTVYMPTAKFHFELSPMQV
P.patensPDI-C                381 THDHYLQVVKTEVITLHRKQDLRVLEQYDYTAH----SNMIQSTKVPVVKFHYELSPMQV
S.moellendorffiiPDI-C        370 THDHYLQVVKTEVVSLRKRKEFSLLEQYDYTSH----SNTIQNTNVPVAKFHYELSPMQV
C.subellipsoideaPDI-C        365 TFEHYMQVVLTTIEPSKHRPELSY-DAYEYTVH----SHTYDTADIPAAKFTYDLSPIQI
C.reinhardtiiPDI-C           370 THEHYLQVVLTTIEPRHSRHTGNY-DAYEYTAH----SHSYQSDSIPSARFTYDLSPIQI
V.carteriPDI-C               370 THEHYLQIVLTSIEPRRSRHSGNY-DAYEYTAH----SHTYQSDAIPSARFTYDLSPIQI
C.reinhardtiiERV-A           318 MFQYFLKVVPTSYTDLSNKTLST--NQFSVTENFRE-AQGGAGRTLPGVFFFYDLSPIKV
V.carteriERV-A               284 MYQYFLKVVPTSYTGIDNKTLAT--NQFSVTENFRESSQGGAGKTLPGVFFFYDLSPIKV
C.subellipsoideaERV-A        280 AYQYFLKVVPTIYVNSHNHTINS--NQYSVTEHFK--GSQDFQAQLPGVFFYYDLSPIKV
O.sativaERV-A3               278 TYQYFIKVVPTIYTDLRGRKIHS--NQFSVTEHFR--DGNIRPKPQPGVFFFYDFSPIKV
Z.maysERV-A2                 278 TYQYFIKVVPTIYTDIRGHNIHS--NQFSVTEHFR--DGNVRPKPQPGVFFFYDFSPIKV
Z.maysERV-A3                 278 TYQYFIKVVPTIYTDIRGRGIHS--NQFSVTEHFR--DGNVRPKSQPGVFFFYDFSPIKV
S.moellendorffiiERV-A        279 MYQYFIKVVPTAYTDMTGHKIVT--NQFSVTDHFKA-VEGLNGRSLPGVFFFYELSPIKV
P.patensERV-A1               281 MYQYFIKVVPTVYTDTRGRKIST--NQFAVTDHFKG-VGPGEDHALPGVFFFYDLSPIKV
P.patensERV-A4               279 MYQYFIKVVPTVYTDIRGSEIAT--NQFSVTEHYT--AGDHGPRVVPGVFFFYDLSPIKV
P.patensERV-A2               279 MYQYFIKVVPTVYTDIKGRKIST--NQFSVTEHYT--AGDHGPRFVPGVFFFYDLSPIKV
P.patensERV-A3               279 MFQYFIKVVPTVYTDIKGRKIST--NQFSVMEHYT--AGDHGPRVIPGVFFFYDLTPIKV
O.sativaERV-A2               281 MYQYFVKVVPTIYTDIRGRKINS--NQFSVTEHFR--EAIGYPRPPPGVYFFYEFSPIKV
P.trichocarpaERV-A3          279 VQQFFIKVVPTIYTDIRGRTVHS--NQYSVTEHFTK-SELMRLDSLPGVYFIYDFSPIKV
P.trichocarpaERV-A4          279 VQQFFIKVVPTIYTDIRGRTVHS--NQYSATEHFKK-SELTPLDSLPGVYFFYDFSPIKV
A.thalianaERV-A2             279 VYQYFIKVVPSIYTDVHQNTIQS--NQFSVTEHFQN-MEAGRMQSPPGVFFYYDLSPIKV
O.sativaERV-A1               279 MYQYFIKVVPTVYTDINEHIILS--NQFSVTEHFRS-SESGRIQAVPGVFFFYDLSPIKV
Z.maysERV-A1                 279 MYQYFIKVVPTVYTDINEHIILS--NQFSVTEHFRS-GESGRMQALPGVFFFYDLSPIKV
A.thalianaERV-A1             279 MYQYFIKVVPTVYTDIRGHTIQS--NQFSVTEHVKS-SEAGQLQSLPGVFFFYDLSPIKV
P.trichocarpaERV-A1          279 MYQYFIKVVPTVYTDVSGHTIQS--NQFSVTEHFRG-TDIGRLQSLPGVFFFYDLSPIKV
P.trichocarpaERV-A2          270 MYQYFIKVVPTVYTDVSGHTIQS--NQFSVTEHFRG-ADIGRLQSLPGVFFFYDLSPIKV
C.reinhardtiiERV-B           241 SYKYFLKVVPTEYYNRLGRATET--HQYSVTEY----AQPLQRGYAPAVDVHYDLSPIVM
V.carteriERV-B               239 SFKYFLKVVPTEYYNRLGRVTET--HQYSVTEY----TQPLEPGYVPTLDVHYDLSPIVM
C.subellipsoideaERV-B        225 TFKYFLKVVPTEYVKLDGTRTTT--NQYSVTEYDT--VVHKGEMQMPSVWFSYDISPISV
P.patensERV-B                242 TFKYFLKIVPTEYHYLHGEVMPT--NQFSVTEYYQ--RTKPSDRSYPAVYFVYDLSPIVV
S.moellendorffiiERV-B        233 TFKYFIKIVPTEYRYLNGGKLPT--NQFSVGEYYL--AARDDDISWPAVYFLYDLSPITV
O.sativaERV-B                243 TFKYYIKIVPTEYRYLSKQVLPT--NQFSVTEYFVP-KRATDRSAWPAVYFLYDLSPITV
Z.maysERV-B                  244 TFKYYIKVVPTEYKYLSKKVLPT--NQFSVTEYFL--PIRPTDRAWPAVYFLYDLSPITV
A.thalianaERV-B              250 TFKYYIKIVPTEYRYLSKDVLST--NQYSVTEYFT--PMTEFDRTWPAVYFLYDLSPITV
P.trichocarpaERV-B1          245 TFKYYIKIVPTEYRYISKEVLPT--NQFSVTEYFS--PMTDFDRTWPAVYFLYDLSPITV
P.trichocarpaERV-B2          245 IFKYYIKIVPTEYRYISKDVLPT--NQFSVTEYFS--PITDFDRTWPAVYFLYDLSPITV
                                 ------------------------------------------------------------
```

```
O.sativaPDI-C           436 LVTELPKSFSHFITNVCAIIGGVFTVAGILDSIFHNTLRLVK-KVELGKNI-
Z.maysPDI-C             434 LVTEVPKSFSHFITNVCAIIGGVFTVAGILDSIFHNTLRMVK-KIELGKNI-
A.thalianaPDI13         435 LISENPKSFSHFITNVCAIIGGVFTVAGILDSIFQNTVRMVK-KIELGKNI-
A.thalianaPDI12         434 LITENPKSFSHFITNLCAIIGGVFTVAGILDSIFHNTVRLVK-KVELGKNI-
A.thalianaPDI7          431 LITENSKSFSHFITNVCAIIGGVFTVAGILDSILHHSMTLMK-KIELGKNF-
P.trichocarpaPDI-C1     435 LITENSKSFSHFITNVCAIIGGVFTVAGILDSILHHTVRMMK-KVELGKNF-
P.trichocarpaPDI-C2     435 LITENPKSFSHFITNVCAIIGGVFTVAGILDSILHNTFRMMK-KVELGKNF-
P.patensPDI-C           437 LVKENPKSFSHFLTNLCAIIGGVFTVAGIIDSMLHNAMHIMK-KVELGKQY-
S.moellendorffiiPDI-C   426 LVKENPKSFSHFITNVCAIIGGVFTVAGIVDSMLHGAMRMVK-KIELGKQF-
C.subellipsoideaPDI-C   420 LVSEKRRAWYHFVTTTCAIIGGVFTVAGIVDGLVHTGARFAK-KVELGKHT-
C.reinhardtiiPDI-C      425 LVHETSKPWYQFLTTSCAIIGGVFTVAGILDALLYQSFKVVK-KLNLGKQG-
V.carteriPDI-C          425 LVQETARPWYQFLTTSCAIIGGVFTVAGILDALLYQSFKVVK-KLNLGKQG-
C.reinhardtiiERV-A      375 KIVEHGSSFLSFLTSVCAIVGGVFTVSGIVDAFVYTGTRMIKKKMELGKFS-
V.carteriERV-A          342 RIVEHSSSFLSFLTSVCAIVGGVFTVSGIVDAFIYTSTRLIRKKMELGKFS-
C.subellipsoideaERV-A   336 KYHETRMSFLHFLTSVCAIVGGIFTVAGIVDAFIYHGHQAIKKKVDLGKQI-
O.sativaERV-A3          334 IFTEENSSLLHYLTNLCAIVGGVFTVSGIIDSFIYHGQKALKKKMELGKYR-
Z.maysERV-A2            334 IFTEESRSLLHYLTNLCAIVGGVFTVSGIIDSFIYHGQKALKKKMELGKYR-
Z.maysERV-A3            334 IFTEENRSLLHYLTNLCAIVGGVFTVSGIIDSFIYHGQKALKKKMELGKYR-
S.moellendorffiiERV-A   336 LFTERKTSFLHFLTNVCAIIGGVFTVSGIIDSFIYHGHRAIKKKMEIGKYI-
P.patensERV-A1          338 KFTEKRMSFFHFLTNVCAIVGGVFSVSGIIDAFVYHGQKQIKKR--LGKDT-
P.patensERV-A4          335 KFTEKRPSFLHFLTTVCAIVGGVYTVASIIDSFIYHGHRAVKKKMELGKFS-
P.patensERV-A2          335 KFSEERPSFLHFLTNVCAIVGGVYSIAGIIDSFVYHGHRAIKKKMELGKLS-
P.patensERV-A3          335 KFTEERPSFLHFLTNVCAIIGGIYTIAGIVDSFIYHGHRAIKKKMELGKLS-
O.sativaERV-A2          337 DFTEENTSLLHFLTNICAIVGGIFTVAGIIDSFVYHGHRAIKKKMEIGKLG-
P.trichocarpaERV-A3     336 TFKEEHTSFLHFMTSICAIIGGIFTIAGIVDSFIYHGRRAIKKKMEIGKFS-
P.trichocarpaERV-A4     336 IFKEEHISFLHFMTSICAIIGGIFTIAGIIDSFIYYGQRAITKKVGIGKFG-
A.thalianaERV-A2        336 IFEEQHVEFLHFLTNVCAIVGGIFTVSGIVDSFIYHGQRAIKKKMEIGKFN-
O.sativaERV-A1          336 TFTEQHVSFLHFLTNVCAIVGGVFTVSGIIDSFVYHGQRAIKKKMEIGKFN-
Z.maysERV-A1            336 TFTEQHVSFLHFLTNVCAIVGGVFTVSGIIDSFVYHSQRAIKKKMEIGKFN-
A.thalianaERV-A1        336 TFTEEHISFLHFLTNVCAIVGGVFTVSGIIDAFIYHGQKAIKKKMEIGKFS-
P.trichocarpaERV-A1     336 TFTEEHVSFLHFLTNVCAIVGGVFTVSGILDTFIYHGQKAIKKKMEIGKFS-
P.trichocarpaERV-A2     327 TFTEEHVSFLHFLTNVCAIVGGVFTVSGILDSFIYHGQKAIKKKMEIGKFS-
C.reinhardtiiERV-B      295 TINERPPSLLHFVVRLCAVVGGVFAITRLTDRWVDWLVRLVNKAAARGP---
V.carteriERV-B          293 TINERPPSLLHFVVRLCAVVGGAFAITRMTDRWVDWFVRLVT-KLK------
C.subellipsoideaERV-B   281 TISEIRKSFAHLLVRFCAVVGGVFAVTGMFDRWVHRIVTAIF-----SASS-
P.patensERV-B           298 TIREHRRNFGHFITRLCAVLGGTFAVTGMLDRWMSRIIDFVMSTSKQGFL--
S.moellendorffiiERV-B   289 LIKEERRSFGHLLTRFCAIVGGTFSLTGMLDRWIYRLVESIT--RAKGVLI-
O.sativaERV-B           300 TIKEERRNFLHFLTRLCAVLGGTFAMTGMLDRWMYRLIESVTKSKTRSVLR-
Z.maysERV-B             300 TIKEERRNFLHFVTRLCAVLGGTFAMTGMLDRWMYQLIKTVTNSKTRSVLR-
A.thalianaERV-B         306 TIKEERRSFLHLITRLCAVLGGTFALTGMLDRWMFRFIESFNKKPSTRA---
P.trichocarpaERV-B1     301 TIKEERRSFLHFITRLCAVLGGTFALTGMLDRWMCRLLEALTKPNPRSVLR-
P.trichocarpaERV-B2     301 TIKEERRSFLHFITRLCAILGGTFALTGMLDRWMYRLLEALT-KPNRGSGFL
```

Multiple sequence alignment for NJ and ML trees in Fig. 3

```
                                BLOCK#1-------------------------------------------------------

B.distachyonPDI-C          1  MISS-SKLKSVDFYRKIPRDLTEASLSGAGLSIVAALAMVFLFGMELSSYLAVNTTTSVI
O.sativaPDI-C              1  MISS-SKLKSVDFYRKIPRDLTEASLSGAGLSIVAALAMVFLFGMELSNYLAVNTSTSVI
E.salsugineumPDI-C1        1  MVSS-TKIKSVDFYRKIPRDLTEASLSGAGLSIVAALVMMLLFGMELSSYLEVSTTTAVV
A.thalianaPDI12            1  MVSS-TKLKSVDFYRKIPRDLTEASLSGAGLSIVAALFMMFLFGMELSSYLEVNTTTAVI
C.rubellaPDI-C1            1  MVSS-TKLKSVDFYRKIPRDLTEASLSGAGLSIVAALFMVFLFGMELSSYLEVNTTTSVI
E.salsugineumPDI-C3        1  MVST-TKIKSVDFYRKIPRDLTEASLSGAGLSIVAALAMVFLFGMELSNYLAVSTNTSVI
A.thalianaPDI13            1  MVST-SKIKSVDFYRKIPRDLTEASLSGAGLSIVAALAMLFLFGMELSSYLAINTSTSVI
C.rubellaPDI-C3            1  MVST-TKIKSVDFFRKIPRDLTEASLSGAGLSIVAALAMLFLFGMELSSYLAVNTSTSVI
E.salsugineumPDI-C2        1  MVST-SKIKSVDFYRKIPRDLTEATLSGAGLSIVAALSMLFLFGMELNNYLAVSTTTSII
A.thalianaPDI7             1  MVST-SKIKSVDFYRKIPRDLTEASLSGAGLSIIAALSMIFLFGMELNNYLAVSTSTSVI
C.rubellaPDI-C2            1  MVST-SKIKSVDFYRKIPRDLTEATLSGAGLSIIAALSMLFLFGMELNNYLAVSTSTSVI
S.lycopersicumPDI-C        1  MVST-SKIKSVDFYRKIPRDLTEASLSGAGLSIVAALCMMFLFGMELNNYLTVSTTTSVI
S.tuberosumPDI-C           1  MVST-SKIKSVDFYRKIPRDLTEASLSGAGLSIVAALSMMFLFGMELNNYLTVSTTTSVV
M.truncatulaPDI-C1         1  MTTA-SKIKSVDFYRKIPRDLTEASLSGAGLSIVAALAMMFLFGMELNEYLSVHTSTSVI
P.vulgarisPDI-C1           1  MISS-SKLKSVDFYRKIPRDLTEASLSGAGLSIVAALCMVFLFGMELNSYLSVSTATSVI
G.maxPDI-C1                1  MISS-SKIKSVDFYRKIPRDLTEASLSGAGLSIVAALAMIFLFGMELNSYLSVTTSTQVI
G.maxPDI-C2                1  MISS-SKIKSVDFYRKIPRDLTEASLSGAGLSIVAALAMIFLFGMELNSYLSVSTSTQVI
M.truncatulaPDI-C2         1  MLSA-SKLKSVDFYRKIPRDLTEASLSGAGLSILAALAMMFLFGMELSNYFAVTTSTSVI
P.vulgarisPDI-C2           1  MISA-TKLKSVDFYRKIPRDLTEASLSGAGLSIVAALSMMFLFGMELSNYLTVSTSTSVI
G.maxPDI-C3                1  MISA-TKLKSVDFYRKIPRDLTEASLSGAGLSIVAALVMMFLFGMELSSYMSVSTSTSVI
G.maxPDI-C4                1  MISA-TKLKSVDFYRKIPRDLTEASLSGAGLSIVAALAMMFLFGMELSSYLSVSTSTSVI
V.viniferaPDI-C            1  MVST-SKIKSVDFYRKIPRDLTEASLSGAGLSVIAALSMMFLFGMELSNYLSVSTSTSVI
P.trichocarpaPDI-C1        1  MVST-NKLKSVDFYRKIPRDLTEASLSGAGLSIVAALAMMFLFGMELNNYLTVNTSTTVI
P.trichocarpaPDI-C2        1  MVST-NKLKSVDFYRKIPRDLTEASLSGAGLSIVAALAMVFLFGMELNNYLTVNTSTSVI
P.patensPDI-C              1  MVST-SKLKSIDFYRKIPRDLTEASLSGAGLSIIAALTMVFLFGMELSAYLSTTTSTSVV
S.moellendorffiiPDI-C      1  MTTA-SKIKSIDFYRKIPRDLTEASLSGAGLSLIAAFAMIFLFGMELNNYLTVSSTTNVV
C.subellipsoideaPDI-C      1  MARVLQKLRSVDFYRKIPNDLTEATLAGAGISLVAAFTIVVLLTAELSSFLAIETKEELI
C.reinhardtiiPDI-C         1  MVRLFSRLKAIDFFKKIPSDLTEATLTGAWLSIVAAVLMILLFVAELSAFLSTTTSSQLV
V.carteriPDI-C             1  MARLFSKLKAIDFFKKIPSDLTEATLTGAWISILAAVIMVFLFTAEMMSFLSTTTTTQLI

                              ------------------------------------------------------------

B.distachyonPDI-C         60  VDRSSDGEFLRIDFNMSFPALSCEFASVDVSDVLGTNRLNITKTVRKFSIDRNLVPTGSE
O.sativaPDI-C             60  VDRSSDGEFLRIDFNLSFPALSCEFASVDVSDVLGTNRLNITKTVRKYSIDRNLVPTGSE
E.salsugineumPDI-C1       60  VDNSADGDFLRITFNISFPALSCEFASVDVSDVLGTNRLNITKTVRKFPIDPHLRTTGAE
A.thalianaPDI12           60  VDKSSDGDFLRIDFNISFPALSCEFASVDVSDVLGTNRLNITKTVRKFPIDPHLRSTGAE
C.rubellaPDI-C1           60  VDKSTDGDFLRIDFNISFPALSCEFASVDVSDVLGTNRLNITKTIRKFPIDPHLRSTGAE
E.salsugineumPDI-C3       60  VDKSSDGDFLRIDFNVSFPSLSCEFASVDVSDVLGTSRLNLTKTIRKVPIDAHLRATGAE
A.thalianaPDI13           60  VDKSSDGDFLNIDFNISFPALSCEFASVDVSDVFGTHRLNISKTIRKVPIDPHLRATAEE
C.rubellaPDI-C3           60  VDKSSDGDILNIDFNISFPALSCEFASVDVSDVLGTHRLNLTKTIRKVPIDPHLRATGTE
E.salsugineumPDI-C2       60  VDRSSDGDFLRMDFNISFPSLSCEFASVDVSDVLGTNRLNVTKTIRKFSIDSNLRPTGTE
A.thalianaPDI7            60  VDRSADGDFLRLDFNISFPSLSCEFASVDVSDVLGTNRLNVTKTIRKFSIDSNMRPTGSE
C.rubellaPDI-C2           60  VDRSSDGDFLRLDFNISFPSLSCEFASLDVSDVLGTNRLNVTKTIRKFSIDSNMRPTGAE
S.lycopersicumPDI-C       60  VDKSSDAEFLRIDFNMSFPALSCEFASVDVSDVLGTNRLNITKTVRKHSIDKNLRPTGSE
S.tuberosumPDI-C          60  VDKSSDAEFLRIDFNMSFPALSCEFASVDVSDVLGTNRLNITKTVRKHSIDKNLRPTGSE
M.truncatulaPDI-C1        60  IDKSSDGEFLRIDFNLSFHALSCEFASVDVSDVLGTNRMNLTKTVRKFSIDSNLRPTGSE
P.vulgarisPDI-C1          60  VDKSSDGDYLRIDFNMSFPALSCEFAAVDVSDVLGTNRLNLTKTIRKFSIDSNLRSTGNE
G.maxPDI-C1               60  VDKSSDGDYLRIDFNISFPALSCEFAAVDVSDVLGTNRLNLTKTVRKFSIDSNLRPTGAE
G.maxPDI-C2               60  VDKSSDGDYLRIDFNISFPALSCEFAAVDVSDVLGTNRLNLTKTVRKFSIDSNLRPTGAE
M.truncatulaPDI-C2        60  VDKSSDGDFLRIDFNFSFPALSCEFASVDVSDVLGTNRLNITKTVRKFSIDSKLRPTGSE
P.vulgarisPDI-C2          60  VDKSSDHDYLRIDFNISFPALSCEFASVDVSDVLGTNRLNITKTVRKFSIDSHLRPTGAE
G.maxPDI-C3               60  VDKSSDGDYLRIDFNISFPALSCEFASVDVSDVLGTNRLNITKTVRKFSIDSNLRPTGAE
G.maxPDI-C4               60  VDKSSDGDYLRIDFNISFPALSCEFASVDVSDVLGTNRLNITKTVRKFSIDSNLRPTGAE
V.viniferaPDI-C           60  VDQSSDGDFLRIEFNISFPALSCEFASVDVSDVLGTNRLNITKTIRKYSIDPDLRPTGAE
P.trichocarpaPDI-C1       60  VDNSSDGEFLRIDFNISFPSLSCEFASVDVSDVLGTNRLNITKTIRKFSIDHDLKPTGSE
P.trichocarpaPDI-C2       60  VDNSSDGEFLRIDFNLSFPSLSCEFASVDVSDVLGTNRLNITKTIRKFSIDHDLKPTGSE
P.patensPDI-C             60  VDRSRDGEYLRIDFNLSFPALSCEFASVDVSDVLGTHRFNLTKTVRKYPIDPLLQRIGQE
S.moellendorffiiPDI-C     60  VDRSKDGEYLRIQFNMSFPALSCEFASVDVSDALGTNRYNLTKTVRKYPIDPNLKIVGPE
C.subellipsoideaPDI-C     61  VDRSAHGDLLRINFNISFPSLSCEFATLDVSDALGTKRMNLTKTIRKLPIDEDGQRAGYY
C.reinhardtiiPDI-C        61  VDRSPQNELLKLNFNISFPALSCEFATVDVSDSLGTKRMNLTKTVRKVPITLDMERQGAA
V.carteriPDI-C            61  VDRSPQNELLKLNFNISFPALSCEFATVDVSDTLGTKRMNLTKTVRKMPITTELERMGSA
```

```
                                 --------------->               BLOCK#2---------------------
B.distachyonPDI-C        120 FHSGPIPTVNKHGDDV--EE--YH-ADGSVALSSRNFDSYSHQYPILVVNFYAPWCYWSN
O.sativaPDI-C            120 FHPGPIPTVSKHGDDV--EE--NH-DDGSVPLSSRNFDSYSHQYPVLVVNFYAPWCYWSN
E.salsugineumPDI-C1      120 FHSGLALHDINHGEES--KE--EF-PDGAIPLTSGSFESYSHHFPILIVNFYAPWCYWSN
A.thalianaPDI12          120 FHSGLALHNINHGEET--KE--EF-PDGAIPLTSASFEALSHHFPILVVNFNAPWCYWSN
C.rubellaPDI-C1          120 FHSGHTLHHINHGEET--KE--EF-PDGAIPLTSGSFESLSHHFPILVVNFNAPWCYWSN
E.salsugineumPDI-C3      120 FHSTTGLDLINHGDED--HH--ENNSYAAIPLTGATFDKFSHHFPILVVNFYAPWCYWSN
A.thalianaPDI13          120 FHSTSDLHLINHGDED--HG--DNSTYADIPLTGAAFEKFTHHFQILVVNFYAPWCYWSN
C.rubellaPDI-C3          120 FHSTSGLHLINHGDED--HE--EN-TYIDIPLTGATFEKYKHHFQILVVNFYAPWCYWSN
E.salsugineumPDI-C2      120 FHSGEVLSLINHGDEA--GE--EL-VEDSVSLTGRNFDTLLHQFPISVVNFYAPWCYWCN
A.thalianaPDI7           120 FHAGEVLSLINHGDET--GE--EI-VEDSVPLTGRNFDTFTHQFPILVVNFYAPWCYWCN
C.rubellaPDI-C2          120 FHAGEVLSLINHGDET--GE--EV-AEDSVSLTGRNFETFTHQFPILVVNFYAPWCYWCN
S.lycopersicumPDI-C      120 FHSGSTATELKHDAED--DE--EY-GEGSVSLNGHSFDRVTHHFPILVVNFFAPWCYWSN
S.tuberosumPDI-C         120 FHSGSTATELKHDAED--DE--EY-GEGSVSLNGHSFDRVTHHYPILVVNFFAPWCYWST
M.truncatulaPDI-C1       120 FYLRPSTNVIKHDDKV--DD--ES-LEGAFVFTSNNFDKYSHQFPITAVNFYAPWCYWSQ
P.vulgarisPDI-C1         120 FHSEPTTTNIKHDNEQ--HE--ES-IGGALELTTDNFDKYAHQFPITVINFYAPWCYWSQ
G.maxPDI-C1              120 FHSEPAANSIKHDNEV--NE--ES-VEGSVVLKTQNFDKYAHQFPITVVNFYAPWCYWSQ
G.maxPDI-C2              120 FHSEPAANSIKHDNEV--NE--ES-VEGSVVLTTQNFDKYAHQFLITVVNFYAPWCYWSQ
M.truncatulaPDI-C2       120 FHSGTIANAVKHDDEV--DT--DF-VEGSLPLTSQHFDKYVQLFPITVVNFYAPWCSWCQ
P.vulgarisPDI-C2         120 FHSGAVSNAVKHDDEV--DG--ES-VEGSFSFTTHNFDKYAHQFPITVVNFYAPWCSWCQ
G.maxPDI-C3              120 FHSGTVANAVKHDDEV--DE--ES-VEGSFSLTTHNFDKYVHQFPVTIVNFYAPWCSWCQ
G.maxPDI-C4              120 FHSGTVANAVKHDDEV--DE--ES-VEGSFSLTTHNFDKYVHQFPITAVNFYAPWCSWCQ
V.viniferaPDI-C          120 FHSGPVGKVIKHGDET--DE--EY-SEGSASLTAQNFYKYSHQHAILVVNFFAPWCYWST
P.trichocarpaPDI-C1      120 FHSGPVLHQIKHGDEV--DE--EG-GEGSVSLKAHNFDQYSHQYPILVVNFFAPWCYWSN
P.trichocarpaPDI-C2      120 FHSGPVLHHINHGDEV--HE--EG-SEGSVSLKAHNFDQYTHQYPILVVNFYAPWCYWSN
P.patensPDI-C            120 FHAGSVPNIKSHGDEDVGEDMFEHLGEGAVELNKNTFDVYAQQFSVLVVNFYAPWCPWSN
S.moellendorffiiPDI-C    120 FHPGPIPNPTSHGDDD--HE-----GEGAHVLTSSTFDEYARRYSVLVVNFYAPWCIWSA
C.subellipsoideaPDI-C    121 VHDDLSNVDIKYDEPS-------VSQDFALPLSKDSFKATLEAYSIVVVNFYAPWCPWCQ
C.reinhardtiiPDI-C       121 VEDTAHKVGPKYDAEGHFDE--EPDIDITVPLSHENFEATLARYPIAVINFYAPWCHWCQ
V.carteriPDI-C           121 VEDSSHKPGPKYDEEGRFDD--EPDIDITVPLSHVNFEATLARYPIVVVNFFAPWCHWCQ

                             ----------------->    BLOCK#3------------------------------
B.distachyonPDI-C        175 RLKPSWEKTAKIIKERYDPEMDGRILLAKVDCTEEGELCKRHHIQGYPSIRIFRKGSDMK
O.sativaPDI-C            175 RLKPSWEKTAKIMRERYDPEMDGRIILAKVDCTEEIDLCRRHHIQGYPSIRIFRKGSDLK
E.salsugineumPDI-C1      175 RLKPSWEKAANIIKQRYDPETDGRVLLGNVDCTEEAALCRRNHIQGYPSIRIFRKGSDLR
A.thalianaPDI12          175 RLKPSWEKAANIIKQRYDPEADGRVLLGNVDCTEEPALCKRNHIQGYPSIRIFRKGSDLR
C.rubellaPDI-C1          175 RLKPSWEKAANIIKQKYDPETDGRVLLGNVDCTEEPALCKRNHIQGYPSIRIFRKGSDLR
E.salsugineumPDI-C3      176 RLKPSWEKAAEITRQKYDPEDDGRVLLGSVDCTEEPTLCKRNHIQGYPSIRIFRKGSDLR
A.thalianaPDI13          176 RLKPSWVKASQITRERYNPGTDDRVLLGSVDCTEEPTLCKSNHIQGYPSIRIFRRGSGLR
C.rubellaPDI-C3          175 RLKPSWEKASQITRERYDPETDDRVLLGSVDCTKESTLCKSNHIQGYPSIRIFRKGSDLR
E.salsugineumPDI-C2      175 LLKPSWEKAAKQIKERYDPEMDGRVILAKVDCTVESDLCRRNHIQGYPSIRIFRKGSDLR
A.thalianaPDI7           175 LLKPSWEKAAKQIKERYDPEMDGRVILAKVDCTQEGDLCRRNHIQGYPSIRIFRKGSDLK
C.rubellaPDI-C2          175 LLKPSWEKAAKQIKERYDPEMDGRVIVAKVDCTQEGDLCRRNHIQGYPSIRIFRKGSDLK
S.lycopersicumPDI-C      175 RLKPSWEKAANIIRERYDRESDGRILVAKVDCTEEVDLCRRNHIQGYPSIRIFRKGTDVR
S.tuberosumPDI-C         175 RLKPSWEKAANIIRERYDRESDGRILVAKVDCTEEVDLCRRNHIQGYPSIRIFRKGSDVR
M.truncatulaPDI-C1       175 RLRPSWEKTAKIIRERYDPEMDGRILLGKVDCTKEADLCRRHHIQGYPSIRIFRKGSDVR
P.vulgarisPDI-C1         175 RLRPSWEKASKIIKERYDPETDGRIVMGRVDCTQNGELCRSHHVQGYPSIRIFRKGSDVR
G.maxPDI-C1              175 RLKPSWEKAAKIIKERYDPEMDGRIILGRVDCTEDGDLCRSHHIQGYPSIRIFRKGSDVR
G.maxPDI-C2              175 RLKPSWEKTAKIIKERYDPEMDGRIILGRVDCTEDGDLCRSHHIQGYPSIRIFRKGSDVR
M.truncatulaPDI-C2       175 RLKPSWEKAAKIMRERYDPEMDGRILLAKVDCTQEGDLCRRHHIQGYPSIRIFRKGSDVR
P.vulgarisPDI-C2         175 RLKPSWEKTAKIMKERYDPEMDGRIIMGKVDCTLEGDLCRRNHIQGYPSIRIFRKGSDLR
G.maxPDI-C3              175 RLKPSWEKTAKIMKERYDPEMDGRIILAKVDCTQEGDLCRRNHIQGYPSIRIFRKGSDLR
G.maxPDI-C4              175 RLKPSWEKTAKIMKERYDPEMDGRIILAKVDCTQEGDLCRRNHIQGYPSIRIFRKGTDLR
V.viniferaPDI-C          175 RLKPSWEKAAKIIRERYDPELDGRIVMAKVDCTEEGELCRRHHIQGYPSIRIFRKGSDVR
P.trichocarpaPDI-C1      175 RLKPSWEKAAKIIRERYDPEMDGRILLAKVDCTEEGDLCRRNHIQGYPSIRIFRKGSNLR
P.trichocarpaPDI-C2      175 RLKPSWEKAAKIIRERYDPEIDGRILLAKVDCTEEGDLCRRNHIQGYPSIRIFRKGSDLR
P.patensPDI-C            180 KLKASWEKAAKIIADKYNPEMDGRILLAKVDCTVNVELCRSHHIQGYPSIRIFRKGHDVR
S.moellendorffiiPDI-C    173 RLKPSWDKAAGIIAEKYHPDT-GRILLGKVDCTDNNDLCRKHHIQGFPSIRIFHKGHDLK
C.subellipsoideaPDI-C    174 RLEPTWEAVTQEVHTKY-PDADGRIRFAKVDCTTEVDLCREHQITGFPSIRVFRSGHDEV
C.reinhardtiiPDI-C       179 RLEPTWEAATKEVHDKY-PEWDGRVRFAKVDCTAEVDLCRQHFIQGFPSIRVFRKGHDDI
V.carteriPDI-C           179 RLEPTWEAATKEVHDKY-PEWDGRIRFAKVDCTQEMELCRTHFIQGFPSIRVFRKGHDDI
```

BLOCK#4

```
B.distachyonPDI-C       235 ENQGHHDHESYYGERDTDSLVAAMETYVGNLPKEAHMLALDDKSNKTVDPAKRPAPMTSG
O.sativaPDI-C           235 ENQGHHDHESYYGDRDTESLVAAMETYVANIPKDAHVLALEDKSNKTVDPAKRPAPLTSG
E.salsugineumPDI-C1     235 EDHGHHEHESYYGDRDTDSIVKMVDGLVAPIHPETHKLALDGISNDTAKNLKK-APVTGG
A.thalianaPDI12         235 EDHGHHEHESYYGDRDTDSIVKMVEGLVAPIHPETHKVALDGKSNDTVKHLKK-GPVTGG
C.rubellaPDI-C1         235 EDHGHHEHESYYGDRDTKSIVKMVDELVAPIHPETHKVALDGKSNDTVKHLKK-APVTGG
E.salsugineumPDI-C3     236 EDHGHHEHESYYGDRDTESLLKMVEELLKPIKKEDHKLPLDGISVNTASSIKK-APVSGG
A.thalianaPDI13         236 EDHGNHEHESYYGDRDTDSLVKMVEELLKPIKKEDHKLALDGKSDNAASTFKK-APVSGG
C.rubellaPDI-C3         235 DDHGHHDHESYYGDRDTDSLVKMVEELLKPIKKEDHKLALDGKSENAASTVKK-APVSGG
E.salsugineumPDI-C2     235 DDNAHHDHESYYGDRDTESLVKMVIGLVEPIHLEPQRLALEDKSDNASKILKK-APSTGG
A.thalianaPDI7          235 DDNAHHDHESYYGDRDTESLVKMVVSLVEPIHLEPHNLALEDKSDNSSRTLKK-APSTGG
C.rubellaPDI-C2         235 DDNAHHEHESYYGDRDTESLVKMVVSLVEPIHLEPHNLALEDKSGNSSKTLKK-APSTGG
S.lycopersicumPDI-C     235 DDHGHHDHESYYGDRDTDSLVKMMEDLVAPIKLDSQTITSDNSSTKLETGLKRPAPVTGG
S.tuberosumPDI-C        235 DDHGHHDHESYYGDRDTDSLVKMMEDLVAPIKLDSQMISSDNSSTKLETGLKRPAPVTGG
M.truncatulaPDI-C1      235 SDHGHHEHESYYGDRDTDSLVKTMENILASFPSEYYKLALEDKL-NVTEDSKRPAPSSGG
P.vulgarisPDI-C1        235 TDHGHHDHESYYGDRDTDSLVKTMDNLVASLPTESQKSHSGDKS-NLASHIKRPAPSSGG
G.maxPDI-C1             235 SNHGHHDHESYYGDRDTDSLVKTMENLVASLPSESQKLPLEDKS-DVAKNTERPAPSTGG
G.maxPDI-C2             235 SDHGHHDHESYYGDRDTDSLVKTMENLVASLPSESQKLPLEDKS-NVATNTKRPAPSTGG
M.truncatulaPDI-C2      235 SDHGHHEHESYYGDRDTESLVKTMETLVASLPTGSQHLALEDKS----NGTKRPAPSTGG
P.vulgarisPDI-C2        235 SEHGHHEHESYYGDRDTESLVEFMENLVTSLPTASQKPALEDKS-NATDNAKRPGPSAGG
G.maxPDI-C3             235 SEHGHHEHESYYGDRDTESLVKFMEDLVTSLPTESQKLALEDKS-NAADNAKRPAPSAGG
G.maxPDI-C4             235 SEHGHHEHESYYGDRDTESLVKFMEDLVTSLPTESQKLALEDKS-NASDNAKRPAPSAGG
V.viniferaPDI-C         235 DDHGHHDHESYYGDRDTDTLVTTMETLVAPIPLESQRLALENKSDSTADHIKRPAPRTGG
P.trichocarpaPDI-C1     235 EDHGRHDHESYYGDRDTESLVKTMEALVAPIAMESQRQALEHKPENATQHVKRPAPSAGG
P.trichocarpaPDI-C2     235 DDHGHHDHESYYGDRDTDSLVKTMEGLVAPIAMESQRHALEHKPENATEHVKRPAPSAGG
P.patensPDI-C           240 DEHGRHDHESYYGERDTESLVAFMVELVPPATVDGKFQLEDKSSITVNATIKRPAPKAGG
S.moellendorffiiPDI-C   232 DEHGHHEHDSYYGERDTDSLVKAMEALVP----KETTLALEDKT---NGTVKRPAPRAGG
C.subellipsoideaPDI-C   233 NVHGVKEHESYRGDRTQASLLAFADNLAPSAGQPHHYIR----------GVTR-MAKTSG
C.reinhardtiiPDI-C      238 YIGGMHEHEAYMGDRTKDALVAFADSLVPSAGQPHRKLA----------GLSA-APKTPG
V.carteriPDI-C          238 VIGGMHEHESYMGDRTKEALVAFADSLVPSAGQPHRKHA----------ALSA-APKTPG
```

```
B.distachyonPDI-C       295 CRVEGFVRVKKVPGSVIISARSGSHSFDPSQINVSHYVTQFSFGNRLSPNMFSELKRLIP
O.sativaPDI-C           295 CRIEGFVRVKKVPGSVVISARSGSHSFDPSQINVSHYVTQFSFGKRLSAKMFNELKRLTP
E.salsugineumPDI-C1     294 CRVEGYVRVKKVPGNLVISAHSGAHSFDSTQMNMSHVVTHLSFGRMISPRLLTDMRRLLP
A.thalianaPDI12         294 CRVEGYVRVKKVPGNLVISAHSGAHSFDSSQMNMSHVVSHFSFGRMISPRLLTDMKRLLP
C.rubellaPDI-C1         294 CRVEGYVRVKKVPGNLVISAHSGAHSFDSSQMNMSHVVSHLSFGRMISPRLLTDMKRLLP
E.salsugineumPDI-C3     295 CRIEGYVRAKKIPGEVVISAHSGAHSFDASQMNMSHFVTHLSFGKLISERLLTDMKRLLP
A.thalianaPDI13         295 CRIEGYVRAKKVPGELVISAHSGAHSFDASQMNMSHIVTHLTFGTMVSERLWTDMKRLLP
C.rubellaPDI-C3         294 CRIEGYVRAKKVPGELVISAHSGAHSFDASQMNMSHIVTHLTFGTMVSERLWTDMKRLLP
E.salsugineumPDI-C2     294 CRIEGYMRVKKVPGNLMVSARSGSHSFDSSQMNMSHVVNHLSFGRRILPQTFADLKRLSP
A.thalianaPDI7          294 CRVEGYMRVKKVPGNLMVSARSGSHSFDSSQMNMSHVVNHLSFGRRIMPQKFSEFKRLSP
C.rubellaPDI-C2         294 CRIEGYMRVKKVPGNLMVSARSGSHSFDSSQMNMSHVVNHLSFGRRILPQKFSELKRLSP
S.lycopersicumPDI-C     295 CRIEGFVRVKKVPGNLVISARSAAHSFDASQMNMSHVISSFSFGKTITPKVMSDIKILLP
S.tuberosumPDI-C        295 CRIEGFVRVKKVPGNLVISARSAAHSFDASQMNMSHVISSFSFGKTITPKVMSDIKILLP
M.truncatulaPDI-C1      294 CRIEGYVRVKKVPGNLIISARSDAHSFDASQMNMSHAVHHLSFGKKLSPKLMSDVQRLIP
P.vulgarisPDI-C1        294 CRIEGYVRVKKVPGNLIISARSDAHSFDASQMNMSHVVNHLSFGRKVSPRVMSDVKRLIP
G.maxPDI-C1             294 CRIDGYVRVKKVPGNLIFSARSNAHSFDASQMNMSHVINHLSFGRKVSPRVMSDVKRLIP
G.maxPDI-C2             294 CRIDGYVRVKKVPGNLIISARSNAHSFDASQMNMSHVINHLSFGRKVSLRVMSDVKRLIP
M.truncatulaPDI-C2      291 CRVEGYVRVKKVPGSLVVSARSDAHSFDASQMNMSHVINHLSFGKKVTPRAMIDVKHWIP
P.vulgarisPDI-C2        294 CRIEGYVRVKKVPGNLIISARSDAHSFDASQMNMSHVVNNLSFGKKVTPRAMSDVKLLIP
G.maxPDI-C3             294 CRVEGYVRVKKVPGNLIISARSDAHSFDASQMNMSHVINNLSFGKKVTPRAMSDVKLLIP
G.maxPDI-C4             294 CRVEGYVRVKKVPGNLIISARSDAHSFDASQMNMSHFINNLSFGKKVTPRAMSDVKLLIP
V.viniferaPDI-C         295 CRIEGFVRVKKVPGNLVISARSGSHSFDPSQMNMSHVISHLSFGRKIAPRVMSDMKRVLP
P.trichocarpaPDI-C1     295 CRIEGYVRVKKVPGNLMISALSGAHSFDSKQMNLSHVISHFSFGMKVLPRVMSDVKRLLP
P.trichocarpaPDI-C2     295 CRIEGYVRVKKVPGNLVISARSGAHSFDSAQMNLSHVISHFSFGMKVLPRVMSDVKRLIP
P.patensPDI-C           300 CRVEGFVRVKKVPGELMISAHSGSHSFDATSMNMTHYVGFFSFGRKTSWRSVHWVNEMLP
S.moellendorffiiPDI-C   285 CRIEGFIRAKKVPGNIIISAHSGSHSFDASAMNMTHYVSQFTFGRELNFWMRRELYRIYP
C.subellipsoideaPDI-C   282 CALSGFVLVKKVPGALHFLAKSPGHSFDYQAMNMSHVVNYLYFGNKPSPRRHQSLAKLHP
C.reinhardtiiPDI-C      287 CNLAGFVMVKKVPGTVHFVARSEGHSFDHTWMNMTHMIHSFHVGTRPSPRKYQQLKRLHP
V.carteriPDI-C          287 CNLAGFVMVKKVPGTLTVVARSEGHSFDHTWMNMTHLVHTFHVGTRPSPRKYQQLKRLHP
```

```
                                                    BLOCK#5------------>          BLOCK#6
B.distachyonPDI-C      355 YVGGHHD----RLAGQSYIVKHGDNNANVTIEHYLQIVKTELVTLRSSKELKVFEEYEYT
O.sativaPDI-C          355 YVGGHHD----RLAGQSYIVKHGDVNANVTIEHYLQIVKTELVTLRSSKELKLVEEYEYT
E.salsugineumPDI-C1    354 YLGQSHG----RLDEKAFINQ-HEFGANVTIEHYLQIVKTEVITRKSGQEHSLIEEYEYT
A.thalianaPDI12        354 YLGLSHD----RLDGKAFINQ-HEFGANVTIEHYLQTVKTEVITRRSGQEHSLIEEYEYT
C.rubellaPDI-C1        354 YLGQSHD----RLDGKAFINQ-HEFGANVTIEHYLQIVKTEVITRRSGLEQAVIEEYEYT
E.salsugineumPDI-C3    355 YLGQSHD----RLNGKWFLNQ-GQFAANVTIEHYLQIVKTEVVSRRFGQEHSLIEEYEYT
A.thalianaPDI13        355 YLGQSYD----RLNGKSFINE-RQLDANVTIEHYLQIIKTEVISRRSGQEHSLIEEYEYT
C.rubellaPDI-C3        354 YLGQSHD----RLNGKSFINQ-RQLDANVTIEHYLQIVKTEVISRRFGREHSLIEEYEYT
E.salsugineumPDI-C2    354 YLGRSHD----RLDGRPFINQ-RDLGPNVTIEHYLQIVKTEVVKSN---GQALIEEYEYT
A.thalianaPDI7         354 YLGLSHD----RLDGRSFINQ-RDLGPNVTIEHYLQIVKTEVVKSN---GQALVEAYEYT
C.rubellaPDI-C2        354 YLGRNHD----RLDGRPFINQ-RDLGPNVTIEHYLQIVKTEVVKSN---GQVLIEEYEYT
S.lycopersicumPDI-C    355 HLGRSHD----RLNGNSYVTNPRDSTENVTIEHFLQVVKTEVMTR----SYKLVEEYEYT
S.tuberosumPDI-C       355 HLGRSHD----RLNGNSYVTNPRDSTENVTIEHFLQVVKTEVMTR----SYKLVEEYEYT
M.truncatulaPDI-C1     354 YVGNSHD----RLDGLSFINS-HDFGANVTLEHYLQIVKTEVITRQ---GYQLVEEYEYT
P.vulgarisPDI-C1       354 YVGSSHD----RLNGLSFINT-RDFGANVTMEHYLQIVKTEVITRK---DHKLVEEYEYT
G.maxPDI-C1            354 YVGSSHD----RLNGRSFINT-HDLGANVTMEHYLQIVKTEVITRK---DYKLVEEYEYT
G.maxPDI-C2            354 YVGSSHD----RLNGRSFINT-HDLGANVTIEHYLQIVKTEVITRK---EYKLVEEYEYT
M.truncatulaPDI-C2     351 YLGINHD----RLNGRSFINT-RDLEGNVTIEHYIQVVKTEVITRK---GYKLIEEYEYT
P.vulgarisPDI-C2       354 YLGSSHD----RLSGRSFSNT-RDFGANVTIEHYIQIVKTEVVNRK---GYKLIEEYEYT
G.maxPDI-C3            354 YIGSSHD----RLNGRSFINT-RDLGANVTIEHYIQIVKTEVVTRK---GYKLIEEYEYT
G.maxPDI-C4            354 YIGSSHD----RLNGRSFTNT-HDLGANVTIEHYIQIVKTEVVTRN---GYKLIEEYEYT
V.viniferaPDI-C        355 YIGGSHD----RLNGRSYISHPSDSNANVTIEHYLQVVKTEVITTR---DHKLVEEYEYT
P.trichocarpaPDI-C1    355 YIGRSHD----KLNGRSFINH-RDVGANVTIEHYLQVVKTEVVTRRSSSERKLIEEYEYT
P.trichocarpaPDI-C2    355 HIGRSHD----KLNGRSFINH-RDVGANVTIEHYLQVVKTEVVTRRSSAEHKLIEEYEYT
P.patensPDI-C          360 ALDSNID----RLTGQVFPSE----YENITHDHYLQVVKTEVITLHRKQDLRVLEQYDYT
S.moellendorffiiPDI-C  345 HLASVYDTVEANLTGRIYVSQ----HENITHDHYLQVVKTEVVSLRKRKEFSLLEQYDYT
C.subellipsoideaPDI-C  342 -AGLSDDWAD-KLAGQDFFSR----AAKATFEHYMQVVLTTIEPSKHRPELSY-DAYEYT
C.reinhardtiiPDI-C     347 -AGLTADWAD-KLHDQLFVSE----HTQSTHEHYLQVVLTTIEPRHSRHTGNY-DAYEYT
V.carteriPDI-C         347 -AGLTHDWAD-QLRDQFFLSE----HPQSTHEHYLQIVLTSIEPRRSRHSGNY-DAYEYT

                           ------------------------------------------------------------
B.distachyonPDI-C      411 AHSSLVHSFYVPVVKFHFEPSPMQVLVTELPKSFSHFITNVCAIIGGVFTVAGILDSILH
O.sativaPDI-C          411 AHSSLVHSFYVPVVKFHFEPSPMQVLVTELPKSFSHFITNVCAIIGGVFTVAGILDSIFH
E.salsugineumPDI-C1    409 AHSSVAQTYYLPVAKFHFELSPMQILITENPKSFSHFITNLCAIIGGVFTVAGILDSIFH
A.thalianaPDI12        409 AHSSVAQTYYLPVAKFHFELSPMQILITENPKSFSHFITNLCAIIGGVFTVAGILDSIFH
C.rubellaPDI-C1        409 AHSSVAQTYYLPVAKFHFELSPMQILITEDPKSFSHFITNLCAIIGGVFTVAGILDSIFH
E.salsugineumPDI-C3    410 AHSNVAHSYYYPVAKFHFELSPMQVLISENPKSFSHFITNVCAIIGGVFTVAGILDSIFQ
A.thalianaPDI13        410 AHSSVARSYHYPEAKFHFELSPMQVLISENPKSFSHFITNVCAIIGGVFTVAGILDSIFQ
C.rubellaPDI-C3        409 AHSNVAHSYHYPEAKFHFELSPMQVLISENPKSFSHFITNVCAIIGGVFTVAGILDSIFQ
E.salsugineumPDI-C2    406 AHSSVAQSYYLPVAKFHFELSPMQVLITETSKSFSHFITNVCAIIGGVFTVAGILDSILH
A.thalianaPDI7         406 AHSSVAHSYYLPVAKFHFELSPMQVLITENSKSFSHFITNVCAIIGGVFTVAGILDSILH
C.rubellaPDI-C2        406 AHSSVAQSYYLPVAKFHFELSPMQVLITENSKSFSHFITNVCAIIGGVFTVAGILDSILH
S.lycopersicumPDI-C    407 AHSSLVHSLHIPVAKFHYEPSPMQVLITENPKSFSHFLTNVCAIIGGVFTVAGILDSILH
S.tuberosumPDI-C       407 AHSSLVHSLHIPVVKFHYEPSPMQVLITENPKSFSHFLTNVCAIIGGVFTVAGILDSILH
M.truncatulaPDI-C1     406 AHSSLAHSLHVPVARFHLQLSPMQVLITEDHKSFSHFITNVCAIVGGVFTVAGITESILH
P.vulgarisPDI-C1       406 AHSSVAQSLHIPVAKFHLELSPMQVLITENPKSFSHFITNVCAIIGGVFTVAGILDSILH
G.maxPDI-C1            406 AHSSVAQSLHIPVAKFHLELSPMQVLITENQKSFSHFITNVCAIVGGIFTVAGIMDAILH
G.maxPDI-C2            406 AHSSVAQSLHIPVAKFHLELSPMQVLITENQKSFSHFITNVCAIIGGIFTVAGIMDAIFH
M.truncatulaPDI-C2     403 AHSSVAHSVNIPVARFHLELSPMQVLITENQKSFSHFITNVCAIIGGVFTVAGILDSILH
P.vulgarisPDI-C2       406 AHSSVAHSVDIPVAKFHIELSPMQVLITENQRSFSHFITNVCAIIGGIFTVAGILDSILH
G.maxPDI-C3            406 AHSSVAHSLDIPVAKFHLELSPMQVLITENQRSFSHFITNVCAIIGGVFTVAGILDSILH
G.maxPDI-C4            406 AHSSVAHSVDIPAAKFHLELSPMQVLITENQRSFSHFITNVCAIIGGVFTVAGILDSILH
V.viniferaPDI-C        408 AHSSLVQSLYIPVAKFHFELSPMQVLVTENRKSFWHFITNVCAIIGGVFTVAGILDSVLH
P.trichocarpaPDI-C1    410 AHSSLSQTVYMPTAKFHFELSPMQVLITENSKSFSHFITNVCAIIGGVFTVAGILDSILH
P.trichocarpaPDI-C2    410 AHSSLAQTVYMPTAKFHFELSPMQVLITENPKSFSHFITNVCAIIGGVFTVAGILDSILH
P.patensPDI-C          412 AHSNMIQSTKVPVVKFHYELSPMQVLVKENPKSFSHFLTNLCAIIGGVFTVAGIIDSMLH
S.moellendorffiiPDI-C  401 SHSNTIQNTNVPVAKFHYELSPMQVLVKENPKSFSHFITNVCAIIGGVFTVAGIVDSMLH
C.subellipsoideaPDI-C  395 VHSHTYDTADIPAAKFTYDLSPIQILVSEKRRAWYHFVTTTCAIIGGVFTVAGIVDGLVH
C.reinhardtiiPDI-C     400 AHSHSYQSDSIPSARFTYDLSPIQILVHETSKPWYQFLTTSCAIIGGVFTVAGILDALLY
V.carteriPDI-C         400 AHSHTYQSDAIPSARFTYDLSPIQILVQETARPWYQFLTTSCAIIGGVFTVAGILDALLY
```

```
B.distachyonPDI-C        471 NTLRLVKKVELGKDI
O.sativaPDI-C            471 NTLRLVKKVELGKNI
E.salsugineumPDI-C1      469 NTIRLVKKVELGKNI
A.thalianaPDI12          469 NTVRLVKKVELGKNI
C.rubellaPDI-C1          469 NTIRLIKKVELGKNI
E.salsugineumPDI-C3      470 NTIRLVKKVELGKNI
A.thalianaPDI13          470 NTVRMVKKIELGKNI
C.rubellaPDI-C3          469 NTVRLVKKIELGKNI
E.salsugineumPDI-C2      466 QTMTLMKKIELGKNF
A.thalianaPDI7           466 HSMTLMKKIELGKNF
C.rubellaPDI-C2          466 HTMTLMKKIELGKNF
S.lycopersicumPDI-C      467 NTMRMVKKVELGKNF
S.tuberosumPDI-C         467 NTMRMVKKVELGKNF
M.truncatulaPDI-C1       466 NTIRLMRKVELGKNF
P.vulgarisPDI-C1         466 NTIRLMKKVELGKNF
G.maxPDI-C1              466 NTIRLMKKVELGKNF
G.maxPDI-C2              466 NTIRLMKKVELGKNF
M.truncatulaPDI-C2       463 NTIKAMKKIEIGKNF
P.vulgarisPDI-C2         466 NTIRMMKKVELGKNF
G.maxPDI-C3              466 NTIRMVKKIELGKNF
G.maxPDI-C4              466 NTIRMMKKVELGKNF
V.viniferaPDI-C          468 NTMRLMKKIELGKNF
P.trichocarpaPDI-C1      470 HTVRMMKKVELGKNF
P.trichocarpaPDI-C2      470 NTFRMMKKVELGKNF
P.patensPDI-C            472 NAMHIMKKVELGKQY
S.moellendorffiiPDI-C    461 GAMRMVKKIELGKQF
C.subellipsoideaPDI-C    455 TGARFAKKVELGKHT
C.reinhardtiiPDI-C       460 QSFKVVKKLNLGKQG
V.carteriPDI-C           460 QSFKVVKKLNLGKQG
```

Multiple sequence alignment for NJ tree in Online Reference 4

```
                                                BLOCK#1------------------------------------------
C.subellipsoideaPDI-C     1 ----MARVL-QKLRSVDFYRKIPNDLTEATLAGAGISLVAAFTIVVLLTAELSSFLAIET
C.reinhardtiiPDI-C        1 ----MVRLF-SRLKAIDFFKKIPSDLTEATLTGAWLSIVAAVLMILLFVAELSAFLSTTT
V.carteriPDI-C            1 ----MARLF-SKLKAIDFFKKIPSDLTEATLTGAWISILAAVIMVFLFTAEMMSFLSTTT
K.flaccidumPDI-C          1 ----MNRVL-GRLRSVDFYRRIPRDLTEATLAGGTLSVVASIAMVLLFGMELQAYLTTSA
B.rapaPDI-C1              1 ------MVSPTKLKSMDFYRKIPRDLTEASLSGAGLSIVAALVMMLLFGMELSSYLAVNT
B.rapaPDI-C2              1 ------MVSPTKLKSVDFYRKIPRDLTEASLSGAGLSIVAALVMMLLFGMELSSYLEVTT
E.salsugineumPDI-C1       1 ------MVSSTKIKSVDFYRKIPRDLTEASLSGAGLSIVAALVMMLLFGMELSSYLEVST
A.thalianaPDI12           1 ------MVSSTKLKSVDFYRKIPRDLTEASLSGAGLSIVAALFMMFLFGMELSSYLEVNT
C.rubellaPDI-C1           1 ------MVSSTKLKSVDFYRKIPRDLTEASLSGAGLSIVAALFMVFLFGMELSSYLEVNT
B.rapaPDI-C5              1 ------MVSTTKIKSVDFYRKIPRDLTEASLSGAGLSIIAALAMMFLFGMELSTYLAVTT
A.thalianaPDI13           1 ------MVSTSKIKSVDFYRKIPRDLTEASLSGAGLSIVAALAMLFLFGMELSSYLAINT
C.rubellaPDI-C3           1 ------MVSTTKIKSVDFFRKIPRDLTEASLSGAGLSIVAALAMLFLFGMELSSYLAVNT
B.rapaPDI-C6              1 ------MVSTTKIKSVDFYRKIPRDLTEASLSGAGLSIIAALAMVFLFGMELSTYLAVTT
E.salsugineumPDI-C3       1 ------MVSTTKIKSVDFYRKIPRDLTEASLSGAGLSIVAALAMVFLFGMELSNYLAVST
S.bicolorPDI-C            1 ------MISSSKLKSVDFYRKIPRDLTEASLSGAGLSIVAALAMVFLFGMELSNYLAVNT
Z.maysPDI-C               1 ------MISSSKLKSVDFYRKIPRDLTEASLSGAGLSIVAALAMVFLFGMELSSYLAVNT
B.distachyonPDI-C         1 ------MISSSKLKSVDFYRKIPRDLTEASLSGAGLSIVAALAMVFLFGMELSSYLAVNT
O.sativaPDI-C             1 ------MISSSKLKSVDFYRKIPRDLTEASLSGAGLSIVAALAMVFLFGMELSNYLAVNT
B.rapaPDI-C4              1 ------MISPRKIKSVDFYRKIPRDLTEASLSGAGLSIVAALSMLLLFGMELSSYLTVST
A.thalianaPDI7            1 ------MVSTSKIKSVDFYRKIPRDLTEASLSGAGLSIIAALSMIFLFGMELNNYLAVST
C.rubellaPDI-C2           1 ------MVSTSKIKSVDFYRKIPRDLTEATLSGAGLSIIAALSMLFLFGMELNNYLAVST
B.rapaPDI-C3              1 ------MVSTSRIKSVDFYRKIPRDLTEATLSGAGLSIVAALSMLFLFGMELNNYLAVST
E.salsugineumPDI-C2       1 ------MVSTSKIKSVDFYRKIPRDLTEATLSGAGLSIVAALSMLFLFGMELNNYLAVST
S.lycopersicumPDI-C       1 ------MVSTSKIKSVDFYRKIPRDLTEASLSGAGLSIVAALCMMFLFGMELNNYLTVST
S.tuberosumPDI-C          1 ------MVSTSKIKSVDFYRKIPRDLTEASLSGAGLSIVAALSMMFLFGMELNNYLTVST
M.truncatulaPDI-C1        1 ------MTTASKIKSVDFYRKIPRDLTEASLSGAGLSIVAALAMMFLFGMELNEYLSVHT
P.vulgarisPDI-C1          1 ------MISSSKLKSVDFYRKIPRDLTEASLSGAGLSIVAALCMVFLFGMELNSYLSVST
G.maxPDI-C1               1 ------MISSSKIKSVDFYRKIPRDLTEASLSGAGLSIVAALAMIFLFGMELNSYLSVTT
G.maxPDI-C2               1 ------MISSSKIKSVDFYRKIPRDLTEASLSGAGLSIVAALAMIFLFGMELNSYLSVST
M.truncatulaPDI-C2        1 ------MLSASKLKSVDFYRKIPRDLTEASLSGAGLSILAALAMMFLFGMELSNYFAVTT
P.vulgarisPDI-C2          1 ------MISATKLKSVDFYRKIPRDLTEASLSGAGLSIVAALSMMFLFGMELSNYLTVST
G.maxPDI-C3               1 ------MISATKLKSVDFYRKIPRDLTEASLSGAGLSIVAALVMMFLFGMELSSYMSVST
G.maxPDI-C4               1 ------MISATKLKSVDFYRKIPRDLTEASLSGAGLSIVAALAMMFLFGMELSSYLSVST
V.viniferaPDI-C           1 ------MVSTSKIKSVDFYRKIPRDLTEASLSGAGLSVIAALSMMFLFGMELSNYLSVST
C.sativusPDI-C            1 ------MISSTKLKSVDFYRKIPRDLTEATLSGAGLSIVAALSMVFLFGMELSNYLSVST
P.persicaPDI-C            1 ------MISTGKIKSVDFYRKIPRDLTEASLSGAGLSIIAALAMMFLFGMELNSYLAFST
T.cacaoPDI-C              1 ------MISSSKIKSVDFYRKIPRDLTEASLSGAGLSIVAALAMMFLFGMELSNYLTVST
P.trichocarpaPDI-C1       1 ------MVSTNKLKSVDFYRKIPRDLTEASLSGAGLSIVAALAMMFLFGMELNNYLTVNT
P.trichocarpaPDI-C2       1 ------MVSTNKLKSVDFYRKIPRDLTEASLSGAGLSIVAALAMVFLFGMELNNYLTVNT
S.moellendorffiiPDI-C     1 ------MTTASKIKSIDFYRKIPRDLTEASLSGAGLSLIAAFAMIFLFGMELNNYLTVSS
P.patensPDI-C             1 ------MVSTSKLKSIDFYRKIPRDLTEASLSGAGLSIIAALTMVFLFGMELSAYLSTTT
C.reinhardtiiERV-A        1 --MSGGGFL-GKLKALDAYPKINEDFFTKTMSGGIITIVSSVVMVLLFLSELRLFLTTSS
V.carteriERV-A            1 MSNSGGGFL-SKLKALDAYPKINEDFFTKTMSGGIITIVASVVMVLLFLSELRLYMTTQS
C.subellipsoideaERV-A     1 ----MEGIV-SKLKNLDAYPKVNEDFFQRTLSGGIITIGSSIIMLCLFLSELSLFMKITT
K.flaccidumERV-A          1 ----MGDVL-NYLKKLDAYPKINEDFYSRTLSGGLITLVSSIFIVLLFFTELGLFLTTKT
B.distachyonERV-A3        1 ----MDGFL-QKLKGLDAYPKVNEDFYKRTLSGGVVTLVSAVVMLLLFISETSSYLNSAT
O.sativaERV-A3            1 ----MEGFL-QKLKGLDAYPKVNEDFYKRTLSGGVVTVVASVVMLLLFVSETRSYFYSAT
Z.maysERV-A3              1 ----MDAFL-HRLKRLDAYPKVNEDFYKRTLSGGIVTLVAAVVMLLLFISETRSYFYSST
Z.maysERV-A2              1 ----MDAFL-QRLKRLDAYPKVNEDFYKRTLSGGIVTLVAAVVMLLLFISETRSYFYSAT
S.bicolorERV-A3           1 ----MDAFL-QRLKRLDAYPKVNEDFYKRTLSGGIVTLVAAVVMLLLFISETRSYFYSAT
P.patensERV-A1            1 ---MALQMI-QKLKSLDAYPKINEDFYSRTLSGGIITIISATFMVLLFFSELKLYLAAQV
P.patensERV-A4            1 -----MSFF-NKLKHLDAYPKISEDFYSRTLSGGLITLVSSVFMTLLFITEFRIYLSAQT
P.patensERV-A2            1 -----MAVF-NKLKQLDAYPKISEDFYSRTLSGGVITLVSTVFMFVLFVTEISLYLSAQT
P.patensERV-A3            1 -----MAIF-NKLKQLDAHPKISEDFYSRTLSGGVITLVSSIFMFLLFVTEFRIYLSAQT
S.bicolorERV-A2           1 -----MELW-SKLRNLDAYPKVNEDFYSRTLSGGLITILSSLAILLLFFSEIRLYLYSAT
B.distachyonERV-A2        1 -----MDLW-NKLRSLDAYPKVNEDFYSRTLSGGLITIASSLAILLLFFSEIRLYLYSAT
O.sativaERV-A2            1 -----MDLW-NKLRSLDAYPKVNEDFYSRTLSGGLITIASSLAILLLFLSEIRLYLYSAT
S.moellendorffiiERV-A     1 -----MQML-KKLQQLDAYPKINEDFHSRTLSGGVITVVSSIFMAILFITELKLFLLPGT
C.sativusERV-A3           1 ----MDAIF-NKLRNLDAYPKINEDFYRRTFSGGLITLASSFFMLFLFFSELRMYLHAKT
M.truncatulaERV-A2        1 ----MDKVF-NKLRNLDAYPKVNEDFYNRTLAGGVVTVVSAAVMLFLFISELRLYLYTVT
P.vulgarisERV-A2          1 ----MDKVF-NKLRNLDAYPKVNEDFYSRTLAGGVVTVVSAAVMLFLFFSELNLYLYTVT
G.maxERV-A3               1 ----MDKVF-NKLRNLDAYPKVNEDFYNRTLAGGVVTVVSAAVMLFLFFSELSLYLYTVT
G.maxERV-A4               1 ----MDKVF-NKLRNLDAYPKVNEDFYNRTLAGGVVTVVSAAVMLFLFFSELSLYLYTVT
S.lycopersicumERV-A2      1 ----MERVF-SKLRNLDAYPKINEDFYNRTLSGGIITLVASVIMLVLFVNELGLYIHSYT
S.tuberosumERV-A2         1 ----MERVF-SKLRNLDAYPKINEDFYNRTLSGGIITLVASIIMLVLFVNELGLYIHSYT
P.persicaERV-A2           1 ----MDSIL-QKLRNLDAYPKINDDFFSRTLSGGIITLASSLLILLLFFSELRLYLHTVT
T.cacaoERV-A2             1 ----MENVF-NKLRNLDAYPKVNEDFYSRTLSGGIITLVSSLAIFFLFFSEFRLYLHTVT
P.trichocarpaERV-A3       1 ----MEGIY-QKLRNLDAYPKINEDFYSRTLSGGLITLISSIIMLFLFFSEFSLYLHAVT
P.trichocarpaERV-A4       1 ----MDRIY-QKVRNLDAYPKINEDFYSRTLSGGLITLISSVLILFLFFSELSLYLHKVT
C.sativusERV-A1           1 ----MESLM-NKIRKLDAYPKISEDFYNRTLSGGFITIASSIIMFLLFFSELRLYVHTAT
V.viniferaERV-A2          1 ----MDRVF-QRLRNLDAYPKINEDFYSRTFSGGLITLISSIVMLFLFFSELRLYLHTVT
B.rapaERV-A4              1 --------M-NRLRNLDAYPKINEDFYKRTLSGGVITLVSSVVMLILFFSELRLYMHPVT
B.rapaERV-A3              1 --------M-NRLRNLDAYPKINEDFYNRTLSGGLITLFSSLLMLILFFSELRLYMHPVT
E.salsugineumERV-A2       1 ----MVGVM-NRLRNLDAYPKINEDFYRRTLSGGVITLVSSIVMLILFFSELQLYLHPVT
```

```
                                                  BLOCK#1--------------------------------------
A.thalianaERV-A2             1 ----MVGVM-NRLRNLDAYPKINEDFYRRTLSGGVITLASSIVMLILFFSELQLYIHPVT
C.rubellaERV-A2              1 ----MVGVM-NRLRNLDAYPKINEDFYRRTLSGGVITLVSSLLMLVLFFSELQLYIHPVT
B.rapaERV-A2                 1 ----MAGVM-NRLKNLDAYPKINDDFYSRTLSGGLITLASSLVMLILFFSELRLYLHPVT
E.salsugineumERV-A3          1 ----MPGVM-NRLRNLDAYPKINDDFYSRSLSGGLITLVSSIFMLILFFSELRLYLHPVT
B.distachyonERV-A1           1 ----MDGLM-SKLRNLDAYPKVNEDFYSRTLSGGVITLASSFVMLLLFVSELRLYLHAVT
O.sativaERV-A1               1 ----MEGLL-SKLRSLDAYPKVNEDFYSRTLSGGIITLASSVVMLLLFVSELRLYLHAVT
S.bicolorERV-A1              1 ----MDGLL-SKLRSLDAYPKVNEDFYSRTLSGGVITLASSVIMLLLFVSELRLYLHAVT
Z.maysERV-A1                 1 ----MDGLL-SKLRSLDAYPKVNEDFYSRTLSGGIITLVSSAVMLLLFVSELRLYLHAVT
S.lycopersicumERV-A1         1 ----MDSFV-SKIRSLDAYPKINEDFYSRTLSGGVITLASSIIMTLLFISELRLYLHAAT
S.tuberosumERV-A1            1 ----MDSFI-SKIRSLDAYPKINEDFYSRTLSGGVITLASSIIMTLLFISELRLYLHAAT
B.rapaERV-A1                 1 ----MAGIL-NKLRNLDAYPKINEDFYSRTLSGGVITLVSSVVMFLLFFSELRLYLHSVT
E.salsugineumERV-A1          1 ----MAGIL-NKLRNLDAYPKINEDFYSRTLSGGVITLFSSVVMFLLFFSELRLYLHTVT
A.thalianaERV-A1             1 ----MAGIL-NKLRNLDAYPKINEDFYSRTLSGGVITLLSSVVMFLLFFSELRLYLHTVT
C.rubellaERV-A1              1 ----MAGIL-NKLRNLDAYPKINEDFYSRTLSGGVITLLSSVVMFLLFFSELRLYLHTVT
V.viniferaERV-A1             1 ----MDNII-NKLRNLDAYPKINEDFYSRTLSGGVITLASSIFMLLLFISELRLYLHAVT
C.sativusERV-A2              1 ----MDNII-SKLRNLDAYPKINEDFYSRTLSGGVITLSSSILMLLLFISELRLYLHAVT
M.truncatulaERV-A1           1 ----MDSIM-NKLRNLDAYPKINEDFYSRTLSGGLITIVSSILMLLLFFSELRLYLHAAT
P.persicaERV-A1              1 ----MENMM-SRLRNLDAYPKINEDFYSRTLSGGVITLASSIVMLLLFLSELRLYLHAVT
P.vulgarisERV-A1             1 ----MEGIM-SKLRNLDAYPKINEDFYSRTLSGGVITLASSIIMLLLFFSELRLYLQSVT
G.maxERV-A1                  1 ----MDSIM-SKLRNLDAYPKINEDFYSRTLSGGVITLASSILMLLLFFSELRLYLHAVT
G.maxERV-A2                  1 ----MESII-SKLRNLDAYPKINEDFYSRTLSGGVITLASSILMLLLFYSELRLYLHAVT
T.cacaoERV-A1                1 ----MDGIM-NKLRNLDAYPKINEDFYSRTLSGGVITLVSSVVMFFLFFSELRLYLHAVT
P.trichocarpaERV-A1          1 ----MEGLM-SKLRNLDAYPKINEDFYSRTLSGGVITLASSVVMFLLFFSELRLYLHAVT
P.trichocarpaERV-A2          1 ----MDGLM-SKLRNFDAYPKINEDFYSRTLSGGVITLASSIVMFLLFFSELRLYLHAVT
C.reinhardtiiERV-B           1 ---MGKGFR---LSSLSAYVKPEAHLVNQTIHGALVTLCGVLLATLLTLHEIKSFYQMHR
V.carteriERV-B               1 ------MKF--KLSSLSAYVKPEAHLVQQTVHGALVTLCGILLAAMLFVHELGSFYRQHR
C.subellipsoideaERV-B        1 -----MKLK--SFNRFSAYARAESHLVQRTYFGAIVTVLGVILAIVLFANELREYTTPFS
K.flaccidumERV-B             1 -----MAFKIAALSRIDAYPRAESHLTQRTISGAAISIMGISLMVLLFVNELQFYMTPYT
P.patensERV-B                1 ---MRKEKW-QVIKNLDAFPRAEEHLLQKTSSGAAVSAIGLFIMGVLFFHELRFYLETVT
S.moellendorffiiERV-B        1 -----MGLK---MKNINAFAHADEHLTQKTVSGAILTIVGVSIILVLFAYEFKFYLSTNV
S.bicolorERV-B               1 -----MARI-PSLKSLNAFPHAEEHLLKKTYSGAVVTILGLLVMITLFVHELQFYLTTYT
Z.maysERV-B                  1 -----MARI-PSLKSLNAFPHAEEHLLKKTYSGAVVTIFGLLIMITLFVHELQFYLTTYT
B.distachyonERV-B            1 -----MGRI-PSLKNFNAFPHAEDHLLKKTYSGAIVTIFGLIIMFTLFVHELKFYLTTYT
O.sativaERV-B                1 -----MGRI-PSLKNFNAFPHAEDHLLKKTYSGAIVTIFGLIIMVTLFAHELKFYLTTYT
B.rapaERV-B2                 1 -----MGVK-QALRSIDAFPRAEDHLLQKTQSGAVVSIVGLLIMVILFLSELSYYLNTLT
B.rapaERV-B1                 1 -----MGVK-QALRSIDAFPRAEDHLLQKTQSGAVVSIVGLLIMATLFLHELSYYLNTLT
E.salsugineumERV-B           1 -----MGVK-QALKSIDAFPRAEDHLLQKTQSGAVVSIVGLIIMATLFLHELSYYLNTLT
A.thalianaERV-B              1 -----MGVK-QALRSIDAFPRAEDHLLQKTQSGAVVSIVGLLIMATLFLHELSYYLNTLT
C.rubellaERV-B               1 -----MGVK-QALRSIDAFPRAEDHLLQKTQSGAVVSIVGLLIMATLFLHELSYYLNTLT
M.truncatulaERV-B            1 -----MGMK-QVIKNLDAFPRTEDHLLQKTQSGALVSIIGLIIMATLFLHELGYYLTTYT
P.vulgarisERV-B              1 -----MGMK-QVIKNLDAFPRAEDHLLQKTQSGALVSVIGLIIMATLFVHELRYYLTTYT
G.maxERV-B1                  1 -----MGMK-QVIKNLDAFPRAEDHLLQKTQSGALVSVIGLIIMATLFVHELGYYLTTYT
G.maxERV-B2                  1 -----MGMK-QVIKNLDAFPRAEDHLLQKTQSGALVSVIGLIIMATLFVHELGYYLTTYT
C.sativusERV-B               1 -----MGLK-QTIKSLDAFPRAEEHLLQKTQTGAFVSVVGLVIMATLFLHELRYYLSTYT
S.lycopersicumERV-B1         1 -----MGVK-QVLRAMDAFPRAEEHLLQKTKSGAFVSIVGLVIMSTLFLHELSYYLNTIT
S.tuberosumERV-B1            1 -----MGVK-QVLRAMDAFPRAEEHLLQKTKSGAFVSIVGLVIMSTLFLHELSYYLNTIT
S.lycopersicumERV-B2         1 -----MGVK-QALRAIDAFPRAEEHLLLKTKFGAFVSIVGLLIMVTLFLHELSYYLNIYT
S.tuberosumERV-B2            1 -----MGVK-QALRAIDAFPRAEEHLLQKTKFGAFVSIVGLLIMVTLFLHELSYYLNIYT
P.persicaERV-B               1 -----MGVK-QALKSLDAFPRAEEHLLQKTQTGAVVSVVGLLIMATLFVHELRYYLTTYT
P.trichocarpaERV-B1          1 -----MGMK-QAIKKLDAFPRAEEHLLQKTQSGALVSIIGLVTMATLFYHELAYYLTTYT
P.trichocarpaERV-B2          1 -----MGVK-QAIKSLDAFPRAEEHLLQKTQSGALVSVIGLVIMATLFYHELAYYLTTYT
V.viniferaERV-B              1 -----MGVK-QFIKSLHAFPRAEEHLLQKTQSGAVVSIIGLVIMATLFLHELRYYLTTYT
T.cacaoERV-B                 1 -----MGVK-QALKSLDAFPRAEEHLLQKTQSGALVSIVGLVIMAALFFHELTYYLTTYT
```

```
                                 ---------> BLOCK#2-------------------------->
C.subellipsoideaPDI-C     56 KEELIVDRSAHGDLLRINFNISFPSLSCEFATLDVSDALGTKRMN----LTKTIRKLPI-
C.reinhardtiiPDI-C        56 SSQLVVDRSPQNELLKLNFNISFPALSCEFATVDVSDSLGTKRMN----LTKTVRKVPIT
V.carteriPDI-C            56 TTQLIVDRSPQNELLKLNFNISFPALSCEFATVDVSDTLGTKRMN----LTKTVRKMPI-
K.flaccidumPDI-C          56 TTTVIIDRSRDGDVMRINFNFSFPALSCEFASVDVSDVLGTHRIN----LSKTVRKFPI-
B.rapaPDI-C1              55 TTAVVVDKSADGDFLRIHFNISFPALSCEFASVDVSDVLGTNRLN----ITKTIRKFPI-
B.rapaPDI-C2              55 TTAVVVDKSSDGDFLRIDFNISFPALSCEFASLDVNDVLGTNRLN----ITKTVRKFPI-
E.salsugineumPDI-C1       55 TTAVVVDNSADGDFLRITFNISFPALSCEFASVDVSDVLGTNRLN----ITKTVRKFPI-
A.thalianaPDI12           55 TTAVIVDKSSDGDFLRIDFNISFPALSCEFASVDVSDVLGTNRLN----ITKTVRKFPI-
C.rubellaPDI-C1           55 TTSVIVDKSTDGDFLRIDFNISFPALSCEFASVDVSDVLGTNRLN----ITKTIRKFPI-
B.rapaPDI-C5              55 QTSVVVDNSSDDDFLQIDFNVSFPALSCEFATFEVSDVLSTNRLN----LTKTIKKVPI-
A.thalianaPDI13           55 STSVIVDKSSDGDFLNIDFNISFPALSCEFASVDVSDVFGTHRLN----ISKTIRKVPI-
C.rubellaPDI-C3           55 STSVIVDKSSDGDILNIDFNISFPALSCEFASVDVSDVLGTHRLN----LTKTIRKVPI-
B.rapaPDI-C6              55 NTSVIVDNSSDGDFLRIDFNVSFPSLSCEFASVDVSNVLGTKRLN----LTKTIKKVPI-
E.salsugineumPDI-C3       55 NTSVIVDKSSDGDFLRIDFNVSFPSLSCEFASVDVSDVLGTSRLN----LTKTIRKVPI-
S.bicolorPDI-C            55 TTSVIVDRSSDGEFLRIDFNISFPALSCEFVSVDVSDVLGTNRLN----IRKTVRKYSI-
Z.maysPDI-C               55 TTSVIVDRSSDGEFLRIDFNMSFPALSCEFASVDVSDVLGTNRLN----ITKTVRKYSI-
B.distachyonPDI-C         55 TTSVIVDRSSDGEFLRIDFNMSFPALSCEFASVDVSDVLGTNRLN----ITKTVRKFSI-
O.sativaPDI-C             55 STSVIVDRSSDGEFLRIDFNLSFPALSCEFASVDVSDVLGTNRLN----ITKTVRKYSI-
B.rapaPDI-C4              55 TTSIIIDRSSDGDFLRMDFNISFPSVSCEFASVDVSDVLGTNRLN----VTKTIRKFSI-
A.thalianaPDI7            55 STSVIVDRSADGDFLRLDFNISFPSLSCEFASVDVSDVLGTNRLN----VTKTIRKFSI-
C.rubellaPDI-C2           55 STSVIVDRSSDGDFLRLDFNISFPSLSCEFASLDVSDVLGTNRLN----VTKTIRKFSI-
B.rapaPDI-C3              55 TTSIIVDRSSDGDFLRMDFNISFPSLSCEFASVDVSDVLGTNRLN----VTKTIRKFSI-
E.salsugineumPDI-C2       55 TTSIIVDRSSDGDFLRMDFNISFPSLSCEFASVDVSDVLGTNRLN----VTKTIRKFSI-
S.lycopersicumPDI-C       55 TTSVIVDKSSDAEFLRIDFNMSFPALSCEFASVDVSDVLGTNRLN----ITKTVRKHSI-
S.tuberosumPDI-C          55 TTSVVVDKSSDAEFLRIDFNMSFPALSCEFASVDVSDVLGTNRLN----ITKTVRKHSI-
M.truncatulaPDI-C1        55 STSVIIDKSSDGEFLRIDFNLSFHALSCEFASVDVSDVLGTNRMN----LTKTVRKFSI-
P.vulgarisPDI-C1          55 ATSVIVDKSSDGDYLRIDFNMSFPALSCEFAAVDVSDVLGTNRLN----LTKTIRKFSI-
G.maxPDI-C1               55 STQVIVDKSSDGDYLRIDFNISFPALSCEFAAVDVSDVLGTNRLN----LTKTVRKFSI-
G.maxPDI-C2               55 STQVIVDKSSDGDYLRIDFNISFPALSCEFAAVDVSDVLGTNRLN----LTKTVRKFSI-
M.truncatulaPDI-C2        55 STSVIVDKSSDGDFLRIDFNFSFPALSCEFASVDVSDVLGTNRLN----ITKTVRKFSI-
P.vulgarisPDI-C2          55 STSVIVDKSSDHDYLRIDFNISFPALSCEFASVDVSDVLGTNRLN----ITKTVRKFSI-
G.maxPDI-C3               55 STSVIVDKSSDGDYLRIDFNISFPALSCEFASVDVSDVLGTNRLN----ITKTVRKFSI-
G.maxPDI-C4               55 STSVIVDKSSDGDYLRIDFNISFPALSCEFASVDVSDVLGTNRLN----ITKTVRKFSI-
V.viniferaPDI-C           55 STSVIVDQSSDGDFLRIEFNISFPALSCEFASVDVSDVLGTNRLN----ITKTIRKYSI-
C.sativusPDI-C            55 STSVIVDNSTDGDFLRMDFNISFPALSCEFAAVDVNDVLGTNRLN----ITKTIRKFSI-
P.persicaPDI-C            55 STSVIVDKSSDGDFLRIEFNISFPALSCEFASIDVSDVLGTNRLN----ITKTIRKFSI-
T.cacaoPDI-C              55 STSVIVDKSSDGEFLRIDFNISFPALSCEFASVDVSDVLGTNRLN----ITKTIRKFSI-
P.trichocarpaPDI-C1       55 STTVIVDNSSDGEFLRIDFNISFPSLSCEFASVDVSDVLGTNRLN----ITKTIRKFSI-
P.trichocarpaPDI-C2       55 STSVIVDNSSDGEFLRIDFNLSFPSLSCEFASVDVSDVLGTNRLN----ITKTIRKFSI-
S.moellendorffiiPDI-C     55 TTNVVVDRSKDGEYLRIQFNMSFPALSCEFASVDVSDALGTNRYN----LTKTVRKYPI-
P.patensPDI-C             55 STSVVVDRSRDGEYLRIDFNLSFPALSCEFASVDVSDVLGTHRFN----LTKTVRKYPI-
C.reinhardtiiERV-A        58 AHELSVDVGR-GEKIKIHFDVTFPKVPCAWLSLDAMDISGELHLD----LDHDVYKQRL-
V.carteriERV-A            60 VHELSVDVGR-GEKIQIHFDLTFPKVPCSWLSLDAMDISGELHLD----LDHDVYKQRL-
C.subellipsoideaERV-A     56 TNELSVDTTR-GDQLSINFDMTFPALPCEWISLDLMDISGEMHLD----VDHDVYKRRL-
K.flaccidumERV-A          56 VNELSVDTSR-GEQLQINFDVTYPHLACSILSLDAMDVSGEQHLD----VVHNIFKRRL-
B.distachyonERV-A3        56 ETKLVVDTSR-GERLRVNFDITFPSIPCTLLSVDTRDISGEQHQD----IRHDIEKKRL-
O.sativaERV-A3            56 ETKLVVDTSR-GERLRVNFDVTFPSVPCTLLSVDTMDISGEQHHD----IRHDIEKRRL-
Z.maysERV-A3              56 ETKLVVDTSR-GERLRVNFDITFPSIPCTLLSVDTTDISGEQHHD----IRHDIEKRRL-
Z.maysERV-A2              56 ETKLVVDTSR-GERLRVNFDITFLSIPCTLLSVDTMDISGEQHQD----IRHDIEKIRL-
S.bicolorERV-A3           56 ETKLVVDTSR-GERLRVNFDITFPSIPCTLLSVDTMDISGEQHHD----IRHDIEKRRL-
P.patensERV-A1            57 ANDLVVDTER-GGTIQINLDVTFPALACSVVSLDAMDISGEAHLD----VKHNIFKKRL-
P.patensERV-A4            55 QNQLVVDTSR-GETLQINLDITFSALACSVVSLDAMDISGEQHLN----VRHNIFKKRL-
P.patensERV-A2            55 QNQLVVDTSR-GETLQINLDITFPALACSMVSLDAMDISGEQHLN----VRHNIFKKRL-
P.patensERV-A3            55 QNQLVVDTSR-GETLQINLDITFPALACSVVSLDAMDISGELHLD----VRHNIYKKRL-
S.bicolorERV-A2           55 ESKLTVDTSR-GERLHINFDVTFPALPCSLVAVDTMDVSGEQHYD----IRHDITKKRI-
B.distachyonERV-A2        55 ESKLTVDTSR-GERLHINFDVTFPALPCSLVAIDTMDVSGEQHYD----IRHDIFKKRI-
O.sativaERV-A2            55 DSKLTVDTSR-GERLHINFDVTFPALPCSLVAVDTMDVSGEQHYD----IRHDIIKKRI-
S.moellendorffiiERV-A     55 TSELLVDTSR-GETLQINFDITFPALACSVISLDAMDVSGEQHLD----VKHNIFKKRL-
C.sativusERV-A3           56 ETQLVVDTSR-GGELHINFDLSFPAIPCSILSLDAIDISGEQHLD----IRHNIIKKRI-
M.truncatulaERV-A2        56 ESKLLVDTSR-GETLNINFDVTFPAVRCSILSLDTMDISGERHHD----ILHNIMKQRI-
P.vulgarisERV-A2          56 ESTLLVDTSR-GDTLHINFDVTFPAVRCSILSLDAMDISGEQHLD----IRHNIVKKRI-
G.maxERV-A3               56 ESKLLVDTSR-GDTLHINFDVTFPAVRCSILSLDAMDISGEQHLD----IRHNIVKKRI-
G.maxERV-A4               56 ESKLLVDTSR-GDTLHINFDVTFPAVRCSILSLDAMDISGEQHLD----IRHNIVKKRI-
S.lycopersicumERV-A2      56 ETQLVVDTSR-GGKLHIHFDITFPAVPCSLLSLDARDISGEEHFD----IRHDIFKKRI-
S.tuberosumERV-A2         56 ETQLVVDTSR-GGKLHIHFDITFPAVPCSLLSLDARDISGEEHFD----IRHDIFKKRI-
P.persicaERV-A2           56 ESKLLVDASR-GETLHINFDVTFPAIRCSLLSLDTMDISGEQHFD----IRHDIVKKRI-
T.cacaoERV-A2             56 ETKLLVDTSR-GEALRINFDVTFPAIPCTLLNLDAMDISGEQHLD----IKHDIIKKRI-
P.trichocarpaERV-A3       56 ETKLLVDTTR-GQTLRINFDITFPAIRCSLLSVDAIDISGEQHHD----IRHDITKKRI-
P.trichocarpaERV-A4       56 ETKLLVDTSR-GQSLRINFDVTFPAIRCSLLSVDAIDISGEQHLD----IRHDISKKRI-
C.sativusERV-A1           56 ETKLIVDTSR-GEHLRINFDVTFPALPCSVLSLHAMDISGEQHLD----VKHDIVKKRI-
V.viniferaERV-A2          56 ETKLVVDTSR-GGTLRINFDVTFPAVPCSVLTLDAMDISGEQHHD----IKHDIVKKRI-
B.rapaERV-A4              52 ESHLRVDTTR-GEKLRINFDVTFPALQCSIISIDTMDISGERHLD----VRHDIFKRRL-
B.rapaERV-A3              52 ETQLRVDTSR-GEKLRINFDVTFPALQCSIISIDTMDISGERHLD----VRHDIIKRRL-
E.salsugineumERV-A2       56 ETQLRVDTSR-GEKLRINFDVTFPALQCSIISIDTMDISGERHLD----VRHDIIKRRL-
```

```
                              ---------> BLOCK#2-------------------------->
A.thalianaERV-A2          56 ETQLRVDTSR-GEKLRINFDVTFPALQCSIISLDSMDISGERHLD----VRHDIIKRRL-
C.rubellaERV-A2           56 ETQLRVDTSR-GEKLRINFDVTFPALQCSIISLDSMDISGERHLD----VRHDIIKRRL-
B.rapaERV-A2              56 ESQLRVDTSR-GEKLRINFDATFPALACSIISLDSMDISGEQHLD----VRHDMSKRRL-
E.salsugineumERV-A3       56 ESQLRVDTSR-GEKLRINFDVTFPALACSIISLDSMDISGEQHLD----VRHDIAKRRL-
B.distachyonERV-A1        56 ETTLRVDTSR-GEKLRINFDITFPALQCSIISIDVMDISGQEHLD----VKHDVFKQRI-
O.sativaERV-A1            56 ETTLRVDTSR-GETLRINFDVTFPALQCSIISLDAMDISGQEHLD----VKHDIFKQRI-
S.bicolorERV-A1           56 ETTLRVDTSR-GETLRINFDVTFPALQCSIISLDAMDISGQEHLD----VKHDVFKQRI-
Z.maysERV-A1              56 ETTLRVDTSR-GETLRINFDVTFPALQCSIISLDAMDISGQEHLD----VKHDVFKQRI-
S.lycopersicumERV-A1      56 ETKLVVDTSR-GETLRINFDITFPAIPCSILSVDAMDISGEQHLD----IRHDIIKKRI-
S.tuberosumERV-A1         56 ETKLIVDTSR-GETLRINFDITFPAIPCSILSVDAMDISGEQHLD----IRHDIIKKRI-
B.rapaERV-A1              56 ETKLVVDTSR-GETLRINFDMTFPALACSILSVDAMDISGELHLD----VKHDVVKRRL-
E.salsugineumERV-A1       56 ETKLVVDTSR-GETLRINFDITFPALACSILSVDAMDISGELHLD----VKHDIIKRRL-
A.thalianaERV-A1          56 ETKLIVDTSR-GETLRINFDITFPALACSILSVDAMDISGELHLD----VKHDIIKRRL-
C.rubellaERV-A1           56 ETKLIVDTSR-GETLRINFDITFPALACSILSVDAMDISGELHLD----VKHDIIKRRL-
V.viniferaERV-A1          56 ETKLVVDTSR-GETLRINFDVTFPALPCSILSLDAMDISGEQHLD----VRHDIIKKRI-
C.sativusERV-A2           56 ETKLVVDTSR-GETLRINFDVTFPALPCSLLSLDAMDISGEQHLD----VKHDIIKKRL-
M.truncatulaERV-A1        56 ETKLVVDTSR-GETLRINFDVTFPALACSIVSLDAMDISGEQHLD----VRHDIIKKRI-
P.persicaERV-A1           56 ETKLVVDTSR-GETLRINFDVTFSALPCSILSLDAMDISGEQHLD----VKHDIIKKRL-
P.vulgarisERV-A1          56 ETKLVVDTSR-GETLRINFDVTFPALPCSILSLDAMDISGEQHLD----VKHDIIKKRL-
G.maxERV-A1               56 ETKLVVDTSR-AETLRINFDVTFPALPCSILSLDAMDISGEQHLD----VKHDIIKKRL-
G.maxERV-A2               56 ETKLVVDTSR-AETLRINFDVTFPALPCSILSLDAMDISGEQRLD----VKHDIIKKRL-
T.cacaoERV-A1             56 ETKLVVDTSR-GETLRINFDVTFPALACSILSLDAMDISGEQHLD----VRHDIIKKRL-
P.trichocarpaERV-A1       56 ETKLVVDTSR-GETLRINFDVTFPALPCSILSLDAMDISGEQHLD----VKHDIIKKRL-
P.trichocarpaERV-A2       56 ETKLVVDTSR-GETLRINFDVTFPALPCSILSLDAMDISGEQHLD----VKHDIIKKRL-
C.reinhardtiiERV-B        55 VTVLSVDLAR-RHALTINLDITFPSVPCAVLSIDVLDISGTAENDASFAHHMRVHKMRL-
V.carteriERV-B            53 VTQMSVDLAR-RNALTINIDLTFPAIPCAVLSIDVLDIAGTAENDASYAHHMHIHKLRL-
C.subellipsoideaERV-B     54 IQTMSVDTSR-AHYIRMNFNFTYPSMPCQVLSLDATDMSGEKSGDSGHAANGEIHKVRL-
K.flaccidumERV-B          56 ENEMTVDVKG-REKLPIHINITFPSLPCSVLSLDALDMSGKHEVD----ISNNIWKARL-
P.patensERV-B             57 VHEMSVDVKR-GEKLPIHINMTFPALPCEVLSLDAIDMSGKHEVD----LDTNIWKLRI-
S.moellendorffiiERV-B     53 VHQMSVDTTR-GQNLPIHINITFPSLPCQILSVDAIDMSGKHEVD----LDTNIWKLRL-
S.bicolorERV-B            55 VHQMSVDLKR-GETLPIHINMSFPSLPCEVLSVDAIDMSGKHEVD----LHTNIWKLRL-
Z.maysERV-B               55 VHQMSVDLKR-GETLPIHINMSFPSLPCEVLSVDAIDMSGKHEVD----LHTNIWKLRL-
B.distachyonERV-B         55 MHQMSVDLKR-GETLPIHINMSFPSLPCEVLSVDAIDMSGKHEVD----LHTNIWKLRL-
O.sativaERV-B             55 VHQMSVDLKR-GETLPIHINMSFPSLPCEVLSVDAIDMSGKHEVD----LHTNIWKLRL-
B.rapaERV-B2              55 VHQMSVDLKR-GETLPIHVNMTFPSLPCDVLSMDAIDMSGKHEVD----LDTNIWKLRL-
B.rapaERV-B1              55 VHQMSVDLKR-GETLPIHVNMTFPSLPCDVLSMDAIDMSGKHEVD----LDTNIWKLRL-
E.salsugineumERV-B        55 VHQMSVDLKR-GETLPIHVNMTFPSLPCDVLSMDAIDMSGKHEVD----LDTNIWKLRL-
A.thalianaERV-B           55 VHQMSVDLKR-GETLPIHVNMTFPSLPCDVLSVDAIDMSGKHEVD----LDTNIWKLRL-
C.rubellaERV-B            55 VHQMSVDLKR-GETLPIHVNMTFPSLPCDVLSVDAIDMSGKHEVD----LDTNIWKLRL-
M.truncatulaERV-B         55 VHQMSVDLKR-GETLPIHINMTFPSLPCDVLSVDAIDMSGKHEVD----LDTNIWKLRL-
P.vulgarisERV-B           55 VHQMSVDLKR-GETLPIHINMTFPSLPCDVLSVDAIDMSGKHEVD----LDTNIWKLRL-
G.maxERV-B1               55 VHKMSVDLKR-GETLPIHINMTFPSLPCDVLSVDAIDMSGKHEVD----LDTNIWKLRL-
G.maxERV-B2               55 VHQMSVDLKR-GETLPIHINMTFPSLPCDVLSVDAIDMSGKHEVD----LDTNIWKLRL-
C.sativusERV-B            55 VHQMSVDLKR-GETLPIHINMTFPSLPCDVLSVDAIDMSGKHEVD----LDTNIWKLRL-
S.lycopersicumERV-B1      55 VHQMAVDLRR-GETLPIHINMTFPSLPCDVLSVDAIDMSGKHEVD----LDTNIWKLRL-
S.tuberosumERV-B1         55 VHQMAVDLRR-GETLPIHINMTFPSLPCDVLSVDAIDMSGKHEVD----LDTNIWKLRL-
S.lycopersicumERV-B2      55 VHQMSVDSRR-GENLPIHINMTFPSLPCDVLSVDAIDMSGKHEVD----LDTNIWKLRL-
S.tuberosumERV-B2         55 VHQMSVDSRR-GENLPIHINMTFPSLPCDVLSVDAIDMSGKHEVD----LDTNIWKLRL-
P.persicaERV-B            55 VHQMSVDLKR-GETLPIHINITFPSLPCDVLSVDAIDMSGKHEVD----LDTNIWKLRL-
P.trichocarpaERV-B1       55 VHQMSVDLTR-GETLPIHINITFPSLPCDVLSVDAIDMSGKHEVD----LDTSIWKLRL-
P.trichocarpaERV-B2       55 VHQMSVDLQR-GEILPIHVNITFPSLPCDVLSVDAIDMSGKHEVD----LDTNIWKLRL-
V.viniferaERV-B           55 VHQMSVDLKR-GETLPIHINMTFPSLPCDVLSVDAIDMSGKHEVD----LDTNIWKLRL-
T.cacaoERV-B              55 VHQMSVDLKR-GETLPIHINMTFPSLPCDVLSVDAIDMSGKHEVD----LDTNIWKLRL-
```

```
C.subellipsoideaPDI-C  111 ---DEDGQRAG-YYV-----------HDDLSNVDIK----YDE-----------------
C.reinhardtiiPDI-C     112 LDMERQGAAVEDTAH-----------KVGPKYDAEGHFDEEPD-----------------
V.carteriPDI-C         111 ---TTELERMG-SAV-----------EDSSHKPGPK----YDE-----------------
K.flaccidumPDI-C       111 ---DHAGRITG-PEFHHIAHTVREPAHDDPAPDPID----EGAAEMEAKERQLAEGGQPP
B.rapaPDI-C1           110 ---DPHLKTTG-EEF-----------HSGHGSHDIN----HGE-----------------
B.rapaPDI-C2           110 ---DPHLKATG-GEF-----------HSGLASHHIN----HGE-----------------
E.salsugineumPDI-C1    110 ---DPHLRTTG-AEF-----------HSGLALHDIN----HGE-----------------
A.thalianaPDI12        110 ---DPHLRSTG-AEF-----------HSGLALHNIN----HGE-----------------
C.rubellaPDI-C1        110 ---DPHLRSTG-AEF-----------HSGHTLHHIN----HGE-----------------
B.rapaPDI-C5           110 ---DPHLRDTG-EEY-----------HPTPDSDLIN----HGD-----------------
A.thalianaPDI13        110 ---DPHLRATA-EEF-----------HSTSDLHLIN----HGD-----------------
C.rubellaPDI-C3        110 ---DPHLRATG-TEF-----------HSTSGLHLIN----HGD-----------------
B.rapaPDI-C6           110 ---DPYLRATG-AEV-----------HSTSGLHLIN----HGD-----------------
E.salsugineumPDI-C3    110 ---DAHLRATG-AEF-----------HSTTGLDLIN----HGD-----------------
S.bicolorPDI-C         110 ---DRNFVPTG-SEF-----------HPGPIPTVNK----HGD-----------------
Z.maysPDI-C            110 ---DRNLVPTG-SEF-----------HPGPIPILNK----HGD-----------------
B.distachyonPDI-C      110 ---DRNLVPTG-SEF-----------HSGPIPTVNK----HGD-----------------
O.sativaPDI-C          110 ---DRNLVPTG-SEF-----------HPGPIPTVSK----HGD-----------------
B.rapaPDI-C4           110 ---DSNLRPTG-SEF-----------HSGEFLSRVN----HGD-----------------
A.thalianaPDI7         110 ---DSNMRPTG-SEF-----------HAGEVLSLIN----HGD-----------------
C.rubellaPDI-C2        110 ---DSNMRPTG-AEF-----------HAGEVLSLIN----HGD-----------------
B.rapaPDI-C3           110 ---DSNLRPTG-SEF-----------HSGEVLSHVN----HDE-----------------
E.salsugineumPDI-C2    110 ---DSNLRPTG-TEF-----------HSGEVLSLIN----HGD-----------------
S.lycopersicumPDI-C    110 ---DKNLRPTG-SEF-----------HSGSTATELK----HDA-----------------
S.tuberosumPDI-C       110 ---DKNLRPTG-SEF-----------HSGSTATELK----HDA-----------------
M.truncatulaPDI-C1     110 ---DSNLRPTG-SEF-----------YLRPSTNVIK----HDD-----------------
P.vulgarisPDI-C1       110 ---DSNLRSTG-NEF-----------HSEPTTTNIK----HDN-----------------
G.maxPDI-C1            110 ---DSNLRPTG-AEF-----------HSEPAANSIK----HDN-----------------
G.maxPDI-C2            110 ---DSNLRPTG-AEF-----------HSEPAANSIK----HDN-----------------
M.truncatulaPDI-C2     110 ---DSKLRPTG-SEF-----------HSGTIANAVK----HDD-----------------
P.vulgarisPDI-C2       110 ---DSHLRPTG-AEF-----------HSGAVSNAVK----HDD-----------------
G.maxPDI-C3            110 ---DSNLRPTG-AEF-----------HSGTVANAVK----HDD-----------------
G.maxPDI-C4            110 ---DSNLRPTG-AEF-----------HSGTVANAVK----HDD-----------------
V.viniferaPDI-C        110 ---DPDLRPTG-AEF-----------HSGPVGKVIK----HGD-----------------
C.sativusPDI-C         110 ---DSNLRSTG-SEF-----------HSGPLSNLIK----HGD-----------------
P.persicaPDI-C         110 ---GPDLKPTG-SEF-----------HSGPAFHDIK----HGD-----------------
T.cacaoPDI-C           110 ---DPHLRSTG-AEF-----------HAGPVPHFIK----HGD-----------------
P.trichocarpaPDI-C1    110 ---DHDLKPTG-SEF-----------HSGPVLHQIK----HGD-----------------
P.trichocarpaPDI-C2    110 ---DHDLKPTG-SEF-----------HSGPVLHHIN----HGD-----------------
S.moellendorffiiPDI-C  110 ---DPNLKIVG-PEF-----------HPGPIPNPTS----HGD-----------------
P.patensPDI-C          110 ---DPLLQRIG-QEF-----------HAGSVPNIKS----HGD---------------ED
C.reinhardtiiERV-A     112 ---NANGSPVKEVE------------KHNVDATKKK----PPS---------AVNATASA
V.carteriERV-A         114 ---SANGSPVKEVE------------KHNVEATKKV----VPV-----------------
C.subellipsoideaERV-A  110 ---DSNGVVIP-DSI-----------EKHQVGPELD----DTL-----------------
K.flaccidumERV-A       110 ---SPEGKPIDVGT------------KDEIGGPKVQTGEVKVK-----------------
B.distachyonERV-A3     110 ---NSHGNVIE-SR------------KEGIGGAKIE----RPL-----------------
O.sativaERV-A3         110 ---DAHGNVIE-AR------------KEGIGGAKIE----SPL-----------------
Z.maysERV-A3           110 ---NSHGNVIE-AR------------KEGIGGAKVE----RPL-----------------
Z.maysERV-A2           110 ---DAHGNVIE-AR------------KVSIGGAKIE----RPL-----------------
S.bicolorERV-A3        110 ---DSHGNVIE-AR------------KEGIGGAKIE----RPL-----------------
P.patensERV-A1         111 ---DVNGKVIEPAR------------QESINQPKLD----KPL-----------------
P.patensERV-A4         109 ---DVHGKAIDAPK------------PDAINAPKVQ----RPL-----------------
P.patensERV-A2         109 ---DVHGKVVNAPK------------PDAINAPKVQ----KPL-----------------
P.patensERV-A3         109 ---DVHGKAVDAPK------------PDAINAPKVQ----KPL-----------------
S.bicolorERV-A2        109 ---DHLGNVIE-SR------------KDRVGAPKIE----RPL-----------------
B.distachyonERV-A2     109 ---DHLGNVIE-SR------------KDGVGSPKIE----RPL-----------------
O.sativaERV-A2         109 ---DNLGNVIE-SR------------KDGVGAPKIE----RPL-----------------
S.moellendorffiiERV-A  109 ---DPSGKVVQ-PPV-----------QEDIGGPKID----KPL-----------------
C.sativusERV-A3        110 ---DHLGTVIE-AR------------PDGIGAPKIE----KPL-----------------
M.truncatulaERV-A2     110 ---DANGKVIE-AR------------KEGIGAPKIE----RPL-----------------
P.vulgarisERV-A2       110 ---DANGNVIE-ER------------KDGIGAPKIE----RPL-----------------
G.maxERV-A3            110 ---DANGNVIE-ER------------KDGIGAPKIE----RPL-----------------
G.maxERV-A4            110 ---DANGNVIE-ER------------KDGIGAPKIE----RPL-----------------
S.lycopersicumERV-A2   110 ---DSHGAVIE-VR------------QDGIGAPKIE----KPL-----------------
S.tuberosumERV-A2      110 ---DSHGAVIE-VR------------QDGIGAPKIE----KPL-----------------
P.persicaERV-A2        110 ---DAHGNVIE-AK------------KDGIGAPKID----NPL-----------------
T.cacaoERV-A2          110 ---NAHGDVIE-SR------------QDGIGAPKID----KPL-----------------
P.trichocarpaERV-A3    110 ---NAHGDVIE-VR------------QDGIGAPKID----KPL-----------------
P.trichocarpaERV-A4    110 ---NAHGDVIE-VR------------QEGIGAPKID----RPL-----------------
C.sativusERV-A1        110 ---DYQGNVID-SR------------PDGIGSTEIE----RPL-----------------
V.viniferaERV-A2       110 ---DAHGNVVA-VR------------QDGIGGPQIE----KPL-----------------
B.rapaERV-A4           106 ---DSHGNVIE-AK------------QGGIGHTKIE----KPL-----------------
B.rapaERV-A3           106 ---DSHGNVIE-SK------------QDGIGHTKIE----KPL-----------------
E.salsugineumERV-A2    110 ---DSHGNVIE-AK------------QDGIGHTKIE----KPL-----------------
```

```
A.thalianaERV-A2         110 ---DSSGNVIE-AK------------QDGIGHTKIE----KPL-----------------
C.rubellaERV-A2          110 ---DSTGNVIE-AK------------QDGIGHTKIE----KPL-----------------
B.rapaERV-A2             110 ---DAYGNVIE-TR------------QDGIGHTKIE----NPL-----------------
E.salsugineumERV-A3      110 ---DSSGNVIE-TR------------QDGIGHTKIE----NPL-----------------
B.distachyonERV-A1       110 ---DANGNVIA-TK------------QDAVGGMKVE----KPL-----------------
O.sativaERV-A1           110 ---DVHGNVIA-TK------------QDAVGGMKVE----QPL-----------------
S.bicolorERV-A1          110 ---DAHGNVIA-TR------------QDAVGGMKME----APL-----------------
Z.maysERV-A1             110 ---DAHGNVIA-TR------------QDVVGGMKME----APL-----------------
S.lycopersicumERV-A1     110 ---DVLGNVIE-TR------------KEGIGSPTID----RPL-----------------
S.tuberosumERV-A1        110 ---DVLGNVIE-TR------------KEGIGSPTID----RPL-----------------
B.rapaERV-A1             110 ---DSHGNIIE-SR------------QDGIGAAKIE----KPL-----------------
E.salsugineumERV-A1      110 ---DSHGNTIE-AR------------QDGIGATKIE----NPL-----------------
A.thalianaERV-A1         110 ---DSNGNTIE-AR------------QDGIGATKIE----NPL-----------------
C.rubellaERV-A1          110 ---DSNGNTIE-AR------------QDGIGATKIE----KPL-----------------
V.viniferaERV-A1         110 ---DAHGSVIE-AR------------QDGIGSPKIE----KPL-----------------
C.sativusERV-A2          110 ---DSHGNAIE-AR------------PDGIGAPKIE----KPL-----------------
M.truncatulaERV-A1       110 ---DSHGNVIE-TR------------QDGIGSPNIE----KPL-----------------
P.persicaERV-A1          110 ---DSHGNVIE-SR------------PDGIGAPKIE----KPL-----------------
P.vulgarisERV-A1         110 ---DSHGNVIE-TR------------QEGIGAPKIE----KPL-----------------
G.maxERV-A1              110 ---DSHGNVIE-TR------------QEGIGAPKIE----KPL-----------------
G.maxERV-A2              110 ---DSRGNVIE-TR------------QEGIGAPKIE----KPL-----------------
T.cacaoERV-A1            110 ---DAHGNVIE-SR------------QDGIGAPKIE----KPL-----------------
P.trichocarpaERV-A1      110 ---DFHGNVIE-AR------------QDGIGAPKIE----KPL-----------------
P.trichocarpaERV-A2      110 ---DSHGNVIE-SR------------QDGIGAPKIE----KPL-----------------
C.reinhardtiiERV-B       113 ---DKAGNQIGKAEY-----------HTPQSQQIMD------------------------
V.carteriERV-B           111 ---DGAGKPIGKAEY-----------HTPQSQQIMD----TGA-----------------
C.subellipsoideaERV-B    112 ---NEAGEKIG-----------------------------LGE-----------------
K.flaccidumERV-B         110 ---DSQGRPGI-WQQ-----------VDKL-----D----HADD----------------
P.patensERV-B            111 ---HRDGYVLG-SEF-----------VNDLVEGEHR----KEE-----------------
S.moellendorffiiERV-B    107 ---HKDGHILG-SEY-----------LSDLVEKEHA----HDN-----------------
S.bicolorERV-B           109 ---DKYGHIIG-TEY-----------LSDLVEKGHG------------------------
Z.maysERV-B              109 ---DKYGHIIG-TEY-----------LSDLVEKGHG------------------------
B.distachyonERV-B        109 ---DKYGTIIG-TEY-----------LSDLVEKE------HGA-----------------
O.sativaERV-B            109 ---DKYGHIIG-TEY-----------LNDLVEKE------HGT-----------------
B.rapaERV-B2             109 ---NSHGHIIG-TEY-----------ISDLVEKEHD----HSS-----------------
B.rapaERV-B1             109 ---NSHGHIIG-TEY-----------ISDLVEKEHD----HSS-----------------
E.salsugineumERV-B       109 ---NSHGHIIG-TEY-----------ISDLVEKEHD----HST-----------------
A.thalianaERV-B          109 ---NSHGHIIG-TEY-----------ISDLVEKGHE----HGH-----------------
C.rubellaERV-B           109 ---NSHGHIIG-TEY-----------ISDLVEKGHE----HGH-----------------
M.truncatulaERV-B        109 ---NSYGQIIG-TEY-----------ISDLVEKGHEHDHDHGT-----------------
P.vulgarisERV-B          109 ---NSDGHIIG-TEY-----------ISDLVEKG------HTH-----------------
G.maxERV-B1              109 ---NSYGHIIG-TEY-----------ISDLVEKE------HTN-----------------
G.maxERV-B2              109 ---NSYGHIIG-TEY-----------ISDLVEKEHT----HHK-----------------
C.sativusERV-B           109 ---NSHGQIIG-TEY-----------LSDLVEKE------HVD-----------------
S.lycopersicumERV-B1     109 ---NSDGHITG-TEY-----------LSDLVEKE--------------------------
S.tuberosumERV-B1        109 ---NSDGHITG-TEY-----------LSDLVEKEHK----HDD-----------------
S.lycopersicumERV-B2     109 ---NSDGHITG-TEY-----------LSDLVEKE------REA-----------------
S.tuberosumERV-B2        109 ---NSDGHITG-TEY-----------LSDLVEKE------HEV-----------------
P.persicaERV-B           109 ---NSYGHIIG-TEY-----------LSDLVEREHS------------------------
P.trichocarpaERV-B1      109 ---NSYGHITG-TEY-----------LSDLVEKE------HEA-----------------
P.trichocarpaERV-B2      109 ---NSHGHITG-TEY-----------LSDLVEKE------HEA-----------------
V.viniferaERV-B          109 ---NRDGFIIG-TEY-----------LSDLVEKE------HAD-----------------
T.cacaoERV-B             109 ---NSLGQIVG-TEY-----------LSDLVEKE------HAA-----------------
```

```
C.subellipsoideaPDI-C  135 -----PSVSQDFALPLSKDSFKATLEA-YSIVVVNFYAPWCPWCQRLEPTWEAVTQEVHT
C.reinhardtiiPDI-C     144 ---------IDITVPLSHENFEATLAR-YPIAVINFYAPWCHWCQRLEPTWEAATKEVHD
V.carteriPDI-C         135 EGRFDDEPDIDITVPLSHVNFEATLAR-YPIVVVNFFAPWCHWCQRLEPTWEAATKEVHD
K.flaccidumPDI-C       163 EGDPPPIKDPGGALPLSEATFDTITKESFPIVMVNFFAPWCPWSQRLEPVWEASAKAMLK
B.rapaPDI-C1           134 ---ETKEEIPDGSVPLVSSSFDSFSKH-FPLLIVNFNAPWCYWSNRLKPSWEKASSIIYH
B.rapaPDI-C2           134 ---EIKQEFPDGAIQLTNGGFQSLSHH-FPLLIVNFNAPWCYWSNRLKPSWEKAATIIKQ
E.salsugineumPDI-C1    134 ---ESKEEFPDGAIPLTSGSFESYSHH-FPILIVNFYAPWCYWSNRLKPSWEKAANIIKQ
A.thalianaPDI12        134 ---ETKEEFPDGAIPLTSASFEALSHH-FPILVVNFNAPWCYWSNRLKPSWEKAANIIKQ
C.rubellaPDI-C1        134 ---ETKEEFPDGAIPLTSGSFESLSHH-FPILVVNFNAPWCYWSNRLKPSWEKAANIIKQ
B.rapaPDI-C5           134 ---EHHDDNTYAAIPLSGGTFDKISHK-FPILVVNFYAPWCYWSSRLRPSWEKAAEITRQ
A.thalianaPDI13        134 --EDHGDNSTYADIPLTGAAFEKFTHH-FQILVVNFYAPWCYWSNRLKPSWVKASQITRE
C.rubellaPDI-C3        134 ---EDHEENTYIDIPLTGATFEKYKHH-FQILVVNFYAPWCYWSNRLKPSWEKASQITRE
B.rapaPDI-C6           134 ---EDHGNNTYAAIPLTGATFDKFSHH-FQILVVNFYAPWCYWSNRLKPSWEKAAEITRQ
E.salsugineumPDI-C3    134 --EDHHENNSYAAIPLTGATFDKFSHH-FPILVVNFYAPWCYWSNRLKPSWEKAAEITRQ
S.bicolorPDI-C         134 ---DVEEDHVDGAFSLSSRNFDSFSHQ-YPVLVVNFYAPWCYWSNRLKPSWEKTAKIIRE
Z.maysPDI-C            134 ---DVEEDHVDGAFSLSSRNFDSFSHQ-YPVLVVNFYAPWCYWSNRLKPSWEKTAKIMRE
B.distachyonPDI-C      134 ---DVEEYHADGSVALSSRNFDSYSHQ-YPILVVNFYAPWCYWSNRLKPSWEKTAKIIKE
O.sativaPDI-C          134 ---DVEENHDDGSVPLSSRNFDSYSHQ-YPVLVVNFYAPWCYWSNRLKPSWEKTAKIMRE
B.rapaPDI-C4           134 ---ESAEELVEGSVSLGARNFDTFLHQ-YPISVVNFYAPWCYWCNLLKPSWEKAANQIKE
A.thalianaPDI7         134 ---ETGEEIVEDSVPLTGRNFDTFTHQ-FPILVVNFYAPWCYWCNLLKPSWEKAAKQIKE
C.rubellaPDI-C2        134 ---ETGEEVAEDSVSLTGRNFETFTHQ-FPILVVNFYAPWCYWCNLLKPSWEKAAKQIKE
B.rapaPDI-C3           134 ----AGEEVVEDSVSLTSRNFDTLLHQ-FPISVVNFYAPWCYWCNLLKPSWEKAAKQIKE
E.salsugineumPDI-C2    134 ---EAGEELVEDSVSLTGRNFDTLLHQ-FPISVVNFYAPWCYWCNLLKPSWEKAAKQIKE
S.lycopersicumPDI-C    134 ---EDDEEYGEGSVSLNGHSFDRVTHH-FPILVVNFFAPWCYWSNRLKPSWEKAANIIRE
S.tuberosumPDI-C       134 ---EDDEEYGEGSVSLNGHSFDRVTHH-YPILVVNFFAPWCYWSTRLKPSWEKAANIIRE
M.truncatulaPDI-C1     134 ---KVDDESLEGAFVFTSNNFDKYSHQ-FPITAVNFYAPWCYWSQRLRPSWEKTAKIIRE
P.vulgarisPDI-C1       134 ---EQHEESIGGALELTTDNFDKYAHQ-FPITVINFYAPWCYWSQRLRPSWEKASKIIKE
G.maxPDI-C1            134 ---EVNEESVEGSVVLKTQNFDKYAHQ-FPITVVNFYAPWCYWSQRLKPSWEKAAKIIKE
G.maxPDI-C2            134 ---EVNEESVEGSVVLTTQNFDKYAHQ-FLITVVNFYAPWCYWSQRLKPSWEKTAKIIKE
M.truncatulaPDI-C2     134 ---EVDTDFVEGSLPLTSQHFDKYVQL-FPITVVNFYAPWCSWCQRLKPSWEKAAKIMRE
P.vulgarisPDI-C2       134 ---EVDGESVEGSFSFTTHNFDKYAHQ-FPITVVNFYAPWCSWCQRLKPSWEKTAKIMKE
G.maxPDI-C3            134 ---EVDEESVEGSFSLTTHNFDKYVHQ-FPVTIVNFYAPWCSWCQRLKPSWEKTAKIMKE
G.maxPDI-C4            134 ---EVDEESVEGSFSLTTHNFDKYVHQ-FPITAVNFYAPWCSWCQRLKPSWEKTAKIMKE
V.viniferaPDI-C        134 ---ETDEEYSEGSASLTAQNFYKYSHQ-HAILVVNFFAPWCYWSTRLKPSWEKAAKIIRE
C.sativusPDI-C         134 ---EVDEEANEGSAVLNTRNFDRYANQ-HPILVVNFYAPWCYWSNRLKPSWEKAAKTIRE
P.persicaPDI-C         134 ----GDEYGGDGSVSITARTFEKFTHQ-HPILVVNFYAPWCYWSNRLKPSWEKAAKIIRE
T.cacaoPDI-C           134 ---EVDEETVEGSVPLNGVSFDKLSHL-YPILVVNFYAPWCYWSNRLKPSWEKAAKIIKE
P.trichocarpaPDI-C1    134 ---EVDEEGGEGSVSLKAHNFDQYSHQ-YPILVVNFFAPWCYWSNRLKPSWEKAAKIIRE
P.trichocarpaPDI-C2    134 ---EVHEEGSEGSVSLKAHNFDQYTHQ-YPILVVNFYAPWCYWSNRLKPSWEKAAKIIRE
S.moellendorffiiPDI-C  134 -----DDHEGEGAHVLTSSTFDEYARR-YSVLVVNFYAPWCIWSARLKPSWDKAAGIIAE
P.patensPDI-C          136 VGEDMFEHLGEGAVELNKNTFDVYAQQ-FSVLVVNFYAPWCPWSNKLKASWEKAAKIIAD
C.reinhardtiiERV-A     144 AAGGAPAGGAAGAAGAEGGAAGGAGGG-ENATALANGCGSCYGAEDKQGDCCNTCDEVRA
V.carteriERV-A         138 ----------------NGTENSTATPV----------CGSCYGAEDRQGDCCNTCDEVRA
C.subellipsoideaERV-A  134 ---------------LHKAN--------------ETECGSCYGA-APDEECCNNCEEVRA
K.flaccidumERV-A       138 --------------ESEGG---------------PAYCGSCWGAEENAEDCCNTCEEVRE
B.distachyonERV-A3     133 --------------QKHGGRLD---KG-------EQYCGTCYGAEESDEQCCNSCDEVRE
O.sativaERV-A3         133 --------------QKHGGRLS---KG-------EEYCGTCYGAEESDEQCCNSCEEVRE
Z.maysERV-A3           133 --------------QKHGGRLD---KG-------EQYCGTCYGAEESDEQCCNSCEEVRE
Z.maysERV-A2           133 --------------QKHGGRLD---KG-------EQYCGTCYGAEESDEQCCNSCEEVRE
S.bicolorERV-A3        133 --------------QKHGGRLD---KG-------EQYCGTCYGAEESDEQCCNSCEEVRE
P.patensERV-A1         135 --------------QKHGGRLE---HN-------ETYCGSCFGAETEEDHCCNNCEEVRE
P.patensERV-A4         133 --------------QKHGGRLE---HN-------ETYCGSCFGAASSDDECCNSCEEVRE
P.patensERV-A2         133 --------------QKHGGRLE---HN-------ETYCGSCFGAESSDDECCNNCEEVRE
P.patensERV-A3         133 --------------QKHGGRLE---DH-------ETYCGSCFGAESSDDQCCNSCEEVRE
S.bicolorERV-A2        132 --------------QKHGGRLD---HN-------EVYCGSCYGAEETDDQCCNSCEEVRD
B.distachyonERV-A2     132 --------------QNHGGRLD---HN-------EAYCGSCYGSEESDDQCCNSCEEVRD
O.sativaERV-A2         132 --------------QKHGGRLD---HN-------EVYCGSCYGSEESDDQCCNSCEDVRD
S.moellendorffiiERV-A  133 --------------QKHGGRLE---HN-------ETYCGSCFGAEQSDDECCNSCEEVRE
C.sativusERV-A3        133 --------------QKHGGRLE---HN-------ETYCGSCFGAEASDDDCCNSCEEVRE
M.truncatulaERV-A2     133 --------------QKHGGRLE---HD-------EKYCGSCFGAEESDDHCCNNCEEVRE
P.vulgarisERV-A2       133 --------------QKHGGRLG---HD-------EKYCGSCFGAEETDEQCCNSCEEVRE
G.maxERV-A3            133 --------------QKHGGRLG---HD-------EKYCGSCFGAEESDEHCCNSCEEVRE
G.maxERV-A4            133 --------------QKHGGRLG---HD-------EKYCGSCFGAEESDEHCCNSCEEVRE
S.lycopersicumERV-A2   133 --------------QRHGGRLE---HN-------ETYCGSCFGAETADDECCNSCEEVRE
S.tuberosumERV-A2      133 --------------QRHGGRLE---HN-------ETYCGSCFGAETADDECCNSCEEVRE
P.persicaERV-A2        133 --------------QRHGGRLE---HN-------EKYCGSCFGAEMSDDDCCNSCDEVRD
T.cacaoERV-A2          133 --------------QRHGGRLE---HN-------ETYCGSCFGAEQSDDDCCNSCEEVRE
P.trichocarpaERV-A3    133 --------------QKHGGRLE---HN-------EEYCGSCFGAEMSDDHCCNSCDEVRE
P.trichocarpaERV-A4    133 --------------QSHGGRLG---HN-------EEYCGSCFGGEMSHDDCCNTCEEVRE
C.sativusERV-A1        133 --------------QKHGGRLK---QN-------ETYCGSCYGASG--EDCCNSCQDVRE
V.viniferaERV-A2       133 --------------QRHGGRLE---HN-------EKYCGSCYGAEVTDDDCCNSCDEVRE
B.rapaERV-A4           129 --------------QKHGGRLE---DN-------EKYCGSCFGAEETDDACCNSCEEVRE
B.rapaERV-A3           129 --------------QKHGGRLE---HN-------ETYCGSCFGAETTDDTCCNSCEEVRE
E.salsugineumERV-A2    133 --------------QKHGGRLE---HN-------ETYCGSCFGAEMTDEACCNSCEEVRE
```

```
A.thalianaERV-A2        133 --------------QKHGGRLE---HN-------ETYCGSCFGAEASDDACCNSCEEVRE
C.rubellaERV-A2         133 --------------QKHGGRLE---HN-------ETYCGSCFGAELSDDACCNSCEEVRE
B.rapaERV-A2            133 --------------QKHGGRLE---HN-------ETYCGSCYGAEASDDECCNSCEEVRE
E.salsugineumERV-A3     133 --------------QRHGGRLE---HN-------ETYCGSCYGAEATDEECCNSCEEVGE
B.distachyonERV-A1      133 --------------QMHGGRLE---HN-------ETYCGSCYGAEEPGEQCCNSCEDVRE
O.sativaERV-A1          133 --------------QRHGGRLE---HN-------ETYCGSCYGAEESDEQCCNSCEDVRE
S.bicolorERV-A1         133 --------------QHHGGRLE---HN-------ETYCGSCYGAQESDGQCCNSCEDVRE
Z.maysERV-A1            133 --------------QHHGGRLE---HN-------ETYCGSCYGAQESDDQCCNTCEDVRE
S.lycopersicumERV-A1    133 --------------QRHGGRLE---HN-------ETYCGSCYGAEGSDDHCCNTCEDVRE
S.tuberosumERV-A1       133 --------------QRHGGRLE---HN-------ETYCGSCYGSEGSDDHCCNTCEDVRE
B.rapaERV-A1            133 --------------QKHGGRLE---HN-------ETYCGSCYGAEAEEHDCCNSCEDVRE
E.salsugineumERV-A1     133 --------------QKHGGRLE---HK-------ETYCGSCYGAEAEEHDCCNSCEDVRE
A.thalianaERV-A1        133 --------------QKHGGRLG---HN-------ETYCGSCYGAEAEEHDCCNSCEDVRE
C.rubellaERV-A1         133 --------------QKHGGRLE---HN-------ETYCGSCYGAEAEEHDCCNSCEDVRE
V.viniferaERV-A1        133 --------------QKHGGRLE---HN-------ETYCGSCYGAEASDDDCCNNCEEVRE
C.sativusERV-A2         133 --------------QRHGGRLE---HN-------ETYCGSCFGAESADDDCCNSCEEVRE
M.truncatulaERV-A1      133 --------------QRHGGRLE---HN-------ETYCGSCYGAEASDEECCNSCEEVRE
P.persicaERV-A1         133 --------------QRHGGRLE---HN-------ETYCGSCYGAEAADEDCCNSCEDVRE
P.vulgarisERV-A1        133 --------------QRHGGRLE---HN-------ETYCGSCFGAEASDDDCCNSCEDVRE
G.maxERV-A1             133 --------------QRHGGRLE---HN-------ETYCGSCYGAEESDDDCCNSCEDVRE
G.maxERV-A2             133 --------------QRHGGRLE---HN-------ETYCGSCYGSEVSDDDCCNSCEDVRE
T.cacaoERV-A1           133 --------------QRHGGRLE---HN-------ETYCGSCYGAEASDDDCCNSCEDVRE
P.trichocarpaERV-A1     133 --------------QRHGGRLE---HN-------ETYCGSCYGAEASDEDCCNSCEDVRE
P.trichocarpaERV-A2     133 --------------QRHGGRLE---HN-------ETYC---------DEDCCNSCEEVRE
C.reinhardtiiERV-B      135 ----------------TGGEQL---------------------VSVNIQEAMQHLVDMED
V.carteriERV-B          136 -------------EQLVSVNIQ---EA------------------------MQHLVDMEE
C.subellipsoideaERV-B   123 ------------------------------------YIPPRRWGFMMGKP-RQEVMEVNQ
K.flaccidumERV-B        130 --------------TAHNEAHA--EDT------TEHDFALANARRAAQADANKQVEDIKA
P.patensERV-B           135 --------------PKADKKDE---HK---------------DGDHRKKDPQKVINEVKK
S.moellendorffiiERV-B   131 -------------------------------------LTGIFHSHEELRSAVKVVNEINK
S.bicolorERV-B          130 --------------AHHDHDHGQEHHD-----------EQKKPEQTFNEEAEKMIKSVKQ
Z.maysERV-B             130 --------------AHHDHDHD---HD--------HHDEQKKHEQTFNEEAEKMIKSVKQ
B.distachyonERV-B       131 --------------HHHDNGHE---HH----------DEEKKPEHTFNEDADKMVKSVRQ
O.sativaERV-B           131 --------------HNHDHDHE---HE----------DEQKKQEHTFNEDAEKMVKSVKQ
B.rapaERV-B2            133 --------------HKHDEHKN---------------ETDALNLLGFDEAAETMIKKVKQ
B.rapaERV-B1            133 --------------HKHDGKEE---HK---------NETEAVNTLGFDEAAETMIKKVKQ
E.salsugineumERV-B      133 --------------HKHEHKNE----------------TEALNVLGFDQAAETMIKKVKQ
A.thalianaERV-B         133 ------------SPHKHDGKEE---HK-------NETETEALNILGFDQAAETMIKKVKQ
C.rubellaERV-B          133 ------------SSHKHDGKEE---HK-------NETEIETLNSLGFDQAAETMIKKVKQ
M.truncatulaERV-B       137 --------------HKHDDSKD---HH--------EHSEQKVHLQTFDEATENTIKKVKE
P.vulgarisERV-B         131 --------------HKHDDHKD---HD--------EHSEQKIHLQNLDESTENIIKKVKE
G.maxERV-B1             131 --------------QEHDDNKDHDHHH--------EHSEQKIHLQNLDESTENIIKKVKE
G.maxERV-B2             133 ----------------HDDNKN---HE---------HSEQKIHLQNLDESTENIIKKVKE
C.sativusERV-B          131 --------------HKHDHDHDKEKDH--------------PHIHGFDQAAENLVKKVKQ
S.lycopersicumERV-B1    128 --------------HKHDDHKD---HH--------DDSDNKTHMQGFDQEAENLIKKVKQ
S.tuberosumERV-B1       133 ----------------SDNKTH---------------------MQGFDQDAENLIKKVKQ
S.lycopersicumERV-B2    131 --------------HKHDVHKE---HH---------EDSDKIHLQGIDEESQNMIKKVKQ
S.tuberosumERV-B2       131 --------------HKHDAHKE---HH---------EDSDKIHLQGIDEESQNMIKKVKQ
P.persicaERV-B          130 --------------HKHDDSKD---HH-------EDKDQEIHLQGAFDQAAEDLIKRVKH
P.trichocarpaERV-B1     131 --------------HNHDHNKD---HH--------EDSHAKQHTHGFDDAAETMVKKVKQ
P.trichocarpaERV-B2     131 --------------HNHDHDKD---HH--------KDSHEEQHTHGFDDAAETMIKKVKQ
V.viniferaERV-B         131 --------------HKHDHNKD---HH--------GDSDQKLHAHSFDQDAENMVKKVKQ
T.cacaoERV-B            131 --------------HKHDDEKE---HH--------DDSDKKLHALGFDQEAENMIKKVKQ
```

```
C.subellipsoideaPDI-C   189 KY-PDADGRIRFAKVDCTTEVDLCREHQITGFPSIRVFRSGHDEVNVHGVKEHESYRGDR
C.reinhardtiiPDI-C      194 KY-PEWDGRVRFAKVDCTAEVDLCRQHFIQGFPSIRVFRKGHDDIYIGGMHEHEAYMGDR
V.carteriPDI-C          194 KY-PEWDGRIRFAKVDCTQEMELCRTHFIQGFPSIRVFRKGHDDIVIGGMHEHESYMGDR
K.flaccidumPDI-C        223 KYDVDVNPQILLAKVDCTVEVALCRKHHVQGFPSIRIFRSGQDVRAAQGHHDHESYYGDR
B.rapaPDI-C1            190 KYNPETDGRVLLGSVDCTEEAELCKRNHIQGYPSIRIFRKGSDLKEDHGHHEHESYYGDR
B.rapaPDI-C2            190 RYNPDTDGRVLLGSVDCTEEPALCRRNHIQGYPSIRIFRKGNDLKEDHGHHEHESYYGDR
E.salsugineumPDI-C1     190 RYDPETDGRVLLGNVDCTEEAALCRRNHIQGYPSIRIFRKGSDLREDHGHHEHESYYGDR
A.thalianaPDI12         190 RYDPEADGRVLLGNVDCTEEPALCKRNHIQGYPSIRIFRKGSDLREDHGHHEHESYYGDR
C.rubellaPDI-C1         190 KYDPETDGRVLLGNVDCTEEPALCKRNHIQGYPSIRIFRKGSDLREDHGHHEHESYYGDR
B.rapaPDI-C5            190 KYGPENDGRVLLGSVDCTEEPTLCTKYHIQGYPSIRIFHNGSDLRGDDGHQEHDSYHGNR
A.thalianaPDI13         191 RYNPGTDDRVLLGSVDCTEEPTLCKSNHIQGYPSIRIFRRGSGLREDHGNHEHESYYGDR
C.rubellaPDI-C3         190 RYDPETDDRVLLGSVDCTKESTLCKSNHIQGYPSIRIFRKGSDLRDDHGHHDHESYYGDR
B.rapaPDI-C6            190 RYNPETDGRVLLGSVDCTEETTLCKRNHIQGYPSIRIFRKGSDLKEDHGHHEHESYHGDR
E.salsugineumPDI-C3     191 KYDPEDDGRVLLGSVDCTEEPTLCKRNHIQGYPSIRIFRKGSDLREDHGHHEHESYYGDR
S.bicolorPDI-C          190 RYDPEMDGRILLGKVDCTEEVDLCRRHHIQGYPSIRVFRKGSDIKENQGHHDHESYYGER
Z.maysPDI-C             190 RYDPEMDGRILLGKVDCTEEVELCRRNHIQGYPSIRVFRKGSDIKENQGHHDHESYYGER
B.distachyonPDI-C       190 RYDPEMDGRILLAKVDCTEEGELCKRHHIQGYPSIRIFRKGSDMKENQGHHDHESYYGER
O.sativaPDI-C           190 RYDPEMDGRIILAKVDCTEEIDLCRRHHIQGYPSIRIFRKGSDLKENQGHHDHESYYGDR
B.rapaPDI-C4            190 RYDPEMDGRVILAKVDCTQEADLCRRNHIQGYPSIRIFRKGSDLRDDNAHHDHESYYGDR
A.thalianaPDI7          190 RYDPEMDGRVILAKVDCTQEGDLCRRNHIQGYPSIRIFRKGSDLKDDNAHHDHESYYGDR
C.rubellaPDI-C2         190 RYDPEMDGRVIVAKVDCTQEGDLCRRNHIQGYPSIRIFRKGSDLKDDNAHHEHESYYGDR
B.rapaPDI-C3            189 RYDPEMDGRVILAKVDCTQEADLCRKNHIQGYPSIRIFRQGSDLK-DNAHHDHESYYGDR
E.salsugineumPDI-C2     190 RYDPEMDGRVILAKVDCTVESDLCRRNHIQGYPSIRIFRKGSDLRDDNAHHDHESYYGDR
S.lycopersicumPDI-C     190 RYDRESDGRILVAKVDCTEEVDLCRRNHIQGYPSIRIFRKGTDVRDDHGHHDHESYYGDR
S.tuberosumPDI-C        190 RYDRESDGRILVAKVDCTEEVDLCRRNHIQGYPSIRIFRKGSDVRDDHGHHDHESYYGDR
M.truncatulaPDI-C1      190 RYDPEMDGRILLGKVDCTKEADLCRRHHIQGYPSIRIFRKGSDVRSDHGHHEHESYYGDR
P.vulgarisPDI-C1        190 RYDPETDGRIVMGRVDCTQNGELCRSHHVQGYPSIRIFRKGSDVRTDHGHHDHESYYGDR
G.maxPDI-C1             190 RYDPEMDGRIILGRVDCTEDGDLCRSHHIQGYPSIRIFRKGSDVRSNHGHHDHESYYGDR
G.maxPDI-C2             190 RYDPEMDGRIILGRVDCTEDGDLCRSHHIQGYPSIRIFRKGSDVRSDHGHHDHESYYGDR
M.truncatulaPDI-C2      190 RYDPEMDGRILLAKVDCTQEGDLCRRHHIQGYPSIRIFRKGSDVRSDHGHHEHESYYGDR
P.vulgarisPDI-C2        190 RYDPEMDGRIIMGKVDCTLEGDLCRRNHIQGYPSIRIFRKGSDLRSEHGHHEHESYYGDR
G.maxPDI-C3             190 RYDPEMDGRIILAKVDCTQEGDLCRRNHIQGYPSIRIFRKGSDLRSEHGHHEHESYYGDR
G.maxPDI-C4             190 RYDPEMDGRIILAKVDCTQEGDLCRRNHIQGYPSIRIFRKGTDLRSEHGHHEHESYYGDR
V.viniferaPDI-C         190 RYDPELDGRIVMAKVDCTEEGELCRRHHIQGYPSIRIFRKGSDVRDDHGHHDHESYYGDR
C.sativusPDI-C          190 RYDPELDGRILMAKVDCTEEGDLCRKHHIQGYPSIRIFRKGSDVRDDHGHHDHESYYGDR
P.persicaPDI-C          189 RYDPEIDGRILLAKVDCTEEGDLCKRNHIQGYPSIRIFRKGSDVRDDHGHHDHESYYGDR
T.cacaoPDI-C            190 RYDPEMDGRILLAKVDCTEEVDLCRRHHIQGYPSIRIFRKGSDLREDHGHHDHESYYGDR
P.trichocarpaPDI-C1     190 RYDPEMDGRILLAKVDCTEEGDLCRRNHIQGYPSIRIFRKGSNLREDHGRHDHESYYGDR
P.trichocarpaPDI-C2     190 RYDPEIDGRILLAKVDCTEEGDLCRRNHIQGYPSIRIFRKGSDLRDDHGHHDHESYYGDR
S.moellendorffiiPDI-C   188 KYHPD-TGRILLGKVDCTDNNDLCRKHHIQGFPSIRIFHKGHDLKDEHGHHEHDSYYGER
P.patensPDI-C           195 KYNPEMDGRILLAKVDCTVNVELCRSHHIQGYPSIRIFRKGHDVRDEHGRHDHESYYGER
C.reinhardtiiERV-A      203 AYRRKGWALSNVDHIEQCAHDLYTEA-----------------IKEQAGE----------
V.carteriERV-A          172 AYRRKGWALANVDHIEQCAHDLYTES-----------------IKEQTGE----------
C.subellipsoideaERV-A   164 AYRRKGWGFTDPQQISQCAKEGFVEK-----------------LRAQEGE----------
K.flaccidumERV-A        169 AYRRKGWAFSNAELIEQCSREGFVEK-----------------LKEQEGE----------
B.distachyonERV-A3      169 AYKKKGWALTNPDLIDQCAREDFVER-----------------VKTQHGE----------
O.sativaERV-A3          169 AYKKKGWALTNPDLIDQCTREDFVER-----------------VKTQQGE----------
Z.maysERV-A3            169 AYKKKGWALTNPDLIDQCAREDFIDR-----------------VKTQQDE----------
Z.maysERV-A2            169 AYKKKGWALTNPDLIDQCAREDFVER-----------------VKTQQDE----------
S.bicolorERV-A3         169 AYKKKGWALTNPDLIDQCAREDFVER-----------------VKTQQDE----------
P.patensERV-A1          171 AYRKKGWALNNPDLIDQCKREGFLQK-----------------IKDEDGE----------
P.patensERV-A4          169 AYRKKGWALINIDIIDQCHREGFIER-----------------VKEEAGE----------
P.patensERV-A2          169 AYRKKGWALTNADLIDQCHREGFIER-----------------VKEEAGE----------
P.patensERV-A3          169 AYRKKGWALTNTDLIDQCHREGFIER-----------------IKEEAGE----------
S.bicolorERV-A2         168 VYRKKGWAINNVELIDQCKREGYVQR-----------------LKDETGE----------
B.distachyonERV-A2      168 AYRKKGWALTNVESIDQCKREGFVQR-----------------LKDEQGE----------
O.sativaERV-A2          168 AYRKKGWALTNIEEIDQCKREGFVQR-----------------LKDEQGE----------
S.moellendorffiiERV-A   169 AYRKRGWAIHNADLIDQCKREGWLTK-----------------IKEEEGE----------
C.sativusERV-A3         169 AYRKKGWAITNQDLIDQCQREDFIQK-----------------VKDEEGE----------
M.truncatulaERV-A2      169 AYRKKGWALTNIDLIDQCQREGFVQK-----------------VKDEEGE----------
P.vulgarisERV-A2        169 AYRKKGWALSNMDLIDQCQREGYVQR-----------------VKDEEGE----------
G.maxERV-A3             169 AYRKKGWAMTNMDLIDQCQREGYVQR-----------------VKDEEGE----------
G.maxERV-A4             169 AYRKKGWAMTNMDLIDQCQREGYVQR-----------------VKDEEGE----------
S.lycopersicumERV-A2    169 AYRKRGWGMTNPDLIDQCKREGFVQK-----------------IKDEEGE----------
S.tuberosumERV-A2       169 AYRKRGWGMTNPDLIDQCKREGFVQK-----------------IKDEEGE----------
P.persicaERV-A2         169 AYRKKGWALTNADMIDQCKREGFIQK-----------------IKDEDGE----------
T.cacaoERV-A2           169 AYRRKGWAMTNVDLIDQCKREGFIQR-----------------VKDEDGE----------
P.trichocarpaERV-A3     169 AYRKKGWALTNMDLIDQCIREGFVQM-----------------IKDEEGE----------
P.trichocarpaERV-A4     169 AYRRKGWAMTNMDLIDQCKREGFIQM-----------------IKDEEGE----------
C.sativusERV-A1         167 AYHRKGWALSHPDLIDQCKREGFFQR-----------------VKNEEGE----------
V.viniferaERV-A2        169 AYRKKGWGMTNPDLIDQCKREGFVQK-----------------VKEEEGE----------
B.rapaERV-A4            165 AYRKKGWGLSDPESIDQCQREGYVQK-----------------VKDEEGE----------
B.rapaERV-A3            165 AYRKKGWALSDPESIDQCKREGYVQK-----------------VKDEEGE----------
E.salsugineumERV-A2     169 AYRKKGWALSDPESIDQCKREGFVQK-----------------VKDEEGE----------
```

```
A.thalianaERV-A2        169 AYRKKGWALSDPESIDQCKREGFVQK-----------------VKDEEGE----------
C.rubellaERV-A2         169 AYRKKGWALSDPESIDQCKREGFVQK-----------------VKDEEGE----------
B.rapaERV-A2            169 AYRKKGWAMTDPDIIDQCKREGFVQR-----------------VKEEEGE----------
E.salsugineumERV-A3     169 AYRKKGWAMSDPEIIDQCKREGFVQR-----------------VKDEEGE----------
B.distachyonERV-A1      169 AYRKKGWGVSNPDSIDQCKREGFLQT-----------------IKDEEGE----------
O.sativaERV-A1          169 AYRKKGWGVSNPDLIDQCKREGFLQS-----------------IKDEEGE----------
S.bicolorERV-A1         169 AYRKKGWGVSNPDLLDQCKREGFLQS-----------------IKDEEGE----------
Z.maysERV-A1            169 AYRKKGWGVSNPDLLDQCKREGFLQS-----------------IKDEEGE----------
S.lycopersicumERV-A1    169 AYRKKGWALTNPDEIDQCKREGFLEK-----------------IKEEEGE----------
S.tuberosumERV-A1       169 AYRKKGWALTNPDEIDQCKREGFLEK-----------------IKEEEGE----------
B.rapaERV-A1            169 AYRKKGWGVTNPDLIDQCKREGFLQR-----------------VKDEEGE----------
E.salsugineumERV-A1     169 AYRKKGWGVTNPDLIDQCKREGFLQR-----------------VKDEEGE----------
A.thalianaERV-A1        169 AYRKKGWGVTNPDLIDQCKREGFLQR-----------------VKDEEGE----------
C.rubellaERV-A1         169 AYRKKGWGVTNPDLIDQCKREGFLQR-----------------VKDEEGE----------
V.viniferaERV-A1        169 AYRKKGWAMSNPDLIDQCKREGFLQR-----------------IKDEEGE----------
C.sativusERV-A2         169 AYRKKGWALSNPDLIDQCKREGFLQR-----------------IKDEDGE----------
M.truncatulaERV-A1      169 AYRKKGWALSSPDSIDQCKREGFLER-----------------IKEEEGE----------
P.persicaERV-A1         169 AYRKKGWAISNPDVIDQCKREGFLQR-----------------IKDEEGE----------
P.vulgarisERV-A1        169 AYRKKGWALSNPDLIDQCKREGFLQR-----------------IKDEDGE----------
G.maxERV-A1             169 AYRKKGWALSNPDLIDQCKREGFLQR-----------------IKDEEGE----------
G.maxERV-A2             169 AYRKKGWALSNPDLIDQCKREGFLQR-----------------IKDEEGE----------
T.cacaoERV-A1           169 AYRKKGWALSNPDLVDQCKREGFLQK-----------------IKDEEGE----------
P.trichocarpaERV-A1     169 AYRKKGWAVTNPDLMDQCKREGFLQK-----------------IKDEEGE----------
P.trichocarpaERV-A2     160 AYQKKGWAVTNPDLMDQCKREGFLQR-----------------IKDEEGE----------
C.reinhardtiiERV-B      158 EA--------------------------------------------DHHE----------
V.carteriERV-B          156 EA--------------------------------------------EHHE----------
C.subellipsoideaERV-B   146 AM--------------------------------------------DAHE----------
K.flaccidumERV-B        168 AL--------------------------------------------ANQE----------
P.patensERV-B           163 AI--------------------------------------------DDGE----------
S.moellendorffiiERV-B   154 AL--------------------------------------------QDGE----------
S.bicolorERV-B          165 AL--------------------------------------------GNGE----------
Z.maysERV-B             165 AL--------------------------------------------GNGE----------
B.distachyonERV-B       164 AL--------------------------------------------ENGE----------
O.sativaERV-B           164 AM--------------------------------------------ENGE----------
B.rapaERV-B2            164 AL--------------------------------------------ADGE----------
B.rapaERV-B1            167 AL--------------------------------------------ADGE----------
E.salsugineumERV-B      163 AL--------------------------------------------ADGE----------
A.thalianaERV-B         171 AL--------------------------------------------ADGE----------
C.rubellaERV-B          171 AL--------------------------------------------ADGE----------
M.truncatulaERV-B       172 AL--------------------------------------------KNGE----------
P.vulgarisERV-B         166 AI--------------------------------------------KNQE----------
G.maxERV-B1             169 AL--------------------------------------------KNGE----------
G.maxERV-B2             165 AL--------------------------------------------KNGE----------
C.sativusERV-B          163 AL--------------------------------------------EEAQ----------
S.lycopersicumERV-B1    163 AL--------------------------------------------AHGE----------
S.tuberosumERV-B1       156 AL--------------------------------------------AHGE----------
S.lycopersicumERV-B2    165 AL--------------------------------------------ADGE----------
S.tuberosumERV-B2       165 AL--------------------------------------------ADGE----------
P.persicaERV-B          166 AI--------------------------------------------ANGE----------
P.trichocarpaERV-B1     166 AL--------------------------------------------ANGE----------
P.trichocarpaERV-B2     166 AL--------------------------------------------ANGE----------
V.viniferaERV-B         166 AL--------------------------------------------ANGE----------
T.cacaoERV-B            166 AL--------------------------------------------ANGE----------
```

```
                                                                                        BLOCK#3
C.subellipsoideaPDI-C  248 TQASLLAFADNLAPSAGQPHHYIRGV--------------------TRMAKTSGCALSGF
C.reinhardtiiPDI-C     253 TKDALVAFADSLVPSAGQPHRKLAGL--------------------SAAPKTPGCNLAGF
V.carteriPDI-C         253 TKEALVAFADSLVPSAGQPHRKHAAL--------------------SAAPKTPGCNLAGF
K.flaccidumPDI-C       283 TEAAITQFADDLATGLQQRAHALPGATAQQVVPPWMGKDGRALRVVAKAPQSSGCQIEGF
B.rapaPDI-C1           250 DTDSIVKMVDELVAPIHPETHKLDLD-GIS---------NKTLKHLKKAPVTGGCRVEGY
B.rapaPDI-C2           250 DTESIVKMVDELVAPIHPETHKLALDWGIS---------NDTAKLLKKAPVTGGCRVEGY
E.salsugineumPDI-C1    250 DTDSIVKMVDGLVAPIHPETHKLALD-GIS---------NDTAKNLKKAPVTGGCRVEGY
A.thalianaPDI12        250 DTDSIVKMVEGLVAPIHPETHKVALD-GKS---------NDTVKHLKKGPVTGGCRVEGY
C.rubellaPDI-C1        250 DTKSIVKMVDELVAPIHPETHKVALD-GKS---------NDTVKHLKKAPVTGGCRVEGY
B.rapaPDI-C5           250 DTESLVKMVEELLRPIKK------FD-GTT---------NHAASRIRKAPVSGGCRIEGY
A.thalianaPDI13        251 DTDSLVKMVEELLKPIKKEDHKLALD-GKS---------DNAASTFKKAPVSGGCRIEGY
C.rubellaPDI-C3        250 DTDSLVKMVEELLKPIKKEDHKLALD-GKS---------ENAASTVKKAPVSGGCRIEGY
B.rapaPDI-C6           250 DTESILKMVEELLKPIKKEDHKLALD-GKT---------DNVVSGIKKAPVSGGCRIVGY
E.salsugineumPDI-C3    251 DTESLLKMVEELLKPIKKEDHKLPLD-GIS---------VNTASSIKKAPVSGGCRIEGY
S.bicolorPDI-C         250 DTESLVAAMETYVANIPKEAHVLALE-DKS--------NKTADPAKRPAPMTSGCRIEGF
Z.maysPDI-C            250 DTESLVAAMETYVANIPKEAH--ALE-DKS--------NKTVDPAKRPAPMASGCRIEGF
B.distachyonPDI-C      250 DTDSLVAAMETYVGNLPKEAHMLALD-DKS--------NKTVDPAKRPAPMTSGCRVEGF
O.sativaPDI-C          250 DTESLVAAMETYVANIPKDAHVLALE-DKS--------NKTVDPAKRPAPLTSGCRIEGF
B.rapaPDI-C4           250 DTESLVKMVIGLVEPIHLEPHKLALE-DKS--------GNASKTLKK-APSTGGCRIEGY
A.thalianaPDI7         250 DTESLVKMVVSLVEPIHLEPHNLALE-DKS--------DNSSRTLKK-APSTGGCRVEGY
C.rubellaPDI-C2        250 DTESLVKMVVSLVEPIHLEPHNLALE-DKS--------GNSSKTLKK-APSTGGCRIEGY
B.rapaPDI-C3           248 DTESLVKMVIGLVEPIHLEPHKLALE-DKS--------DNASKTLKK-APSTGGCRIEGY
E.salsugineumPDI-C2    250 DTESLVKMVIGLVEPIHLEPQRLALE-DKS--------DNASKILKK-APSTGGCRIEGY
S.lycopersicumPDI-C    250 DTDSLVKMMEDLVAPIKLDSQTITSD-NSS--------TKLETGLKRPAPVTGGCRIEGF
S.tuberosumPDI-C       250 DTDSLVKMMEDLVAPIKLDSQMISSD-NSS--------TKLETGLKRPAPVTGGCRIEGF
M.truncatulaPDI-C1     250 DTDSLVKTMENILASFPSEYYKLALE-DKL---------NVTEDSKRPAPSSGGCRIEGY
P.vulgarisPDI-C1       250 DTDSLVKTMDNLVASLPTESQKSHSG-DKS---------NLASHIKRPAPSSGGCRIEGY
G.maxPDI-C1            250 DTDSLVKTMENLVASLPSESQKLPLE-DKS---------DVAKNTERPAPSTGGCRIDGY
G.maxPDI-C2            250 DTDSLVKTMENLVASLPSESQKLPLE-DKS---------NVATNTKRPAPSTGGCRIDGY
M.truncatulaPDI-C2     250 DTESLVKTMETLVASLPTGSQHLALE-------------DKSNGTKRPAPSTGGCRVEGY
P.vulgarisPDI-C2       250 DTESLVEFMENLVTSLPTASQKPALE-DKS---------NATDNAKRPGPSAGGCRIEGY
G.maxPDI-C3            250 DTESLVKFMEDLVTSLPTESQKLALE-DKS---------NAADNAKRPAPSAGGCRVEGY
G.maxPDI-C4            250 DTESLVKFMEDLVTSLPTESQKLALE-DKS---------NASDNAKRPAPSAGGCRVEGY
V.viniferaPDI-C        250 DTDTLVTTMETLVAPIPLESQRLALE-NKS--------DSTADHIKRPAPRTGGCRIEGF
C.sativusPDI-C         250 DTDSLVKTMEDLIAPLPAGSQKLALE-DKS--------NNETGNVKRPAPSAGGCRIEGY
P.persicaPDI-C         249 DTDSLVKTMETLVAPIPVEAQKLALE-GKS--------DNGTDNAKRPAPLTGGCRIEGY
T.cacaoPDI-C           250 DTESLVKTMEELVAPIPLESQKLALE-------------DKSNITKRPAPKTGGCRIEGY
P.trichocarpaPDI-C1    250 DTESLVKTMEALVAPIAMESQRQALE-HKP--------ENATQHVKRPAPSAGGCRIEGY
P.trichocarpaPDI-C2    250 DTDSLVKTMEGLVAPIAMESQRHALE-HKP--------ENATEHVKRPAPSAGGCRIEGY
S.moellendorffiiPDI-C  247 DTDSLVKAMEALVPKETT----LALE-DKT-----------NGTVKRPAPRAGGCRIEGF
P.patensPDI-C          255 DTESLVAFMVELVPPATVDGKFQLEDKSSI---------TVNATIKRPAPKAGGCRVEGF
C.reinhardtiiERV-A     236 ------------------------------------------------------GCHMWGM
V.carteriERV-A         205 ------------------------------------------------------GCHMWGM
C.subellipsoideaERV-A  197 ------------------------------------------------------GCHMWGS
K.flaccidumERV-A       202 ------------------------------------------------------GCNVYGY
B.distachyonERV-A3     202 ------------------------------------------------------GCSVHGF
O.sativaERV-A3         202 ------------------------------------------------------GCNVHGF
Z.maysERV-A3           202 ------------------------------------------------------GCNVLGF
Z.maysERV-A2           202 ------------------------------------------------------GCNVHGF
S.bicolorERV-A3        202 ------------------------------------------------------GCNVHGF
P.patensERV-A1         204 ------------------------------------------------------GCNVYGT
P.patensERV-A4         202 ------------------------------------------------------GCNIYGK
P.patensERV-A2         202 ------------------------------------------------------GCNIYGK
P.patensERV-A3         202 ------------------------------------------------------GCNIYGK
S.bicolorERV-A2        201 ------------------------------------------------------GCTIHGF
B.distachyonERV-A2     201 ------------------------------------------------------GCNIHGF
O.sativaERV-A2         201 ------------------------------------------------------GCSIHGF
S.moellendorffiiERV-A  202 ------------------------------------------------------GCNIYGS
C.sativusERV-A3        202 ------------------------------------------------------GCNIEGS
M.truncatulaERV-A2     202 ------------------------------------------------------GCNIHGS
P.vulgarisERV-A2       202 ------------------------------------------------------GCNIQGS
G.maxERV-A3            202 ------------------------------------------------------GCNLQGS
G.maxERV-A4            202 ------------------------------------------------------GCNLQGS
S.lycopersicumERV-A2   202 ------------------------------------------------------GCNIHGS
S.tuberosumERV-A2      202 ------------------------------------------------------GCNIHGS
P.persicaERV-A2        202 ------------------------------------------------------GCNIEGS
T.cacaoERV-A2          202 ------------------------------------------------------GCNIHGS
P.trichocarpaERV-A3    202 ------------------------------------------------------GCNINGS
P.trichocarpaERV-A4    202 ------------------------------------------------------GCNINGS
C.sativusERV-A1        200 ------------------------------------------------------GCNIYGF
V.viniferaERV-A2       202 ------------------------------------------------------GCNVYGF
B.rapaERV-A4           198 ------------------------------------------------------GCNVHGF
B.rapaERV-A3           198 ------------------------------------------------------GCNLHGF
E.salsugineumERV-A2    202 ------------------------------------------------------GCNVHGF
```

BLOCK#3

```
A.thalianaERV-A2         202 -----------------------------------------------------GCNVHGF
C.rubellaERV-A2          202 -----------------------------------------------------GCNIHGF
B.rapaERV-A2             202 -----------------------------------------------------GCNIFGF
E.salsugineumERV-A3      202 -----------------------------------------------------GCNIYGF
B.distachyonERV-A1       202 -----------------------------------------------------GCNIYGF
O.sativaERV-A1           202 -----------------------------------------------------GCNIYGF
S.bicolorERV-A1          202 -----------------------------------------------------GCNIYGF
Z.maysERV-A1             202 -----------------------------------------------------GCNIYGF
S.lycopersicumERV-A1     202 -----------------------------------------------------GCNMYGF
S.tuberosumERV-A1        202 -----------------------------------------------------GCNMYGF
B.rapaERV-A1             202 -----------------------------------------------------GCNIYGF
E.salsugineumERV-A1      202 -----------------------------------------------------GCNIYGF
A.thalianaERV-A1         202 -----------------------------------------------------GCNIYGF
C.rubellaERV-A1          202 -----------------------------------------------------GCNIYGF
V.viniferaERV-A1         202 -----------------------------------------------------GCNIYGF
C.sativusERV-A2          202 -----------------------------------------------------GCNIYGF
M.truncatulaERV-A1       202 -----------------------------------------------------GCNVYGF
P.persicaERV-A1          202 -----------------------------------------------------GCNIYGF
P.vulgarisERV-A1         202 -----------------------------------------------------GCNVYGF
G.maxERV-A1              202 -----------------------------------------------------GCNVYGF
G.maxERV-A2              202 -----------------------------------------------------GCNVYGF
T.cacaoERV-A1            202 -----------------------------------------------------GCNIYGF
P.trichocarpaERV-A1      202 -----------------------------------------------------GCNIYGF
P.trichocarpaERV-A2      193 -----------------------------------------------------GCNIYGF
C.reinhardtiiERV-B       164 -----------------------------------------------------GCHVYGT
V.carteriERV-B           162 -----------------------------------------------------GCHVYGT
C.subellipsoideaERV-B    152 -----------------------------------------------------GCNIFGW
K.flaccidumERV-B         174 -----------------------------------------------------GCRVYGH
P.patensERV-B            169 -----------------------------------------------------GCQIFGV
S.moellendorffiiERV-B    160 -----------------------------------------------------GCRVFGV
S.bicolorERV-B           171 -----------------------------------------------------GCRVYGM
Z.maysERV-B              171 -----------------------------------------------------GCRVYGM
B.distachyonERV-B        170 -----------------------------------------------------GCRVYGM
O.sativaERV-B            170 -----------------------------------------------------GCRVYGV
B.rapaERV-B2             170 -----------------------------------------------------GCRVYGV
B.rapaERV-B1             173 -----------------------------------------------------GCRVYGV
E.salsugineumERV-B       169 -----------------------------------------------------GCRVYGV
A.thalianaERV-B          177 -----------------------------------------------------GCRVYGV
C.rubellaERV-B           177 -----------------------------------------------------GCRVYGV
M.truncatulaERV-B        178 -----------------------------------------------------GCRVYGV
P.vulgarisERV-B          172 -----------------------------------------------------GCRVYGV
G.maxERV-B1              175 -----------------------------------------------------GCRVYGV
G.maxERV-B2              171 -----------------------------------------------------GCRVYGV
C.sativusERV-B           169 -----------------------------------------------------GCRVYGV
S.lycopersicumERV-B1     169 -----------------------------------------------------GCRVYGV
S.tuberosumERV-B1        162 -----------------------------------------------------GCRVYGV
S.lycopersicumERV-B2     171 -----------------------------------------------------GCRVYGV
S.tuberosumERV-B2        171 -----------------------------------------------------GCRVYGI
P.persicaERV-B           172 -----------------------------------------------------GCQVFGV
P.trichocarpaERV-B1      172 -----------------------------------------------------GCRVYGV
P.trichocarpaERV-B2      172 -----------------------------------------------------GCRVYGV
V.viniferaERV-B          172 -----------------------------------------------------GCRVYGV
T.cacaoERV-B             172 -----------------------------------------------------GCRVYGV
```

```
                                   -------->                                        BLOCK#4---->
C.subellipsoideaPDI-C     288 VLVKKVPGALHFLAKSPGHS-----------FDYQAM----NMSHVVNYLYFGNKPSPRR
C.reinhardtiiPDI-C        293 VMVKKVPGTVHFVARSEGHS-----------FDHTWM----NMTHMIHSFHVGTRPSPRK
V.carteriPDI-C            293 VMVKKVPGTLTVVARSEGHS-----------FDHTWM----NMTHLVHTFHVGTRPSPRK
K.flaccidumPDI-C          343 VLVKKVPGNLQVAAVSAGHS-----------FDPATM----NMSHSVNSFSFGRKPTLKQ
B.rapaPDI-C1              300 VRVKKVPGNLIISAHSGAHS-----------FDSSKM----NMSHVVSHLSFGRMFSPRL
B.rapaPDI-C2              301 VRVKKVPGNLVISAHSGAHS-----------FDSSQM----NMSHVVTHLSFGRMIDTRL
E.salsugineumPDI-C1       300 VRVKKVPGNLVISAHSGAHS-----------FDSTQM----NMSHVVTHLSFGRMISPRL
A.thalianaPDI12           300 VRVKKVPGNLVISAHSGAHS-----------FDSSQM----NMSHVVSHFSFGRMISPRL
C.rubellaPDI-C1           300 VRVKKVPGNLVISAHSGAHS-----------FDSSQM----NMSHVVSHLSFGRMISPRL
B.rapaPDI-C5              294 VRAKKVPGELVISAVSGSHS-----------FDASRM----NMTHFVNHLSFGRLISDRL
A.thalianaPDI13           301 VRAKKVPGELVISAHSGAHS-----------FDASQM----NMSHIVTHLTFGTMVSERL
C.rubellaPDI-C3           300 VRAKKVPGELVISAHSGAHS-----------FDASQM----NMSHIVTHLTFGTMVSERL
B.rapaPDI-C6              300 VRAKKVPGEIIISAHSGAHS-----------FDASQM----NMSHYVSHLTFGKMISERL
E.salsugineumPDI-C3       301 VRAKKIPGEVVISAHSGAHS-----------FDASQM----NMSHFVTHLSFGKLISERL
S.bicolorPDI-C            301 VRVKRVPGSVIVAARSGSHS-----------FDPSQI----NVSHYVTQFSFGKRLSHRM
Z.maysPDI-C               299 VRVKRVPGSVVISARSGSHS-----------FDPSQI----NVSHYVTQFSFGKRLSPRM
B.distachyonPDI-C         301 VRVKKVPGSVIISARSGSHS-----------FDPSQI----NVSHYVTQFSFGNRLSPNM
O.sativaPDI-C             301 VRVKKVPGSVVISARSGSHS-----------FDPSQI----NVSHYVTQFSFGKRLSAKM
B.rapaPDI-C4              300 MRVKKVPGNLMVSARSESHS-----------FDTSQM----NMSHVVNHLSFGKRILPEA
A.thalianaPDI7            300 MRVKKVPGNLMVSARSGSHS-----------FDSSQM----NMSHVVNHLSFGRRIMPQK
C.rubellaPDI-C2           300 MRVKKVPGNLMVSARSGSHS-----------FDSSQM----NMSHVVNHLSFGRRILPQK
B.rapaPDI-C3              298 IRVKKVPGNLMVSARSGSHS-----------FDSTQM----NMSHVVNHLSFGRKILPQT
E.salsugineumPDI-C2       300 MRVKKVPGNLMVSARSGSHS-----------FDSSQM----NMSHVVNHLSFGRRILPQT
S.lycopersicumPDI-C       301 VRVKKVPGNLVISARSAAHS-----------FDASQM----NMSHVISSFSFGKTITPKV
S.tuberosumPDI-C          301 VRVKKVPGNLVISARSAAHS-----------FDASQM----NMSHVISSFSFGKTITPKV
M.truncatulaPDI-C1        300 VRVKKVPGNLIISARSDAHS-----------FDASQM----NMSHAVHHLSFGKKLSPKL
P.vulgarisPDI-C1          300 VRVKKVPGNLIISARSDAHS-----------FDASQM----NMSHVVNHLSFGRKVSPRV
G.maxPDI-C1               300 VRVKKVPGNLIFSARSNAHS-----------FDASQM----NMSHVINHLSFGRKVSPRV
G.maxPDI-C2               300 VRVKKVPGNLIISARSNAHS-----------FDASQM----NMSHVINHLSFGRKVSLRV
M.truncatulaPDI-C2        297 VRVKKVPGSLVVSARSDAHS-----------FDASQM----NMSHVINHLSFGKKVTPRA
P.vulgarisPDI-C2          300 VRVKKVPGNLIISARSDAHS-----------FDASQM----NMSHVVNNLSFGKKVTPRA
G.maxPDI-C3               300 VRVKKVPGNLIISARSDAHS-----------FDASQM----NMSHVINNLSFGKKVTPRA
G.maxPDI-C4               300 VRVKKVPGNLIISARSDAHS-----------FDASQM----NMSHFINNLSFGKKVTPRA
V.viniferaPDI-C           301 VRVKKVPGNLVISARSGSHS-----------FDPSQM----NMSHVISHLSFGRKIAPRV
C.sativusPDI-C            301 VRVKKVPGSLVIAARSESHS-----------FDASQM----NMSHIISHLSFGRKISPKA
P.persicaPDI-C            300 VRVKKVPGNLVISAHSGAHS-----------FDASQM----NMSHVISHFSFGRMIAPKV
T.cacaoPDI-C              297 VRVKKVPGNLIISARSAAHS-----------FDASQM----NMSHVISHLSFGKTISPRV
P.trichocarpaPDI-C1       301 VRVKKVPGNLMISALSGAHS-----------FDSKQM----NLSHVISHFSFGMKVLPRV
P.trichocarpaPDI-C2       301 VRVKKVPGNLVISARSGAHS-----------FDSAQM----NLSHVISHFSFGMKVLPRV
S.moellendorffiiPDI-C     291 IRAKKVPGNIIISAHSGSHS-----------FDASAM----NMTHYVSQFTFGRELNFWM
P.patensPDI-C             306 VRVKKVPGELMISAHSGSHS-----------FDATSM----NMTHYVGFFSFGRKTSWRS
C.reinhardtiiERV-A        243 LEVNKVAGNFHFAPGRSYQQGSMHVHD-IAPFGDAVI----DFRHVIHKLSFG-------
V.carteriERV-A            212 LEVNKVAGNFHFAPGRSYQQGSMHVHD-IAPFGDAVI----DFRHTVNKLSFG-------
C.subellipsoideaERV-A     204 LAVNKVAGNFHFAPGKSFQQGPMHVHD-LVPFQGVTF----DLSHRIDKLSFG-------
K.flaccidumERV-A          209 LEVNKVAGNFHFAPGKSFQQAHMHVHD-LMPFASQKF----NVSHIINKLSFG-------
B.distachyonERV-A3        209 LDVSKVAGNFHFAPGRGFYESNVDVPE--LSSLEGGF----NITHKINKLSFG-------
O.sativaERV-A3            209 LDVSKVAGNLHFAPGKGFYESNINVPE--LSALEHGF----NITHKINKLSFG-------
Z.maysERV-A3              209 LDVSKVAGNFHFAPGKGFYESNIDVPE--LSLLEGGF----NISHKINKLSFG-------
Z.maysERV-A2              209 LDVSKVAGNFHFAPGKGFYESNIDVPE--LSLLEGGF----NITHKINKLSFG-------
S.bicolorERV-A3           209 LDVSKVAGNFHFAPGKGFYESNIDVPE--LSVLEGGF----NITHKINKLSFG-------
P.patensERV-A1            211 LEANKVAGNFHFAPGKSFQQANMHVHD-LMAFGKDSF----NVSHKINEISFG-------
P.patensERV-A4            209 LEVNKVAGNFHIAPGKLFQQSAMHLLD-LLGIRSDSF----NVSHIVNELSFG-------
P.patensERV-A2            209 LEVNKVAGNFHFAPGKSFQQSAMHLLD-LMGFITDSF----NVSHTINELSFG-------
P.patensERV-A3            209 LEVNKVAGNFQIAPGKSFQQSAMHLLD-LMGFVTDSF----NVSHTINELSFG-------
S.bicolorERV-A2           208 VNVNKVAGNFHFAPGKSLDQSFNFLQD-LLNIQPETY----NISHKINKLSFG-------
B.distachyonERV-A2        208 VDVNKVAGNFHFAPGKHLDQSFNFLQD-MLNFQPENY----NISHKINKLSFG-------
O.sativaERV-A2            208 VNVNKVAGNFHFAPGKSLDQSFNFLQD-LLNFQQENY----NISHKINKLSFG-------
S.moellendorffiiERV-A     209 LEVNKVAGNFHFAPGKSFSQQHVHVHD-VQSLHKEKF----NVSHYINELSFG-------
C.sativusERV-A3           209 LEVNKVAGSFHFVPGKSFYQSSFNFLG-LLALQTSDY----NVSHRINRLAFG-------
M.truncatulaERV-A2        209 LEVNKVAGNFHFATGQSFLQSAIFLTD-LLALQDNHY----NISHQINKLSFG-------
P.vulgarisERV-A2          209 LEVNKVAGNFHFATGKSFLQSAIFLAD-LLALQDNHY----NISHRINKLSFG-------
G.maxERV-A3               209 LEVNKVAGNFHFATGKSFLQSAIFLAD-LLALQDNHY----NISHRINKLSFG-------
G.maxERV-A4               209 LEVNKVAGNFHFATGKSFLQSAIFLAD-VLALQDNHY----NISHRINKLSFG-------
S.lycopersicumERV-A2      209 LEVNKVAGNFHFAAGKSFHQSTFQLFE-LISLQSDTY----NISHRVNKLAFG-------
S.tuberosumERV-A2         209 LEVNKVAGNFHFAAGKSFHQSTFQLFE-LISLQSDTY----NISHRVNKLAFG-------
P.persicaERV-A2           209 LEVNKVAGNFHFVSGKSFHQSNIHVQD-LLAFQTDSY----NISHKINRLAFG-------
T.cacaoERV-A2             209 LEVNKVAGNFHFAPGKSFHQTNIFLSD-LLAFQKDSY----NISHRINRLAFG-------
P.trichocarpaERV-A3       209 LEVNRVAGNFHFVPGKSFHQSNFQLLD-LLDMQKESY----NISHRINRLAFG-------
P.trichocarpaERV-A4       209 LEVNRVAGSFHFAPWKSFHLSNFLIQD-LLDLQKDSY----NISHRINRLAFG-------
C.sativusERV-A1           207 LEVNKVAGNFHFAPGRGFQLSYFQIHNPLASFQWDAF----NISHRINRLTFG-------
V.viniferaERV-A2          209 LEVNKVAGNFHFSPGKGFYQSNIHVND-LLAISKDGY----NISHRINKLAFG-------
B.rapaERV-A4              205 VEVNKVAGNFHFVPGQSFHQSGFQFHD-MIMFQQGNY----NISHKVNRLAFG-------
B.rapaERV-A3              205 LEVNKVAGNFHFVPGQSFHQSGFQFGD-LIFFQQGNY----NISHKVNRLAFG-------
E.salsugineumERV-A2       209 LEVNKVAGNFHFVPGQSFHHSGFQFHD-LIMFHQGNY----NISHKVNRLAFG-------
```

```
                               -------->                                      BLOCK#4---->
A.thalianaERV-A2          209 LEVNKVAGNFHFIPGQSFHQSGFQFHD-MLLFQQGNY----NISHKVNRLAFG-------
C.rubellaERV-A2           209 LEVNKVAGNFHFIPGQSFHQSGFQFHD-MLLFQQGNY----NISHTVNRLAFG-------
B.rapaERV-A2              209 LEVNKVAGSFHFVPGKTFRQSGFHLQD-LLVFQGDSY----NISHKVNSLTFG-------
E.salsugineumERV-A3       209 LEVNKVAGSFHFVPGKTFRHSGFHLQD-LLAFQGDTY----NISHKVNRLTFG-------
B.distachyonERV-A1        209 VEINKVAGNFHFAPGKSFQQSNVHVHD-LLPFQKDSF----NVSHKINKLSFG-------
O.sativaERV-A1            209 LEVNKVAGNFHFAPGKSFQKANVHVHD-LLPFQKDSF----NVSHKINKLSFG-------
S.bicolorERV-A1           209 IEVNKVAGNFHFAPGKSFQQSNVHVHD-LLPFQKDSF----NVSHKINRLSFG-------
Z.maysERV-A1              209 IEVNKVAGNFHFAPGKSFQQSNVHVHD-LLPFQKDSF----NVSHKINRLSFG-------
S.lycopersicumERV-A1      209 LEVNKVAGNFHFAPGKSFQQSNVHVHD-LLTFQKDSY----NISHKINRLTYG-------
S.tuberosumERV-A1         209 LEVNKVAGNFHFAPGKSFQQSNVHVHD-LLTFQKDSY----NISHKINRLTYG-------
B.rapaERV-A1              209 LEVNKVAGNFHFAPGKSFHQGGVHVHD-LLAFQKDSF----NISHKINRLTFG-------
E.salsugineumERV-A1       209 LEVNKVAGNFHFAPGKSFHQAGVHVHD-LLAFHKDSF----NISHKINRLTFG-------
A.thalianaERV-A1          209 LEVNKVAGNFHFAPGKSFHQSGVHVHD-LLAFQKDSF----NISHKINRLTYG-------
C.rubellaERV-A1           209 LEVNKVAGNFHFAPGKSFHQSGVHVHD-LLAFQKDSF----NISHKINRLTYG-------
V.viniferaERV-A1          209 LEVNKVAGNFHFAPGKSFQQSNIHVHD-LLAFQKDSF----NISHKINRLAFG-------
C.sativusERV-A2           209 LEVNKVAGNFHFAPGKSFQQSNVHVHD-LLAFQKDSF----NISHKINRLAFG-------
M.truncatulaERV-A1        209 LEVNKVAGNFHFAPGKSFQQSGVHVHD-LLAFQKESF----NLSHHINRIAFG-------
P.persicaERV-A1           209 LEVNKVAGNFHFAPGKSFQQSNVHVHD-LLAFQKDSF----NISHTINRLSFG-------
P.vulgarisERV-A1          209 LEVNKVAGNFHFAPGKSFQQSGVHVHD-LLAFQKDSF----NLSHHINRLSYG-------
G.maxERV-A1               209 LEVNKVAGNFHFAPGKSFQQSGVHVHD-LLAFQKDSF----NLSHHINRLAFG-------
G.maxERV-A2               209 LEVNKVAGNFHFAPGKSFQQSGVHVHD-LLAFQKDSF----NLSHHINRLTFG-------
T.cacaoERV-A1             209 LEVNKVAGNFHFAPGKSFQQSNVHVHD-LLAFQKDSF----NISHKINRLAFG-------
P.trichocarpaERV-A1       209 LEVNKVAGNFHFAPGKSFQQSGVHVHD-LLAFQKDSF----NITHKINRLTFG-------
P.trichocarpaERV-A2       200 LEVNKVAGNFHFAPGKSFQQSGVHVHD-LLAFQKDSF----NTSHKINRLAFG-------
C.reinhardtiiERV-B        171 MEVKRVAGRLHL----SVHQNMVFQML-PQLLGTHHIPKILNMSHVIKHLGFG-------
V.carteriERV-B            169 MDVKRVAGRLHF----SVHQNMVFQML-PQLLGAHRIPKVANISHTIKHLGFG-------
C.subellipsoideaERV-B     159 LDLQRVAGNFRV----SVHVEDFFALT-RLQADTTGI----NSSHIIHRVSFG-------
K.flaccidumERV-B          181 LDVERVAGNFHI----SVHGQSYYVLG--QVFASAST---VNVSHHIESVSFG-------
P.patensERV-B             176 LDVERVAGNFHI----SMHGLSLYVAS--KIFEAGYE---VNVSHVIHDLSFG-------
S.moellendorffiiERV-B     167 LDVERVAGNFHI----SMHGMSLQVAR--QIFHSVKE---VNVSHIINDLSFG-------
S.bicolorERV-B            178 LDVQRVAGNFHI----SVHGLNIFVAE--KIFEGSSH---VNVSHVIHELSFG-------
Z.maysERV-B               178 LDVQRVAGNFHI----SVHGLNIFVAE--KIFEGSNH---VNVSHVIHELSFG-------
B.distachyonERV-B         177 LDVQRVAGNFHI----SVHGLNIYVAE--KIFEGSSH---VNVSHVIHELSFG-------
O.sativaERV-B             177 LDVQRVAGNFHI----SVHGLNIFVAE--KIFDGSSH---VNVSHIIHDLSFG-------
B.rapaERV-B2              177 LDVQRVAGNFHI----SVHGLNIYVAQ--MIFGGSKN---VNVSHMIHDLSFG-------
B.rapaERV-B1              180 LDVQRVAGNFHV----SVHGLNIYVAQ--MIFGGSKN---VNVSHMIHDLSFG-------
E.salsugineumERV-B        176 LDVQRVAGNFHV----SVHGLNIYVAQ--MIFGSSKN---VNVSHMIHDLSFG-------
A.thalianaERV-B           184 LDVQRVAGNFHI----SVHGLNIYVAQ--MIFGGSKN---VNVSHMIHDLSFG-------
C.rubellaERV-B            184 LDVQRVAGNFHI----SVHGLNIYVAQ--MIFGGSKN---VNVSHMIHDLSFG-------
M.truncatulaERV-B         185 LDVQRVAGNFHI----SVHGLNIYVAQ--MIFDAGKN---VNVSHVIHDLSFG-------
P.vulgarisERV-B           179 LDVQRVAGNFHI----SVHGLNIYVAQ--MIFDGAKN---VNVSHVIHDLSFG-------
G.maxERV-B1               182 LDVQRVAGNFHI----SVHGLNIYVAQ--MIFDGAKN---VNVSHFIHDLSFG-------
G.maxERV-B2               178 LDVQRVAGNFHI----SVHGLNIYVAQ--MIFDGAKN---VNVSHFIHDLSFG-------
C.sativusERV-B            176 LDVQRVAGNFHI----SVHGLNIFVAQ--MIFGGSKH---VNVSHMIHDLSFG-------
S.lycopersicumERV-B1      176 LDVQRVAGNFHI----SVHGLNIFVAQ--MIFGGTTH---VNVSHIIHDLSFG-------
S.tuberosumERV-B1         169 LDVQRVAGNFHI----SVHGLNIFVAQ--MIFGGTTH---VNVSHIIHDLSFG-------
S.lycopersicumERV-B2      178 LDVQRVAGNFHL----SVHGLNIFVAQ--MIFEKSTH---VNVSHIIHDLSFG-------
S.tuberosumERV-B2         178 LDVQRVAGNFHL----SVHGMNIFVAQ--MIFEKSTH---VNVSHIIHDLSFG-------
P.persicaERV-B            179 LDVQRVAGNFHI----SVHGLNIFVAQ--MIFEGSKN---VNISHIIHDLSFG-------
P.trichocarpaERV-B1       179 LDVQRVAGNFHI----SVHGLNIFVAQ--MIFDGAKH---VNVSHIIHDLSFG-------
P.trichocarpaERV-B2       179 LDVQRVAGNFHI----SVHGLNIFVAQ--MIFDGAKH---VNVSHIIHDLSFG-------
V.viniferaERV-B           179 LDVQRVAGNFHI----SVHGLNIFVAQ--MIFDGAIH---VNVSHIIHDLSFG-------
T.cacaoERV-B              179 LDVQRVAGNFHI----SVHGLNIYVAQ--MIFGGATH---VNVSHMIHDLSFG-------
```

```
C.subellipsoideaPDI-C   333 HQSLAKLHP--AGLSDDWADKLAGQ----DFFSR-AAK---ATFEHYMQVVLTTIEPSKH
C.reinhardtiiPDI-C      338 YQQLKRLHP--AGLTADWADKLHDQ----LFVSE-HTQ---STHEHYLQVVLTTIEPRHS
V.carteriPDI-C          338 YQQLKRLHP--AGLTHDWADQLRDQ----FFLSE-HPQ---STHEHYLQIVLTSIEPRRS
K.flaccidumPDI-C        388 YHEIDRIWSHHPSAGED---RLTGR----YFHSH-SDN---LTHEHYLQVVLTSVEPLKL
B.rapaPDI-C1            345 LTDMRRLLPYIGQSHD----KLNEK----AFINQ-HEFGANVTIEHYLQVVKTEVITRRT
B.rapaPDI-C2            346 LTDLKRLLPYLGQSHD----KLDEK----AFINQ-HEFGANVTIEHYLQIVKTEVITRRY
E.salsugineumPDI-C1     345 LTDMRRLLPYLGQSHG----RLDEK----AFINQ-HEFGANVTIEHYLQIVKTEVITRKS
A.thalianaPDI12         345 LTDMKRLLPYLGLSHD----RLDGK----AFINQ-HEFGANVTIEHYLQTVKTEVITRRS
C.rubellaPDI-C1         345 LTDMKRLLPYLGQSHD----RLDGK----AFINQ-HEFGANVTIEHYLQIVKTEVITRRS
B.rapaPDI-C5            339 LTDMKRLLPYLGLSHD----RLNGK----WFVNE-GKFAANVTIEHYLQVVKTEVVSRRF
A.thalianaPDI13         346 WTDMKRLLPYLGQSYD----RLNGK----SFINE-RQLDANVTIEHYLQIIKTEVISRRS
C.rubellaPDI-C3         345 WTDMKRLLPYLGQSHD----RLNGK----SFINQ-RQLDANVTIEHYLQIVKTEVISRRF
B.rapaPDI-C6            345 LTDMKRLMPYLGLSHD----RLNSK----WFVNE-GQFAANVTIEHYLQVVKTEVVSRRF
E.salsugineumPDI-C3     346 LTDMKRLLPYLGQSHD----RLNGK----WFLNQ-GQFAANVTIEHYLQIVKTEVVSRRF
S.bicolorPDI-C          346 LDEFTRLTPYLSGYND----RLAGQ----SYIVKHGEVNANVTIEHYLQVVKTEIVTQRS
Z.maysPDI-C             344 LHEFIRLTPYLRGYHD----RLAGQ----SYTVKHGEVNANVTIEHYLQVVKTELVTQRS
B.distachyonPDI-C       346 FSELKRLIPYVGGHHD----RLAGQ----SYIVKHGDNNANVTIEHYLQIVKTELVTLRS
O.sativaPDI-C           346 FNELKRLTPYVGGHHD----RLAGQ----SYIVKHGDVNANVTIEHYLQIVKTELVTLRS
B.rapaPDI-C4            345 FSDLKRLAPYLGGSHN----RLDDR----SFINQ-HDLGPNVTIEHYLQIVKTEVLKSNG
A.thalianaPDI7          345 FSEFKRLSPYLGLSHD----RLDGR----SFINQ-RDLGPNVTIEHYLQIVKTEVVKSNG
C.rubellaPDI-C2         345 FSELKRLSPYLGRNHD----RLDGR----PFINQ-RDLGPNVTIEHYLQIVKTEVVKSNG
B.rapaPDI-C3            343 FTDLKRLSPYLGQSHD----RLNGR----PFINQ-RDLGPNVTIEHYLQIVKTEVLKSNG
E.salsugineumPDI-C2     345 FADLKRLSPYLGRSHD----RLDGR----PFINQ-RDLGPNVTIEHYLQIVKTEVVKSNG
S.lycopersicumPDI-C     346 MSDIKILLPHLGRSHD----RLNGN----SYVTNPRDSTENVTIEHFLQVVKTEVMTRSY
S.tuberosumPDI-C        346 MSDIKILLPHLGRSHD----RLNGN----SYVTNPRDSTENVTIEHFLQVVKTEVMTRSY
M.truncatulaPDI-C1      345 MSDVQRLIPYVGNSHD----RLDGL----SFINS-HDFGANVTLEHYLQIVKTEVITRQG
P.vulgarisPDI-C1        345 MSDVKRLIPYVGSSHD----RLNGL----SFINT-RDFGANVTMEHYLQIVKTEVITRKD
G.maxPDI-C1             345 MSDVKRLIPYVGSSHD----RLNGR----SFINT-HDLGANVTMEHYLQIVKTEVITRKD
G.maxPDI-C2             345 MSDVKRLIPYVGSSHD----RLNGR----SFINT-HDLGANVTIEHYLQIVKTEVITRKE
M.truncatulaPDI-C2      342 MIDVKHWIPYLGINHD----RLNGR----SFINT-RDLEGNVTIEHYIQVVKTEVITRKG
P.vulgarisPDI-C2        345 MSDVKLLIPYLGSSHD----RLSGR----SFSNT-RDFGANVTIEHYIQIVKTEVVNRKG
G.maxPDI-C3             345 MSDVKLLIPYIGSSHD----RLNGR----SFINT-RDLGANVTIEHYIQIVKTEVVTRKG
G.maxPDI-C4             345 MSDVKLLIPYIGSSHD----RLNGR----SFTNT-HDLGANVTIEHYIQIVKTEVVTRNG
V.viniferaPDI-C         346 MSDMKRVLPYIGGSHD----RLNGR----SYISHPSDSNANVTIEHYLQVVKTEVITTRD
C.sativusPDI-C          346 FSDAKQLIPYIGISHD----RLNGR----SFINQ-RDLGANVTIEHYLQIVKTEVLTRRS
P.persicaPDI-C          345 MSDVKRLVPYLGGSHD----RLNGR----SFINH-RDLGANVTIEHYLQIVKSEVITGRN
T.cacaoPDI-C            342 LSDVKRLIPYIGRSHD----RLNGR----SFINH-RELDANVTIEHYLQIVKTEVVTRRS
P.trichocarpaPDI-C1     346 MSDVKRLLPYIGRSHD----KLNGR----SFINH-RDVGANVTIEHYLQVVKTEVVTRRS
P.trichocarpaPDI-C2     346 MSDVKRLIPHIGRSHD----KLNGR----SFINH-RDVGANVTIEHYLQVVKTEVVTRRS
S.moellendorffiiPDI-C   336 RRELYRIYPHLASVYDTVEANLTGR----IYVSQ-HEN---ITHDHYLQVVKTEVVSLRK
P.patensPDI-C           351 VHWVNEMLPALDSNID----RLTGQ----VFPSE-YEN---ITHDHYLQVVKTEVITLHR
C.reinhardtiiERV-A      291 ---------EPYPGMKN----PLDGAKAGQAAAAAAAAT---GMFQYFLKVVPTSYTDLSN
V.carteriERV-A          260 ---------APYPGMKN----PLDNA------KAGYKSAAATGMYQYFLKVVPTSYTGIDN
C.subellipsoideaERV-A   252 ---------HEYPGMTN----PLDRV-NLPKFNTR-NPQGLPGAYQYFLKVVPTIYVNSHN
K.flaccidumERV-A        257 ---------ADYPGVVH----PLDNV----RRIQE-ADG---GMYQYFIKVVPTIYTDLSG
B.distachyonERV-A3      256 ---------TEFPGVVN----PLDGA----QWTQP-ASD---GTYQYFIKVVPTNYTDTRG
O.sativaERV-A3          256 ---------TEFPGVVN----PLDGA----QWTQP-ASD---GTYQYFIKVVPTIYTDLRG
Z.maysERV-A3            256 ---------TEFPGVVN----PLDGA----QWTQP-ASD---GTYQYFIKVVPTIYTDIRG
Z.maysERV-A2            256 ---------TEFPGVVN----PLDGA----QWTQP-ASD---GTYQYFIKVVPTIYTDIRG
S.bicolorERV-A3         256 ---------TEFPGVVN----PLDGA----QWIQP-ASD---GTYQYFIKVVPTIYTDIRG
P.patensERV-A1          259 ---------VRYPGAVN----PLDKL----ERIQT-TTH---GMYQYFIKVVPTVYTDTRG
P.patensERV-A4          257 ---------AHFPGRVN----PLDKI----TSIQK-DQN---GMYQYFIKVVPTVYTDIRG
P.patensERV-A2          257 ---------AHFPGAVN----PLDKV----TNIQK-DLN---GMYQYFIKVVPTVYTDIKG
P.patensERV-A3          257 ---------AYFPGAVN----PLDKV----TSIQK-DQN---GMFQYFIKVVPTVYTDIKG
S.bicolorERV-A2         256 ---------EEFPGVVN----PLDGV----EWIQD-NSNGLTGMYQYFVKVVPTIYTDIRG
B.distachyonERV-A2      256 ---------KEFPGVVN----PLDGV----EWKQE-QATGLTGMYQYFVKVVPTIYTDIRG
O.sativaERV-A2          256 ---------VEFPGVVN----PLDGV----EWIQE-HTNGLTGMYQYFVKVVPTIYTDIRG
S.moellendorffiiERV-A   257 ---------ARFPGVVN----PLDKE----KRIQK-FPS---AMYQYFIKVVPTAYTDMTG
C.sativusERV-A3         257 ---------NHYDGLVN----PLDGV----HWEYN-EQN---VMHQYFVKVVPTIYKNIRG
M.truncatulaERV-A2      257 ---------HHYPGLVN----PLDGI----KWVQG-NDH---GMCQYFIKVVPTVYTDIRG
P.vulgarisERV-A2        257 ---------HHFPGLVN----PLDGV----KWVQG-PTH---GMYQYFIKVVPTIYTDIRG
G.maxERV-A3             257 ---------HHFPGLVN----PLDGV----KWVQG-PAH---GMYQYFIKVVPTIYTDIRG
G.maxERV-A4             257 ---------HHFPGLVN----PLDGV----RWVQG-PTH---GMYQYFIKVVPTIYTDIRG
S.lycopersicumERV-A2    257 ---------DSIPGVVN----PLDGV----QWTQE-AQN---GMYQYFIKVVPTIFKGVRG
S.tuberosumERV-A2       257 ---------DSIPGVVN----PLDGV----QWTQE-AQN---GMYQYFIKVVPTIYKGVRG
P.persicaERV-A2         257 ---------DYFPGKLN----PLDGV----QWRQE-TPN---GMYQYFIKVVPTLYTNIRG
T.cacaoERV-A2           257 ---------EYFPGVVN----PLDGA----QWIHE-TSN---GMYQYFIKVVPTIYTDIRG
P.trichocarpaERV-A3     257 ---------DYFPGVVN----PLDGI----QLMHE-TQN---GVQQFFIKVVPTIYTDIRG
P.trichocarpaERV-A4     257 ---------DYFPGVVN----PLAGI----QLMHD-TPN---GVQQFFIKVVPTIYTDIRG
C.sativusERV-A1         256 ---------DDFPGVVN----PLDGV----QWNQG-TLS---GMFQYFIKVVPTVYKAVNG
V.viniferaERV-A2        257 ---------DHFPGVVN----PLDGA----QWFQD-APD---GMYQYFIKVVPTIYTDIRG
B.rapaERV-A4            253 ---------DFFPGVVN----PLDGV----QWNQD-KQN---GVYQYFIKVVPTIYTDVHG
B.rapaERV-A3            253 ---------DFFPGVVN----PLDGV----QWNQE-KQN---GVYQYFVKVVPSIYTDVHG
E.salsugineumERV-A2     257 ---------DFFPGVVN----PLDGV----QWNQE-KQN---GVYQYFIKVVPSVYTDAHE
```

```
A.thalianaERV-A2        257 ---------DFFPGVVN----PLDGV----QWNQG-KQS---GVYQYFIKVVPSIYTDVHQ
C.rubellaERV-A2         257 ---------DFFPGVVN----PLDGV----QWNQG-KQS---GVYQYFIKVVPSIYTDVHR
B.rapaERV-A2            257 ---------DRFPGVVN----PLDGV----QWTQE-TLN---GMYQYFIKVVPTEYTDVRG
E.salsugineumERV-A3     257 ---------DYFPGVVN----PLDSV----QWTQE-TLN---GMYQYFIKVVPTEYTDVKE
B.distachyonERV-A1      257 ---------EPFPGVVN----PLDGA----HWFQH-SPY---GMYQYFVKVVPTVYSHINE
O.sativaERV-A1          257 ---------QRFPGVVN----PLDGA----QWMQH-SSY---GMYQYFIKVVPTVYTDINE
S.bicolorERV-A1         257 ---------EYFPGVVN----PLDGA----SWVQH-SSY---GMYQYFIKVVPTVYTDINE
Z.maysERV-A1            257 ---------EYFPGVVN----PLDGA----NWVQH-SSY---GMYQYFIKVVPTVYTDINE
S.lycopersicumERV-A1    257 ---------EYFPGVVN----PLDGV----KWTQE-TPH---GMYQYFIKVVPTVYTDVSG
S.tuberosumERV-A1       257 ---------EYFPGVVN----PLDGV----MWTQE-TPH---GMYQYFIKVVPTVYTDVSG
B.rapaERV-A1            257 ---------DYFPGVVN----PLDKV----QWSQD-TPN---AMYQYFIKVVPTVYTDISG
E.salsugineumERV-A1     257 ---------DYFPGVVN----PLDRV----QWGQD-TPN---AMYQYFIKVVPTVYTDISG
A.thalianaERV-A1        257 ---------DYFPGVVN----PLDKV----EWSQD-TPN---AMYQYFIKVVPTVYTDIRG
C.rubellaERV-A1         257 ---------DYFPGVVN----PLDKV----QWSQD-TPN---AMYQYFIKVVPTVYTDIRG
V.viniferaERV-A1        257 ---------DYFPGVVN----PLDGV----QWIQA-TPS---GMYQYFIKVVPTVYTHVSG
C.sativusERV-A2         257 ---------EYFPGVVN----PLDSV----QWKQE-TPS---ATYQYFIKVVPTVYNSVSG
M.truncatulaERV-A1      257 ---------DYFPGVVN----PLDRV----HWTQE-TPS---GMYQYFIKVVPTMYTDVSG
P.persicaERV-A1         257 ---------DYFPGVVN----PLDGV----HWAQA-TPS---GMYQYFIKVVPTVYTDVSG
P.vulgarisERV-A1        257 ---------EYFPGVVN----PLDNV----HWIQE-TPS---GMYQYFIKVVPTVYTDVNG
G.maxERV-A1             257 ---------EYFPGVVN----PLDNV----HWTQE-TPS---GMYQYFIKVVPTVYTDVSG
G.maxERV-A2             257 ---------EYFPGVVN----PLDNV----HWTQE-TPS---GMYQYFIKVVPTVYTDVSG
T.cacaoERV-A1           257 ---------DYFPGVVN----PLDGV----HWTQE-QPS---GMYQYFIKVVPTVYTDVSG
P.trichocarpaERV-A1     257 ---------EYFPGVVN----PLDGV----QWTQE-TPS---GMYQYFIKVVPTVYTDVSG
P.trichocarpaERV-A2     248 ---------EYFPGVVN----PLDGV----QWTQE-TPS---GMYQYFIKVVPTVYTDVSG
C.reinhardtiiERV-B      219 ---------PHYPGQLN----PLDGY----VRMVGREPF----SYKYFLKVVPTEYYNRLG
V.carteriERV-B          217 ---------PHYPGQLN----PLDGY----VRMVK-GPP---QSFKYFLKVVPTEYYNRLG
C.subellipsoideaERV-B   203 ---------PTFPGQVN----PLDGA----ERILD-KES---GTFKYFLKVVPTEYVKLDG
K.flaccidumERV-B        225 ---------PPIPGTTN----PLDGY----TRILK-TEQ-ESGTFKYFIKVVPTEYFPLKG
P.patensERV-B           220 ---------PTYPGHHN----PLDGS----ERILH-DTS---GTFKYFLKIVPTEYHYLHG
S.moellendorffiiERV-B   211 ---------PKYPGIHN----PLDRT----VRILR-DTA---GTFKYFIKIVPTEYRYLNG
S.bicolorERV-B          222 ---------PKYPGIHN----PLDET----SRILH-DTS---GTFKYYIKVVPTEYKYLSK
Z.maysERV-B             222 ---------PKYPGIHN----PLDET----SRILH-DTS---GTFKYYIKVVPTEYKYLSK
B.distachyonERV-B       221 ---------PKYPGIHN----PLDDT----TRILH-DAS---GTFKYYIKVVPTEYRYLSK
O.sativaERV-B           221 ---------PKYPGIHN----PLDET----TRILH-DTS---GTFKYYIKIVPTEYRYLSK
B.rapaERV-B2            221 ---------PKYPGIHN----PLDGT----NRILH-DTS---GTFKYYIKIVPTEYRYLSK
B.rapaERV-B1            224 ---------PKYPGIHN----PLDDT----NRILR-DTS---GTFKYYIKIVPTEYRYLSK
E.salsugineumERV-B      220 ---------PKYPGIHN----PLDDT----NRILR-DTS---GTFKYYIKVVPTEYRYLSK
A.thalianaERV-B         228 ---------PKYPGIHN----PLDDT----NRILH-DTS---GTFKYYIKIVPTEYRYLSK
C.rubellaERV-B          228 ---------PKYPGIHN----PLDDT----NRMLH-ETS---GTFKYYIKIVPTEYRYLSK
M.truncatulaERV-B       229 ---------PKYPGIHN----PLDET----SRILH-DAS---GTFKYYIKIVPTEYRYISK
P.vulgarisERV-B         223 ---------PKYPGIHN----PLDDT----NRILH-DTS---GTFKYYIKVVPTEYRYISK
G.maxERV-B1             226 ---------PKYPGLHN----PLDDT----TRILH-DTS---GTFKYYIKVVPTEYRYISK
G.maxERV-B2             222 ---------PKYPGLHN----PLDDT----TRILH-DTS---GTFKYYIKVVPTEYRYISK
C.sativusERV-B          220 ---------PKYPGIHN----PLDGT----VRILR-DTS---GTFKYYIKIVPTEYKYISK
S.lycopersicumERV-B1    220 ---------PKYPGGHN----PLDGT----ERILR-GAS---GTFKYYIKIVPTEYRYLSK
S.tuberosumERV-B1       213 ---------PKYPGSHN----PLDGT----ERILR-GAS---GTFKYYIKIVPTEYRYLSK
S.lycopersicumERV-B2    222 ---------PKYPGIHN----PLDGT----SRILR-GTS---GTFKYYIKVVPTEYRYISK
S.tuberosumERV-B2       222 ---------PKYPGIHN----PLDGT----SRILR-GSS---GTFKYYIKVVPTEYRYISK
P.persicaERV-B          223 ---------PKYPGIHN----PLDGT----ERILH-DTS---GTFKYYIKVVPTEYRYISK
P.trichocarpaERV-B1     223 ---------PKYPGIHN----PLDGT----TRILH-ETS---GTFKYYIKIVPTEYRYISK
P.trichocarpaERV-B2     223 ---------PKYPGIHN----PLDGT----ARILR-ETS---GIFKYYIKIVPTEYRYISK
V.viniferaERV-B         223 ---------PKYPGLHN----PLDGT----VRILR-GAS---GTFKYYIKIVPTEYRYISK
T.cacaoERV-B            223 ---------PKYPGLHN----PLDGT----VRILH-DTS---GTFKYYIKIVPTEYRYISK
```

```
                                                                    BLOCK#5-------------------------
C.subellipsoideaPDI-C  383 RPELSY-DAYEYTVH-----SHTYDTADIPAAKFTYDLSPIQILVSEKRRAWYHFVTTTC
C.reinhardtiiPDI-C     388 RHTGNY-DAYEYTAH-----SHSYQSDSIPSARFTYDLSPIQILVHETSKPWYQFLTTSC
V.carteriPDI-C         388 RHSGNY-DAYEYTAH-----SHTYQSDAIPSARFTYDLSPIQILVQETARPWYQFLTTSC
K.flaccidumPDI-C       437 PAHQYV-QSYDYTAH-----SNVFNSAAVPVAKFHYELSPMQVVITENSRSFSHFITNVC
B.rapaPDI-C1           396 AQEHSLVEEYEYTAH-----SSIAQTYYLPVAKFHFELSPMQIMITENPKSFSHFITNLC
B.rapaPDI-C2           397 GQEHSLTEEHEYTAH-----SSITQTYYLPVAKFHFELSPMQILITENPKSFSHFITNLC
E.salsugineumPDI-C1    396 GQEHSLIEEYEYTAH-----SSVAQTYYLPVAKFHFELSPMQILITENPKSFSHFITNLC
A.thalianaPDI12        396 GQEHSLIEEYEYTAH-----SSVAQTYYLPVAKFHFELSPMQILITENPKSFSHFITNLC
C.rubellaPDI-C1        396 GLEQAVIEEYEYTAH-----SSVAQTYYLPVAKFHFELSPMQILITEDPKSFSHFITNLC
B.rapaPDI-C5           390 GQEHSVIEEYEYTAH-----SSVAHGYYYPVAKFHFDLSPMQVLISENPKSFSHFITNVC
A.thalianaPDI13        397 GQEHSLIEEYEYTAH-----SSVARSYHYPEAKFHFELSPMQVLISENPKSFSHFITNVC
C.rubellaPDI-C3        396 GREHSLIEEYEYTAH-----SNVAHSYHYPEAKFHFELSPMQVLISENPKSFSHFITNVC
B.rapaPDI-C6           396 GQEHSVIEEYEYTAH-----SSVAHGYYYPVAKFRFDLSPMQVLISENPKSFSHFITNVC
E.salsugineumPDI-C3    397 GQEHSLIEEYEYTAH-----SNVAHSYYYPVAKFHFELSPMQVLISENPKSFSHFITNVC
S.bicolorPDI-C         398 SKELKVLEEYEYTAH-----SSLVHSFYVPVVKFHVEPSPMQVLVTEVPRSFSHFITNVC
Z.maysPDI-C            396 SKELKVLEEYEYTAH-----SSLVHSFYVPVVKFHFEPSPMQVLVTEVPKSFSHFITNVC
B.distachyonPDI-C      398 SKELKVFEEYEYTAH-----SSLVHSFYVPVVKFHFEPSPMQVLVTELPKSFSHFITNVC
O.sativaPDI-C          398 SKELKLVEEYEYTAH-----SSLVHSFYVPVVKFHFEPSPMQVLVTELPKSFSHFITNVC
B.rapaPDI-C4           396 HAMI---EEYEYTAH-----SSVAHTYYLPVAKFHFELSAMQVLITENSKSFSHFITNVC
A.thalianaPDI7         396 QALV---EAYEYTAH-----SSVAHSYYLPVAKFHFELSPMQVLITENSKSFSHFITNVC
C.rubellaPDI-C2        396 QVLI---EEYEYTAH-----SSVAQSYYLPVAKFHFELSPMQVLITENSKSFSHFITNVC
B.rapaPDI-C3           394 HAMV---EEYEYTAH-----SSVAHSYYLPVAKFHFELSPMQVLITENSRSFSHFITNVC
E.salsugineumPDI-C2    396 QALI---EEYEYTAH-----SSVAQSYYLPVAKFHFELSPMQVLITETSKSFSHFITNVC
S.lycopersicumPDI-C    398 KLV----EEYEYTAH-----SSLVHSLHIPVAKFHYEPSPMQVLITENPKSFSHFLTNVC
S.tuberosumPDI-C       398 KLV----EEYEYTAH-----SSLVHSLHIPVVKFHYEPSPMQVLITENPKSFSHFLTNVC
M.truncatulaPDI-C1     396 YQLV---EEYEYTAH-----SSLAHSLHVPVARFHLQLSPMQVLITEDHKSFSHFITNVC
P.vulgarisPDI-C1       396 HKLV---EEYEYTAH-----SSVAQSLHIPVAKFHLELSPMQVLITENPKSFSHFITNVC
G.maxPDI-C1            396 YKLV---EEYEYTAH-----SSVAQSLHIPVAKFHLELSPMQVLITENQKSFSHFITNVC
G.maxPDI-C2            396 YKLV---EEYEYTAH-----SSVAQSLHIPVAKFHLELSPMQVLITENQKSFSHFITNVC
M.truncatulaPDI-C2     393 YKLI---EEYEYTAH-----SSVAHSVNIPVARFHLELSPMQVLITENQKSFSHFITNVC
P.vulgarisPDI-C2       396 YKLI---EEYEYTAH-----SSVAHSVDIPVAKFHIELSPMQVLITENQRSFSHFITNVC
G.maxPDI-C3            396 YKLI---EEYEYTAH-----SSVAHSLDIPVAKFHLELSPMQVLITENQRSFSHFITNVC
G.maxPDI-C4            396 YKLI---EEYEYTAH-----SSVAHSVDIPAAKFHLELSPMQVLITENQRSFSHFITNVC
V.viniferaPDI-C        398 HKLV---EEYEYTAH-----SSLVQSLYIPVAKFHFELSPMQVLVTENRKSFWHFITNVC
C.sativusPDI-C         397 GKLL---EEYEYTAH-----SSVSQSLYIPVVKFHFVLSPMQVVITENQKSFSHFITNVC
P.persicaPDI-C         396 HKLI---EEYEYTAH-----SSLVQSLQIPVAKFHFELSPMQVLITENQKSFSHFITNVC
T.cacaoPDI-C           393 SREHTLIEEYEYTAH-----SSLAQSIYIPVTKFHFELSPMQVLITENPKSFSHFITNVC
P.trichocarpaPDI-C1    397 SSERKLIEEYEYTAH-----SSLSQTVYMPTAKFHFELSPMQVLITENSKSFSHFITNVC
P.trichocarpaPDI-C2    397 SAEHKLIEEYEYTAH-----SSLAQTVYMPTAKFHFELSPMQVLITENPKSFSHFITNVC
S.moellendorffiiPDI-C  388 RKEFSLLEQYDYTSH-----SNTIQNTNVPVAKFHYELSPMQVLVKENPKSFSHFITNVC
P.patensPDI-C          399 KQDLRVLEQYDYTAH-----SNMIQSTKVPVVKFHYELSPMQVLVKENPKSFSHFLTNLC
C.reinhardtiiERV-A     336 KTLST--NQFSVTENFRE--AQGGAGRTLPGVFFFYDLSPIKVKIVEHGSSFLSFLTSVC
V.carteriERV-A         302 KTLAT--NQFSVTENFRE-SSQGGAGKTLPGVFFFYDLSPIKVRIVEHSSSFLSFLTSVC
C.subellipsoideaERV-A  298 HTINS--NQYSVTEHFKG--SQDFQAQ-LPGVFFYYDLSPIKVKYHETRMSFLHFLTSVC
K.flaccidumERV-A       297 HVIST--NQFSVTEHFRE--QEVASGRSLPGVFFFYDLSPIKVKFTEQRQSFLHFVTNVC
B.distachyonERV-A3     296 RKIDS--NQFSVTEHFRD--GNV-HPRPQPGVFFFYDFSPIKVIFTEENKSFLHYLTNLC
O.sativaERV-A3         296 RKIHS--NQFSVTEHFRD--GNI-RPKPQPGVFFFYDFSPIKVIFTEENSSLLHYLTNLC
Z.maysERV-A3           296 RGIHS--NQFSVTEHFRD--GNV-RPKSQPGVFFFYDFSPIKVIFTEENRSLLHYLTNLC
Z.maysERV-A2           296 HNIHS--NQFSVTEHFRD--GNV-RPKPQPGVFFFYDFSPIKVIFTEESRSLLHYLTNLC
S.bicolorERV-A3        296 HNIHS--NQFSVTEHFRD--GNI-LPKPQPGVFFFYDFSPIKVIFTEENRSLLHYLTNLC
P.patensERV-A1         299 RKIST--NQFAVTDHFKG--VGPGEDHALPGVFFFYDLSPIKVKFTEKRMSFFHFLTNVC
P.patensERV-A4         297 SEIAT--NQFSVTEHYTA--GDHG-PRVVPGVFFFYDLSPIKVKFTEKRPSFLHFLTTVC
P.patensERV-A2         297 RKIST--NQFSVTEHYTA--GDHG-PRFVPGVFFFYDLSPIKVKFSEERPSFLHFLTNVC
P.patensERV-A3         297 RKIST--NQFSVMEHYTA--GDHGPRV-IPGVFFFYDLTPIKVKFTEERPSFLHFLTNVC
S.bicolorERV-A2        299 RKIYS--NQFSVTEHFR---EAIGYPRPPPGVYFFYEFSPIKVDFTEENTSLLHFLTNIC
B.distachyonERV-A2     299 RKIHS--NQFSVTEHFR---EAIGFPRPPPGVYFFYEFSPIKVDFTEENTSLLHFLTNIC
O.sativaERV-A2         299 RKINS--NQFSVTEHFR---EAIGYPRPPPGVYFFYEFSPIKVDFTEENTSLLHFLTNIC
S.moellendorffiiERV-A  297 HKIVT--NQFSVTDHFKA--VEGLNGRSLPGVFFFYELSPIKVLFTERKTSFLHFLTNVC
C.sativusERV-A3        297 RTVHS--NQYSVTEHFKS--VEFGSSQSIPGVFFYYDLSPVKVTYTEEHVPFLHFMTHIC
M.truncatulaERV-A2     297 RVIHS--NQYSVTEHFKS--SELGAA--VPGVFFFYDISPIKVNFKEEHIPFLHFLTNIC
P.vulgarisERV-A2       297 RVIHS--NQYSVTEHFKS--SELGVA--VPGVFFFYDISPIKVNFKEEHIPFLHFLTNVC
G.maxERV-A3            297 RVIHS--NQYSVTEHFKS--SELGVA--VPGVFFFYDISPIKVNFKEEHIPFLHFLTNIC
G.maxERV-A4            297 RVIHS--NQYSVTEHFKS--SELGVA--VPGVFFFYDISPIKVNFKEEHTPFLHFLTNIC
S.lycopersicumERV-A2   297 RTIDS--NQFSVTEHFKG--SDLGLFQSITGVYFFYDLSPIKVTFTEEHVSFFHFLTNVC
S.tuberosumERV-A2      297 RTIDS--NQFSVTEHFKG--SDLGLFQSITGVYFFYDLSPIKVTFTEEHVSFFHFLTNVC
P.persicaERV-A2        297 RTIRS--NQYSVTEHFKS--SELGHSQLLPGVFFFYDLSPIKVTLKEEHVPFLHFMTHIC
T.cacaoERV-A2          297 RTVHS--NQYSVTQHFKN--LEVIYPNSHPGVFFIYDFSPIKVTFKEEHISFLHFITNIC
P.trichocarpaERV-A3    297 RTVHS--NQYSVTEHFTK--SELMRLDSLPGVYFIYDFSPIKVTFKEEHTSFLHFMTSIC
P.trichocarpaERV-A4    297 RTVHS--NQYSATEHFKK--SELTPLDSLPGVYFFYDFSPIKVIFKEEHISFLHFMTSIC
C.sativusERV-A1        296 KAIKS--NQFSVTQHLRG--IDGESFQALHGVFFFYDLSPIKVTFTEEHISFFHFLTNVC
V.viniferaERV-A2       297 HTIQS--NQFSVTEHFRS--AEPGRPHSLPGVYFFYDLSPIKVTSKEEHSSFLHFMTNIC
B.rapaERV-A4           293 KTIQS--NQFSVTEHFQN--AEAGRMQSPPGVFFYYDLSPIKVIFEEQHVEFLHFLTNVC
B.rapaERV-A3           293 HTIQS--NQFSVTEHFQK--MEAGRMQSPPGVFFYYDLSPIKVIFEEQHVEFLHFLTNVC
E.salsugineumERV-A2    297 NTIQS--NQFSVTEHFQN--AEAGRMQSPPGVFFYYDLSPIKVIFEEQHVEFLHFLTNVC
```

```
                                                                      BLOCK#5------------------------
A.thalianaERV-A2          297 NTIQS--NQFSVTEHFQN--MEAGRMQSPPGVFFYYDLSPIKVIFEEQHVEFLHFLTNVC
C.rubellaERV-A2           297 NTIQS--NQFSVTEHFQG--MEAGRMQSPPGVFFYYDLSPIKVIFEEQHVEFLHFLTNVC
B.rapaERV-A2              297 HVIQS--NQFSVTEHFEK--TEAGRTQSLPGVFFFYDLSPIKVIFTEQHVEFLHFLTNVC
E.salsugineumERV-A3       297 HIIQS--NQFSVTEHFEK--TEAGHTQSLPGVFFFYDLSPIKVIFTEQHVEFLHFLTNVC
B.distachyonERV-A1        297 QIILS--NQFSVTEHARS--SESVRMQALPGVFFFYDLSPIKVTFTERHVSFLHFLTNVC
O.sativaERV-A1            297 HIILS--NQFSVTEHFRS--SESGRIQAVPGVFFFYDLSPIKVTFTEQHVSFLHFLTNVC
S.bicolorERV-A1           297 HIILS--NQFSVTEHFRS--GESGRMQALPGVFFFYDLSPIKVTFTEQHVSFLHFLTNVC
Z.maysERV-A1              297 HIILS--NQFSVTEHFRS--GESGRMQALPGVFFFYDLSPIKVTFTEQHVSFLHFLTNVC
S.lycopersicumERV-A1      297 HTIQT--NQFSVTEHFKG--ADFGRFQSIPGVFFFYDLSPIKVTFTEQHASFLHFLTNVC
S.tuberosumERV-A1         297 HTIQS--NQFSVTEHFQG--ADFGRFQSIPGVFFFYDLSPIKVTFTEQHVSFLHFLTNVC
B.rapaERV-A1              297 HTIQS--NQFSVTEHVKS--SEAGQLQSLPGVFFFYDLSPIKVTFTEEHISFLHFLTNVC
E.salsugineumERV-A1       297 HSIHS--NQFSVTEHVKS--SEAGQLQSLPGVFFFYDLSPIKVTFTEEHISFLHFLTNVC
A.thalianaERV-A1          297 HTIQS--NQFSVTEHVKS--SEAGQLQSLPGVFFFYDLSPIKVTFTEEHISFLHFLTNVC
C.rubellaERV-A1           297 HTIQS--NQFSVTEHVKS--SEAGQLQSLPGVFFFYDLSPIKVTFTEEHISFLHFLTNVC
V.viniferaERV-A1          297 HTIST--NQFSVTEHFRN--AELGRLQSLPGVFFFYDLSPIKVTFTEEHVSFLHFLTNVC
C.sativusERV-A2           297 YTIQS--NQFSVTEHVRT--AEVGRLQSLPAVFFFYDLSPIKVTFTEEHVSFLHFLTNVC
M.truncatulaERV-A1        297 NTIQS--NQFSVTEHFRT--ADVGRLQSLPGVFFFYDLSPIKVTFTEEHVSFLHFLTNVC
P.persicaERV-A1           297 HTIQS--NQFSVTEHFRG--TEVGNLQYLPGVFFFYDLSPIKVTFTEEHISFLHFLTNVC
P.vulgarisERV-A1          297 HAIQS--NQFSVTEHFRT--GDVGRLQSLPGVFFFYDLSPIKVTFTEEHVSFLHFLTNVC
G.maxERV-A1               297 HTIQS--NQFSVTEHFRT--GDVGRLQSLPGVFFFYDLSPIKVTFTEENVSFLHFLTNVC
G.maxERV-A2               297 HTIQS--NQFSVTEHFRT--GDMGRLQSLPGVFFFYDLSPIKVTFTEENVSFLHFLTNVC
T.cacaoERV-A1             297 HTIQS--NQFSVTEHFKG--AEINRLQSLPGVFFFYDLSPIKVTFTEQHVSFLHFLTNVC
P.trichocarpaERV-A1       297 HTIQS--NQFSVTEHFRG--TDIGRLQSLPGVFFFYDLSPIKVTFTEEHVSFLHFLTNVC
P.trichocarpaERV-A2       288 HTIQS--NQFSVTEHFRG--ADIGRLQSLPGVFFFYDLSPIKVTFTEEHVSFLHFLTNVC
C.reinhardtiiERV-B        259 RATET--HQYSVTEY-----AQPLQRGYAPAVDVHYDLSPIVMTINERPPSLLHFVVRLC
V.carteriERV-B            257 RVTET--HQYSVTEY-----TQPLEPGYVPTLDVHYDLSPIVMTINERPPSLLHFVVRLC
C.subellipsoideaERV-B     243 TRTTT--NQYSVTEYDTV--VHKGEMQ-MPSVWFSYDISPISVTISEIRKSFAHLLVRFC
K.flaccidumERV-B          267 DHVET--NQYSVTEYFMP---SSMQPGGLPAVYFLYDLSPIAVKVTERRRNFGHFLTRIC
P.patensERV-B             260 EVMPT--NQFSVTEYYQ---RTKPSDRSYPAVYFVYDLSPIVVTIREHRRNFGHFITRLC
S.moellendorffiiERV-B     251 GKLPT--NQFSVGEYYL---AARDDDISWPAVYFLYDLSPITVLIKEERRSFGHLLTRFC
S.bicolorERV-B            262 KVLPT--NQFSVTEYFLP-IRPSDRA--WPAVYFLYDLSPITVTIKEERRNFLHFITRLC
Z.maysERV-B               262 KVLPT--NQFSVTEYFLP-IRPTDRA--WPAVYFLYDLSPITVTIKEERRNFLHFVTRLC
B.distachyonERV-B         261 QVLPT--NQFSVTEYFVP-IRPADRS--WPAVYFLYDLSPITVTIKEERRNFLHFITRLC
O.sativaERV-B             261 QVLPT--NQFSVTEYFVP--KRATDRSAWPAVYFLYDLSPITVTIKEERRNFLHFLTRLC
B.rapaERV-B2              261 DILTT--NQYSVTEYYTP-MNEFDRT--WPAVYFLYDLSPITVTIKEERRSFLHLITRLC
B.rapaERV-B1              264 DVLTT--NQYSVTEYYTP-MNEFDRT--WPAVYFLYDLSPITVTIKEERRSFLHLITRLC
E.salsugineumERV-B        260 DVLST--NQYSVTEYYTP-MNEFDRT--WPAVYFLYDLSPITVTIKEERRSFLHLITRLC
A.thalianaERV-B           268 DVLST--NQYSVTEYFTP-MTEFDRT--WPAVYFLYDLSPITVTIKEERRSFLHLITRLC
C.rubellaERV-B            268 DVLST--NQYSVTEYYTP-MTDFDRT--WPAVYFLYDLSPITVTIKEERRSFLHLITRLC
M.truncatulaERV-B         269 EVLPT--NQFSVTEYFSPITSQFDRT--WPAVYFLYDLSPITVTIKEERRSFLHFITRLC
P.vulgarisERV-B           263 EVLPT--NQFSVSEYYSP-INQFDRT--WPAVYFLYDLSPITVTIKEERRSFLHFITRLC
G.maxERV-B1               266 EVLPT--NQFSVSEYYSP-INQFDRT--WPAVYFLYDLSPITVTIKEERRSFLHFITRLC
G.maxERV-B2               262 EVLPT--NQFSVSEYYSP-INQFDRT--WPAVYFLYDLSPITVTIKEERRSFLHFITRLC
C.sativusERV-B            260 AVLPT--NQFSVTEYFSP-MTDSDRS--WPAVYFLYDLSPITVTIKEERRSFLHFITRLC
S.lycopersicumERV-B1      260 EVLPT--NQFSVSEYFSP-INEFDRT--WPAVYFLYDLSPITVTIREERRNFLHFITRLC
S.tuberosumERV-B1         253 EVLPT--NQFSVSEYFSP-INEFDRT--WPAVYFLYDLSPITVTIREERRNFLHFITRLC
S.lycopersicumERV-B2      262 EVLPT--NQFSVTEYFSP-IHDFERT--WPAVYFLYDLSPITVTIREERRSFLHFITRLC
S.tuberosumERV-B2         262 EVSPT--NQFSVTEYFSP-IHDFERT--WPAVYFLYDLSPITVTIREERRSFLHFITRLC
P.persicaERV-B            263 EVLPT--NQFSVTEYFSP-MKQFDRT--WPAVYFLYDLSPITVTIKEERRSFLHFITRLC
P.trichocarpaERV-B1       263 EVLPT--NQFSVTEYFSP-MTDFDRT--WPAVYFLYDLSPITVTIKEERRSFLHFITRLC
P.trichocarpaERV-B2       263 DVLPT--NQFSVTEYFSP-ITDFDRT--WPAVYFLYDLSPITVTIKEERRSFLHFITRLC
V.viniferaERV-B           263 EVLPT--NQFSVMEYFSP-MNEFDRT--WPAVYFLYDLSPVTVTIKEERRSFLHFITRLC
T.cacaoERV-B              263 EVLPT--NQFSVSEYFSP-MHEFDRT--WPAVYFLYDLSPITVTIKEERRSFLHFITRLC
```

```
C.subellipsoideaPDI-C    437 AIIGGVFTVAGIVDGLVHTGARFAKK--VELGKHT
C.reinhardtiiPDI-C       442 AIIGGVFTVAGILDALLYQSFKVVKK--LNLGKQG
V.carteriPDI-C           442 AIIGGVFTVAGILDALLYQSFKVVKK--LNLGKQG
K.flaccidumPDI-C         491 AIIGGVFTVAGIVDGILFQAASAIKK--VQLGKQF
B.rapaPDI-C1             451 AIIGGVFTVAGILDSIFHNTIRLVKK--VELGKNF
B.rapaPDI-C2             452 AIIGGVFTVAGIIDSVLHNTIRLIKK--VELGKNI
E.salsugineumPDI-C1      451 AIIGGVFTVAGILDSIFHNTIRLVKK--VELGKNI
A.thalianaPDI12          451 AIIGGVFTVAGILDSIFHNTVRLVKK--VELGKNI
C.rubellaPDI-C1          451 AIIGGVFTVAGILDSIFHNTIRLIKK--VELGKNI
B.rapaPDI-C5             445 AIIGGVFTVAGILDSIFQSTYGIMKK--VELGKNF
A.thalianaPDI13          452 AIIGGVFTVAGILDSIFQNTVRMVKK--IELGKNI
C.rubellaPDI-C3          451 AIIGGVFTVAGILDSIFQNTVRLVKK--IELGKNI
B.rapaPDI-C6             451 AIIGGVFTVAGILDSIFQNTFRLVKK--IELGKNI
E.salsugineumPDI-C3      452 AIIGGVFTVAGILDSIFQNTIRLVKK--VELGKNI
S.bicolorPDI-C           453 AIIGGVFTVAGILDSIFHNTLRMVKK--VELGKNI
Z.maysPDI-C              451 AIIGGVFTVAGILDSIFHNTLRMVKK--IELGKNI
B.distachyonPDI-C        453 AIIGGVFTVAGILDSILHNTLRLVKK--VELGKDI
O.sativaPDI-C            453 AIIGGVFTVAGILDSIFHNTLRLVKK--VELGKNI
B.rapaPDI-C4             448 AIIGGVFTVAGILDSILHQTMTLMKK--IELGKNF
A.thalianaPDI7           448 AIIGGVFTVAGILDSILHHSMTLMKK--IELGKNF
C.rubellaPDI-C2          448 AIIGGVFTVAGILDSILHHTMTLMKK--IELGKNF
B.rapaPDI-C3             446 AIIGGVFTVAGILDSILHQTMTLMKK--IELGKNF
E.salsugineumPDI-C2      448 AIIGGVFTVAGILDSILHQTMTLMKK--IELGKNF
S.lycopersicumPDI-C      449 AIIGGVFTVAGILDSILHNTMRMVKK--VELGKNF
S.tuberosumPDI-C         449 AIIGGVFTVAGILDSILHNTMRMVKK--VELGKNF
M.truncatulaPDI-C1       448 AIVGGVFTVAGITESILHNTIRLMRK--VELGKNF
P.vulgarisPDI-C1         448 AIIGGVFTVAGILDSILHNTIRLMKK--VELGKNF
G.maxPDI-C1              448 AIVGGIFTVAGIMDAILHNTIRLMKK--VELGKNF
G.maxPDI-C2              448 AIIGGIFTVAGIMDAIFHNTIRLMKK--VELGKNF
M.truncatulaPDI-C2       445 AIIGGVFTVAGILDSILHNTIKAMKK--IEIGKNF
P.vulgarisPDI-C2         448 AIIGGIFTVAGILDSILHNTIRMMKK--VELGKNF
G.maxPDI-C3              448 AIIGGVFTVAGILDSILHNTIRMVKK--IELGKNF
G.maxPDI-C4              448 AIIGGVFTVAGILDSILHNTIRMMKK--VELGKNF
V.viniferaPDI-C          450 AIIGGVFTVAGILDSVLHNTMRLMKK--IELGKNF
C.sativusPDI-C           449 AIIGGVFTVAGILDALLHNTIRLMKK--VELGKNF
P.persicaPDI-C           448 AIIGGVFTVAGIVDSILHNTIRMMRK--VELGKNF
T.cacaoPDI-C             448 AIIGGVFTVAGILDSILHNTIRLMKK--VELGKNF
P.trichocarpaPDI-C1      452 AIIGGVFTVAGILDSILHHTVRMMKK--VELGKNF
P.trichocarpaPDI-C2      452 AIIGGVFTVAGILDSILHNTFRMMKK--VELGKNF
S.moellendorffiiPDI-C    443 AIIGGVFTVAGIVDSMLHGAMRMVKK--IELGKQF
P.patensPDI-C            454 AIIGGVFTVAGIIDSMLHNAMHIMKK--VELGKQY
C.reinhardtiiERV-A       392 AIVGGVFTVSGIVDAFVYTGTRMIKK-KMELGKFS
V.carteriERV-A           359 AIVGGVFTVSGIVDAFIYTSTRLIRK-KMELGKFS
C.subellipsoideaERV-A    353 AIVGGIFTVAGIVDAFIYHGHQAIKK-KVDLGKQI
K.flaccidumERV-A         353 AIVGGVFTVSGIIDAFVYHGHKVIKK-KMELGKLN
B.distachyonERV-A3       351 AIVGGIFTVSGIIDSFIYHGQKALKK-KMEIGKYR
O.sativaERV-A3           351 AIVGGVFTVSGIIDSFIYHGQKALKK-KMELGKYR
Z.maysERV-A3             351 AIVGGVFTVSGIIDSFIYHGQKALKK-KMELGKYR
Z.maysERV-A2             351 AIVGGVFTVSGIIDSFIYHGQKALKK-KMELGKYR
S.bicolorERV-A3          351 AIVGGVFTVSGIIDSFIYHGQKALKK-KMELGKYR
P.patensERV-A1           355 AIVGGVFSVSGIIDAFVYHGQKQIKK---RLGKDT
P.patensERV-A4           352 AIVGGVYTVASIIDSFIYHGHRAVKK-KMELGKFS
P.patensERV-A2           352 AIVGGVYSIAGIIDSFVYHGHRAIKK-KMELGKLS
P.patensERV-A3           352 AIIGGIYTIAGIVDSFIYHGHRAIKK-KMELGKLS
S.bicolorERV-A2          354 AIVGGIFTVAGIIDSFVYHGHRAIKK-KMELGKLG
B.distachyonERV-A2       354 AIVGGIFTVAGIIDSFVYHGHRAIKK-KMEIGKLG
O.sativaERV-A2           354 AIVGGIFTVAGIIDSFVYHGHRAIKK-KMEIGKLG
S.moellendorffiiERV-A    353 AIIGGVFTVSGIIDSFIYHGHRAIKK-KMEIGKYI
C.sativusERV-A3          353 AIIGGVFSVAGIIDAFIYHGQRKMKK-KVEIGKFG
M.truncatulaERV-A2       351 AIIGGIFTIAGIVDSSIYYGQKTIKK-KMEIGKYR
P.vulgarisERV-A2         351 AIIGGIFTVAGIIDSSIYYGQRTIKK-KMEIGKYT
G.maxERV-A3              351 AIIGGVFTVAGIIDSSIYYGQRTIKR-KMELGKFT
G.maxERV-A4              351 AIIGGVFTVAGIIDSSIYYGQRTIKR-KMELGKFT
S.lycopersicumERV-A2     353 AIIGGVFTVAGILDACIYHSQKAIKK-KVELGKFG
S.tuberosumERV-A2        353 AIIGGVFTVAGILDACIYHSQKAIKK-KVELGKFG
P.persicaERV-A2          353 AIVGGIFTVAGLIDSFVYHGQRAMKK-KMEIGKFG
T.cacaoERV-A2            353 AVIGGIFTVAGIIDSFVYHGQRRMKK-KMEIGKFR
P.trichocarpaERV-A3      353 AIIGGIFTIAGIVDSFIYHGRRAIKK-KMEIGKFS
P.trichocarpaERV-A4      353 AIIGGIFTIAGIIDSFIYYGQRAITK-KVGIGKFG
C.sativusERV-A1          352 AIVGGVFTISGILDSIIYHGQKAIKK-KMALGKFT
V.viniferaERV-A2         353 AIVGGIFTVSGIIDSFVYHGHRAIKK-KMELGKFS
B.rapaERV-A4             349 AIVGGIFTVSGIVDSFIYHGQRAIKK-KMEIGKFN
B.rapaERV-A3             349 AIVGGIFTVSGIVDSFIYHGQRAIKK-KMEIGKFN
E.salsugineumERV-A2      353 AIVGGVFTVSGIVDSFIYHGQRAIKK-KMEIGKFN
```

```
A.thalianaERV-A2           353 AIVGGIFTVSGIVDSFIYHGQRAIKK-KMEIGKFN
C.rubellaERV-A2            353 AIVGGIFTVSGILDSFIYHGQRAIKK-KMEIGKFN
B.rapaERV-A2               353 AIVGGIFTVSGIIDSFVYHGQRAIKK-KMEIGKFG
E.salsugineumERV-A3        353 AIVGGIFTVSGIIDSFIYHGQRAIKK-KMEIGKFG
B.distachyonERV-A1         353 AIVGGVFTVSGIIDSFVYHGQRAITK-KREIGKFN
O.sativaERV-A1             353 AIVGGVFTVSGIIDSFVYHGQRAIKK-KMEIGKFN
S.bicolorERV-A1            353 AIVGGVFTVSGIIDSFVYHSQRAIKK-KMEIGKFN
Z.maysERV-A1               353 AIVGGVFTVSGIIDSFVYHSQRAIKK-KMEIGKFN
S.lycopersicumERV-A1       353 AIVGGVFTVSGILDSFIYHGQKAIKK-KMELGKFS
S.tuberosumERV-A1          353 AIVGGVFTVSGILDSFIYHGQKAIKK-KMELGKFS
B.rapaERV-A1               353 AIVGGVFTVSGIIDAFIYHGQKAIKK-KMEIGKFS
E.salsugineumERV-A1        353 AIVGGVFTVSGIIDAFIYHGQKAIKK-KMEIGKFS
A.thalianaERV-A1           353 AIVGGVFTVSGIIDAFIYHGQKAIKK-KMEIGKFS
C.rubellaERV-A1            353 AIVGGVFTVSGIIDAFIYHGQKAIKK-KMEIGKFN
V.viniferaERV-A1           353 AIVGGVFTVSGILDSFIYHSQKAIKK-KIEIGKFS
C.sativusERV-A2            353 AIVGGVFTVSGILDSFIYHGQKVIKK-KMEIGKFS
M.truncatulaERV-A1         353 AIVGGIFTVSGILDSFIYHGQKAIKK-KMELGKFS
P.persicaERV-A1            353 AIVGGVFTVSGILDSFIYHGQKAIKK-KMEIGKFS
P.vulgarisERV-A1           353 AIVGGIFTVSGILDSFIYHGQRAIKK-KMELGKFN
G.maxERV-A1                353 AIVGGIFTVSGILDSFIYHGQRAIKK-KMELGKFN
G.maxERV-A2                353 AIVGGIFTVSGILDSFIYHGQRAIKK-KMELGKFN
T.cacaoERV-A1              353 AIVGGVFTVSGILDSFIYHGQRAIKK-KIEIGKYS
P.trichocarpaERV-A1        353 AIVGGVFTVSGILDTFIYHGQKAIKK-KMEIGKFS
P.trichocarpaERV-A2        344 AIVGGVFTVSGILDSFIYHGQKAIKK-KMEIGKFS
C.reinhardtiiERV-B         312 AVVGGVFAITRLTDRWVDWLVRLVNK-AAARGP--
V.carteriERV-B             310 AVVGGAFAITRMTDRWVDWFVRLVTK-------LK
C.subellipsoideaERV-B      298 AVVGGVFAVTGMFDRWVHRIVTAIFS------ASS
K.flaccidumERV-B           322 AVLGGTFAVTGMIDKWTYKTVQLFTS-QRSSGSQ-
P.patensERV-B              315 AVLGGTFAVTGMLDRWMSRIIDFVMS--TSKQGFL
S.moellendorffiiERV-B      306 AIVGGTFSLTGMLDRWIYRLVESITR---AKGVLI
S.bicolorERV-B             317 AVLGGTFAMTGMLDRWMYRLIESVTN-SKTRSVLR
Z.maysERV-B                317 AVLGGTFAMTGMLDRWMYQLIKTVTN-SKTRSVLR
B.distachyonERV-B          316 AVLGGTFAMTGMLDRWMYRIIESVSS-SKPRSVLR
O.sativaERV-B              317 AVLGGTFAMTGMLDRWMYRLIESVTK-SKTRSVLR
B.rapaERV-B2               316 AVLGGTFALTGMLDRWMFRIIESFTKKPSTRSIHK
B.rapaERV-B1               319 AVLGGTFALTGMLDRWMFRLIESFTKKPSTRSIHK
E.salsugineumERV-B         315 AVLGGTFALTGMLDRWMFRFIESFTKKPSTRSIHK
A.thalianaERV-B            323 AVLGGTFALTGMLDRWMFRFIESFNKKPSTRA---
C.rubellaERV-B             323 AVLGGTFALTGMLDRWMFRLIESFNK-NTNTRSMH
M.truncatulaERV-B          325 AVLGGTFAVTGMLDRWMYRLVEAATK-PKNKK---
P.vulgarisERV-B            318 AVLGGTFAVTGMLDRWMYRLIEALTK-SKSKR---
G.maxERV-B1                321 AVLGGTFAVTGMLDRWMYRLLEALTK-SKSKR---
G.maxERV-B2                317 AVLGGTFAVTGMLDRWMYRLLETLTK-SKSKR---
C.sativusERV-B             315 AVLGGTFAVTGMLDRWMFRFLEALTK---PKRRTR
S.lycopersicumERV-B1       315 AVLGGTFALTGMLDKWMFRFLEAVMK-KNSRSLVR
S.tuberosumERV-B1          308 AVLGGTFALTGMLDKWMFRFLEAVMK-KDSRGLVR
S.lycopersicumERV-B2       317 AVLGGTFALTGMLDGWMYKILESVTK-KNSRTIVR
S.tuberosumERV-B2          317 AVLGGTFALTGMLDGWMYKILESFTK-KNSRTIVR
P.persicaERV-B             318 AVLGGTFALTGMLDRWMYRFLEAVTK-PNARSVLR
P.trichocarpaERV-B1        318 AVLGGTFALTGMLDRWMCRLLEALTK-PNPRSVLR
P.trichocarpaERV-B2        318 AILGGTFALTGMLDRWMYRLLEALTK-PNRGSGFL
V.viniferaERV-B            318 AVLGGTFALTGMLDRWMYRFLEMLTK-PNAKSVYR
T.cacaoERV-B               318 AVLGGTFALTGMLDRWMFRLIEGLTK-PSHKGVLR
```
