# Supplementary figures and images for: Phylogenetic characterization and promoter expression analysis of a novel hybrid protein disulfide isomerase/cargo receptor subfamily unique to plants and chromalveolates
Source: Mol Genet Genomics. 2015 Aug 25;291:455–69. doi: 10.1007/s00438-015-1106-7 (PMC4729789; doi:10.1007/s00438-015-1106-7)

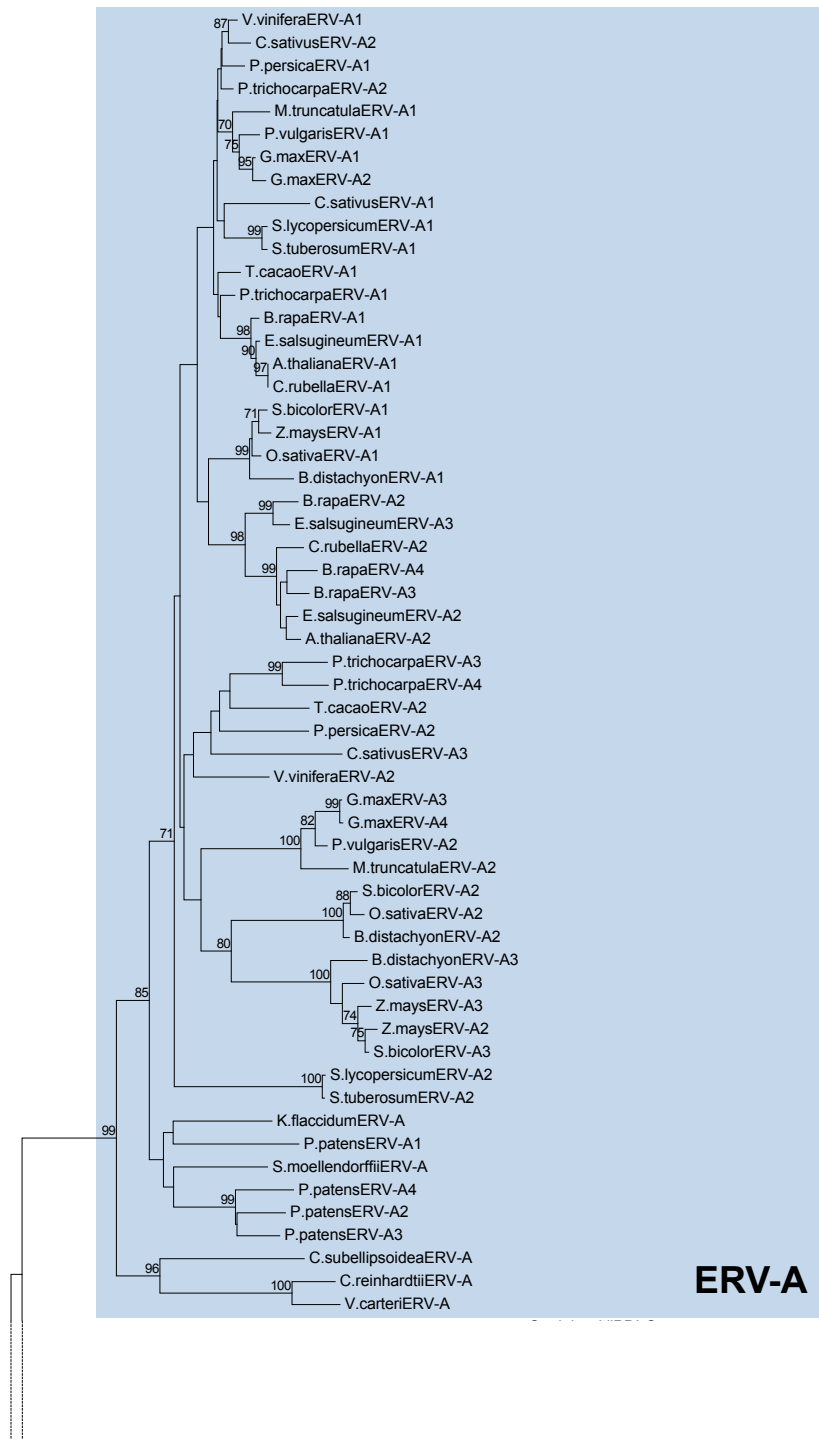

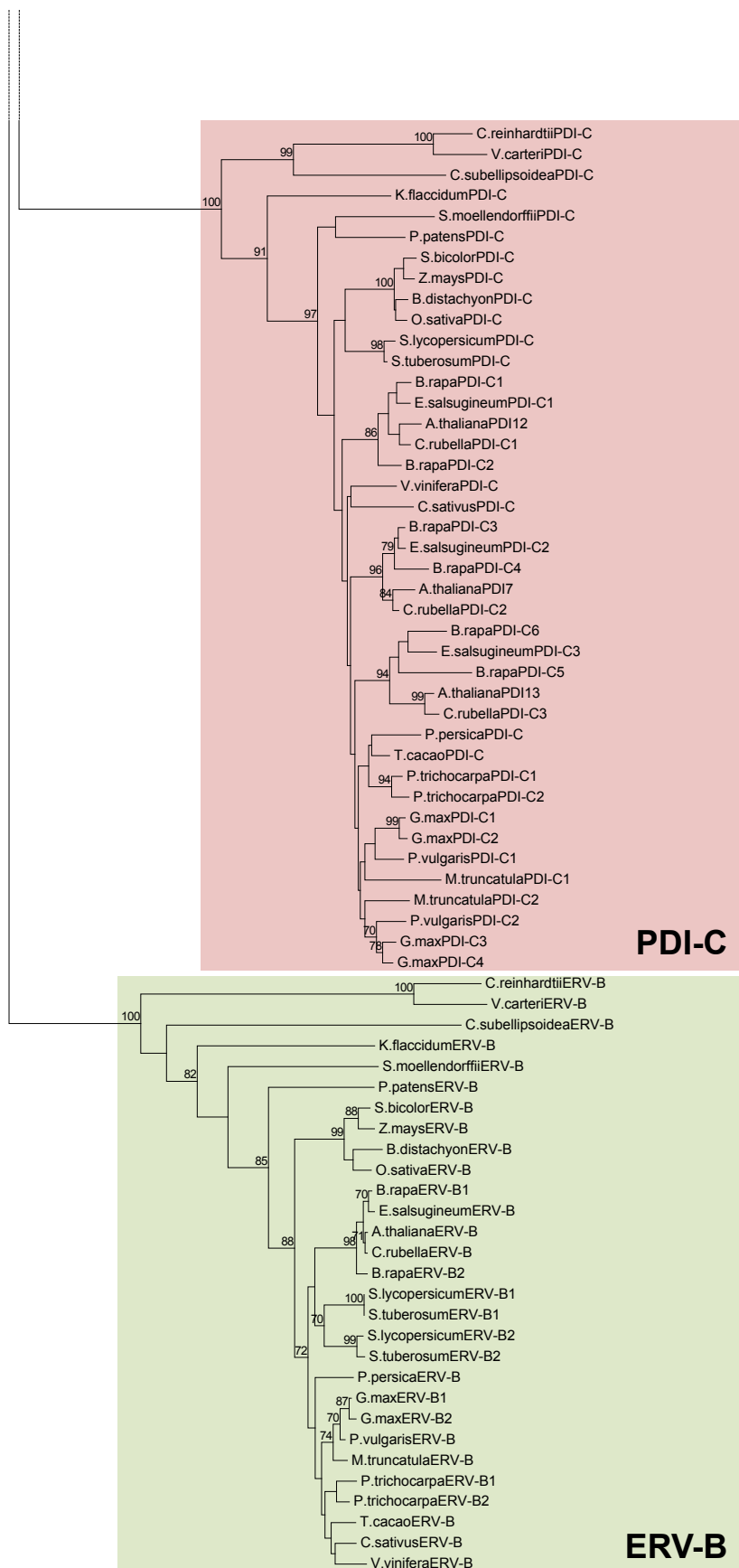

Supplement: Supplementary file 4 — Online Resource 4. Phylogenetic analysis of all plant Erv41p/Erv46p homologs surveyed in this study. The unrooted NJ tree was generated with evolutionary distances computed using the Poisson correction method. The tree is drawn to scale, with branch lengths proportional to the number of amino acid substitutions per site. NJ analysis was performed using a Gblocks-trimmed multiple sequence alignment consisting of 173 positions. Support values are shown above the branches, and are calculated from 1000 bootstrap replicates. Only bootstrap values ≥70 % are shown. The ERV-A clade is shaded in blue, the ERV-B clade in green, and the PDI-C clade in red (PDF 74 kb) [file 438_2015_1106_MOESM4_ESM.pdf]
