# Supplementary material for: Phylogenetic characterization and promoter expression analysis of a novel hybrid protein disulfide isomerase/cargo receptor subfamily unique to plants and chromalveolates
Source: Mol Genet Genomics. 2015 Aug 25;291:455–69. doi: 10.1007/s00438-015-1106-7 (PMC4729789; doi:10.1007/s00438-015-1106-7)
Supplement: Supplementary file 5 — Online Resource 5. Secondary structure predictions of yeast Erv41p and Erv46p and their Arabidopsis homologs. The multiple sequence alignment was generated by MUSCLE. Secondary structure predictions were performed with Jpred4. The Pfam-defined boundaries of the ERGIC-N domain, thioredoxin domain, and COPII-coated Erv domain are boxed in blue, red and green, respectively. The predicted all-beta regions of the ERGIC-N and COPII-coated Erv domains are boxed in orange. The positions of the 9 conserved Cys residues of ERV-A proteins are indicated above the alignment. H: helix, E: β-strand (PDF 146 kb) [file 438_2015_1106_MOESM5_ESM.pdf]

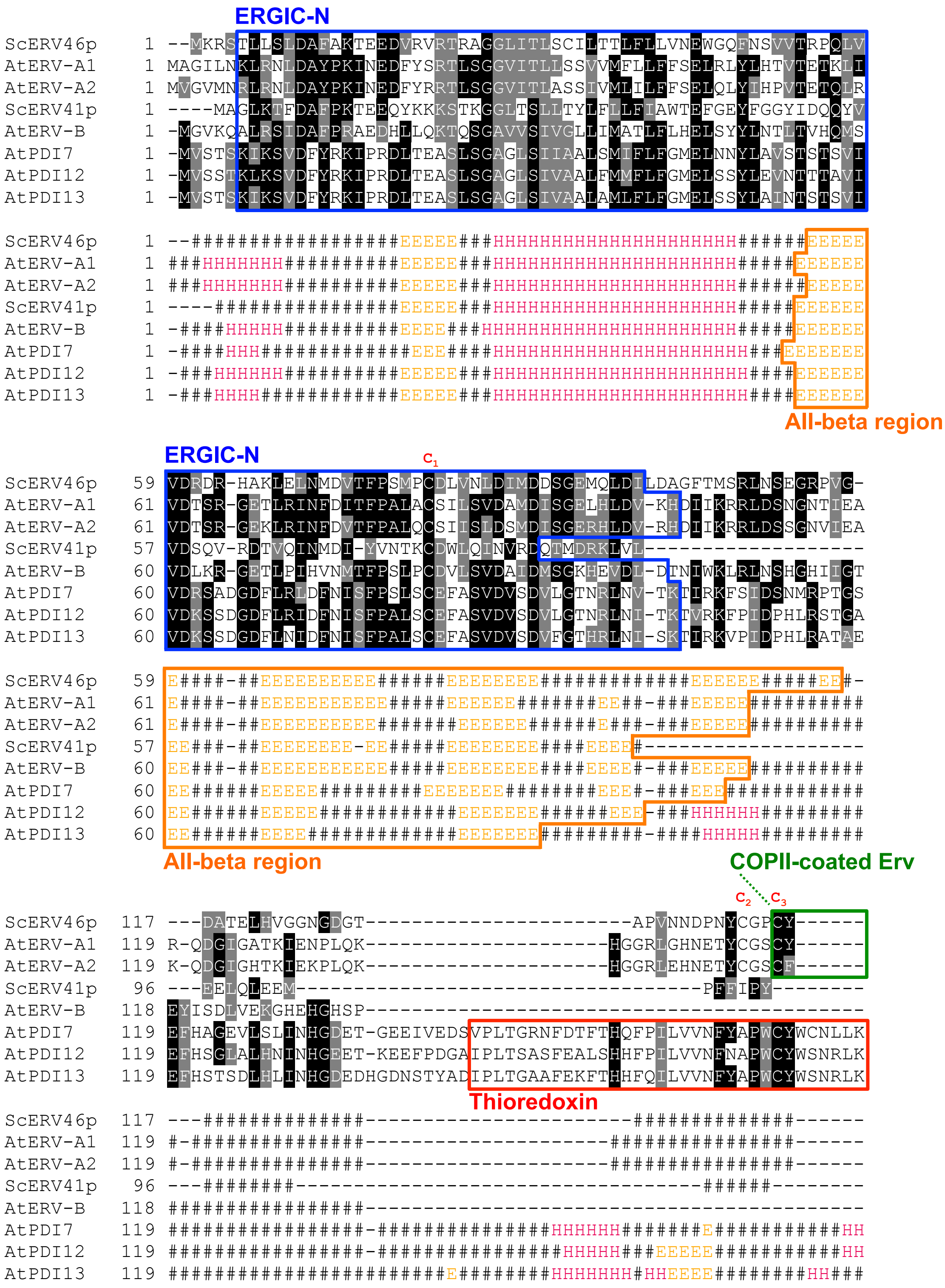

ERGIC-N
ScERV46p
AtERV-A1
AtERV-A2
ScERV41p
AtERV-B
AtPDI7
AtPDI12
AtPDI13
All-beta region
C_1
COPII-coated Erv
C_2
C_3
Thioredoxin

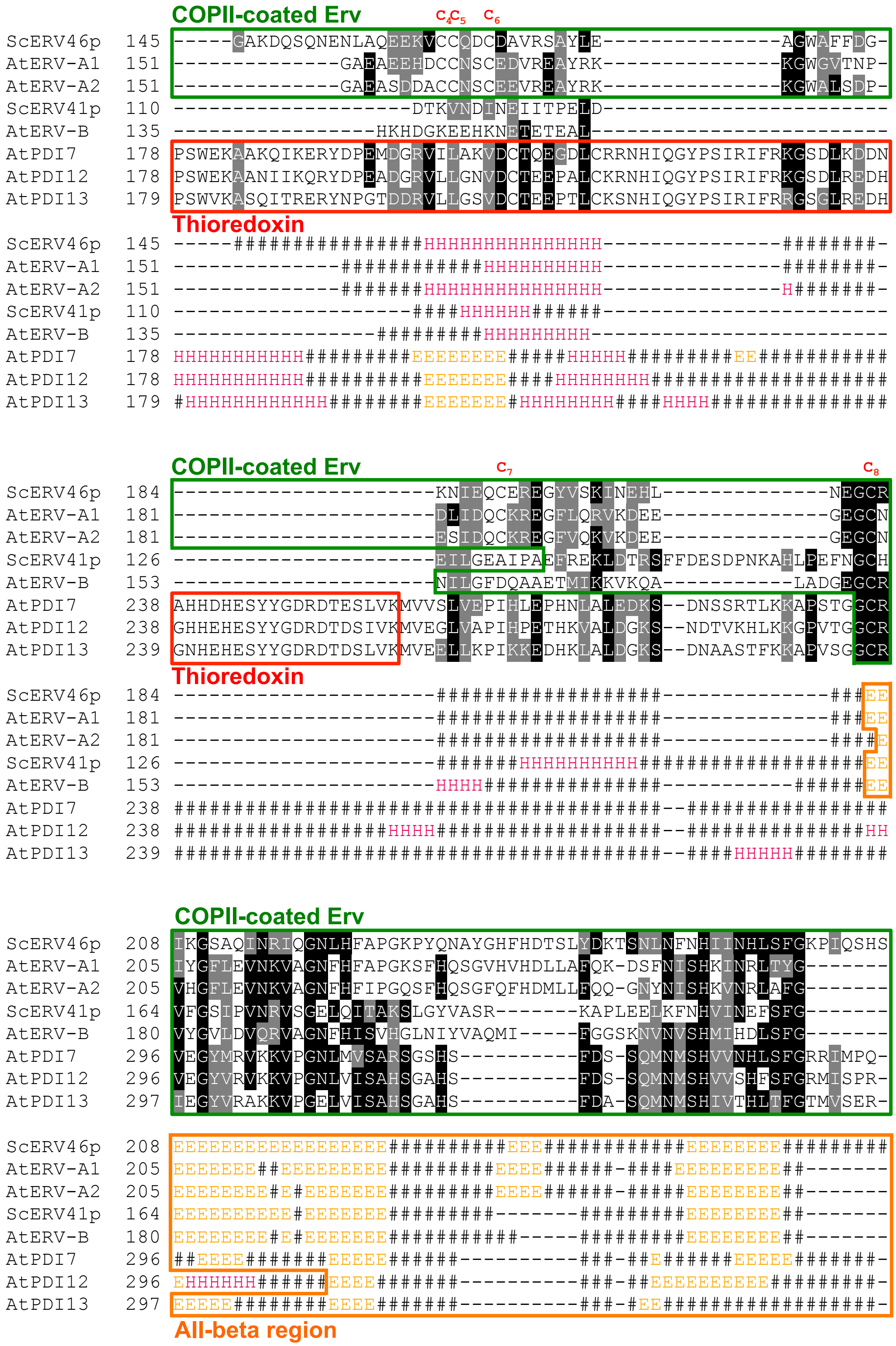

COPII-coated Erv
C4C5
C6
ScERV46p 145 -----GAKDQSQNENLAQEEKVCCQDCDAVRSAYLE----------------AGWAFFDG-
AtERV-A1 151 ---------------GAEAEEHDCCNSCEDVREAYRK----------------KGWGVTNP-
AtERV-A2 151 ---------------GAEASDDACCNSCEEVREAYRK----------------KGWALSDP-
ScERV41p 110 ---------------------DTKVNDINEIITPELD-------------------------
AtERV-B 135 -----------------HKHDGKEEHKNETETEAL---------------------------
AtPDI7 178 PSWEKAAKQIKERYDPEMDGRVIIAKVDCTQEGDLCRRNHIQGYPSIRIFRKGSDLKDDN
AtPDI12 178 PSWEKAANIIKQRYDPEADGRVLLGNVDCTEEPALCKRNHIQGYPSIRIFRKGSDLREDH
AtPDI13 179 PSWVKASQITRERYNPGTDDRVLLGSVDCTEEPTLCKSNHIQGYPSIRIFRRGSGLREDH
Thioredoxin
ScERV46p 145 -----###############HHHHHHHHHHHHHHH----------------########-
AtERV-A1 151 ---------------############HHHHHHHHHH----------------########-
AtERV-A2 151 ---------------#######HHHHHHHHHHHHHHH---------------H#######-
ScERV41p 110 ---------------------####HHHHHH######-------------------------
AtERV-B 135 -----------------#########HHHHHHHHH---------------------------
AtPDI7 178 HHHHHHHHHHH#########EEEEEEEE#####HHHHH#########EE###########
AtPDI12 178 HHHHHHHHHHH##########EEEEEEE####HHHHHHHH###################
AtPDI13 179 #HHHHHHHHHHHH#######EEEEEEE#HHHHHHHH####HHHH###############
COPII-coated Erv
C7
C8
ScERV46p 184 ----------------------KNIEQCEREGYVSKINEHL--------------NEGCR
AtERV-A1 181 ----------------------DLIDQCKREGFLQRVKDEE--------------GEGCN
AtERV-A2 181 ----------------------ESIDQCKREGFVQKVKDEE--------------GEGCN
ScERV41p 126 ----------------------EIILGEAIPAEFREKLDTRSFFDESDPNKAHLPEFNGCH
AtERV-B 153 ----------------------NILGFDQAAETMIKKVKQA-----------LADGEGCR
AtPDI7 238 AHHDHESYYGDRDTESLVKMVVSLVEPIHLEPHNLALEDKS--DNSSRTLKKAPSTGGCR
AtPDI12 238 GHHEHESYYGDRDTDSIVKMVEGLVAPIHPETHKVALDGKS--NDTVKHLKKGPVTGGCR
AtPDI13 239 GNHEHESYYGDRDTDSLVKMVEELLKPIKKEDHKLALDGKS--DNAASTFKKAPVSGGCR
Thioredoxin
ScERV46p 184 ----------------------###################--------------###EE
AtERV-A1 181 ----------------------###################--------------###EE
AtERV-A2 181 ----------------------###################--------------####E
ScERV41p 126 ----------------------#######HHHHHHHHHH##################EE
AtERV-B 153 ----------------------HHHH###############-----------######EE
AtPDI7 238 ###--#################
AtPDI12 238 #################HHHH####################--###############HH
AtPDI13 239 ###--####HHHHH########
COPII-coated Erv
ScERV46p 208 IKGSAQINRIQGNLHFAPGKPYQNAYGHFHDTSLYDKTSNLNFNHIINHLSFGKPIQSHS
AtERV-A1 205 IYGFLEVNKVAGNFHFAPGKSFHQSGVHVHDLLAFQK-DSFNISHKINRLTYG-------
AtERV-A2 205 VHGFLEVNKVAGNFHFIPGQSFHQSGFQFHDMLLFQQ-GNYNISHKVNRLAFG-------
ScERV41p 164 VFGSIPVNRVSGELQITAKSLGYVASR-------KAPLEEIKFNHVINEFSFG-------
AtERV-B 180 VYGVLDVQRVAGNFHISVHGLNIYVAQMI-----FGGSKNVNVSHMIHDLSFG-------
AtPDI7 296 VEGYMRVKKVPGNLMVSARSGSHS----------FDS-SQMNMSHVVNHLSFGRRIMPQ-
AtPDI12 296 VEGYVRVKKVPGNLVISAHSGAHS----------FDS-SQMNMSHVVSHFSFGRMISPR-
AtPDI13 297 IEGYVRAKKVPGELVISAHSGAHS----------FDA-SQMNMSHIVTHLTFGTMVSER-
ScERV46p 208 EEEEEEEEEEEEEEEEEE##########EEE############EEEEEEEE#########
AtERV-A1 205 EEEEEEE##EEEEEEEEE#########EEEE######-####EEEEEEEEE##-------
AtERV-A2 205 EEEEEEEE#E#EEEEEEE#########EEEE######-#####EEEEEEEE##-------
ScERV41p 164 EEEEEEEEEE#EEEEEEE#########-------#########EEEEEEEE##-------
AtERV-B 180 EEEEEEEE#E#EEEEEEE###########-----#########EEEEEEEE##-------
AtPDI7 296 ##EEEE########EEEEE######----------###-##E######EEEEE#######-
AtPDI12 296 EHHHHHH######EEEE#######----------###-##EEEEEEEEEE#########-
AtPDI13 297 EEEEE########EEEE#######----------###-#EE###################-
All-beta region

COPII-coated Erv

```
ScERV46p  268 KLLGNDKRHGGAVVATSPLDGRQVFPDRNTHFH-QFSYFAKIVPTRY--EYLDNVV----
AtERV-A1  257 ------DYFPGVV---NPLDKVEW-SQDTPNA--MYQYFIKVVPTVY--TDIRGHT----
AtERV-A2  257 ------DFFPGVV---NPLDGVQWNQGKQSG---VYQYFIKVVPSIY--TDVHQNT----
ScERV41p  210 ------DFYPYID---NPLDNTAQFNQDEPLT--TYVYYTSVVPTLF--KKLGAEVDTNQ
AtERV-B   228 ------PKYPGIH---NPLDDTNRILHDTSG---TFKYYIKIVPTEY--RYLSKDV----
AtPDI7    344 KFSEFKRLSPYLGLSHDRLDGRSFINQRDLGPNVTIEHYLQIVKTEV--VKSNGQA----
AtPDI12   344 LLTDMKRLLPYLGLSHDRLDGKAFINQHEFGANVTIEHYLQTVKTEVITRRSGQEH----
AtPDI13   345 LWTDMKRLLPYLGQSYDRLNGKSFINERQLDANVTIEHYLQIIKTEVISRRSGQEH----

ScERV46p  268 ######################EEEE######E-EEEEEEEEEEEEE--EE####E----
AtERV-A1  257 ------#######---######EE-E######--EEEEEEEEEEEEE--EE####E----
AtERV-A2  257 ------#######---######EE#######---EEEEEEEEEEEEE--EE####E----
ScERV41p  210 ------#######---######EEEE######--EEEEEEEEEEEEE--EE###EEEEEE
AtERV-B   228 ------#######---######EEEE#####---EEEEEEEEEEEEE--EE####E----
AtPDI7    344 #########################EEE######EEEEEEEEEEEEE--E#####E----
AtPDI12   344 ######################EEEE########EEEEEEEEEEEEEE#######E----
AtPDI13   345 ######################EEE########EEEEEEEEEEEEEEE######EE----
```

All-beta region

COPII-coated Erv

```
ScERV46p  321 IETAQFSATFHSRPLAGGRDKDHPNTLHVRGGIPGMFVFFEMSPLKVINKEQHGQTWSGF
AtERV-A1  299 IQSNQFSVTEHVKSSEAGQLQS----------LPGVFFFYDLSPIKVTFTEEH-ISFLHF
AtERV-A2  299 IQSNQFSVTEHFQNMEAGRMQS----------PPGVFFYYDLSPIKVIFEEQH-VEFLHF
ScERV41p  257 YSVNDYRYLYKDVAAKGDK-------------MPGIFFKYNFEPLSIVVSDVR-LSFIQF
AtERV-B   270 LSTNQYSVTEYFTPMTEFD-RT----------WPAVYFLYDLSPITVTIKEER-RSFLHL
AtPDI7    398 -LVEAYEYTAHSSVAHSYY-------------LPVAKFHFELSPMQVLITENS-KSFSHF
AtPDI12   400 SLIEEYEYTAHSSVAQTYY-------------LPVAKFHFELSPMQILITENP-KSFSHF
AtPDI13   401 SLIEEYEYTAHSSVARSYH-------------YPEAKFHFELSPMQVLISENP-KSFSHF

ScERV46p  321 EEEEEEEEEEEEEE###################EEEEEEEE###EEEEEEEE####HHHH
AtERV-A1  299 EEEEEEEEEEEEEE########----------EEEEEEEEE##EEEEEEEEE#-##HHHH
AtERV-A2  299 EEEEEEEEEEEEEE########---------#EEEEEEEEE#EEEEEEEEE#-##HHHH
ScERV41p  257 EEEEEEEEE##########-------------#EEEEEEEE##EEEEEEEEE#-##HHHH
AtERV-B   270 EEEEEEEEEEEEEE#####-##----------#EEEEEEEE###EEEEEEEE#-##HHHH
AtPDI7    398 -EEEEEEEEEEE#######-------------#EEEEEEEEE#EEEEEEEE##-##HHHH
AtPDI12   400 EEEEEEEEE##########-------------##EEEEEEE##EEEEEEEEE#-##HHHH
AtPDI13   401 EE#EEEEEEE#########-------------##EEEEEEEEEEEEEEEEEE#-##HHHH
```

All-beta region

COPII-coated Erv

C_9

```
ScERV46p  381 ILNCITSIG---------------GVLAVGTVMDKLFYKAQRSIWGKKSQ----
AtERV-A1  348 LTNVCAIVG---------------GVFTVSGIIDAFIYHGQKAIKKKMEIGKFS
AtERV-A2  348 LTNVCAIVG---------------GIFTVSGIVDSFIYHGQRAIKKKMEIGKFN
ScERV41p  303 LVRLVAICSFLVYCASWIFTLLDMALITIMGPKWSLRYQPDDKTKGILDR----
AtERV-B   318 ITRLCAVLG---------------GTFALTGMLDRWMFRFIESFNKKPSTRA--
AtPDI7    443 ITNVCAIIG---------------GVFTVAGILDSILHHSMTLMK-KIELGKNF
AtPDI12   446 ITNLCAIIG---------------GVFTVAGILDSIFHNTVRLVK-KVELGKNI
AtPDI13   447 ITNVCAIIG---------------GVFTVAGILDSIFQNTVRMVK-KIELGKNI

ScERV46p  381 HHHHHHHHH---------------HHHHHHHHHHHHHHHHHHHHHHH###----
AtERV-A1  348 HHHHHHHHH---------------HHHHHHHHHHHHHHHHHHHHHHHHH#####
AtERV-A2  348 HHHHHHHHH---------------HHHHHHHHHHHHHHHHHHHHHHHHH#####
ScERV41p  303 HHHHHHHHHHHHHHHHHHHHHHHHHHHHHHHHHHHH##############----
AtERV-B   318 HHHHHHHHH---------------HHHHHHHHHHHHHHHHHHHHHH######--
AtPDI7    443 HHHHHHHHH---------------HHHHHHHHHHHHHHHHHHHHH-HHHH####
AtPDI12   446 HHHHHHHH#---------------#HHHHHHHHHHHHHHHHHHHH-HHHH####
AtPDI13   447 HHHHHHHHH---------------HHHHHHHHHHHHHHHHHHHHH-HHHH####
```
